# Engineered C–N Lyases for Stereoselective Synthesis of Tertiary Amines

**DOI:** 10.1002/anie.202507311

**Published:** 2025-07-04

**Authors:** Laura Bothof, Xiaofang Gong, Marrit E. Onclin, Peter Fodran, Gerrit J. Poelarends

**Affiliations:** ^1^ Department of Chemical and Pharmaceutical Biology Groningen Research Institute of Pharmacy University of Groningen Antonius Deusinglaan 1 Groningen 9713 AV The Netherlands; ^2^ Present address: Department of Bioproducts and Biosystems School of Chemical Engineering Aalto University Espoo 02150 Finland

**Keywords:** Asymmetric synthesis, Biocatalysis, C–N lyase, *N*‐functionalized amino acids, Tertiary amines

## Abstract

Optically pure *N*‐functionalized α‐amino acids are valuable chiral building blocks for pharmaceuticals, nutraceuticals, and agrochemicals. Ethylenediamine‐*N,N*‐disuccinic acid lyase from *Chelativorans* sp. BNC1 catalyzes the addition of a wide range of aliphatic and aromatic primary amines to fumarate, producing the corresponding enantioenriched *N*‐substituted *L‐*aspartic acids. In this work, the enzyme was subjected to iterative cycles of site‐saturation mutagenesis and screened for increased activity for the addition of 2‐((methylamino)methyl)aniline to fumarate. The final variant displayed an activity of three orders of magnitude higher compared to the wild‐type enzyme. Unexpectedly, the enzyme catalyzed the hydroamination of fumarate with the aliphatic secondary amine of the starting substrate, rather than with the aromatic primary amine, leading to the formation of a tertiary amine. Exploring the substrate scope showed that the enzyme accepts various substituted *N*‐methyl‐1‐phenylmethanamines for the hydroamination of fumarate, yielding *N,N*‐disubstituted *L*‐aspartic acids in high optical purity (up to >99% ee). Furthermore, we showed that the enzyme accepts several *ortho*‐substituted anilines that were previously not accepted by the wild‐type enzyme, yielding the corresponding *N*‐arylated *L*‐aspartic acids in high enantiomeric excess (>99% ee). This serendipitous finding enables a new strategy for the biocatalytic synthesis of tertiary amines, unlocked within the C‐N lyase toolbox.

## Graphical abstract

EDDS lyase was subjected to iterative cycles of site‐saturation mutagenesis and screened for increased activity for the addition of 2‐((methylamino)methyl)aniline to fumarate. The final mutant, EDDS lyase CEA, accepts various *N*‐methyl‐1‐phenylmethanamines derivatives, yielding *N,N*‐disubstituted *L*‐aspartic acids, as well as several *ortho*‐substituted anilines, producing *N*‐arylated *L*‐aspartic acids.

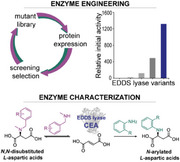

Optically pure *N*‐functionalized α‐amino acids and their derivatives are valuable chiral building blocks for active pharmaceutical ingredients (APIs).^[^ ^1^ ^]^ For instance, *N*‐aryl‐ and *N*‐benzyl‐substituted α‐amino acids are part of the core structures of various pharmaceutically relevant compounds, such as Farglitazar,^[^ ^2^, ^3^, ^4^ ^]^ Lotrafiban,^[^ ^5^, ^6^, ^7^ ^]^ Indolactam V,^[^ ^8^, ^9^, ^10^ ^]^ Valsartan,^[^ ^11^, ^12^, ^13^ ^]^ and Pemetrexed (Figure 1).^[^ ^14^, ^15^, ^16^ ^]^ In some cases, such as Indolactam V and Valsartan, the α‐amino group is a tertiary amine with an additional substituent attached to the nitrogen.

**Figure 1:**
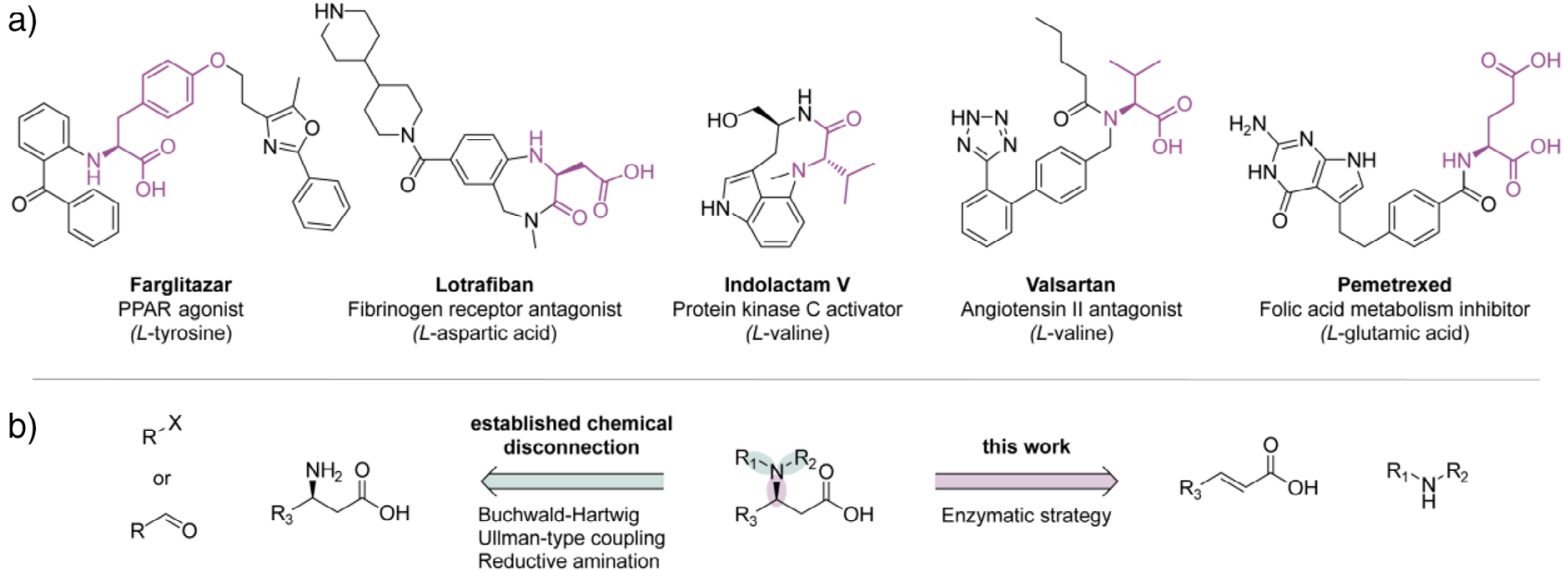
a) Biologically active molecules containing *N*‐aryl‐ and *N*‐benzyl‐substituted α‐amino acids. The α‐amino acid moieties are depicted in fuchsia. b) Established synthetic strategies and our enzymatic route for preparation of *N*‐substituted aspartic acids. X = Br, Cl, or I. R = H, aryl, alkyl, or desired functional group.

There are several well‐established chemical strategies for the asymmetric synthesis of *N*‐arylated or *N*‐alkylated α‐amino acids or esters: (I) the *N*‐arylation/alkylation of α‐amino acids catalyzed by copper (e.g., Ullmann‐type coupling)^[^ ^17^, ^18^, ^19^, ^20^ ^]^ or palladium (e.g., Buchwald–Hartwig coupling)^[^ ^21^, ^22^, ^23^ ^]^ and (II) the reductive amination of carbonyl compounds using reducing agents.^[^ ^24^, ^25^, ^26^, ^27^, ^28^ ^]^ Furthermore, intriguing routes have been reported, such as the enantioselective carbene insertion into the N─H bonds of primary and secondary amines.^[^ ^29^, ^30^, ^31^ ^]^ However, these strategies have limitations, such as the use of precious transition metals, the need for chiral starting materials or ligands, and harsh reaction conditions such as high temperatures or the requirement for strong, frequently pyrophoric bases. Biocatalysis is an attractive alternative because enzymes typically require milder, aqueous conditions, and afford the products with high regio‐ and enantioselectivity.^[^ ^32^ ^]^ There are two main enzymatic routes for the production of chiral *N*‐alkyl‐ and *N*‐aryl‐functionalized α‐amino acids:^[^ ^33^ ^]^ (I) the reductive amination of keto acids by oxidoreductases^[^ ^34^, ^35^ ^]^ such as opine dehydrogenases, *N*‐methylamino acid dehydrogenases, ketimine reductases, Δ^1^‐pyrroline‐5‐carboxylate reductases, and imine reductases (IREDs); and (II) the addition of amines to α,β‐unsaturated carboxylic acids by C–N lyases,^[^ ^36^, ^37^ ^]^ such as aspartate ammonia‐lyases, methylaspartate ammonia‐lyases (MALs), and ethylenediamine‐*N,N*‐disuccinic acid (EDDS) lyases. The latter C–N lyase enzymes are attractive biocatalysts as they provide a highly selective and atom‐economic synthesis route that does not require external organic cofactors and uses readily available prochiral starting materials.^[^ ^37^ ^]^

EDDS lyase from *Chelativorans* sp. BNC1 was previously used to prepare a variety of *N*‐substituted *L‐*aspartic acids, including *N*‐benzylated^[^ ^38^, ^39^ ^]^ and *N*‐arylated^[^ ^40^, ^41^ ^]^ *L‐*aspartic acids. Even though the enzyme naturally catalyzes the addition of ethylenediamine to two molecules of fumarate (Figure 2), it accepts many nonnative amine substrates, including amino acids,^[^ ^42^ ^]^ linear mono‐ and diamines,^[^ ^43^ ^]^ (cyclo)alkylamines,^[^ ^38^, ^44^ ^]^ arylamines,^[^ ^40^, ^41^ ^]^ benzylamines,^[^ ^38^, ^39^ ^]^ and arylhydrazines.^[^ ^40^ ^]^ This substrate promiscuity prompted us to explore EDDS lyase as a biocatalyst for the asymmetric synthesis of the precursor for Lotrafiban, a blocker of the glycoprotein IIb/IIIa receptor.^[^ ^5^ ^]^ We envisioned that asymmetric hydroamination of fumaric acid (**1**) with 2‐((methylamino)methyl)aniline (**2a**) would lead to the *N*‐arylated *L*‐aspartic acid **3a** (Figure 2). The (methylamine)methyl‐substitution could then potentially undergo a condensation reaction with the carboxylic acid, forming the seven‐membered heterocycle (**4**), and thus the precursor of Lotrafiban. Accordingly, we subjected EDDS lyase to iterative cycles of site‐saturation mutagenesis to accept **2a** as nonnative substrate for the addition to fumarate. Surprisingly, we found that the engineered enzyme variant produced a *N,N*‐disubstituted aspartic acid product, which is the result of the addition of the aliphatic secondary amine to fumarate, rather than the aromatic primary amine. Although the initially envisioned biocatalytic route toward Lotrafiban could not be pursued, we were intrigued by this unanticipated novel reactivity. We, therefore, explored the amine substrate scope of this enzyme variant and produced a range of *N,N*‐disubstituted aspartic acids as well as *N*‐substituted aspartic acids with high optical purity (up to >99% ee).

**Figure 2:**
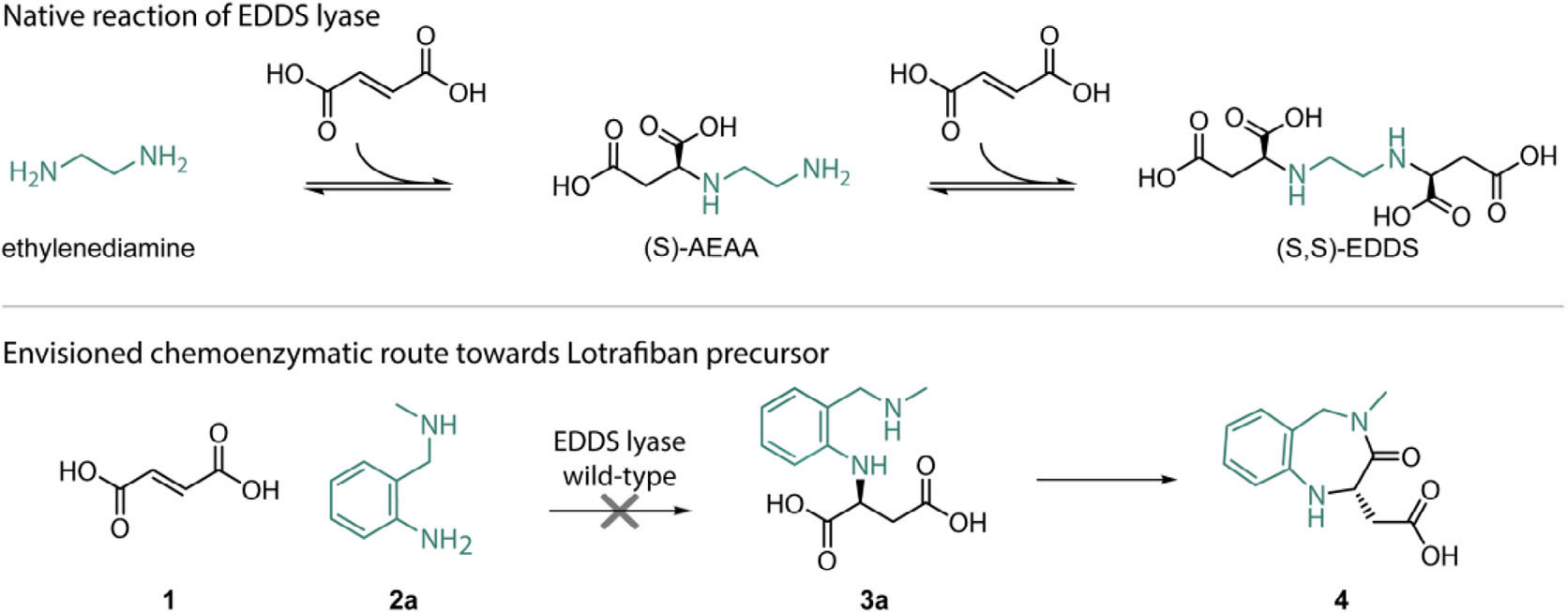
Top, EDDS lyase naturally catalyzes the stepwise addition of ethylenediamine to two molecules of fumarate. Bottom, Envisioned chemoenzymatic route toward Lotrafiban precursor **4**.

We started our investigations by testing whether wild‐type EDDS lyase can accept amine **2a** for the asymmetric addition to **1** (Figure 2). Unfortunately, amine **2a** was found to be a very poor substrate for EDDS lyase, with hardly any product formed upon prolonged incubation. Notably, in previous explorations of the substrate scope of this enzyme, we observed poor acceptance of anilines with varying substitutions at the *ortho*‐position, whereas substrates with the same substitutions at the *meta*‐ and *para*‐positions showed good conversions.^[^ ^41^ ^]^ For this reason, we hypothesized that the limited acceptance of *ortho*‐substituted anilines was not caused by electronic effects but by steric hindrance. Inspired by the previously engineered EDDS lyase MM mutant,^[^ ^38^ ^]^ which features a slightly reshaped binding pocket to accommodate bulkier substrates, we envisioned that EDDS lyase could also be engineered to accept amine **2a** as a nonnative substrate.

To engineer EDDS lyase for the proficient addition of **2a** to **1**, a structure‐inspired protein engineering strategy was applied, where we selected residues for mutagenesis based on the crystal structure of EDDS lyase in complex with its natural substrate (*S,S*)‐EDDS (Figure 3a).^[^ ^43^ ^]^ In total, we selected six residues for iterative rounds of site‐saturation mutagenesis (SSM). These residues (R112, N113, Q159, N288, D290, and Y320) are in close proximity to the substrate (4–6 Å), with some residues presumed to contribute to the positioning of the substrate or the stabilization of the *aci*‐carboxylate intermediate.^[^ ^43^ ^]^

**Figure 3:**
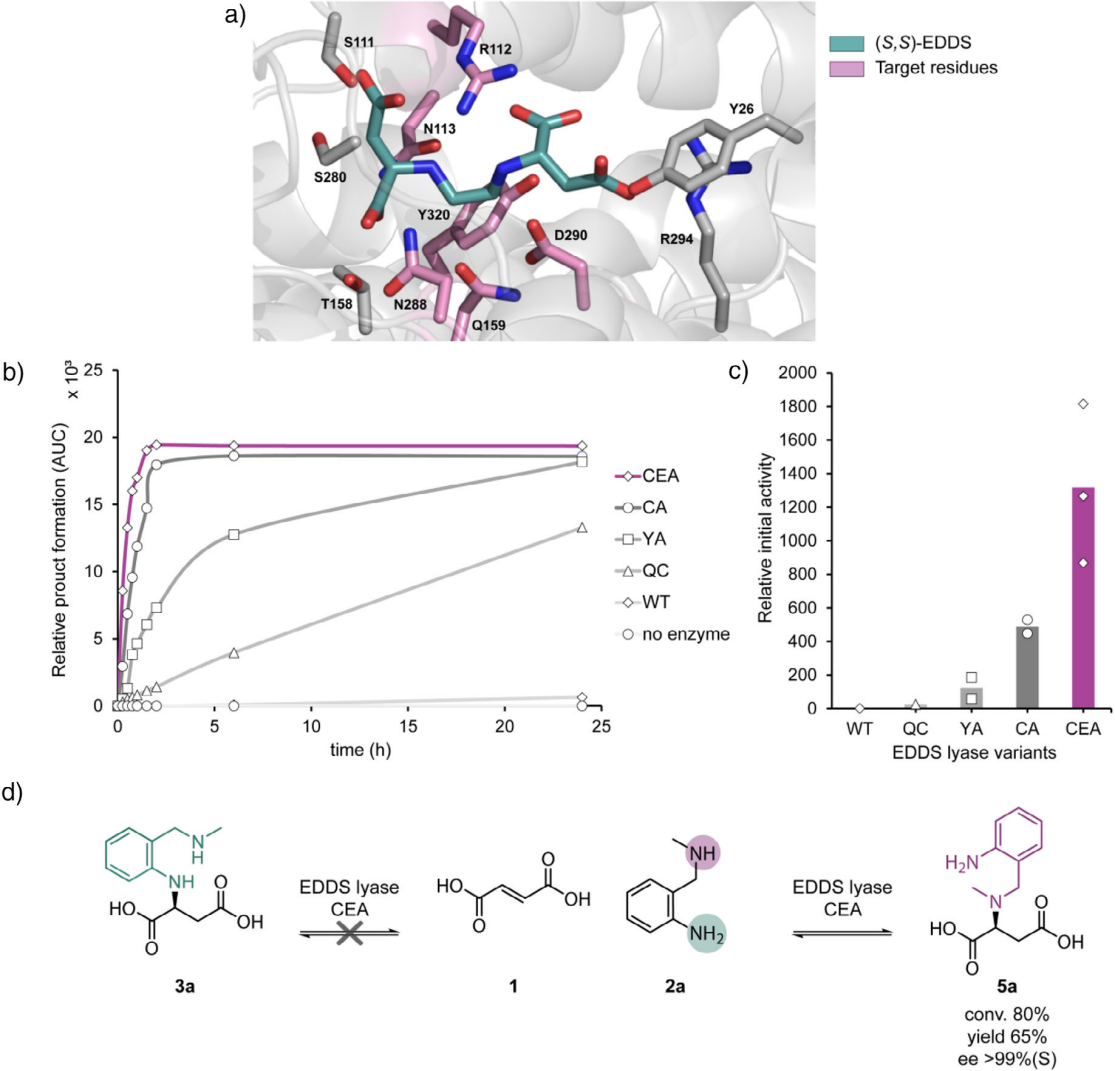
Evolutionary pathway of EDDS lyase CEA. a) Close‐up view of the active site of EDDS lyase with bound (*S*,*S*)‐EDDS (PDB: 6G3H). The substrate (green) and side chains of amino acid residues playing a role in substrate binding/stabilization are shown in stick representation. The residues targeted for mutagenesis are depicted in fuchsia. b) Reaction progress curves of different EDDS lyase variants. Product formation was monitored by HPLC using UV detection. QC and YA are single mutants Q159C and Y320A, respectively. CA is double mutant Q159C/Y320A, and CEA is triple mutant Q159C/D290E/Y320A. c) Relative activity improvement of EDDS lyase variants over WT by comparing initial activity. Each datapoint represents an independent experiment (*n* ≥ 2), and bars indicate mean values. d) Reaction scheme of the EDDS lyase‐catalyzed addition of **2a** to **1**, yielding product **5a** instead of **3a**. Conditions and reagents: fumaric acid (**1**, 500 mM), 2‐((methylamino)methyl)aniline (**2a**, 20 mM) and purified EDDS lyase CEA (0.10 mol%) in 50 mM NaHCO_3_/Na_2_CO_3_ buffer pH 9.5 at room temperature for 24 h.

The individual SSM libraries were constructed and expressed in *Escherichia coli*. We used cell‐free extracts (CFEs) to evaluate the variants in preliminary screenings (94 transformants per SSM library). To prevent the consumption of fumarate by indigenous fumarase in the CFE, the reaction mixture contained 45% glycerol, which inhibits fumarase activity without affecting the activity of EDDS lyase.^[^ ^38^ ^]^ The activity of the mutants was evaluated by monitoring substrate consumption and product formation by HPLC analysis. Screening of the SSM libraries resulted in the identification of multiple active mutants with mutations at residues Q159 and Y320. We purified seven mutants with the highest activity (Y320V, Y320P, Y320M, Y320A, Y320G, Q159C, Q159M) and tested them for the addition of **2a** to **1** (Figures 3b and S3). The Y320X variants showed higher activity compared to Q159X variants. Next, we performed a second round of mutagenesis with all seven variants as template and randomized the other respective position. Screening these Q159X/Y320X libraries revealed multiple double mutants with increased activity. Based on assays with purified enzyme variants, the mutant Q159C/Y320A (termed EDDS lyase CA) was the best‐performing mutant (Figures 3b and S3). This variant was subsequently targeted for SSM at the remaining four residue positions. Strikingly, there was only one variant, which contained the additional mutation D290E, that showed increased activity compared to the double mutant. The resulting triple mutant (termed EDDS lyase CEA) served as the template for a fourth round of SSM at the remaining three positions, but no better variant was identified.

The engineered EDDS lyase variant CEA showed an impressive activity of over three orders of magnitude higher than that of the wild‐type enzyme (Figure 3c). However, the enzymatic reaction reached an equilibrium at around 40% conversion. To enhance the conversion, we increased the concentration of starting amine **2a** to 20 mM, used 25 times excess of **1** over **2a**, and performed the reaction in NaHCO_3_/Na_2_CO_3_ buffer at pH 9.5 (Figure S4). After optimizing the reaction conditions, we performed the reaction on preparative scale with excellent conversion (80% in 24 h) and good isolated product yield (65%, 326 mg). However, to our surprise, comparing the ^1^H NMR spectrum of the enzymatic product with that of chemically prepared **3a** showed significant differences, indicating that the enzymatic product is not the desired target compound **3a** (Figure 3d). After an in‐depth analysis of the enzymatic product using 1D and 2D NMR spectroscopy (Figures S5–S9), we concluded that the hydroamination of fumarate did not occur with the aromatic primary amine of **2a** but rather with the aliphatic secondary amine. The ^1^H‐^13^C HMBC NMR spectrum showed a correlation between the protons of the ─CH_3_ in the (methylamino)methyl group with the carbon of the ─CH group in the succinic acid moiety, indicating the formation of product **5a** rather than **3a** (Figures 3d and S8). Comparison of the ^1^H NMR spectra of the enzymatic product with chemically synthesized **5a** confirmed this result (Figures S10 and S31). Hence, we have evolved a unique EDDS lyase variant that can accept a secondary amine for the hydroamination of fumarate, producing a tertiary amine, that is *N*‐(2‐aminobenzyl)‐*N*‐methylaspartic acid (**5a**).

To gain a more comprehensive insight into the biocatalytic proficiency of EDDS lyase CEA, its substrate scope was investigated by examining a panel of *N*‐methyl‐1‐phenylmethanamine derivatives (Table S2). We were pleased to find that EDDS lyase CEA accepts various *N*‐methyl‐1‐phenylmethanamine derivatives with reasonable conversion (**2b‐h**, 41–60%). Compared to substrate **2a**, a slight drop in conversion was seen for most other substrates, except for the *ortho*‐hydroxy substituted substrate (**2d**). A significant drop in conversion was observed when the linker length between the amino and aryl group was increased (**2c**, *n* = 2). To further demonstrate the synthetic applicability of EDDS lyase CEA, reactions with substrates **2b‐h** were performed at a semi‐preparative scale (Figure 4), giving the corresponding products **5b‐h** in 15–44% isolated yield. Analysis of the enzymatic products by chiral HPLC demonstrated the excellent enantioselectivity of the EDDS lyase CEA mutant, yielding all products in high optical purity (83–99% ee, Figures S54–S60).

**Figure 4:**
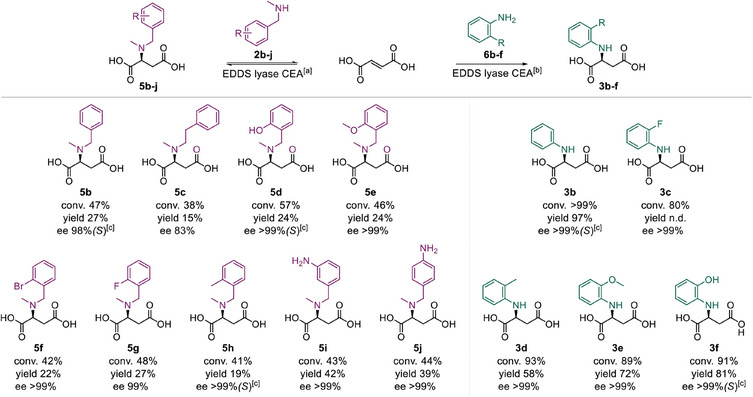
Enzymatic synthesis of *N,N*‐disubstituted aspartic acids **5b‐j** and *N*‐arylated aspartic acids **3b‐f**. a) Conditions and reagents: fumaric acid (**1**, 500 mM), amine substrate (**2b‐j**, 25 mM) and purified EDDS lyase CEA (0.05 mol%) in 50 mM NaH_2_PO_4_‐NaOH buffer pH 8.5, with 5% DMSO as co‐solvent at room temperature for 24 h. b) Conditions and reagents: fumaric acid (**1**, 100 mM), amine substrate (**6b‐f**, 25 mM) and purified EDDS lyase CEA (0.05 mol% for **6b** & **6f**, 0.10 mol% for **6c**‐**e**) in 50 mM NaH_2_PO_4_‐NaOH buffer pH 8.5, with 5% DMSO as co‐solvent at room temperature for 24 h for **6b** & **6f**, and 72 h for **6c‐e**. Conversions were determined by comparing ^1^H NMR signals of substrates and corresponding products. The turnover numbers range from 760 to 1140 for **5b‐j** and 800 to 2000 for **3b‐f**. The enantiomeric excess (ee) was established by chiral HPLC using chemically prepared racemic standards. c) The absolute configurations were determined by chiral HPLC comparing the enzymatic product with the chemically synthesized authentic standards with known *S* configuration.

The ability of EDDS lyase wild type to accept aromatic primary amines prompted us to also test several anilines (**6b‐f**) as nonnative substrates for EDDS lyase CEA (Table S2). Pleasingly, EDDS lyase CEA was able to accept these anilines for the hydroamination of fumarate, giving high conversions (80–99%). This is impressive given that wild‐type EDDS lyase shows poor acceptance of anilines **6c**, **6d**, and **6e**.^[^ ^40^ ^]^ To demonstrate the synthetic usefulness of the engineered enzyme, the reactions with substrates **6b‐f** were performed at semi‐preparative scale (Figure 4), giving the corresponding products **3b‐f** in moderate to good yields (58%–97%) and with excellent enantiopurity (>99% ee, Figures S63–S67). Hence, EDDS lyase CEA showed a broadened substrate scope, accepting various *N*‐methyl‐1‐phenylmethanamines and anilines that were not previously accepted by EDDS lyase wild type.

To further investigate the chemoselectivity of this enzyme, we also tested substrates **2i** and **2j** with the aromatic primary amine at the *meta*‐ and *para*‐position relative to the (methylamino)methyl‐substitution (Figure 5). Chiral HPLC analysis of the purified enzymatic products showed that EDDS lyase CEA has remarkable selectivity toward the aliphatic secondary amine for all three substrates **2a**, **2i**, and **2j**, forming predominantly products **5a**, **5i**, and **5j** (>98%, Figure 5) with excellent optical purity (>99% ee). This chemoselectivity, favoring secondary amines over primary amines in bifunctional substrates, is likely related to the precise positioning of these substrates in the active site of the engineered enzyme, where only the secondary amine is placed in a productive pose for the selective addition to fumarate. Apparently, an altered location of the primary amine on the aromatic ring of the substrate does not preclude optimal positioning of the secondary amine. The high activity observed with the aniline substrates further suggests that in the absence of the (methylamino)methyl‐substitution, the lack of steric constraints enables the aromatic primary amine to still adopt a productive conformation for addition to fumarate.

**Figure 5:**
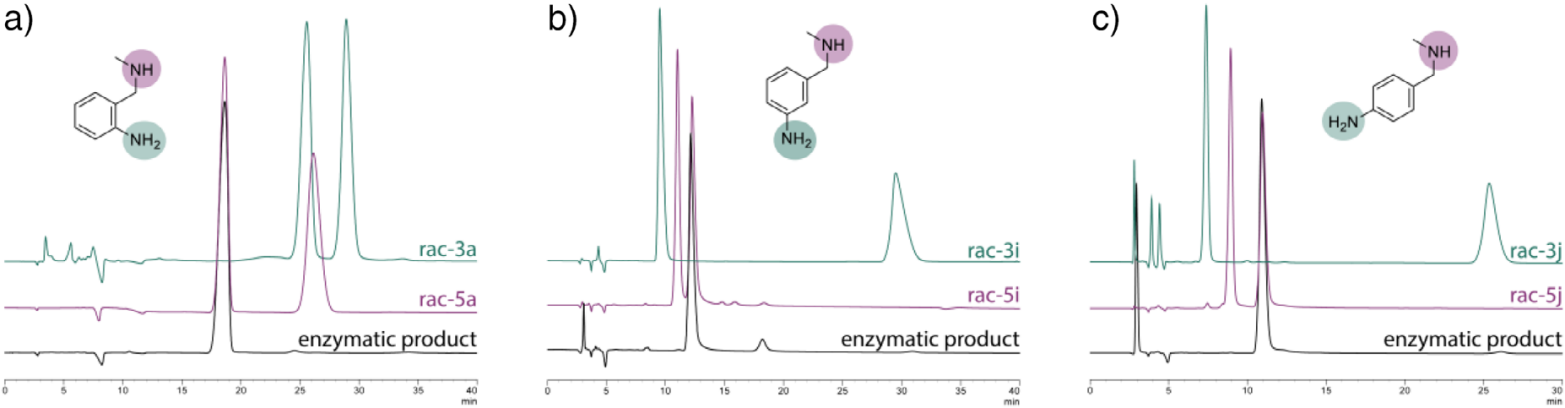
Chemoselectivity of EDDS lyase CEA toward substrates with the primary amine at the a) *ortho‐* (**2a**); b) *meta‐* (**2i**); and c) *para‐* (**2j**) position relative to the (methylamino)methyl‐substitution. Chiral HPLC chromatograms of the racemic reference for the product of hydroamination with the primary aromatic amine in green (*rac‐* **3a**,**‐3i‐j**), the racemic reference for the product of hydroamination with the secondary aliphatic amine in fuchsia (*rac‐* **5a**, ‐**5i‐j**), and the enzymatic product in black (*enz*‐**5a**, ‐**5i‐j**). HPLC conditions: Chirex 3126 (*D*)‐penicillamine column (250 x 4.6 mm, Phenomenex), phase A: 2.0 mM aqueous CuSO_4_, phase B: isopropanol, 2% B (v/v), flow rate 1.0 mL min^−1^, 50 °C, UV detection at 254 nm.

While several (engineered) enzymes, such as monoamine oxidases,^[^ ^45^ ^]^ IREDs^[^ ^46^, ^47^, ^48^, ^49^, ^50^, ^51^, ^52^ ^]^ and reductive aminases^[^ ^53^, ^54^, ^55^ ^]^ have been reported to produce optically pure tertiary amines, the toolbox of biocatalysts for tertiary amine synthesis is far from complete and a proficient C–N lyase has so far not been reported. To the best of our knowledge, only MAL from *Clostridium tetanomorphum* has been shown to accept a small secondary amine (i.e., dimethylamine) for the hydroamination of fumarate, forming *N,N*‐dimethyl aspartic acid.^[^ ^56^ ^]^ The highly active EDDS lyase variant reported here readily accepts sterically bulky secondary amines for the addition to fumarate and therefore represents a valuable expansion of the biocatalytic toolbox for the synthesis of chiral tertiary amines. Encouraged by these results, current engineering efforts focus on expanding the substrate scope toward a wider range of secondary amines, which will be relevant to generate valuable drug intermediates.

In summary, we have demonstrated that the range of transformations accessible with C–N lyases can be extended to include the asymmetric hydroamination of fumaric acid with secondary amines to obtain tertiary amines. After three rounds of directed evolution, the evolved EDDS lyase variant CEA displayed a more than 1000‐fold increased activity compared to the wild‐type enzyme. EDDS lyase CEA has a broad substrate scope, accepting various anilines for the synthesis of enantiopure *N*‐arylated *L*‐aspartic acid derivatives, as well as *N*‐methyl‐1‐phenylmethanamine and derivatives for the asymmetric synthesis of highly enantioenriched *N,N*‐disubstituted *L*‐aspartic acids. The engineered enzyme exhibits high chemoselectivity, favoring secondary amines over primary amines in bifunctional substrates. In future work, we aim to determine the crystal structure of EDDS lyase CEA in complex with *N*,*N*‐disubstituted *L*‐aspartic acids to reveal how the active site has been remodeled to accommodate the nonnative substrates.

## Supporting Information

For detailed experimental procedures and characterization of compounds, see the supporting information.

## Conflict of Interests

The authors declare no conflict of interest.

## Supporting information



Supporting Information

## Data Availability

The data that support the findings of this study are available in the Supporting Information of this article.
