# Supplementary material for: Engineered C–N Lyases for Stereoselective Synthesis of Tertiary Amines
Source: Angew Chem Int Ed Engl. 2025 Jul 4;64(34):e202507311. doi: 10.1002/anie.202507311 (PMC12363623; doi:10.1002/anie.202507311)
Supplement: Supplementary file 1 — Supporting Information [file ANIE-64-e202507311-s001.pdf]

Supporting information

Engineered C-N Lyases for Stereoselective Synthesis of Tertiary Amines

Laura Bothof,[1†] Xiaofang Gong,[1] Marrit E. Onclin,[1] Peter Fodran*,[1] and Gerrit J. Poelarends*,[1]

[1]Department of Chemical and Pharmaceutical Biology, Groningen Research Institute of Pharmacy, University of Groningen, Antonius Deusinglaan 1, 9713 AV Groningen, The Netherlands.

† Current address:

L. Bothof: Department of Bioproducts and Biosystems, School of Chemical Engineering, Aalto University, 02150 Espoo, Finland

*Address correspondence to Dr. Peter Fodran (E-mail: p.fodran@rug.nl) or Prof. Dr. Gerrit J. Poelarends (E-mail: g.j.poelarends@rug.nl).

Table of Contents

I General information 3

II Detailed Experimental Procedures 4

1. Protein expression and purification 4
2. Semi-rational engineering of EDDS lyase 6
   - 2a. Preparation of site-saturation mutagenesis libraries 6
   - 2b. Library screening 7
   - 2c. Activity assay with purified EDDS lyase variants 8
   - 2d. Preparative scale enzymatic synthesis of *N*-(2-aminobenzyl)-*N*-methyl-*L*-aspartic acid 11
3. Substrate scope exploration 12
4. Enzymatic synthesis of *ortho*-substituted *N*-benzyl-*N*-methyl-*L*-aspartic acids (***enz*-5b-j**) 13
5. Enzymatic synthesis of *ortho*-substituted phenyl-*L*-aspartic acids (***enz*-3b-f**) 17
6. Chemical synthesis of precursors (**2a**, **2d** & **2h**) 21
   - 6a. Chemical synthesis of 2-((methylamino)methyl)aniline (**2a**) 21
   - 6b. Chemical synthesis of *ortho*-substituted *N*-methyl-1-phenylmethanamine (**2d**) and (**2h**) 22
7. Chemical synthesis of racemic reference compounds 23
   - 7a. Chemical synthesis of *N*-(2-aminobenzyl)-*N*-methylaspartic acid (***rac*-5a**) 23
   - 7b. Chemical synthesis of *ortho*-substituted *N*-benzyl-*N*-methylaspartic acids (***rac*-5b-j**) 24
   - 7c. Chemical synthesis of *ortho*-substituted phenylaspartic acids (***rac*-3b-f**) 28
   - 7d. Chemical synthesis of (methylamino)methyl-substituted phenylaspartic acids (***rac*-3a**, **-3i** & **-3j**) 31
8. Chemical synthesis of enantioenriched *ortho*-substituted *N*-benzyl-*N*-methyl-*L*-aspartic acids ((***S***)**-5a**, **-5b**, **-5d** & **-5h**) 34
   - 8a. Chemical synthesis of *N*-(2-aminobenzyl)-*N*-methyl-*L*-aspartic acid ((***S***)**-5a**) 34
   - 8b. Chemical synthesis of *N*-benzyl-*N*-methyl-*L*-aspartic acid ((***S***)**-5b**, **d** & **h**) 36

III NMR spectra 39

IV Chiral HPLC data 85

V References 100

I General information

Ingredients for media and buffers were purchased from Duchefa Biochemie (Haarlem, the Netherlands) or Merck (Darmstadt, Germany). Ni sepharose 6 fast flow resin was obtained from GE Healthcare (Chicago, Illinois, US) and PD-10 Sephadex G-25 desalting columns from VWR (Radnor, Pennsylvania, US). Proteins were analyzed by sodium dodecyl sulfate polyacrylamine gel electrophoresis (SDS-PAGE) on precast NuPAGE™ 4-12% Bis-Tris protein gels from Invitrogen™ (Thermo Fisher Scientific, Waltham, Massachusetts, US). The gels were stained with Rapid Protein Stain Coomassie Blue (Westburg life sciences, Leusden, the Netherlands).

Plasmids were purified using the QIAprep Spin Miniprep Kit and PCR products were purified using the QIAquick PCR Purification Kit (Qiagen, Venlo, the Netherlands). PCR ingredients, including dNTPs, 5x Phusion® GC Reaction buffer and Phusion® DNA Polymerase, were purchased from New England Biolabs (Ipswich, Massachusetts, US). Primers used for site-directed mutagenesis were synthesized by Eurofins Genomics (Ebersberg, Germany). FastDigest *Dpn*I was purchased from Thermo Fisher Scientific (Waltham, Massachusetts, US). Mutagenesis libraries were prepared and cultured using Greiner CELLSTAR® 96 well plates and Greiner Bio-One™ Masterblock™ 96-Well MicroPlates (Greiner, Kremsmünster, Austria) sealed with gas-permeable sterile sealing film from Carl Roth (Karlsruhe, Germany). DNA sequencing was performed by Macrogen (Amsterdam, the Netherlands).

Solvents were purchased from Biosolve (Valkenswaard, the Netherlands) or Sigma-Aldrich Chemical Co (Saint Louis, Missouri, US). Chemicals were purchased from Merck (Darmstadt, Germany), Fisher Scientific (Hampton, New Hampshire, US), Sigma-Aldrich Chemical Co., ABCR (Karlsruhe, Germany), TCI chemicals (Zwijndrecht, Belgium), or BLDpharm (BLD Pharmatech GmbH, Reinbek, Germany). Amberchrom™ 50WX8 resin (hydrogen form, 100-200 mesh) was bought from Supelco (Merck, Darmstadt, Germany).

High performance liquid chromatography (HPLC) for activity screening was performed using a Shimadzu LC-10AT HPLC with a Shimadzu SPD-M10A diode array detector and chiral HPLC was performed using Shimadzu LC-20AD HPLC with a Shimadzu SPD-20A dual UV detector. NMR spectroscopy was recorded on a Brucker Avance NEO 600 MHz spectrometer equipped with a Bruker 5 mm CryoProbe Prodigy BBO, at the Stratingh Institute for Chemistry at the University of Groningen.

NMR spectra of purified products are recorded under basic (0.1 M NaOD in D_2O) or acidic (0.1 N DCl in D_2O) conditions to ensure the products are in the same protonation state, facilitating comparison between enzymatically obtained products and chemically derived reference compounds. ^{1}H NMR data are reported as follows: chemical shift (δ ppm), multiplicity (s = singlet, d = doublet, t = triplet, q = quartet, m = multiplet, dd = double doublet), coupling constant (Hz), and integration. NMR spectra were referenced to the residual protio solvent at 4.79 ppm for ^{1}H in deuterium oxide, and 7.26 ppm for ^{1}H and 77.16 ppm for ^{13}C NMR in chloroform-d. HRMS spectra were recorded in positive mode on a Thermo Scientific Q Exactive plus hybrid quadrupole-orbitrap mass spectrometer (Thermo Fisher Scientific) at the Interfaculty Mass Spectrometry Center of the University of Groningen and University Medical Center Groningen.

II Detailed Experimental Procedures

1. Protein expression and purification

Expression and purification of wild-type (WT) EDDS lyase and its variants were performed using previously described protocols, with slight modifications.[1] Amino acid sequences of EDDS lyase WT and the best performing variant (EDDS lyase CEA) can be found in Figure S1. Individual EDDS lyase variants with C-terminal His_6-tag were expressed using the pBADN expression system and purified from *E. coli* TOP10 cells. Typically, 0.2-1 L terrific broth medium, supplemented with 100 µg/mL ampicillin, was inoculated with 1.0-4.0 mL of an overnight culture of TOP10 cells containing the desired plasmid. The cells were grown at 37 °C and 210 rpm until an OD_{600} of 0.6-0.8 was reached. The cultures were cooled down, *L*-arabinose (0.05% w/v) was added to induce enzyme expression, and incubation was continued at 18 °C and 180 rpm overnight. The next day, the cells were harvested by centrifugation (5000 rpm, 30 min, 4 °C), washed with 20 mL of lysis buffer (50 mM Tris-HCl, 300 mM NaCl, 20 mM imidazole, pH 8.0) and the cell pellet stored at -20 °C until further use.

```
EDDS lyase WT     1 MNINVPDATRIGRATGAKAPEFQELYDFDAAALTLTSAVFPYDSQIHRAHVVMLTEQGIL  60
EDDS lyase CEA    1 MNINVPDATRIGRATGAKAPEFQELYDFDAAALTLTSAVFPYDSQIHRAHVVMLTEQGIL  60

EDDS lyase WT    61 TVEESATILSGLAQVDELAATDGSLRTYLPYEAALKRTIGSVAGKMHIGRSRNDLANAGK 120
EDDS lyase CEA   61 TVEESATILSGLAQVDELAATDGSLRTYLPYEAALKRTIGSVAGKMHIGRSRNDLANAGK 120

EDDS lyase WT   121 RMFLRDQLLRTIEAVIGYREAVVHKAADHLDTVMVVYTQRKEAQPITLGHYLMAISENLA 180
EDDS lyase CEA  121 RMFLRDQLLRTIEAVIGYREAVVHKAADHLDTVMVVYTCRKEAQPITLGHYLMAISENLA 180

EDDS lyase WT   181 KNLDRYRELYARINLCPLGAAATAGTGWPLNRDRTSALLGFDGLVVNSIEGVAGWDHVAE 240
EDDS lyase CEA  181 KNLDRYRELYARINLCPLGAAATAGTGWPLNRDRTSALLGFDGLVVNSIEGVAGWDHVAE 240

EDDS lyase WT   241 HAFVNAVFLSGLSRLASEIQLWSTDEYQVAELDASFAGTSSIMPQKKNPDSLERSRKAAF 300
EDDS lyase CEA  241 HAFVNAVFLSGLSRLASEIQLWSTDEYQVAELDASFAGTSSIMPQKKNPESLERSRKAAF 300

EDDS lyase WT   301 AAMGPLVGILTSLNAIEYQYSAARVELEPRSIDALIAATHAMTGVVRTLHPNKERMRQYA 360
EDDS lyase CEA  301 AAMGPLVGILTSLNAIEYQASAARVELEPRSIDALIAATHAMTGVVRTLHPNKERMRQYA 360

EDDS lyase WT   361 AENYSTMTDLTDMLVRRVGIDYREAHEIVAHVVITAIEKGIKANKIGLDLVQEAAVAQTG 420
EDDS lyase CEA  361 AENYSTMTDLTDMLVRRVGIDYREAHEIVAHVVITAIEKGIKANKIGLDLVQEAAVAQTG 420

EDDS lyase WT   421 AGINVSADDIKDALDPWQNVLRREGKGMPAPMSVKASIDDAMAELHKDRAWLANATQALA 480
EDDS lyase CEA  421 AGINVSADDIKDALDPWQNVLRREGKGMPAPMSVKASIDDAMAELHKDRAWLANATQALA 480

EDDS lyase WT   481 NAKQTLADSVQQIIQTDRKYLRHHHHHH                                 508
EDDS lyase CEA  481 NAKQTLADSVQQIIQTDRKYLRHHHHHH                                 508
```

Figure S1. Sequence alignment of the protein sequences of EDDS lyase WT from *Chelativorans* sp. BNC1 and EDDS lyase CEA. Mutated residues are highlighted in fuchsia. The sequence alignment was performed and visualized within Jalview.[2]

In a typical purification protocol, the cells were thawed and resuspended in 15 mL of lysis buffer. Cells were disrupted by sonication using 7 cycles of 40 s at 60 W output, with cooling steps in between each cycle. Unbroken cells and cell debris were removed by centrifugation (18000 rpm, 45 min, 4 °C). The supernatant was incubated with 1.0 mL of Ni-Sepharose, which had previously been equilibrated with lysis buffer (2 x 15 mL), under slow rotation for 2h – 18h at 4 °C. The Ni-Sepharose and lysate mixture was loaded on a column and non-bound proteins were removed by gravity flow. The column was washed with 2 x 15 mL of lysis buffer, followed with 2 x 15 mL of wash buffer (50 mM Tris-HCl, 300 mM NaCl, 40 mM imidazole, pH 8.0). The target protein was eluted with 2 x 2.5 mL of elution buffer (50 mM Tris-HCl, 300 mM NaCl, 300 mM imidazole, pH 8.0). Fractions were collected and analyzed by SDS-PAGE on gels containing 4-12% acrylamide. The fractions containing the target protein were combined and the buffer was exchanged with 50 mM NaH_2PO_4-NaOH buffer, pH 8.5, using a PD-10 desalting column. The protein solution was snap-frozen in liquid nitrogen and stored at -20 °C until further use. Protein concentration was determined using the measured absorbance at 280 nm, the theoretical molecular weight, and the calculated extinction coefficient of the protein.

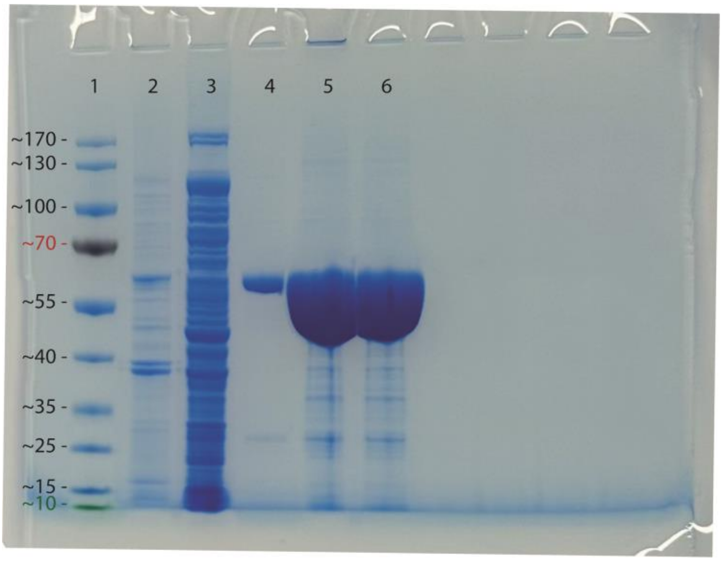

Figure S2. SDS-PAGE analysis of protein samples during purification of EDDS lyase CEA using Ni-affinity chromatography. Lane 1: PageRuler^{TM} prestained protein ladder (Thermo Scientific). Lane 2: cell pellet. Lane 3: unbound proteins in flow-through fractions. Lane 4: washing fractions. Lane 5 & 6: elution fractions. EDDS lyase CEA including His-tag has a molecular weight of approximately 56 kDa.

2. Semi-rational engineering of EDDS lyase

2a. Preparation of site-saturation mutagenesis libraries

EDDS lyase WT served as a starting template for iterative rounds of site-saturation mutagenesis. The residues at positions 112, 113, 159, 288, 290, and 320 were individually targeted each round. The mutant libraries were generated by amplifying the plasmid containing the gene encoding EDDS lyase WT or variant, using QuikChange technology. Phusion DNA Polymerase was employed in a reaction with primers containing degenerate NNK codons (Table S1). The PCR products were purified using a PCR Clean-up Kit and then subjected to *Dpn*I digestion to remove parental DNA. The DNA was subsequently transformed into chemically competent *E. coli* TOP10 cells. For each site-saturation mutagenesis library, 94 single colonies were picked from agar plates and used to inoculate LB-Amp media (LB media containing 150 µL/well ampicillin) in a 96-well plate. The two remaining wells were inoculated with colonies producing the parental variant as a reference for activity. Cells were cultured overnight at 37 °C and 220 rpm. 30 µL of each pre-culture was then used to inoculate 770 µL of LB-Amp media in a 96-well deep-well plate for library screening. Additionally, a glycerol stock of the remaining plate cultures was prepared by adding sterilized glycerol solution (50%, 80 µL) to give a final glycerol concentration of approximately 20%. The glycerol-library was sealed and stored at -80 °C.

Table S1. Primers used for mutagenesis. The altered codons are highlighted in bold. Fw: forward primer, rv: reverse primer

R112NNK	FW: 5'-CATTGGTCGTTCA**NNK**AATGATCTTGCAAATG-3'
	RV: 5'-TTTGCAAGATCATT**MNN**TGAACGACCAATGTG-3'
N113NNK	FW: 5'-CATTGGTCGTTCACGC**NNK**GATCTTGCAAATG-3'
	RV: 5'-CATTGGTCGTTCACGC**NNK**GATCTTGCAAATG-3'
Q159NNK	FW: 5'-GTCGTTTACACT**NNK**CGCAAAGAAGCCCAACC-3'
	RV: 5'-GGTTGGGCTTCTTTGCG**MNN**AGTGTAAACGAC-3'
N288NNK	FW: 5'-TGCCGCAGAAGAAG**NNK**CCGGATAGCCTGG-3'
	RV: 5'-CCAGGCTATCCGG**MNN**CTTCTTCTGC-3'
D290NNK	FW: 5'-CGCAGAAGAAGAACCCG**NNK**AGCCTGGAACGTAGTCGC-3'
	RV: 5'-GCGACTACGTTCCAGGCT**MNN**CGGGTTCTTCTTCTGCG-3'
Y320NNK	FW: 5'-CCATCGAATACCAG**NNK**TCCGCAGCGCGCGTAG-3'
	RV: 5'-CTACGCGCGCTGCGGA**MNN**CTGGTATTCGATGG-3'

2b. Library screening

For library screening, each well of a 96-well deep-well plate containing 770 µL of LB-Amp media was inoculated with 30 µL of pre-culture. The cultures were incubated at 37 °C and 220 rpm. When an OD_{600} of approximately 0.8-1 was reached, the cultures were cooled to 18 °C, induced with *L*-arabinose (final concentration 0.05%, w/v) and incubated overnight at 18 °C and 200 rpm. Cell pellets were harvested by centrifugation (3700 rpm, 30 min), frozen and stored overnight. The following day, the cells were thawed and incubated with 150 µL/well lysozyme solution (1 mg/mL, prepared with 50 mM NaH_2PO_4-NaOH buffer, pH 8.5) at 37 °C and 220 rpm for 1-1.5 hours. The obtained crude cell lysates were centrifuged (4 °C, 3700 rpm, 1 h) and the cell-free extracts (40 µL/well) were transferred into a new 96-well plate. Reactions were initiated by adding the substrate mixture (80 µL/well) to the cell-free extracts. The final reaction mixtures (120 µL/well) consisted of fumaric acid (**1**, 50 mM), 2-

((methylamino)methyl)aniline (**2a**, 10 mM), glycerol (45%, v/v), and cell-free extract (33% v/v) in 50 mM NaH_2PO_4-NaOH buffer (pH 8.5). Reactions were performed at room temperature and stopped at indicated time points by adding acetonitrile (160 µL/well). The plates were centrifuged (3700 rpm, 45 min) to remove precipitated proteins and the supernatant was used for HPLC analysis. Plasmids encoding the most active variants were isolated, sequenced and served as templates for the next round of mutagenesis.

HPLC analysis was performed using the Kinetex EVO C18 column (160 x 4.6 mm, 5 µm, Phenomenex), with an isocratic mobile phase consisting of 5% acetonitrile in water, containing 0.1 % formic acid (v/v). The flow rate was set to 1.0 mL/min, the column temperature maintained at 40 °C. Detection was performed at 254 nm.

2c. Activity assay with purified EDDS lyase variants

Comparison of EDDS lyase variants

The activity of purified EDDS lyase and its variants was compared by monitoring product formation over time (Figure S2). The reaction mixtures (2.0 mL) consisted of fumaric acid (**1**, 50 mM), 2-((methylamino)methyl)aniline (**2a**, 5 mM), glycerol (0% or 45% v/v), and purified enzyme (5 µM) in 50 mM NaH_2PO_4-NaOH buffer (pH 8.5). The reactions were incubated at room temperature. At each time point, a 150 µL sample was transferred to an Eppendorf tube, and the enzyme was inactivated by heating to 80 °C for 10 min. The samples were centrifuged (10000 rpm, 10 min) to remove precipitated proteins, and the supernatant was used for HPLC analysis. HPLC analysis was performed as described before for library screening.

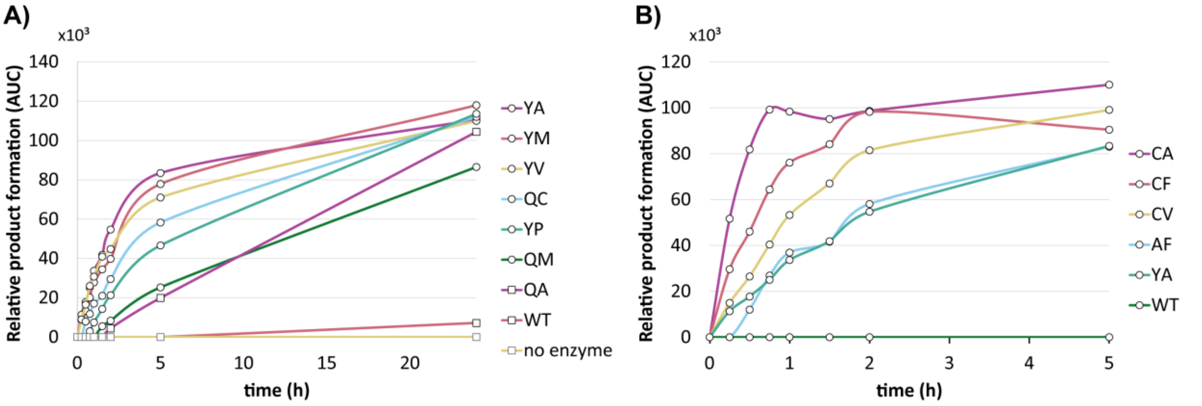

Figure S3. Activity comparison of EDDS lyase wild-type, single mutant variants, and double mutant variants. A) Product formation by EDDS lyase Y320A (YA), Y320M (YM), Y320V (YV), Q159C (QC), Y320P (YP), Q159M (QM), Q159A (QA), and wild-type (WT). B) Product formation by EDDS lyase Q159C/Y320A (CA), Q159C/Y320F (CF), Q159C/Y320V (CV), Q159A/Y320F (AF), Y320A (YA), and wild-type (WT).

Optimization of reaction conditions

Optimal reaction conditions were determined using NMR spectroscopy. Reaction mixtures (2.0 mL) were prepared, varying one parameter at a time: pH, substrate loading, or buffer system. After 24 h, a 1.0 mL sample was taken from the reaction mixture, and the enzyme was inactivated by heating to 80 °C for 10 min. Precipitated proteins were removed by centrifugation (10000 rpm, 10 min). The supernatant was evaporated under vacuum, and the resulting residue was dissolved in 0.5 mL of D_2O for ^1H NMR measurement. The conversion was estimated by comparing ^1H NMR signals of substrates and corresponding products (Figure S3a). To determine the effect of pH on the conversion after 24 h, reaction mixtures (2.0 mL) consisted of fumaric acid (**1**, 50 mM), 2-((methylamino)methyl)aniline (**2a**, 5 mM) and purified enzyme (0.10 mol%) in 50 mM NaH_2PO_4-NaOH buffer. The pH of the substrate mixture was adjusted to the desired level (pH 8.0-9.5), whereafter purified enzyme was added to initiate the reaction. To determine the influence of substrate loading on the conversion after 24 h, reaction mixtures (2.0 mL) consisted of fumaric acid (**1**, 50-500 mM), 2-((methylamino)methyl)aniline (**2a**, 10-20 mM) and purified enzyme (0.10 mol%) in 50 mM NaH_2PO_4-NaOH buffer, pH 8.5. Because the conversion of substrate **2a** is more favorable at pH 9.5, we tested whether a $NaHCO_3$/Na_2CO_3 buffer, which maintains a higher pH range than NaH_2PO_4-NaOH buffer, could be used for this reaction. To determine the influence of buffer, reaction mixtures (15 mL) consisted of **1** (500 mM), **2a** (5 mM) and purified enzyme (0.10 mol%) in 50 mM NaH_2PO_4-NaOH buffer or 50 mM $NaHCO_3$/Na_2CO_3 buffer, adjusted to pH 9.5.

The effect of pH on the reaction progress was determined by monitoring product formation over time

using HPLC analysis (Figure S3b). The reaction mixtures (2.0 mL) consisted of fumaric acid (**1**, 50 mM), 2-((methylamino)methyl)aniline (**2a**, 5 mM), and purified EDDS lyase CEA (0.10 mol%) in 50 mM NaH_2PO_4-NaOH buffer (pH 8.0-9.5). The pH of the substrate mixture was adjusted to the desired level, whereafter purified enzyme was added to initiate the reaction. The reactions were incubated at room temperature and at each time point, a 150 µL sample was transferred to an Eppendorf tube. The enzyme was inactivated by heating to 80 °C for 10 min. Precipitated proteins were removed by centrifugation (10,000 rpm, 10 min), and the supernatant was used for HPLC analysis, as described previously.

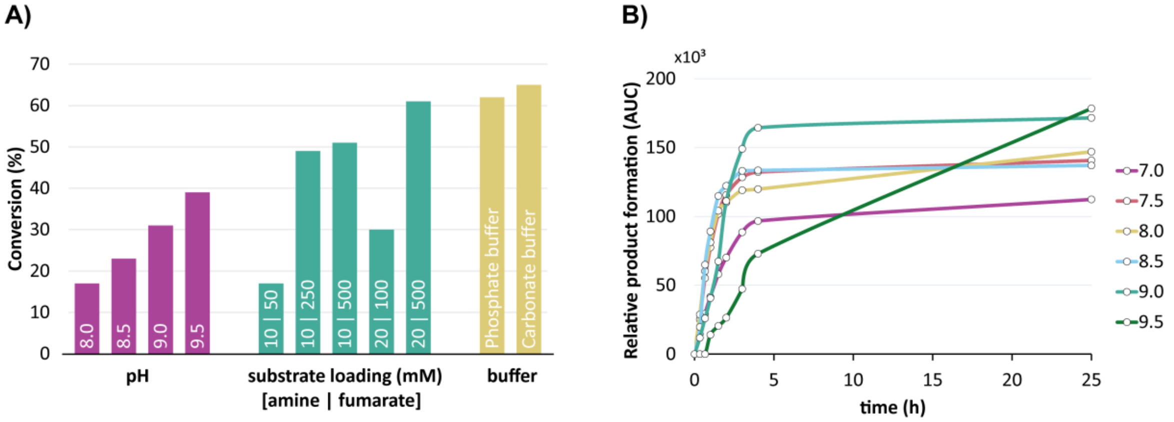

Figure S4. Optimization of reaction conditions. A) Comparison of the conversion after 24 h under various reaction conditions (pH, substrate loading, and buffer). Conversion was determined by comparing ^{1}H NMR signals of substrates and corresponding products. For different pH values, the reaction mixture consisted of fumaric acid (**1**, 50 mM), 2-((methylamino)methyl)aniline (**2a**, 5 mM) and purified EDDS lyase CEA (0.10 mol%) in 50 mM NaH_2PO_4-NaOH buffer with the pH adjusted to the desired level (pH 8.0-9.5). For different substrate loading, the reaction mixtures consisted of fumaric acid (**1**, 50-500 mM), 2-((methylamino)methyl)aniline (**2a**, 10-20 mM) and purified EDDS lyase CEA (0.10 mol%) in 50 mM NaH_2PO_4-NaOH buffer, pH 8.5. For the different buffers, the reaction mixtures consisted of **1** (500 mM), **2a** (5 mM) and purified enzyme (0.10 mol%) in 50 mM NaH_2PO_4-NaOH buffer adjusted to pH 9.5 or 50 mM $NaHCO_3$/Na_2CO_3 buffer adjusted to pH 9.5. B) Comparison of reaction progress when performed at different pH values. Product formation was monitored over time using HPLC analysis. The reaction mixtures consisted of fumaric acid (**1**, 50 mM), 2-((methylamino)methyl)aniline (**2a**, 5 mM), and purified EDDS lyase CEA (0.10 mol%) in 50 mM NaH_2PO_4-NaOH buffer (pH 8.0-9.5). Data shown are from a single experiment.

2d. Preparative scale enzymatic synthesis of *N*-(2-aminobenzyl)-*N*-methyl-*L*-aspartic acid

The reaction mixture (100 mL) consisted of fumaric acid (**1**, 500 mM) and 2-((methylamino)methyl)aniline (**2a**, 20 mM, 268 mg) in 50 mM $NaHCO_3/Na_2CO_3$ buffer, adjusted to pH 9.5. The enzymatic reaction was initiated by adding EDDS lyase CEA (0.10 mol%). The mixture was then incubated at room temperature under gentle shaking. After 24 h, the reaction mixture was heated to 80 °C for 10 min to inactivate the enzyme and a 1.0 mL sample was taken for ^{1}H NMR analysis. The conversion was determined to be 81% by comparing integrated signals of the amine substrate and corresponding product.

For the purification procedure, the precipitated enzyme was removed by centrifugation. The supernatant was washed with ethyl acetate (3x 50 mL) to remove the remaining amine substrate. The aqueous layer was acidified with 1 M HCl to pH 1.0, and the precipitated fumaric acid was filtered off. The filtrate was then loaded onto a cation-exchange column (10 mL Dowex 50W X8 resin, 100-200 mesh, H^+ form), which was pretreated with 2 M aqueous ammonia (5 column volumes), water until pH neutral, 1 M HCl (5 column volumes) and water until pH neutral. The column was washed with water to remove the remaining fumaric acid and salts, until the pH of the eluent was neutral. The product was eluted with 2 M aqueous ammonia. The ninhydrin-positive fractions were collected, concentrated under vacuum and lyophilized to provide the desired product as an ammonium salt (326 mg, 66% yield).

***N*-(2-aminobenzyl)-*N*-methyl-*L*-aspartic acid (*enz*-5a)**

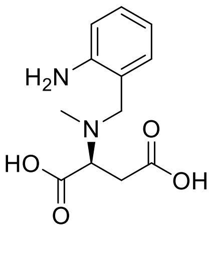

Yellowish solid, 326 mg (65% yield). **^{1}H NMR** (600 MHz, Deuterium Oxide) δ 7.22 – 7.15 (m, 2H), 6.85 – 6.75 (m, 2H), 4.05 (s, 2H), 3.87 (s, 1H), 2.73 (dd, J = 16.8, 4.9 Hz, 1H), 2.64 (dd, J = 16.6, 8.4 Hz, 1H), 2.52 (s, 3H). **^{13}C NMR** (151 MHz, D_2O) δ 178.25, 174.57, 160.33, 146.30, 132.64, 130.64, 119.17, 117.43, 66.42, 56.17, 36.65, 35.84. **HRMS** (ESI$^+$): calcd. for $C_{12}H_{17}N_2O_4$ $[M+H]^+$: 253.1183, found: 253.1180. **Chiral HPLC**: Chirex 3126 (*D*)-penicillamine column (250 x 4.6 mm, Phenomenex), mobile phase: 2.0 mM aqueous $CuSO_4$, flow rate 1.0 mL/min, 50 °C, UV detection at 254 nm. T_R(5a) = 22.6 min (>99%). The *ee* was determined to be >99% (*S*) by chiral HPLC analysis using racemic and enantiomerically enriched standards.

3. Substrate scope exploration

Table S2. Substrate scope exploration of EDDS lyase CEA

No	Substrate		Product	Conv.[c]	No	Substrate		Product	Conv.[c]
1	**2a**[a]	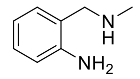	**5a**	60	10	**2j**[a]	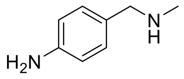	**5j**	44
2	**2b**[a]	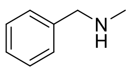	**5b**	47	11	**2k**[a]	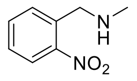	**5k**	0
3	**2c**[a]	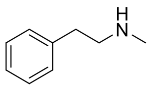	**5c**	38	12	**2l**[a]	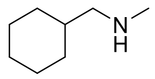	**5l**	2
4	**2d**[a]	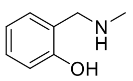	**5d**	57	13	**2m**[a]	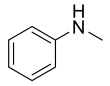	**5m**	0
5	**2e**[a]	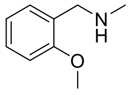	**5e**	46	14	**4b**[b]	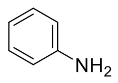	**3b**	>99
6	**2f**[a]	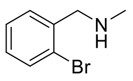	**5f**	42	15	**4c**[b]	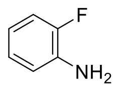	**3c**	80
7	**2g**[a]	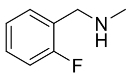	**5g**	48	16	**4d**[b]	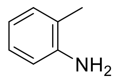	**3d**	93
8	**2h**[a]	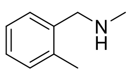	**5h**	41	17	**4e**[b]	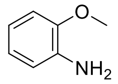	**3e**	89
9	**2i**[a]	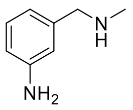	**5i**	43	18	**4f**[b]	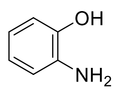	**3f**	91

[a]Conditions and reagents: fumaric acid (**1**, 500 mM), amine substrate (**2a-m**, 25 mM) and purified EDDS lyase CEA (0.05 mol%) in 50 mM NaH_2PO_4-NaOH buffer pH 8.5, with 5% DMSO as co-solvent at room temperature for 24 h. [b]Conditions and reagents: fumaric acid (**1**, 100 mM), amine substrate (**4b-f**, 25 mM) and purified EDDS lyase CEA (0.05 mol% for **4b** & **4f**, 0.10 mol% for **4c-e**) in 50 mM NaH_2PO_4-NaOH buffer pH 8.5, with 5% DMSO as co-solvent at room temperature for 24 h for **4b** & **4f**, and 72 h for **4c-e**. [c]Conversions were determined by comparing ^{1}H NMR signals of substrates and corresponding products, and are depicted in %.

4. Enzymatic synthesis of *ortho*-substituted *N*-benzyl-*N*-methyl-*L*-aspartic acids (*enz*-5b-j)

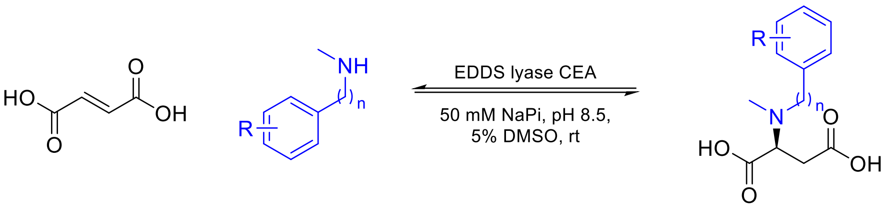

1 **2b-j** **5b-j**

	R=	*n*=
**5b**	*o*-H	1
**5c**	*o*-H	2
**5d**	*o*-OH	1
**5e**	*o*-OMe	1
**5f**	*o*-Br	1
**5g**	*o*-F	1
**5h**	*o*-Me	1
**5i**	*m*-NH_2	1
**5j**	*p*-NH_2	1

General procedure: The reaction mixture (40 mL) consisted of fumaric acid (**1**, 500 mM) and an *N*-methylphenylmethanamine substrate (**2b-j**, 25 mM) in 50 mM NaH_2PO_4-NaOH buffer (pH 8.5) with 5% DMSO as cosolvent. The pH of the reaction was adjusted to pH 8.5. The enzymatic reaction was started by the addition of EDDS lyase CEA (0.05 mol%) and incubated at room temperature. After 24 h, the enzyme was inactivated by heating to 80 °C for 10 min and the conversion was determined using ^{1}H NMR spectroscopy, by comparing the signals of the substrates and corresponding products (Table S2).

For a typical purification procedure, the precipitated enzyme was removed by centrifugation. The filtrate was basified with $NaHCO_3$, and washed with ethyl acetate (3x 20 mL) to remove the remaining amine substrate. The aqueous layer was acidified to pH 1.0 with 1 M HCl, and the precipitated fumaric acid was filtered off. The filtrate was then loaded onto a cation-exchange column (5.0 mL Dowex 50W X8 resin, 100-200 mesh, H^+ form), which was pretreated with 2 M aqueous ammonia (5 column volumes), water until eluent pH reached neutral, 1 M HCl (5 column volumes) and water until eluent pH reached neutral. The column was washed with water to remove the remaining fumaric acid and eluted with 2 M aqueous ammonia until the desired product was collected. The fractions that stained positive with ninhydrin or potassium permanganate were collected, concentrated under vacuum and lyophilized to provide the desired products as ammonium salts.

N-benzyl-*N*-methyl-*L*-aspartic acid (*enz*-5b)

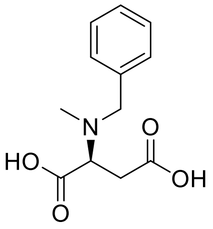

White solid, 64 mg (27% yield). **^{1}H NMR** (600 MHz, 0.1 M NaOD in Deuterium Oxide) δ 7.44 – 7.37 (m, 4H), 7.37 – 7.32 (m, 1H), 3.75 (d, *J* = 12.7 Hz, 1H), 3.54 (dd, *J* = 8.0, 6.5 Hz, 1H), 3.50 (d, *J* = 12.6 Hz, 1H), 2.66 (dd, *J* = 15.3, 8.2 Hz, 1H), 2.49 (dd, *J* = 15.2, 6.3 Hz, 1H), 2.17 (s, 3H). **^{13}C NMR** (151 MHz, 0.1 M NaOD in

D_2O) δ 180.36, 178.83, 138.41, 130.06, 128.45, 127.44, 67.22, 58.08, 38.71, 38.26. **HRMS** (ESI^+): calcd. for $C_{12}H_{16}NO_4$ $[M+H]^+$: 238.1074, found: 238.1070. **Chiral HPLC**: Nucleosil Chiral-1 column (5 µm, 250 x 4 mm, Macherey-Nagel), mobile phase: 0.5 mM aqueous $CuSO_4$, flow rate 1.0 mL/min, 60 °C, UV detection at 254 nm. T_R = 6.1 min. The *ee* was determined to be 98% (*S*) by chiral HPLC analysis using racemic and enantiomerically enriched standards.

***N*-methyl-*N*-phenethyl-*L*-aspartic acid (*enz*-5c)**

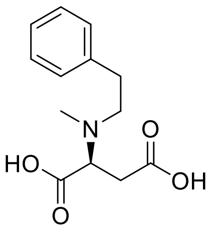

White solid, 38 mg (15% yield). **^{1}H NMR** (600 MHz, 0.1 N DCl in Deuterium Oxide) δ 7.44 (t, *J* = 7.4 Hz, 2H), 7.37 (d, *J* = 7.7 Hz, 3H), 4.39 (t, *J* = 6.2 Hz, 1H), 3.61 – 3.38 (m, 2H), 3.22 – 3.07 (m, 4H), 2.99 (s, 3H). **^{13}C NMR*** (151 MHz, 0.1 N DCl in D_2O) δ 173.33, 135.89, 129.14, 128.89, 127.49, 30.85, 30.46. **HRMS** (ESI^+): calcd. for $C_{13}H_{18}NO_4$ $[M+H]^+$: 252.1230 found: 252.1225. **Chiral HPLC**: Nucleosil Chiral-1 column (5 µm, 250 x 4 mm, Macherey-Nagel), mobile phase: 0.5 mM aqueous $CuSO_4$, flow rate 1.0 mL/min, 60 °C, UV detection at 254 nm. T_R = 9.6 min. The ee was determined to be at least 83% by chiral HPLC analysis using a racemic standard. *In the recorded ^{13}C NMR, some expected carbon peaks are missing. This observation is consistent with the spectrum of the reference compound ***rac*-5c**. Despite the absence of these specific carbon signals, all other data, including HPLC, HRMS and ^{1}H NMR, are consistent with the structure of the expected product.

***N*-(2-hydroxybenzyl)-*N*-methyl-*L*-aspartic acid (*enz*-5d)**

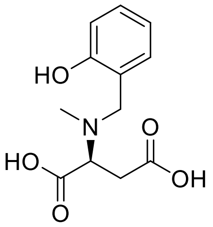

Yellowish solid, 60 mg (24% yield). **^{1}H NMR** (600 MHz, 0.1 M NaOD in Deuterium Oxide) δ 7.23 (d, *J* = 7.3 Hz, 1H), 7.10 (t, *J* = 7.5 Hz, 1H), 6.65 (d, *J* = 8.1 Hz, 1H), 6.61 (t, *J* = 7.3 Hz, 1H), 3.68 – 3.57 (m, 3H), 2.69 (dd, *J* = 15.3, 8.2 Hz, 1H), 2.55 (dd, *J* = 15.0, 5.6 Hz, 1H), 2.19 (s, 3H). **^{13}C NMR** (151 MHz, 0.1 M NaOD in D_2O) δ 180.58, 179.21, 164.85, 131.17, 128.63, 126.44, 118.98, 114.33, 67.60, 53.13, 38.26, 37.54. **HRMS** (ESI^+): calcd. for $C_{12}H_{16}NO_5$ $[M+H]^+$: 254.1023 found: 254.1020. **Chiral HPLC**: Chirex 3126 (*D*)-penicillamine column (250 x 4.6 mm, Phenomenex), phase A: 2.0 mM aqueous $CuSO_4$, phase B: isopropanol, 10% B (v/v), flow rate 1.0 mL/min, 50 °C, UV detection at 254 nm. T_R = 13.7 min. The *ee* was determined to be >99% (*S*) by chiral HPLC analysis using racemic and enantiomerically enriched standards.

N-(2-methoxybenzyl)-N-methyl-L-aspartic acid (enz-5e)

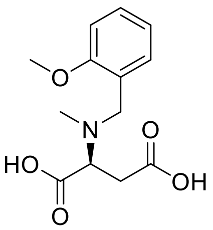

Yellowish solid, 64 mg (24% yield). **^{1}H NMR** (600 MHz, 0.1 M NaOD in Deuterium Oxide) δ 7.40 – 7.29 (m, 2H), 7.05 (d, *J* = 8.2 Hz, 1H), 7.01 (t, *J* = 7.4 Hz, 1H), 3.85 (s, 3H), 3.76 (d, *J* = 12.6 Hz, 1H), 3.54 (dd, *J* = 8.8, 5.5 Hz, 1H), 3.46 (d, *J* = 12.6 Hz, 1H), 2.73 (dd, *J* = 15.5, 9.0 Hz, 1H), 2.52 (dd, *J* = 15.5, 5.3 Hz, 1H), 2.18 (s, 3H). **^{13}C NMR** (151 MHz, 0.1 M NaOD in D_2O) δ 180.42, 178.66, 157.86, 132.02, 129.13, 126.34, 120.54, 111.52, 67.54, 55.46, 51.95, 38.66, 38.57. **HRMS** (ESI^+): calcd. for $C_{13}H_{18}NO_5$ $[M+H]^+$: 268.1179, found: 268.1179. **Chiral HPLC**: Chirex 3126 (*D*)-penicillamine column (250 x 4.6 mm, Phenomenex), phase A: 2.0 mM aqueous $CuSO_4$, phase B: isopropanol, 5% B (v/v). Flow rate of 1.0 mL/min, 50 °C, UV detection at 254 nm. T_R = 15.8 min. The *ee* was determined to be >99% by chiral HPLC analysis using a racemic standard.

N-(2-bromobenzyl)-N-methyl-L-aspartic acid (enz-5f)

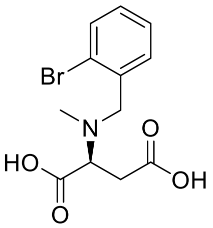

Yellowish solid, 69 mg (22% yield). **^{1}H NMR** (600 MHz, 0.1 M NaOD in Deuterium Oxide) δ 7.60 (d, *J* = 8.0 Hz, 1H), 7.41 (d, *J* = 7.5 Hz, 1H), 7.33 (t, *J* = 7.4 Hz, 1H), 7.19 (t, *J* = 7.7 Hz, 1H), 3.77 (d, *J* = 13.1 Hz, 1H), 3.68 (d, *J* = 13.1 Hz, 1H), 3.60 (dd, *J* = 8.6, 6.1 Hz, 1H), 2.68 (dd, *J* = 15.5, 8.8 Hz, 1H), 2.51 (dd, *J* = 15.4, 5.8 Hz, 1H), 2.16 (s, 3H). **^{13}C NMR** (151 MHz, 0.1 M NaOD in D_2O) δ 180.35, 178.49, 137.39, 132.86, 132.29, 129.23, 127.50, 124.91, 67.98, 57.96, 38.77, 38.14. **HRMS** (ESI^+): calcd. for $C_{12}H_{15}NO_4Br$ $[M+H]^+$: 316.0179, found: 316.0180. **Chiral HPLC**: Nucleosil Chiral-1 column (5 µm, 250 x 4 mm, Macherey-Nagel), mobile phase: 0.5 mM aqueous $CuSO_4$, flow rate 1.0 mL/min, 60 °C, UV detection at 254 nm. T_R = 6.1 min. The *ee* was determined to be >99% by chiral HPLC analysis using a racemic standard.

N-(2-fluorobenzyl)-N-methyl-L-aspartic acid (enz-5g)

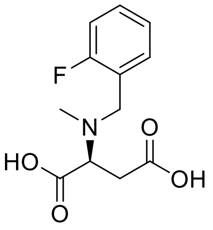

Yellowish solid, 68 mg (27% yield). **^{1}H NMR** (600 MHz, 0.1 M NaOD in Deuterium Oxide) δ 7.37 – 7.33 (m, 1H), 7.33 – 7.28 (m, 1H), 7.14 (t, *J* = 7.5 Hz, 1H), 7.11 – 7.06 (m, 1H), 3.74 (d, *J* = 12.9 Hz, 1H), 3.53 – 3.46 (m, 2H), 2.65 (dd, *J* = 15.4, 8.8 Hz, 1H), 2.44 (dd, *J* = 15.4, 5.7 Hz, 1H), 2.15 (s, 3H). **^{13}C NMR*** (151 MHz, 0.1 M NaOD in D_2O) δ 180.23, 178.51, 160.56, 132.58 – 132.53 (m), 129.58 (d, *J* = 8.7 Hz), 124.10 (d, *J* = 2.6 Hz, 115.32 (d, *J* = 22.0 Hz), 67.51, 50.93, 38.68, 38.37. **HRMS** (ESI^+): calcd. for $C_{12}H_{15}NO_4F$ $[M+H]^+$: 256.0980, found: 256.0977. **Chiral HPLC**: Nucleosil Chiral-1 column (5 µm,

250 x 4 mm, Macherey-Nagel), mobile phase: 0.5 mM aqueous $CuSO_4$, flow rate 1.0 mL/min, 60 °C, UV detection at 254 nm. T_R = 5.6 min. The *ee* was determined to be 99% by chiral HPLC analysis using a racemic standard. *In the recorded ^{13}C NMR, one expected carbon peak is absent. This might be attributed to the spectrum's resolution or quality. However, all other analytical data including HPLC, HRMS and ^{1}H NMR are consistent with the structure of the expected product.

***N*-methyl-*N*-(2-methylbenzyl)-*L*-aspartic acid (*enz*-5h)**

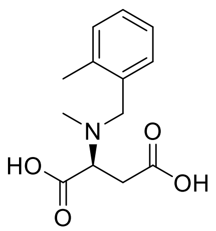

Yellowish solid, 47 mg (19% yield). **^{1}H NMR** (600 MHz, 0.1 M NaOD in Deuterium Oxide) δ 7.33 (d, *J* = 7.1 Hz, 1H), 7.29 – 7.21 (m, 3H), 3.70 (d, *J* = 12.9 Hz, 1H), 3.64 – 3.57 (m, 2H), 2.69 (dd, *J* = 15.2, 8.2 Hz, 1H), 2.52 (dd, *J* = 15.2, 6.4 Hz, 1H), 2.36 (s, 3H), 2.18 (s, 3H). **^{13}C NMR** (151 MHz, 0.1 M NaOD in D_2O) δ 180.43, 178.73, 138.06, 136.60, 130.93, 130.39, 127.55, 125.73, 68.06, 55.69, 38.63, 37.97, 18.64. **HRMS** (ESI$^+$): calcd. for $C_{13}H_{18}NO_4$ [M+H]$^+$: 252.1230, found: 252.1220. **Chiral HPLC**: Nucleosil Chiral-1 column (5 μm, 250 x 4 mm, Macherey-Nagel), mobile phase: 0.5 mM aqueous $CuSO_4$, flow rate 1.0 mL/min, 60 °C, UV detection at 254 nm. T_R = 6.4 min. The *ee* was determined to be >99% by chiral HPLC analysis using racemic and enantiomerically enriched standards.

***N*-(3-aminobenzyl)-*N*-methyl-*L*-aspartic acid (*enz*-5i)**

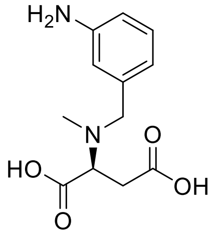

Yellowish solid, 107 mg (42% yield). **^{1}H NMR** (600 MHz, 0.1 M NaOD in Deuterium Oxide) δ 7.20 (t, *J* = 7.6 Hz, 1H), 6.84 (d, *J* = 7.0 Hz, 2H), 6.76 (d, *J* = 8.6 Hz, 1H), 3.67 (d, *J* = 12.7 Hz, 1H), 3.54 (t, *J* = 7.3 Hz, 1H), 3.41 (d, *J* = 12.7 Hz, 1H), 2.64 (dd, *J* = 15.2, 8.0 Hz, 1H), 2.48 (dd, *J* = 15.2, 6.6 Hz, 1H), 2.17 (s, 3H). **^{13}C NMR** (151 MHz, 0.1 M NaOD in D_2O) δ 180.34, 178.83, 146.12, 139.78, 129.28, 121.04, 117.81, 115.27, 67.18, 58.01, 38.73, 38.32. **HRMS** (ESI$^+$): calcd. for $C_{12}H_{17}N_2O_4$ [M+H]$^+$: 253.1183, found: 253.1177. **Chiral HPLC**: Chirex 3126 (*D*)-penicillamine column (250 x 4.6 mm, Phenomenex), phase A: 2.0 mM aqueous $CuSO_4$, phase B: isopropanol, 10% B (v/v), flow rate 1.0 mL/min, 50 °C, UV detection at 254 nm. T_R(5i) = 12.1 min (>98%). The *ee* was determined to be >99% (*S*) by chiral HPLC analysis using a racemic standard.

***N*-(4-aminobenzyl)-*N*-methyl-*L*-aspartic acid (*enz*-5j)**

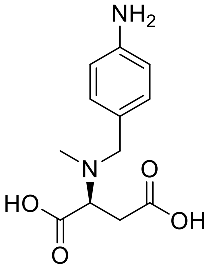

Yellowish solid, 108 mg (92 w% **5j**, 39% yield). **^{1}H NMR** (600 MHz, 0.1 M NaOD in Deuterium Oxide) δ 7.20 (d, *J* = 7.7 Hz, 2H), 6.83 (d, *J* = 7.9 Hz, 2H), 3.64 (d, *J* = 12.6 Hz, 1H), 3.53 (t, *J* = 7.1 Hz, 1H), 3.40 (d, *J* = 12.6 Hz, 1H), 2.66 (dd, *J* = 15.3, 8.4 Hz, 1H), 2.47 (dd, *J* = 15.3, 6.0 Hz, 1H), 2.15 (s, 3H). **^{13}C NMR** (151 MHz, 0.1 M NaOD in D_2O) δ 180.37, 178.83, 145.31, 131.17, 131.17, 129.09, 116.20, 67.05, 57.53, 38.72, 38.01. **HRMS** (ESI$^+$): calcd. for $C_{12}H_{17}N_2O_4$ [M+H]$^+$: 253.1183, found: 253.1183. **Chiral HPLC**: Chirex 3126 (*D*)-penicillamine column (250 x 4.6 mm, Phenomenex), phase A: 2.0 mM aqueous $CuSO_4$, phase B: isopropanol, 10% B (v/v), flow rate 1.0 mL/min, 50 °C, UV detection at 254 nm. T_R(5j) = 10.9 min. The *ee* was determined to be >99% (*S*) by chiral HPLC analysis using a racemic standard.

5. Enzymatic synthesis of *ortho*-substituted phenyl-*L*-aspartic acids (*enz*-3b-f)

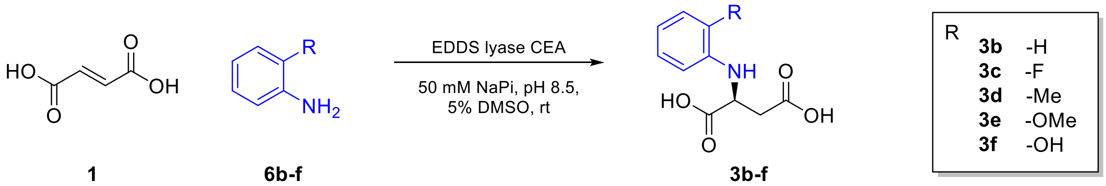

General procedure: The reaction mixture (20 mL) consisted of fumaric acid (**1**, 100 mM) and an *ortho*-substituted aniline (**6b-f**, 25 mM) in 50 mM NaH_2PO_4-NaOH buffer (pH 8.5) with 5% DMSO as cosolvent. The pH of the reaction was adjusted to pH 8.5. The enzymatic reaction was started by the addition of EDDS lyase CEA (0.05 mol% for **6b** & **6f**, 0.10 mol% for **6c-e**) and incubated at room temperature (24 h for **6b** & **6f**, and 72 h for **6c-e**). After the reaction was completed, the enzyme was inactivated by heating to 80 °C for 10 min. The conversion was determined using ^{1}H NMR spectroscopy, by comparing the signals of the substrates and corresponding products (see Table S2). For substrates **6b** and **6f**, additional 10 mL reactions were performed under the same conditions using EDDS lyase WT.[3]

The amino acid products were purified by cation-exchange chromatography. For a typical purification procedure, the precipitated enzyme was removed by centrifugation. The filtrate was basified with $NaHCO_3$, and washed with ethyl acetate (3x 20 mL) to remove the remaining amine substrate. The aqueous layer was acidified to pH 1.0 (1 M HCl) and subjected to a cation-exchange column, as described previously for the *N*-benzyl-*N*-methyl-*L*-aspartic acids (***enz*-5a-j**). The ninhydrin-positive

fractions were collected, concentrated under vacuum, and lyophilized to provide the desired products as ammonium salts. However, enzymatic product **3c** showed no affinity to the cation-exchange resin. Instead, the aqueous layer was evaporated to dryness and triturated with methanol. The methanolic solutions were combined and the volatiles were removed under reduced pressure. The resulting product, still containing fumaric acid, was used for analysis.

phenyl-*L*-aspartic acid (*enz*-3b obtained with EDDS lyase CEA)

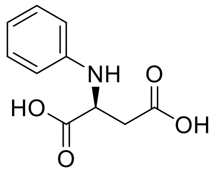

Yellowish solid, 101 mg (97% yield). **^{1}H NMR** (600 MHz, 0.1 M NaOD in Deuterium Oxide) δ 7.26 (t, *J* = 7.6 Hz, 2H), 6.81 (t, *J* = 7.2 Hz, 1H), 6.75 (d, *J* = 7.9 Hz, 2H), 4.15 (dd, *J* = 10.1, 3.2 Hz, 1H), 2.73 (dd, *J* = 14.8, 3.4 Hz, 1H), 2.46 (dd, *J* = 14.7, 10.5 Hz, 1H). **^{13}C NMR** (151 MHz, 0.1 M NaOD in D_2O) δ 181.64, 179.48, 147.93, 129.47, 118.31, 114.13, 57.73, 41.26. **HRMS** (ESI$^+$): calcd. for $C_{10}H_{12}NO_4$ [M+H]$^+$: 210.0761, found: 210.0759. **Chiral HPLC**: Nucleosil Chiral-1 column (5 µm, 250 x 4 mm, Macherey-Nagel), mobile phase: 0.5 mM aqueous $CuSO_4$, flow rate 1.0 mL/min, 60 °C, UV detection at 254 nm. T_R(*S*) = 6.2 min. The *ee* was determined to be >99% by chiral HPLC analysis using racemic and enantiomerically enriched standards and based on previously reported data.[3]

phenyl-*L*-aspartic acid (*enz*-3b obtained with WT EDDS lyase)

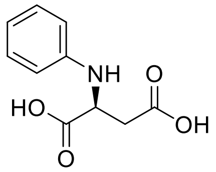

Yellowish solid, 39 mg (75% yield). **^{1}H NMR** (600 MHz, 0.1 M NaOD in Deuterium Oxide) δ 7.26 (t, *J* = 7.8 Hz, 2H), 6.81 (t, *J* = 7.3 Hz, 1H), 6.75 (d, *J* = 7.7 Hz, 2H), 4.15 (dd, *J* = 10.1, 2.8 Hz, 1H), 2.72 (dd, *J* = 14.7, 3.2 Hz, 1H), 2.45 (dd, *J* = 14.7, 10.4 Hz, 1H). **^{13}C NMR** (151 MHz, 0.1 M NaOD in D_2O) δ 181.63, 179.48, 147.93, 129.47, 118.31, 114.13, 57.73, 41.25. **HRMS** (ESI$^+$): calcd. for $C_{10}H_{12}NO_4$ [M+H]$^+$: 210.0761, found: 210.0758. **Chiral HPLC**: Nucleosil Chiral-1 column (5 µm, 250 x 4 mm, Macherey-Nagel), mobile phase: 0.5 mM aqueous $CuSO_4$, flow rate 1.0 mL/min, 60 °C, UV detection at 254 nm. T_R(*R*) = 4.7 min T_R(*S*) = 6.4 min. The *ee* was determined to be 89% (*S*) by chiral HPLC analysis using racemic standard and based on previously reported data.[3]

(2-fluorophenyl)-*L*-aspartic acid (*enz*-3c)

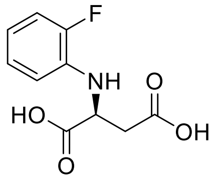

White solid, mixture with **1**, 54 mg (51 w% **3c**). **^{1}H NMR** (600 MHz, 0.1 M NaOD in Deuterium Oxide) δ 7.12 – 7.01 (m, 2H), 6.81 – 6.69 (m, 2H), 4.15 (dd, *J* = 10.1, 3.7 Hz, 1H), 2.74 (dd, *J* = 14.9, 3.8 Hz, 1H), 2.50 (dd, *J* = 14.9, 10.2 Hz, 1H). **^{13}C**

NMR (151 MHz, 0.1 M NaOD in D_2O) δ 181.09, 179.40, 151.81 (d, *J* = 237.7 Hz), 135.92 (d, *J* = 12.2 Hz), 124.86 (d, *J* = 3.3 Hz), δ 118.12 (d, *J* = 7.0 Hz), 114.77 (d, *J* = 18.7 Hz), 113.86 – 113.83 (m), 57.31, 41.08. **HRMS** (ESI^+): calcd. for $C_{10}H_{11}NO_4F$ $[M+H]^+$: 228.0667, found: 228.0663. **Chiral HPLC**: Nucleosil Chiral-1 column (5 µm, 250 x 4 mm, Macherey-Nagel), mobile phase: 0.5 mM aqueous $CuSO_4$, flow rate 1.0 mL/min, 60 °C, UV detection at 254 nm. T_R(*S*) = 5.2 min. The *ee* was determined to be >99% by chiral HPLC analysis using a racemic standard.

***o*-tolyl-*L*-aspartic acid (*enz*-3d)**

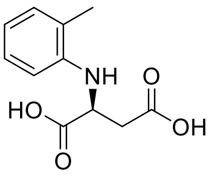

Light brown solid, 65 mg (58% yield). **^{1}H NMR** (600 MHz, 0.1 M NaOD in Deuterium Oxide) δ 7.24 – 7.11 (m, 2H), 6.76 (t, *J* = 7.3 Hz, 1H), 6.63 (d, *J* = 7.9 Hz, 1H), 4.15 (dd, *J* = 10.0, 3.4 Hz, 1H), 2.76 (dd, *J* = 14.5, 3.6 Hz, 1H), 2.53 (dd, *J* = 14.4, 10.3 Hz, 1H), 2.21 (s, 3H). **^{13}C NMR** (151 MHz, 0.1 M NaOD in D_2O) δ 181.53, 179.56, 145.76, 130.23, 127.21, 123.85, 118.01, 111.23, 57.69, 41.19, 16.64. **HRMS** (ESI^+): calcd. for $C_{11}H_{14}NO_4$ $[M+H]^+$: 224.0917, found: 224.0917. **Chiral HPLC**: Nucleosil Chiral-1 column (5 µm, 250 x 4 mm, Macherey-Nagel), mobile phase: 0.5 mM aqueous $CuSO_4$, flow rate 0.8 mL/min, 60 °C, UV detection at 254 nm. T_R(*S*) = 7.3 min. The *ee* was determined to be >99% by chiral HPLC analysis using a racemic standard.

(2-methoxyphenyl)-*L*-aspartic acid (*enz*-3e)

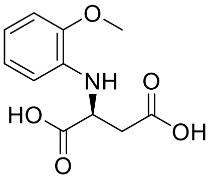

Yellowish solid, 86 mg (72% yield). **^{1}H NMR** (600 MHz, 0.1 M NaOD in Deuterium Oxide) δ 6.98 (d, *J* = 7.9 Hz, 1H), 6.94 (t, *J* = 7.6 Hz, 1H), 6.81 (t, *J* = 7.6 Hz, 1H), 6.68 (d, *J* = 7.8 Hz, 1H), 4.21 – 4.09 (m, 1H), 3.88 (s, 3H), 2.74 (dd, *J* = 14.8, 3.3 Hz, 1H), 2.53 – 2.47 (m, 1H). **^{13}C NMR** (151 MHz, 0.1 M NaOD in D_2O) δ 181.43, 179.49, 147.41, 137.59, 121.71, 118.13, 111.82, 111.24, 57.48, 56.04, 41.32. **HRMS** (ESI^+): calcd. for $C_{11}H_{14}NO_5$ $[M+H]^+$: 240.0866, found: 240.0867. **Chiral HPLC**: Nucleosil Chiral-1 column (5 µm, 250 x 4 mm, Macherey-Nagel), mobile phase: 0.5 mM aqueous $CuSO_4$, flow rate 0.8 mL/min, 60 °C, UV detection at 254 nm. T_R(*S*) = 7.1 min. The *ee* was determined to be >99% by chiral HPLC analysis using a racemic standard.

(2-hydroxyphenyl)-*L*-aspartic acid (*enz*-3f obtained with EDDS lyase CEA)

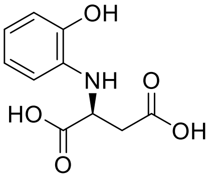

Dark brown solid, 100 mg (81 w% **3f**, 72% yield). **^{1}H NMR*** **(**600 MHz, 0.1 M NaOD in Deuterium Oxide) δ 6.62 (t, *J* = 7.4 Hz, 1H), 6.58 – 6.46 (m, 3H), 4.10 (dd, *J* = 9.5, 4.3 Hz, 1H), 2.68 (dd, *J* = 14.5, 4.3 Hz, 1H), 2.50 (dd, *J* = 14.5, 9.7 Hz, 1H). **^{13}C NMR*** (151 MHz, 0.1 M NaOD in D_2O) δ 182.24, 179.91, 154.98, 139.55, 119.02, 116.22, 114.55, 111.29, 58.05, 41.78. **HRMS** (ESI^+): calcd. for $C_{10}H_{12}NO_5$ $[M+H]^+$: 226.0710, found: 226.0712. **Chiral HPLC**: Chirex 3126 (*D*)-penicillamine column (250 x 4.6 mm, Phenomenex), phase A: 2.0 mM aqueous $CuSO_4$, phase B: isopropanol, 2% B (v/v), flow rate 1.0 mL/min, 50 °C, UV detection at 254 nm. T_R = 11.5 min. The *ee* was determined to be >99% (*S*) by chiral HPLC analysis using a racemic and enantiomerically enriched standards, and based on previously reported data.[4] *Both ^{1}H and ^{13}C NMR spectra (Figure S24) contain traces of aspartic acid, as confirmed by spiking the sample with *L*-aspartic acid (Figure S25). This observation is consistent with the spectrum of the reference compound ***rac*-3f** (Figure S44), suggesting that the formation of aspartic acid may result from the properties and/or instability of the compound.

(2-hydroxyphenyl)-*L*-aspartic acid (*enz*-3f obtained with WT EDDS lyase)

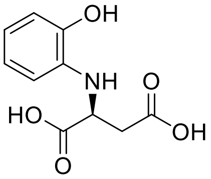

Dark brown solid, 46 mg (87 w% **3f**, 71% yield). **^{1}H NMR*** (600 MHz, 0.1 M NaOD in Deuterium Oxide) δ 6.72 – 6.64 (m, 3H), 6.64 – 6.60 (m, 1H), 4.11 (dd, *J* = 9.9, 4.0 Hz, 1H), 2.73 – 2.70 (m, 1H), 2.51 (dd, *J* = 14.6, 10.0 Hz, 1H). **^{13}C NMR*** (151 MHz, 0.1 M NaOD in D_2O) δ 181.94, 179.79, 150.54, 138.40, 119.00, 117.35, 115.78, 112.29, 58.04, 41.59. **HRMS** (ESI^+): calcd. for $C_{10}H_{12}NO_5$ $[M+H]^+$: 226.0710, found 226.0713. **Chiral HPLC**: Chirex 3126 (*D*)-penicillamine column (250 x 4.6 mm, Phenomenex), phase A: 2.0 mM aqueous $CuSO_4$, phase B: isopropanol, 2% B (v/v), flow rate 1.0 mL/min, 50 °C, UV detection at 254 nm. T_R(*S*) = 11.5 min and T_R(*R*) = 12.1 min. The *ee* was determined to be 87% (*S*) by chiral HPLC analysis using racemic standard and based on previously reported data.[4] *Both ^{1}H and ^{13}C NMR spectra (Figure S26) contain traces of aspartic acid, as confirmed by spiking the sample with *L*-aspartic acid (Figure S25). This observation is consistent with the spectrum of the reference compound ***rac*-3f** (Figure S44), suggesting that the formation of aspartic acid may result from the properties and/or instability of the compound.

6. Chemical synthesis of precursors (2a, 2d & 2h)

For the synthesis of substrates **2a**, **2d** & **2h**, published procedures were followed with slight modifications.[5]

6a. Chemical synthesis of 2-((methylamino)methyl)aniline (2a)

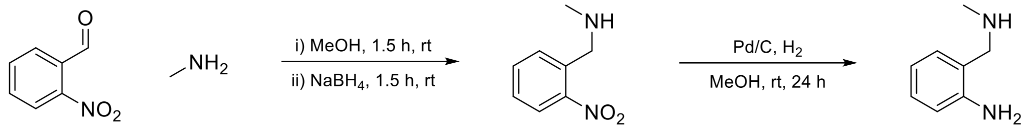

Step 1. To a stirred solution of **S1a** (5.0 g, 33.1 mmol) in methanol (30 mL) under nitrogen atmosphere, a 40% aqueous methylamine solution (3.6 mL, 36.3 mmol) was added dropwise. The solution was left to stir at room temperature for 1.5 h and turned pale yellow. Then, a sodium borohydride solution ($NaBH_4$, 1.0 g in 3.0 mL water, 26.5 mmol) was added over the course of 10 min and the mixture was stirred at room temperature for an additional 1.5 h. After completion of the reaction, methanol was removed under reduced pressure. The residue was re-dissolved in water (15 mL), followed by extraction with dichloromethane (3 x 25 mL). The organic layers were combined, dried over Na_2SO_4, filtered, and evaporated under reduced pressure to give crude intermediate **S2a**, which was used in the next step without further purification.

Step 2. To a stirred solution of **S2a** (5.0 g, 30.1 mmol) in absolute methanol (25 mL) was added Pd/C (640 mg, 6.0 mmol, 10 wt%) under nitrogen atmosphere. The reaction was stirred under hydrogen atmosphere (balloon) at room temperature for 24 h. After completion of the reaction, the reaction mixture was filtered through Celite and the bed rinsed with methanol (10 mL). The filtrate and rinse were combined and concentrated *in vacuo*. The resulting crude was purified with flash chromatography using a gradient of 5-10% methanol/dichloromethane to give **2a** as brown oil in 51% yield.

2-((methylamino)methyl)aniline (2a)

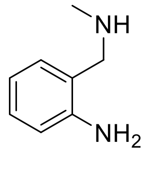

Brown oil, 2.09 g (51% yield). **^{1}H NMR** (600 MHz, Chloroform-*d*) δ 7.11 – 7.07 (m, 1H), 7.04 (d, J = 7.1 Hz, 1H), 6.71 – 6.64 (m, 2H), 3.75 (s, 2H), 2.44 (s, 3H). **^{13}C NMR** (151 MHz, $CDCl_3$) δ 146.87, 129.90, 128.35, 124.04, 117.68, 115.67, 55.12, 36.03. **HRMS** (ESI^+): calcd. for $C_8H_{13}N_2$ $[M+H]^+$: 137.1073, found: 137.1073.

6b. Chemical synthesis of *ortho*-substituted *N*-methyl-1-phenylmethanamine (2d) and (2h)

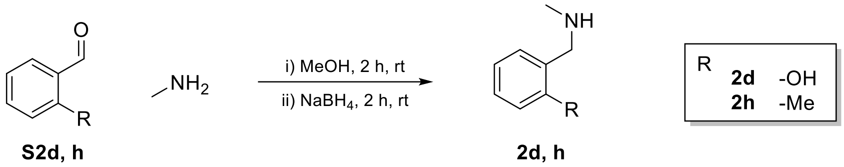

To a stirred solution of **S2d/h** (10.0 mmol) in absolute methanol (10 mL) under nitrogen atmosphere, was added a 40% aqueous methylamine solution (0.95 mL, 11.0 mmol) in a dropwise manner. The reaction mixture was left to stir at room temperature. After 2 h, $NaBH_4$ (320 mg, 10.0 mmol) was added portion-wise and the mixture was stirred for an additional 2 h at room temperature. After completion of the reaction, methanol was removed under reduced pressure. The residue was re-dissolved in water (15 mL) and extracted with ethyl acetate (3 x 15 mL). The organic layers were combined, concentrated under reduced pressure and purified with flash chromatography using a gradient of 5-50% dichloromethane/methanol.

2-((methylamino)methyl)phenol (2d)

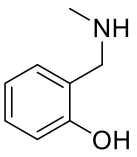

Yellowish solid, 923 mg (67% yield). **^{1}H NMR** (600 MHz, Chloroform-*d*) δ 7.21 – 7.13 (m, 1H), 6.99 (d, *J* = 7.4 Hz, 1H), 6.83 (d, *J* = 8.1 Hz, 1H), 6.81 – 6.74 (m, 1H), 3.97 (s, 2H), 2.48 (s, 3H). **^{13}C NMR** (151 MHz, $CDCl_3$) δ 158.37, 128.70, 128.33, 122.28, 118.92, 116.35, 54.72, 35.23. **HRMS** (ESI$^+$): calcd. for $C_8H_{12}NO$ [M+H]$^+$: 138.0913, found: 138.0914.

***N*-methyl-1-(o-tolyl)methanamine (2h)**

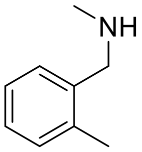

Light yellow oil, 426 mg (32% yield). **^{1}H NMR** (600 MHz, Chloroform-*d*) δ 7.33 – 7.26 (m, 1H), 7.23 – 7.09 (m, 3H), 3.74 (s, 2H), 2.50 (s, 3H), 2.35 (s, 3H). **^{13}C NMR** (151 MHz, $CDCl_3$) δ 138.20, 136.25, 130.25, 128.36, 126.95, 125.89, 53.72, 36.45, 18.91. **HRMS** (ESI$^+$): calcd. for $C_9H_{14}N$ [M+H]$^+$: 136.1121, found: 136.1121.

7. Chemical synthesis of racemic reference compounds

7a. Chemical synthesis of *N*-(2-aminobenzyl)-*N*-methylaspartic acid (*rac*-5a)

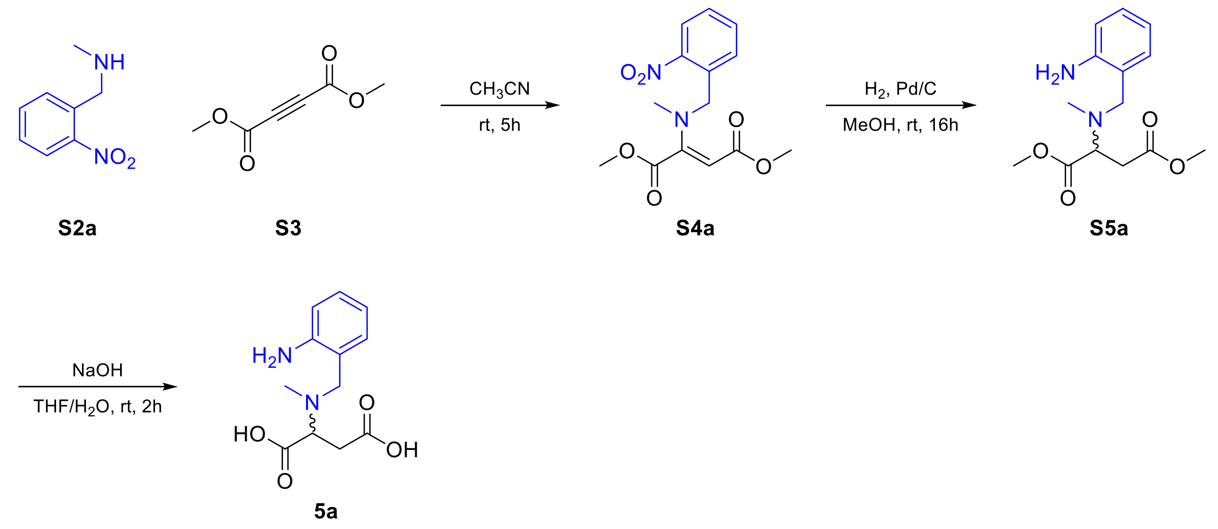

For the synthesis of *rac*-**5a**, published procedures were followed with slight modifications.[3, 5]

Step 1. To a stirred solution of dimethyl acetylenedicarboxylate (**S3**, 312 mg, 264 µL, 2.2 mmol) in acetonitrile (10 mL), **S2a** (616 mg, 3.7 mmol) was added. The reaction mixture was stirred for 5 h at room temperature. After completion of the reaction, the solvent was removed under reduced pressure to give crude product **S4a** as a yellow oil, which was directly used for the next step without purification.

Step 2. To a stirred solution of **S4a** in methanol (10 mL) under nitrogen atmosphere, 10% Pd/C (50% wet, 426 mg, 0.2 mmol) was added. The reaction was stirred under H_2 atmosphere (balloon) overnight at room temperature. After completion of the reaction, the mixture was filtered through Celite and the filtrate was concentrated *in vacuo*. The crude was purified by flash chromatography with a gradient from 5-40% pentane/ethyl acetate to give **S5a** as a yellow oil in 16.4 % yield (101 mg).

Step 3. To a stirred solution of **S5a** (92 mg, 0.3 mmol) in THF (2.0 mL), 0.4 M NaOH (2.0 mL) was added, and the reaction mixture was stirred for 2 h at room temperature. After completion of the reaction, volatiles were removed *in vacuo*. The aqueous layer was acidified with 1 N HCl and subjected to cation exchange chromatography, following the procedure described for its enzymatic counterpart. The ninhydrin-positive fractions were collected and lyophilized to yield the desired product *rac*-**5a** as an ammonium salt in 95.4% yield.

N-(2-aminobenzyl)-*N*-methylaspartic acid (*rac*-5a)

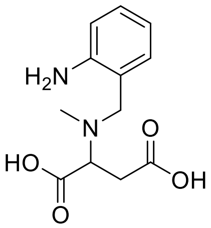

Yellowish solid. 79 mg (16 % overall yield). **^{1}H NMR** (600 MHz, 0.1 M NaOD in Deuterium Oxide) δ 7.17 (t, J = 7.6 Hz, 1H), 7.13 (d, J = 7.3 Hz, 1H), 6.80 (t, J = 7.7 Hz, 2H), 3.60 (s, 2H), 3.56 (t, J = 7.3 Hz, 1H), 2.51 (d, J = 7.2 Hz, 2H), 2.11 (s, 3H). **^{13}C NMR** (151 MHz, 0.1 M NaOD in D_2O) δ 180.82, 179.58, 146.03, 131.38, 128.64, 124.47, 118.82, 116.80, 67.31, 57.38, 38.28, 36.51. **HRMS** (ESI$^+$): calcd. for $C_{12}H_{17}N_2O_4$ [M+H]$^+$: 253.1183, found: 253.1175. **Chiral HPLC**: Chirex 3126 (*D*)-penicillamine column (250 x 4.6 mm, Phenomenex), mobile phase: 2.0 mM aqueous $CuSO_4$, flow rate 1.0 mL/min, 50 °C, UV detection at 254 nm. $T_R(S)$ = 22.8 min, $T_R(R)$ = 33.2 min.

7b. Chemical synthesis of *ortho*-substituted *N*-benzyl-*N*-methylaspartic acids (*rac*-5b-j)

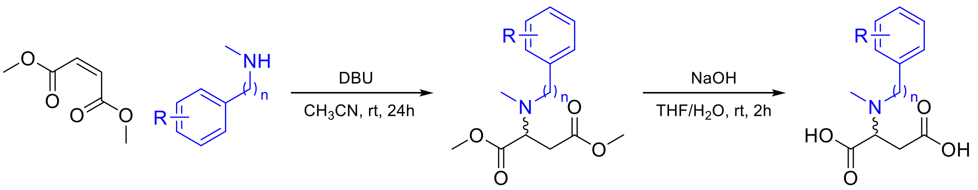

S6 2b-j S5b-j 5b-j

	R=	*n*=
**5b**	*o*-H	1
**5c**	*o*-H	2
**5d**	*o*-OH	1
**5e**	*o*-OMe	1
**5f**	*o*-Br	1
**5g**	*o*-F	1
**5h**	*o*-Me	1
**5i**	*m*-NH_2	1
**5j**	*p*-NH_2	1

For the synthesis of *rac*-**5b-j**, published procedures were followed with slight modifications.[3, 6]

Step 1. To a stirred solution of the appropriate *N*-methylbenzylamine (**2b-j**, 1.0 mmol) and dimethyl maleate (**S6**, 213 mg, 184 µL, 1.5 mmol) in acetonitrile (0.5 mL) at room temperature, was added 1,8-diazabicyclo[5.4.0]undec-7-ene (DBU, 76 mg, 75 µL 0.5 mmol). After 24 h, the solvent was removed under reduced pressure and the crude product was re-dissolved in ethyl acetate (2.0 mL), followed by extraction with 1 N HCl (3x 1.5 mL). The organic layer was washed with brine, filtered over hydrophobic paper, and evaporated under reduced pressure. The crude product was directly used for the next step without purification.

Step 2. To a stirred solution of crude **S5b-j** in THF (2.0 mL) was added 0.2 M NaOH (2.0 mL). The reaction was stirred for 2 h at room temperature. After completion of the reaction, volatiles were removed under reduced pressure and the residue was washed with ethyl acetate (5x 3.0 mL). The aqueous layer was acidified with 1 N HCl and subjected to cation exchange chromatography, following the procedure described for its enzymatic counterpart. The fractions that stained positive with ninhydrin

or potassium permanganate were collected, concentrated under vacuum and lyophilized to provide the desired products **5b-j** as ammonium salts.

***N*-(benzyl)-*N*-methylaspartic acid (*rac*-5b)**

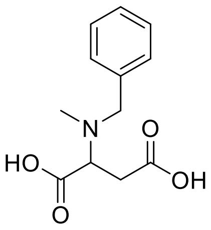

White solid. 27 mg (11 % yield). **^{1}H NMR** (600 MHz, 0.1 M NaOD in Deuterium Oxide) δ 7.41 – 7.35 (m, 4H), 7.35 – 7.30 (m, 1H), 3.73 (d, J = 12.7 Hz, 1H), 3.53 (t, J = 7.2 Hz, 1H), 3.48 (d, J = 12.7 Hz, 1H), 2.64 (dd, J = 15.2, 8.1 Hz, 1H), 2.47 (dd, J = 15.2, 6.4 Hz, 1H), 2.15 (s, 3H). **^{13}C NMR** (151 MHz, 0.1 M NaOD in D_2O) δ 180.32, 178.80, 138.42, 130.03, 128.41, 127.39, 67.21, 58.06, 38.73, 38.23. **HRMS** (ESI$^+$): calcd. for $C_{12}H_{16}NO_4$ [M+H]$^+$: 238.1074, found: 238.1065. **Chiral HPLC**: Nucleosil Chiral-1 column (5 μm, 250 x 4 mm, Macherey-Nagel), mobile phase: 0.5 mM aqueous $CuSO_4$, flow rate 1.0 mL/min, 60 °C, UV detection at 254 nm. T_R(*R*) = 5.0 min, T_R(*S*) = 6.0 min.

***N*-methyl-*N*-phenethylaspartic acid (*rac*-5c)**

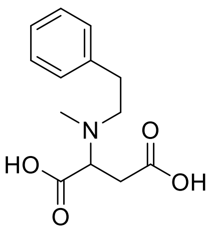

White solid, 42 mg (17% yield). **^{1}H NMR** (600 MHz, 0.1 N DCl in Deuterium Oxide) δ 7.42 (t, J = 7.5 Hz, 2H), 7.35 (t, J = 7.7 Hz, 3H), 4.48 (t, J = 6.1 Hz, 1H), 3.62 – 3.37 (m, 2H), 3.20 – 3.07 (m, 4H), 2.99 (s, 3H). **^{13}C NMR** (151 MHz, 0.1 N DCl in D_2O) δ 173.03, 169.84, 135.79, 129.13, 128.86, 127.50, 30.59, 30.40. **HRMS** (ESI$^+$): calcd. for $C_{13}H_{18}NO_4$ [M+H]$^+$: 252.1230, found: 252.1226. **Chiral HPLC**: Nucleosil Chiral-1 column (5 μm, 250 x 4 mm, Macherey-Nagel), mobile phase: 0.5 mM aqueous $CuSO_4$, flow rate 1.0 mL/min, 60 °C, UV detection at 254 nm. T_R(*1*) = 6.6 min, T_R(*2*) = 10.1 min. *In the recorded ^{13}C NMR, some expected carbon peaks are missing. This observation is consistent with the spectrum of the enzymatic product ***enz*-5c**. Despite the absence of these specific carbon signals, all other data, including HPLC, HRMS and ^{1}H NMR, are consistent with the structure of the expected product.

***N*-(2-hydroxybenzyl)-*N*-methylaspartic acid (*rac*-5d)**

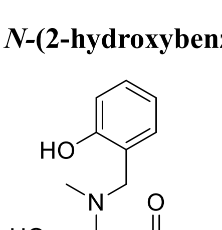

Yellowish solid, 21 mg (8% yield). **^{1}H NMR** (600 MHz, 0.1 M NaOD in Deuterium Oxide) δ 7.23 (d, J = 7.4 Hz, 1H), 7.13 – 7.09 (m, 1H), 6.66 (d, J = 8.0 Hz, 1H), 6.62 (t, J = 7.3 Hz, 1H), 3.68 – 3.59 (m, 3H), 2.69 (dd, J = 15.4, 8.0 Hz, 1H), 2.56 (dd, J = 15.4, 6.2 Hz, 1H), 2.20 (s, 3H). **^{13}C NMR** (151 MHz, 0.1 M NaOD in D_2O) δ 180.51, 179.08, 164.53, 131.11, 128.67, 126.18, 118.85, 114.52, 67.53, 53.31, 38.19, 37.48. **HRMS**

(ESI$^+$): calcd. for $C_{12}H_{16}NO_5$ [M+H]$^+$: 254.1023, found: 254.1017. **Chiral HPLC**: Chirex 3126 (*D*)-penicillamine column (250 x 4.6 mm, Phenomenex), phase A: 2.0 mM aqueous $CuSO_4$, phase B: isopropanol, 10% B (v/v), flow rate 1.0 mL/min, 50 °C, UV detection at 254 nm. T_R(*S*) = 14.0 min, T_R(*R*) = 14.7 min.

***N*-(2-methoxybenzyl)-*N*-methylaspartic acid (*rac*-5e)**

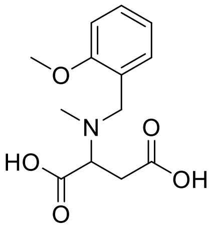

Yellowish solid, 72 mg (27% yield). **^{1}H NMR** (600 MHz, 0.1 M NaOD in Deuterium Oxide) δ 7.42 – 7.30 (m, 2H), 7.07 (d, *J* = 8.2 Hz, 1H), 7.02 (t, *J* = 7.4 Hz, 1H), 3.86 (s, 3H), 3.79 (d, *J* = 12.6 Hz, 1H), 3.59 – 3.55 (m, 1H), 3.49 (d, *J* = 12.7 Hz, 1H), 2.74 (dd, *J* = 15.5, 8.9 Hz, 1H), 2.54 (dd, *J* = 15.5, 5.4 Hz, 1H), 2.21 (s, 3H). **^{13}C NMR** (151 MHz, 0.1 M NaOD in D_2O) δ 180.36, 178.56, 157.87, 132.02, 129.15, 126.24, 120.54, 111.49, 67.50, 55.44, 51.98, 38.63, 38.55. **HRMS** (ESI$^+$): calcd. for $C_{13}H_{18}NO_5$ [M+H]$^+$: 268.1179, found: 268.1173. **Chiral HPLC**: Chirex 3126 (*D*)-penicillamine column (250 x 4.6 mm, Phenomenex), phase A: 2.0 mM aqueous $CuSO_4$, phase B: isopropanol, 5% B (v/v), flow rate of 1.0 mL/min, 50 °C, UV detection at 254 nm. T_R(*1*) = 15.7 min, T_R(*2*) = 16.8 min.

***N*-(2-bromobenzyl)-*N*-methylaspartic acid (*rac*-5f)**

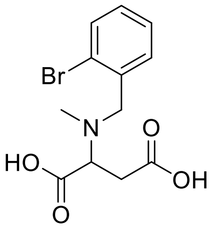

Yellowish solid, 108 mg (34% yield). **^{1}H NMR** (600 MHz, 0.1 M NaOD in Deuterium Oxide) δ 7.63 (d, *J* = 8.0 Hz, 1H), 7.44 (d, *J* = 7.6 Hz, 1H), 7.37 (t, *J* = 7.5 Hz, 1H), 7.22 (t, *J* = 7.6 Hz, 1H), 3.81 (d, *J* = 13.1 Hz, 1H), 3.71 (d, *J* = 13.1 Hz, 1H), 3.64 (dd, *J* = 8.4, 6.3 Hz, 1H), 2.75 – 2.68 (m, 2H), 2.54 (dd, *J* = 15.3, 6.0 Hz, 1H), 2.20 (s, 3H). **^{13}C NMR** (151 MHz, 0.1 M NaOD in D_2O) δ 180.34, 178.49, 137.42, 132.86, 132.29, 129.21, 127.50, 124.92, 68.00, 57.97, 38.81, 38.14. **HRMS** (ESI$^+$): calcd. for $C_{12}H_{15}NO_4Br$ [M+H]$^+$: 316.0179, found: 316.0178. **Chiral HPLC**: Nucleosil Chiral-1 column (5 µm, 250 x 4 mm, Macherey-Nagel), mobile phase: 0.5 mM aqueous $CuSO_4$, flow rate 1.0 mL/min, 60 °C, UV detection at 254 nm. T_R(*1*) = 5.5 min, T_R(*2*) = 6.1 min.

***N*-(2-fluorobenzyl)-*N*-methylaspartic acid (*rac*-5g)**

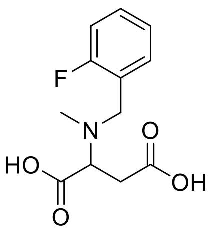

Yellowish solid, 77 mg (30% yield). **^{1}H NMR** (600 MHz, 0.1 M NaOD in Deuterium Oxide) δ 7.46 (t, *J* = 7.9 Hz, 1H), 7.44 – 7.39 (m, 1H), 7.24 (t, *J* = 7.4 Hz, 1H), 7.19 (t, *J* = 9.3 Hz, 1H), 4.03 (d, *J* = 12.9 Hz, 1H), 3.86 (d, *J* = 13.0 Hz, 1H), 3.73 (t, *J* = 7.0 Hz, 1H), 2.76 (dd, *J* = 16.0, 7.4 Hz, 1H), 2.59 (dd, *J* = 16.0, 6.8 Hz, 1H), 2.42 (s, 3H). **^{13}C NMR** (151 MHz, 0.1 M NaOD in D_2O) δ 179.29, 176.46, 161.43 (d, *J* = 245.6 Hz), 132.68 (d, *J* = 3.8 Hz), 130.52 (d, *J* = 8.4 Hz), 124.42 (d, *J* = 3.5 Hz), 122.48 (d, *J* = 15.2 Hz), 115.53 (d, *J* = 21.6 Hz), 66.93, 51.33, 37.92, 37.42. **HRMS** (ESI$^+$): calcd. for $C_{12}H_{15}NO_4F$ [M+H]$^+$: 256.0980, found: 256.0965. **Chiral HPLC**: Nucleosil Chiral-1 column (5 µm, 250 x 4 mm, Macherey-Nagel), mobile phase: 0.5 mM aqueous $CuSO_4$, flow rate 1.0 mL/min, 60 °C, UV detection at 254 nm. $T_R(1)$ = 4.8 min, $T_R(2)$ = 5.5 min.

***N*-methyl-*N*-(2-methylbenzyl)aspartic acid (*rac*-5h)**

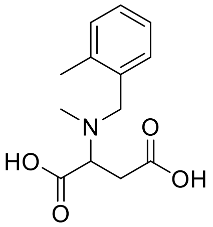

Yellowish solid, 121 mg (48% yield). **^{1}H NMR** (600 MHz, 0.1 M NaOD in Deuterium Oxide) δ 7.35 (d, *J* = 6.9 Hz, 1H), 7.30 – 7.22 (m, 3H), 3.71 (d, *J* = 12.9 Hz, 1H), 3.66 – 3.58 (m, 2H), 2.69 (dd, *J* = 15.2, 8.0 Hz, 1H), 2.53 (dd, *J* = 15.2, 6.6 Hz, 1H), 2.37 (s, 3H), 2.19 (s, 3H). **^{13}C NMR** (151 MHz, 0.1 M NaOD in D_2O) δ 180.39, 178.70, 138.06, 136.60, 130.91, 130.36, 127.52, 125.71, 68.05, 55.69, 38.65, 37.91, 18.61. **HRMS** (ESI$^+$): calcd. for $C_{13}H_{18}NO_4$ [M+H]$^+$: 252.1230, found: 252.1217. **Chiral HPLC**: Nucleosil Chiral-1 column (5 µm, 250 x 4 mm, Macherey-Nagel), mobile phase: 0.5 mM aqueous $CuSO_4$, flow rate 1.0 mL/min, 60 °C, UV detection at 254 nm. $T_R(R)$ = 5.3 min, $T_R(S)$ = 6.1 min.

***N*-(3-aminobenzyl)-*N*-methylaspartic acid (*rac*-5i)**

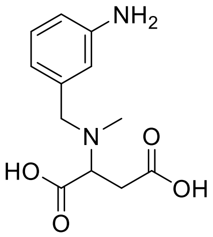

Yellowish solid. 144 mg (57% yield). **^{1}H NMR** (600 MHz, 0.1 M NaOD in Deuterium Oxide) δ 7.22 (t, *J* = 7.7 Hz, 1H), 6.86 (d, *J* = 7.1 Hz, 2H), 6.79 (d, *J* = 8.0 Hz, 1H), 3.74 (d, *J* = 12.7 Hz, 1H), 3.60 (t, *J* = 7.2 Hz, 1H), 3.50 (d, *J* = 12.7 Hz, 1H), 2.68 (dd, *J* = 15.4, 7.6 Hz, 1H), 2.52 (dd, *J* = 15.4, 6.8 Hz, 1H), 2.24 (s, 3H). **^{13}C NMR** (151 MHz, 0.1 M NaOD in D_2O) δ 180.06, 178.24, 146.21, 139.02, 129.37, 121.07, 117.82, 115.48, 67.05, 58.05, 38.37, 38.19. **HRMS** (ESI$^+$): calcd. for $C_{12}H_{17}N_2O_4$ [M+H]$^+$: 253.1183, found: 253.1180. **Chiral HPLC**: Chirex 3126 (*D*)-penicillamine column (250 x 4.6 mm, Phenomenex), phase A: 2.0 mM aqueous $CuSO_4$, phase B: isopropanol, 10% B (v/v), flow rate 1.0 mL/min, 50 °C, UV

detection at 254 nm. T_R(*1*) = 11.0 min, T_R(*2*) = 12.3 min.

***N*-(4-aminobenzyl)-*N*-methylaspartic acid (*rac*-5j)**

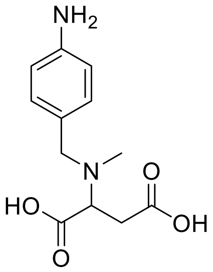

Yellowish solid, 201 mg (80% yield). **^{1}H NMR** (600 MHz, 0.1 M NaOD in Deuterium Oxide) δ 7.20 (d, *J* = 8.0 Hz, 2H), 6.83 (d, *J* = 8.0 Hz, 2H), 3.65 (d, *J* = 12.6 Hz, 1H), 3.54 (t, *J* = 7.2 Hz, 1H), 3.41 (d, *J* = 12.6 Hz, 1H), 2.66 (dd, *J* = 15.3, 8.1 Hz, 1H), 2.48 (dd, *J* = 15.3, 6.3 Hz, 1H), 2.16 (s, 3H). **^{13}C NMR** (151 MHz, 0.1 M NaOD in D_2O) δ 180.31, 178.78, 145.27, 131.15, 129.10, 116.18, 67.04, 57.51, 38.74, 37.97. **HRMS** (ESI$^+$): calcd. for $C_{12}H_{17}N_2O_4$ [M+H]$^+$: 253.1183, found: 253.1186. **Chiral HPLC**: Chirex 3126 (*D*)-penicillamine column (250 x 4.6 mm, Phenomenex), phase A: 2.0 mM aqueous $CuSO_4$, phase B: isopropanol, 10% B (v/v), flow rate 1.0 mL/min, 50 °C, UV detection at 254 nm. T_R(*1*) = 8.9 min, T_R(*2*) = 11.0 min.

7c. Chemical synthesis of *ortho*-substituted phenylaspartic acids (*rac*-3b-f)

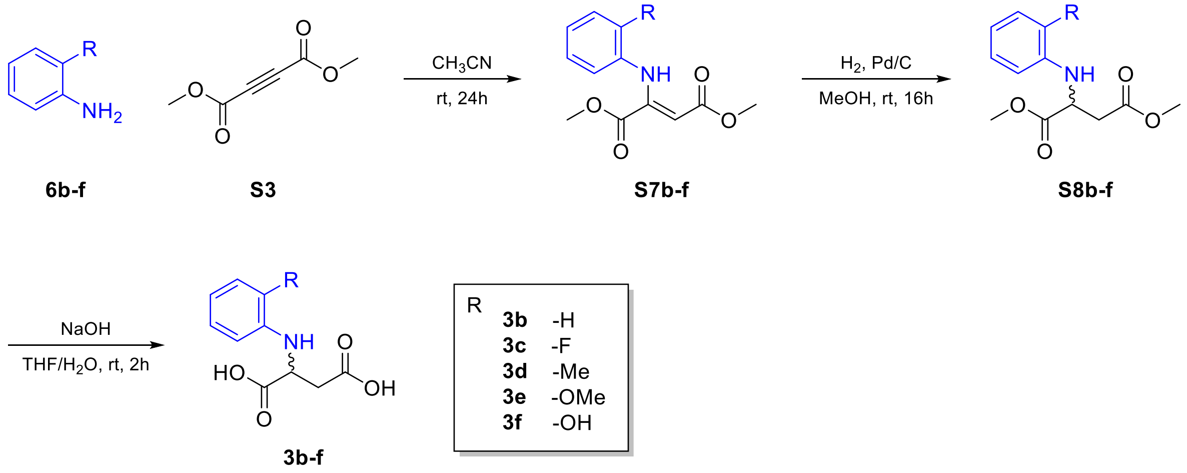

For the synthesis of *rac*-**3b-f**, published procedures were followed with slight modifications.[3]

Step 1. To a stirred solution of dimethyl acetylenedicarboxylate (**S3**, 156 mg, 132 μL, 1.1 mmol) in acetonitrile (5.0 mL) was added *ortho*-substituted aniline (**6b-f**, 1.0 mmol). The reaction mixture was stirred at room temperature for 24-48 h. After completion of the reaction, the solvent was removed under reduced pressure to give crude product **S7b-f**, which was directly used for the next step without purification.

Step 2. To a stirred solution of **S7b-f** in methanol (5.0 mL) was added 10% Pd/C (50% wet, 22 mg, 0.01 mmol) under nitrogen atmosphere. The reaction was stirred under H_2 atmosphere (balloon) for 16 h at

room temperature. After completion of the reaction, the mixture was filtered through Celite and the filtrate was concentrated *in vacuo* providing crude **S8b-f**, which was directly used for the next step without purification

Step 3. To a stirred solution of **S8b-f** in THF (2.0 mL) was added 0.4 M NaOH (2.0 mL), and the reaction mixture was stirred for 2 h at room temperature. After completion of the reaction, volatiles were removed *in vacuo*. The aqueous layer was acidified with 1 N HCl and subjected to cation exchange chromatography, following the procedure described for its enzymatic counterpart. The ninhydrin-positive fractions were collected, concentrated *in vacuo*, and lyophilized to yield the desired products **3b-f** as ammonium salts.

phenylaspartic acid (*rac*-3b)

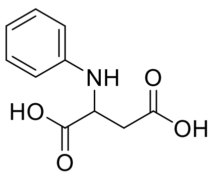

White solid, 139 mg (66% yield). **^{1}H NMR** (600 MHz, 0.1 M NaOD in Deuterium Oxide) δ 7.19 (t, *J* = 7.6 Hz, 2H), 6.74 (t, *J* = 7.3 Hz, 1H), 6.67 (d, *J* = 7.8 Hz, 2H), 4.07 (dd, *J* = 9.7, 3.7 Hz, 1H), 2.64 (dd, *J* = 14.9, 3.4 Hz, 1H), 2.39 (dd, *J* = 14.6, 10.3 Hz, 1H). **^{13}C NMR** (151 MHz, 0.1 M NaOD in D_2O) δ 181.56, 179.49, 147.87, 129.47, 118.35, 114.15, 57.66, 41.23. **HRMS** (ESI$^+$): calcd. for $C_{10}H_{12}NO_4$ $[M+H]^+$: 210.0761, found: 210.0759. **Chiral HPLC**: Nucleosil Chiral-1 column (5 μm, 250 x 4 mm, Macherey-Nagel), mobile phase: 0.5 mM aqueous $CuSO_4$, flow rate 1.0 mL/min, 60 °C, UV detection at 254 nm. T_R(*R*) = 4.2 min T_R(*S*) = 5.8 min.

(2-fluorophenyl)aspartic acid (*rac*-3c)

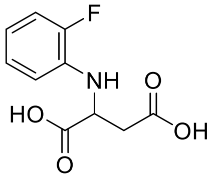

Yellowish solid, 77 mg (34% yield). **^{1}H NMR*** (600 MHz, 0.1 M NaOD in Deuterium Oxide) δ 7.16 – 7.02 (m, 2H), 6.77 (t, *J* = 9.0 Hz, 2H), 4.17 (dd, *J* = 10.2, 3.6 Hz, 1H), 2.76 (dd, *J* = 14.9, 3.7 Hz, 1H), 2.51 (dd, *J* = 14.9, 10.3 Hz, 1H). **^{13}C NMR*** (151 MHz, 0.1 M NaOD in D_2O) δ 181.10, 179.39, 151.81 (d, *J* = 238.0 Hz), 135.94 (d, *J* = 11.9 Hz), 124.85 (d, *J* = 3.4 Hz), 118.09 (d, *J* = 7.0 Hz), 114.76 (d, *J* = 18.5 Hz), 113.82 (d, *J* = 2.9 Hz), 57.33, 41.09. **HRMS** (ESI$^+$): calcd. for $C_{10}H_{11}NO_4F$ $[M+H]^+$: 228.0667, found: 228.0666. **Chiral HPLC**: Nucleosil Chiral-1 column (5 μm, 250 x 4 mm, Macherey-Nagel), mobile phase: 0.5 mM aqueous $CuSO_4$, flow rate 1.0 mL/min, 60 °C, UV detection at 254 nm. T_R(*R*) = 4.2 min T_R(*S*) = 5.0 min.

_o_-tolylaspartic acid (_rac_-3d)

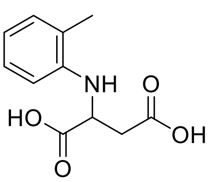

White solid, 70 mg (31% yield). **^{1}H NMR** (600 MHz, 0.1 M NaOD in Deuterium Oxide) δ 7.20 – 7.13 (m, 2H), 6.76 (t, *J* = 7.3 Hz, 1H), 6.63 (d, *J* = 7.9 Hz, 1H), 4.14 (dd, *J* = 10.0, 3.5 Hz, 1H), 2.75 (dd, *J* = 14.5, 3.7 Hz, 1H), 2.53 (dd, *J* = 14.4, 10.2 Hz, 1H), 2.20 (s, 3H). **^{13}C NMR** (151 MHz, 0.1 M NaOD in D_2O) δ 181.53, 179.57, 145.76, 130.24, 127.21, 123.86, 118.03, 111.24, 57.69, 41.19, 16.65. **HRMS** (ESI^+): calcd. for $C_{11}H_{14}NO_4$ $[M+H]^+$: 224.0917, found: 244.0922. **Chiral HPLC**: Nucleosil Chiral-1 column (5 µm, 250 x 4 mm, Macherey-Nagel), mobile phase: 0.5 mM aqueous $CuSO_4$, flow rate 0.8 mL/min, 60 °C, UV detection at 254 nm. . T_R(*R*) = 6.1 min T_R(*S*) = 7.4 min.

(2-methoxyphenyl)aspartic acid (enz-3e)

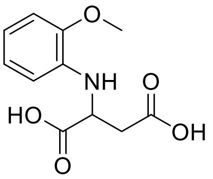

White solid, 170 mg (71% yield). **^{1}H NMR** (600 MHz, 0.1 M NaOD in Deuterium Oxide) δ 6.98 (d, *J* = 8.0 Hz, 1H), 6.94 (t, *J* = 7.6 Hz, 1H), 6.81 (t, *J* = 7.7 Hz, 1H), 6.68 (d, *J* = 7.8 Hz, 1H), 4.15 (dd, *J* = 10.1, 3.5 Hz, 1H), 3.89 (s, 3H), 2.74 (dd, *J* = 14.7, 3.6 Hz, 1H), 2.50 (dd, *J* = 14.5, 10.3 Hz, 1H). **^{13}C NMR** (151 MHz, 0.1 M NaOD in D_2O) δ 181.44, 179.49, 147.41, 137.60, 121.72, 118.12, 111.82, 111.24, 57.49, 56.04, 41.33. **HRMS** (ESI^+): calcd. for $C_{11}H_{14}NO_5$ $[M+H]^+$: 240.0866, found: 240.0869. **Chiral HPLC**: Nucleosil Chiral-1 column (5 µm, 250 x 4 mm, Macherey-Nagel), mobile phase: 0.5 mM aqueous $CuSO_4$, flow rate 0.8 mL/min, 60 °C, UV detection at 254 nm. T_R(*1*) = 6.4 min T_R(*2*) = 7.1 min.

(2-hydroxyphenyl)aspartic acid (_rac_-3f)

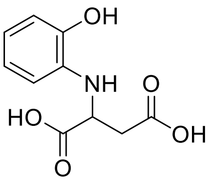

Dark brown solid, 81 mg (80 w% **3f**, 29% yield). **^{1}H NMR*** (600 MHz, 0.1 M NaOD in Deuterium Oxide) δ 6.60 (t, *J* = 7.4 Hz, 1H), 6.57 – 6.44 (m, 3H), 4.08 (dd, *J* = 9.3, 4.2 Hz, 1H), 2.67 (dd, *J* = 14.5, 4.3 Hz, 1H), 2.49 (dd, *J* = 14.5, 9.7 Hz, 1H). **^{13}C NMR*** (151 MHz, 0.1 M NaOD in D_2O) δ 182.22, 179.90, 154.98, 139.53, 119.03, 116.21, 114.54, 111.29, 58.04, 41.76. **HRMS** (ESI^+): calcd. for $C_{10}H_{12}NO_5$ $[M+H]^+$: 226.0710, found: 226.0712. **Chiral HPLC**: Chirex 3126 (*D*)-penicillamine column (250 x 4.6 mm, Phenomenex), phase A: 2.0 mM aqueous $CuSO_4$, phase B: isopropanol, 2% B (v/v), flow rate 1.0 mL/min, 50 °C, UV detection at 254 nm. T_R(*S*) = 11.6 min and T_R(*R*) = 12.1 min.[4] *Both ^{1}H and ^{13}C NMR spectra (Figure S44) contain traces of aspartic acid (Figure S25). This observation is consistent with the spectrum of the enzymatic products ***enz*-3f** (Figure S24 & S26), suggesting that the formation of aspartic acid may result

from the properties and/or instability of the compound.

7d. Chemical synthesis of (methylamino)methyl-substituted phenylaspartic acids (*rac*-3a, -3i & -3j)

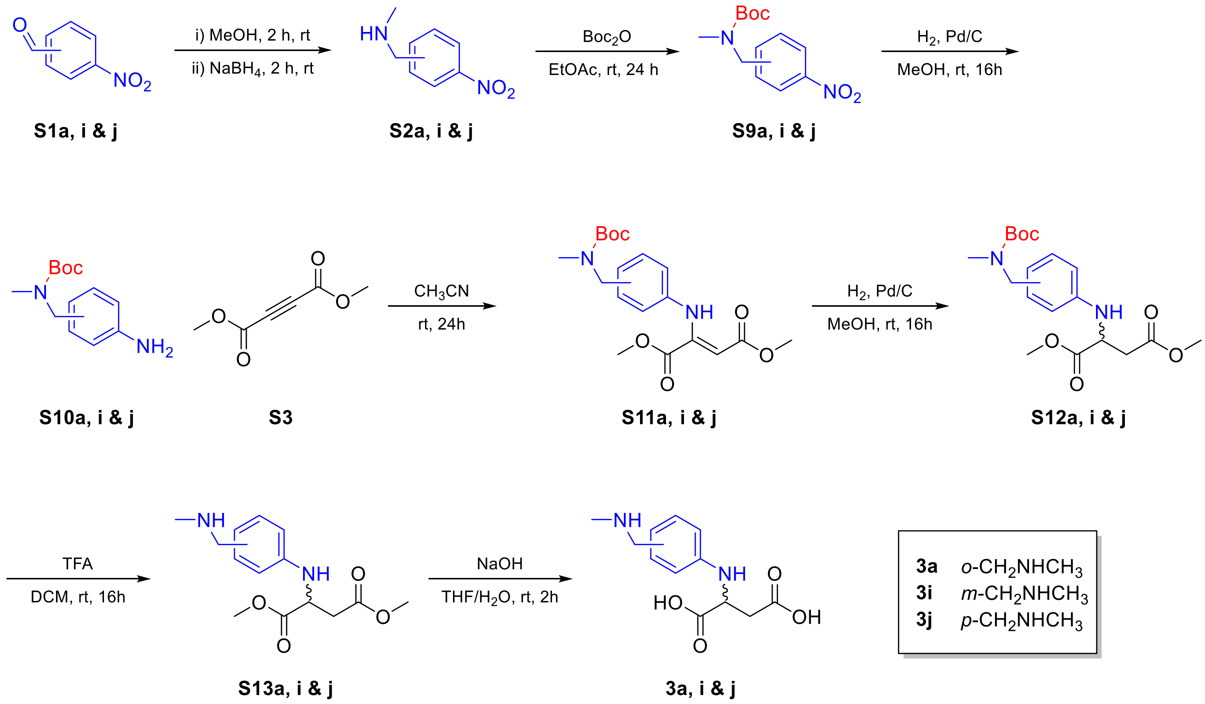

For the synthesis of *rac*-**3a, -i** & **-j**, published procedures were followed with slight modifications.[3, 5]

Step 1. Procedure for reductive amination were followed as previously described for the synthesis of **2a**, **d** & **h**. To a stirred solution of nitrobenzaldehyde (**S1a, -i** & **-j**, 1.5 g, 10.0 mmol) in absolute methanol (10 mL) under nitrogen atmosphere, was added 40% aqueous methylamine solution (0.95 mL, 11.0 mmol) in a dropwise manner. The reaction mixture was left to stir at room temperature. After 2 h, $NaBH_4$ (320 mg, 10.0 mmol) was added portion-wise and the mixture was stirred for an additional 2 h at room temperature. After completion of the reaction, the solvent was removed under reduced pressure. The residue was re-dissolved in water (10 mL) and extracted with ethyl acetate (3 x 15 mL). The organic layers were combined, concentrated under pressure and purified with flash chromatography using a gradient of 5-50% dichloromethane/methanol, resulting in product **S2**.

Step 2. To a stirred solution of **S2** (500 mg, 3.0 mmol) in ethyl acetate, Boc anhydride (Boc_2O, 1.3 g, 6.0 mmol) was added in a portion-wise manner. After 24 h, the solvent was removed *in vacuo*. The residue was re-dissolved in ethanol (10 mL) and imidazole (240 mg, 3.5 mmol) was added. The solution was allowed to stir for 2 h at room temperature, after which the mixture was extracted with 0.1 N HCl

(3x 10 mL). The organic layer was collected and solvent removed under reduced pressure to yield crude **S9**, which was used for the next step without further purification.

Step 3. To a stirred solution of the Boc-protected **S9** in methanol (20 mL) under nitrogen atmosphere, Pd/C (10%, 50%wet, 43 mg, 0.02 mmol) was added. The reaction was stirred under H_2 atmosphere (balloon) for 16 h at room temperature. After completion of the reaction, the mixture was filtered through Celite and the filtrate was concentrated under reduced pressure. The crude was purified with flash chromatography using a gradient of 5-50% pentane/ethyl acetate, to provide product **S10**.

Step 4. To a stirred solution of **S10** (504 mg, 2.1 mmol) in acetonitrile (10 mL), a solution containing dimethyl acetylenedicarboxylate (**S3**, 284 mg, 246 µL, 2.0 mmol) in acetonitrile (5.0 mL) was added. The reaction mixture was stirred at room temperature for 24 h. After completion of the reaction, the solvent was removed under reduced pressure to give crude product **S11**, which was directly used for the next step without purification.

Step 5. To a stirred solution of crude **S11** in methanol (10 mL) under nitrogen atmosphere, was added 10% Pd/C (50% wet, 43 mg, 0.02 mmol) was added. The reaction was stirred under H_2 atmosphere (balloon) for 16 h at room temperature. After completion of the reaction, the mixture was filtered through Celite and the filtrate was concentrated under reduced pressure, to yield crude crude **S12**.

Step 6. To a stirred solution of the Boc-protected **S12** in dichloromethane (8.0 mL), trifluoroacetic acid (TFA, 2.0 mL) was added in a dropwise manner. The reaction was allowed to stir for 16 h room temperature. After completion of the reaction, the solvent was removed under reduced pressure. The residue was redissolved in 30 mL of dichloromethane and removed, which was repeated 3 times to remove any residual TFA.

Step 7. To a stirred solution of **S13** (500 mg, 1.7 mmol) in THF (5.0 mL) was added 0.4 M NaOH (5.0 mL), and the reaction mixture was stirred for 2 h at room temperature. After completion of the reaction, volatiles were removed *in vacuo*. The aqueous layer was acidified with 1 N HCl and subjected to cation exchange chromatography, as described previously. The ninhydrin-positive fractions were

collected, concentrated *in vacuo*, and lyophilized to yield the desired products rac-**3a, -3i & -3j** as ammonium salts.

(2-((methylamino)methyl)phenyl)aspartic acid (*rac*-3a)

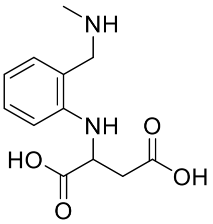

White solid, 47 mg (8% yield). **^{1}H NMR** (600 MHz, 0.1 M NaOD in Deuterium Oxide) δ 7.27 – 7.16 (m, 2H), 6.80 (t, *J* = 7.0 Hz, 1H), 6.65 (d, *J* = 7.7 Hz, 1H), 4.12 (d, *J* = 8.5 Hz, 1H), 3.66 (s, 2H), 2.76 (d, *J* = 12.7 Hz, 1H), 2.55 – 2.46 (m, 1H), 2.37 (s, 3H). **^{13}C NMR** (151 MHz, 0.1 M NaOD in D_2O) δ 181.59, 179.56, 145.62, 129.92, 128.75, 124.33, 117.77, 111.80, 57.65, 50.55, 41.22, 34.57. **HRMS** (ESI$^+$): calcd. for $C_{12}H_{17}N_2O_4$ [M+H]$^+$: 253.1183, found: 253.1182. **Chiral HPLC**: Chirex 3126 (*D*)-penicillamine column (250 x 4.6 mm, Phenomenex), mobile phase: 2.0 mM aqueous $CuSO_4$, flow rate 1.0 mL/min, 50 °C, UV detection at 254 nm. T_R(*1*) = 30.5 min, T_R(*2*) = 34.3 min.

(3-((methylamino)methyl)phenyl)aspartic acid (*rac*-3i)

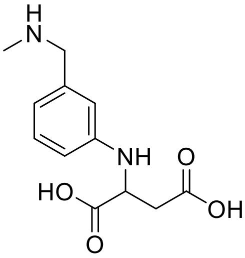

Yellow solid, 213 mg (28% yield). **^{1}H NMR** (600 MHz, 0.1 M NaOD in Deuterium Oxide) δ 7.24 (t, *J* = 7.8 Hz, 1H), 6.76 (d, *J* = 7.4 Hz, 1H), 6.71 (d, *J* = 8.2 Hz, 1H), 6.68 (s, 1H), 4.15 (dd, *J* = 10.3, 3.0 Hz, 1H), 3.79 (s, 2H), 2.73 (dd, *J* = 14.8, 3.2 Hz, 1H), 2.45 (s, 4H). **^{13}C NMR** (151 MHz, 0.1 M NaOD in D_2O) δ 181.50, 179.43, 148.28, 137.38, 129.75, 118.54, 113.93, 113.78, 57.68, 53.74, 41.19, 33.36. **HRMS** (ESI$^+$): calcd. for $C_{12}H_{17}N_2O_4$ [M+H]$^+$: 253.1183, found: 253.1179. **Chiral HPLC**: Chirex 3126 (*D*)-penicillamine column (250 x 4.6 mm, Phenomenex), phase A: 2.0 mM aqueous $CuSO_4$, phase B: isopropanol, 10% B (v/v), flow rate 1.0 mL/min, 50 °C, UV detection at 254 nm. T_R(*1*) = 9.5 min, T_R(*2*) = 29.5 min.

(4-((methylamino)methyl)phenyl)aspartic acid (*rac*-3j)

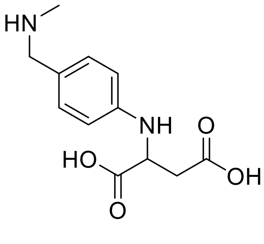

Yellow solid, 111 mg (43% yield). **^{1}H NMR** (600 MHz, 0.1 M NaOD in Deuterium Oxide) δ 7.25 (d, *J* = 8.2 Hz, 2H), 6.73 (d, *J* = 8.2 Hz, 2H), 4.15 (dd, *J* = 10.3, 3.1 Hz, 1H), 4.06 (s, 2H), 2.75 (dd, *J* = 14.9, 3.3 Hz, 1H), 2.67 (s, 3H), 2.46 (dd, *J* = 14.8, 10.6 Hz, 1H). **^{13}C NMR** (151 MHz, 0.1 M NaOD in D_2O) δ 181.20, 179.19, 148.88, 131.10, 119.57, 113.85, 57.37, 52.19, 40.92, 31.65. **HRMS** (ESI$^+$): calcd. for $C_{12}H_{17}N_2O_4$ [M+H]$^+$: 253.1183, observed fragment ion: 222.0758 (corresponding to the loss of CH_4N from the parent compound). **Chiral HPLC**: Chirex 3126 (*D*)-penicillamine column (250 x 4.6

mm, Phenomenex), phase A: 2.0 mM aqueous $CuSO_4$, phase B: isopropanol, 10% B (v/v), flow rate 1.0 mL/min, 50 °C, UV detection at 254 nm. $T_R(1) = 7.4$ min, $T_R(2) = 25.4$ min.

8. Chemical synthesis of enantioenriched *ortho*-substituted *N*-benzyl-*N*-methyl-*L*-aspartic acids ((*S*)-5a, -5b, -5d & -5h)

For the synthesis of enantioenriched reference compounds, published procedures were followed with slight modifications.[3, 7]

8a. Chemical synthesis of *N*-(2-aminobenzyl)-*N*-methyl-*L*-aspartic acid ((*S*)-5a)

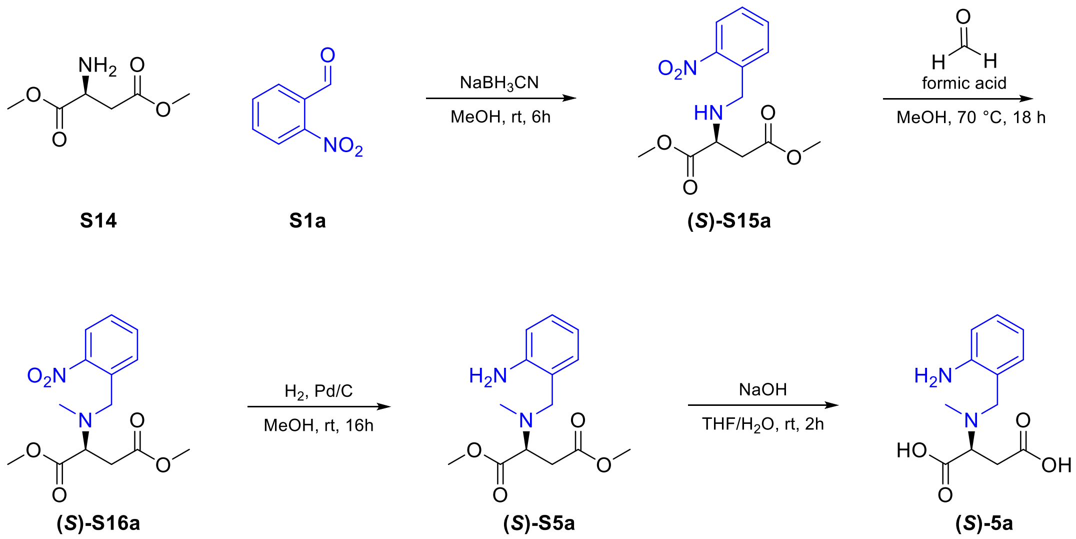

Step 1. To a stirred solution of *L*-dimethyl aspartate hydrochloride (**S14**, 455 mg, 2.3 mmol) and sodium cyanoborohydride ($NaBH_3CN$, 168 mg, 2.7 mmol) in methanol (12 mL) was added **S1a** (384 mg, 2.5 mmol). The reaction mixture was stirred for 6 h. After completion of the reaction, the solvent was removed under reduced pressure. The residue was re-dissolved in a minimum volume of water and the pH was raised to ca. pH 9.0 using saturated aqueous Na_2CO_3, followed by extraction with ethyl acetate (3 x 15 mL). The combined organic portions were concentrated under reduced pressure, and purified with flash chromatography using 30% pentane/ethyl acetate to give **(*S*)-S15a** as a yellow oil in 44% yield (298 mg).

Step 2. To a stirred solution of formaldehyde (37% solution, 7.4 mL, 200 mmol) and formic acid (6.6 mL, 175 mmol) in methanol (7.5 mL) was added *N*-benzyl-*N*-methyl-*L*-aspartate ester **(*S*)-S15a** (298 mg, 1.0 mmol). The reaction mixture was heated to reflux for 4.5 h under nitrogen atmosphere, giving a clear light-yellow solution. After completion of the reaction, volatiles were removed under reduced

pressure. The aqueous layer was basified with NaH_2CO_3 to pH 9.0, and extracted with ethyl acetate (3 x 10 mL). The organic layers were combined, filtered through hydrophobic filter paper and evaporated under reduced pressure. The crude product was purified with flash chromatography using a mixture of acetone, heptane, and triethylamine (7:90:3) to give **(*S*)-S16a** as a yellowish oil in 48% yield (151 mg).

Step 3. To a stirred solution of **(*S*)-S16a** (30 mg, 0.1 mmol) in methanol (5.0 mL) was added 10% Pd/C (50%wet, 2 mg, 0.001 mmol) under nitrogen atmosphere. The reaction was stirred under H_2 atmosphere (balloon) overnight at room temperature. After completion of the reaction, the mixture was filtered through Celite and the filtrate was concentrated *in vacuo* providing crude **(*S*)-S5a**, which was directly used for the next step.

Step 4. To a stirred solution of crude **(*S*)-S5a** (29 mg, 0.1 mmol) in THF (2.0 mL) was added 0.4 M NaOH (2.0 mL), and the reaction mixture was stirred at room temperature for 2 h. After completion of the reaction, volatiles were removed *in vacuo*. The aqueous layer was acidified with 1 N HCl and subjected to cation exchange chromatography, following the procedure described for its enzymatic counterpart. The ninhydrin-positive fractions were collected and lyophilized to yield the desired product **(*S*)-5a** as ammonium salt with 82% yield over the last two steps (20 mg).

***N*-(2-aminobenzyl)-*N*-methyl-*L*-aspartic acid ((*S*)-5a)**

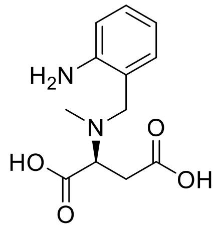

Yellowish solid, 20 mg (17% overall yield). **^{1}H NMR** (600 MHz, 0.1 M NaOD in Deuterium Oxide) δ 7.15 (t, *J* = 7.6 Hz, 1H), 7.12 (d, *J* = 7.3 Hz, 1H), 6.79 (t, *J* = 7.7 Hz, 2H), 3.59 (s, 2H), 3.54 (t, *J* = 7.3 Hz, 1H), 2.49 (d, *J* = 7.1 Hz, 2H), 2.10 (s, 3H). **^{13}C NMR** (151 MHz, 0.1 M NaOD in D_2O) δ 180.82, 179.58, 146.02, 131.37, 128.64, 124.46, 118.81, 116.79, 67.30, 57.35, 38.26, 36.52. **HRMS** (ESI^+): calcd. for $C_{12}H_{17}N_2O_4$ $[M+H]^+$: 253.1183, found: 253.1181. **Chiral HPLC**: Chirex 3126 (*D*)-penicillamine column (250 x 4.6 mm, Phenomenex), mobile phase: 2.0 mM aqueous $CuSO_4$, flow rate 1.0 mL/min, 50 °C, UV detection at 254 nm. $T_R(S)$ = 22.5 min, $T_R(R)$ = 33.6. The *ee* was determined to be 91% (*S*) by chiral HPLC analysis using a racemic standard.

8b. Chemical synthesis of *N*-benzyl-*N*-methyl-*L*-aspartic acid ((*S*)-5b, d & h)

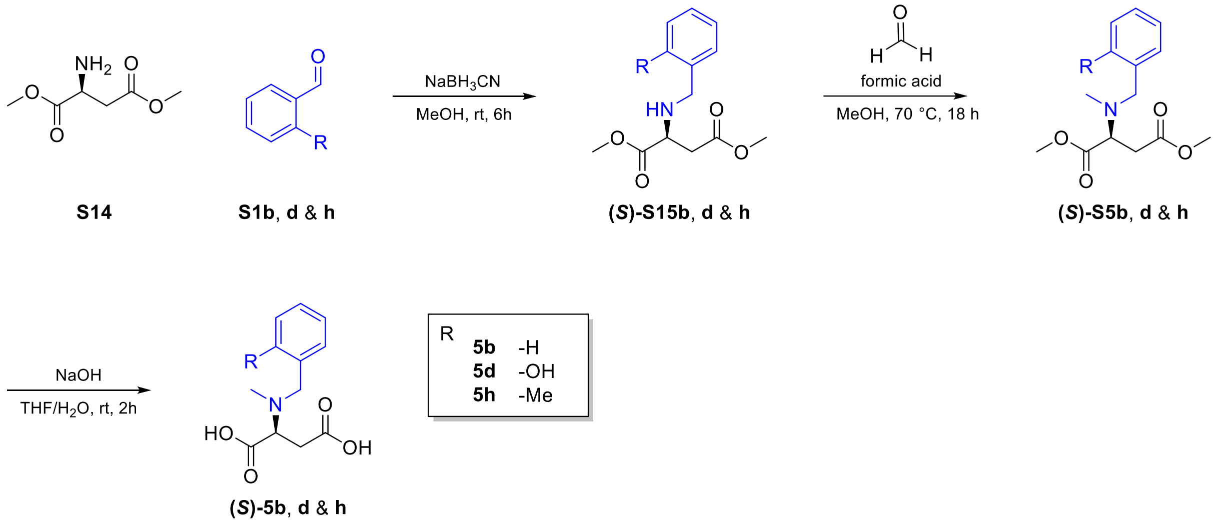

Step 1. To a vigorously stirred solution of **S14** (198 mg, 1.0 mmol) in absolute methanol (5.0 mL), a substituted 2-nitrobenzaldehyde (**S1b**, **d**, or **h**, 1.2 mmol) was added. The mixture was stirred for 30 minutes, after which $NaBH_3CN$ (67 mg, 1.1 mmol) was added portion-wise. The reaction mixture was stirred for 4-7 h. After completion of the reaction, the solvent was removed under reduced pressure. The residue was re-dissolved in a minimum volume of water and the pH was raised to ca. pH 9.0 using saturated aqueous Na_2CO_3, followed by extraction with ethyl acetate (3 x 15 mL). The combined organic portions were concentrated under reduced pressure, and purified with flash chromatography using 20% pentane/ethyl acetate to give **(*S*)-S15b**, **d** or **h**.

Step 2. To a stirred solution of *N*-benzyl-*N*-methyl-*L*-aspartate esters **(*S*)-S15b**, **d** or **h** (0.7-1.0 mmol) in methanol (5.0-7.0 mL) was added formaldehyde (37% solution, 100 eq.) and formic acid (100 eq.). The mixture was heated to 70 °C for 18 h. After completion of the reaction, volatiles were removed under reduced pressure. The aqueous layer was basified with $NaHCO_3$ to pH ~9.0 and extracted with ethyl acetate (3 x 10 mL). The organic layers were combined, filtered through hydrophobic filter paper and evaporated under pressure. The crude product was purified with flash chromatography using a mixture of acetone, heptane, and triethylamine (7:90:3) to give **(*S*)-S5b**, **d** or **h**.

Step 3. To a stirred solution of **(*S*)-S5b**, **d** or **h** (0.1-0.3 mmol) in THF (2.5 mL) was added 0.4 M NaOH (2.5 mL), and the reaction mixture was stirred at room temperature for 2 h. After completion of the reaction, volatiles were removed *in vacuo*. The aqueous layer was acidified with 1 N HCl and subjected to cation exchange chromatography, following the procedure described for its enzymatic counterpart.

The fractions that stained positive with ninhydrin or potassium permanganate were collected, concentrated under vacuum and lyophilized to provide the desired products **5b-m** as ammonium salts.

***N*-benzyl-*N*-methyl-*L*-aspartic acid ((*S*)-5b)**

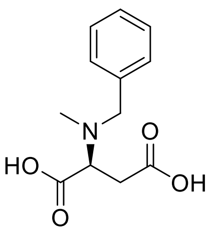

Yellowish solid, 51 mg (22% yield). **^{1}H NMR** (600 MHz, 0.1 M NaOD in Deuterium Oxide) δ 7.44 (d, *J* = 4.3 Hz, 4H), 7.42 – 7.37 (m, 1H), 3.87 (d, *J* = 12.7 Hz, 1H), 3.68 – 3.63 (m, 2H), 2.71 (dd, *J* = 15.6, 7.4 Hz, 1H), 2.55 (dd, *J* = 15.6, 6.9 Hz, 1H), 2.29 (s, 3H). **^{13}C NMR** (151 MHz, 0.1 M NaOD in D_2O) δ 179.83, 177.77, 137.11, 130.15, 128.56, 127.81, 66.98, 58.14, 38.11, 38.02. **HRMS** (ESI$^+$): calcd. For $C_{12}H_{16}NO_4$ [M+H]$^+$: 238.1074, found: 238.1061. **Chiral HPLC**: Nucleosil Chiral-1 column (5 µm, 250 x 4 mm, Macherey-Nagel), mobile phase: 0.5 mM aqueous $CuSO_4$, flow rate 1.0 mL/min, 60 °C, UV detection at 254 nm. T_R(*R*) = 5.2 min, T_R(*S*) = 6.1. The *ee* was determined to be 61% (*S*) by chiral HPLC analysis using a racemic standard.

***N*-(2-hydroxybenzyl)-*N*-methyl-*L*-aspartic acid ((*S*)-5d)**

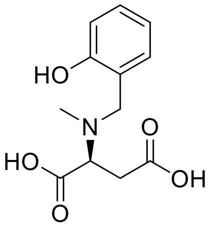

Yellowish solid, 30 mg (12% yield). **^{1}H NMR** (600 MHz, 0.1 M NaOD in Deuterium Oxide) δ 7.19 (d, *J* = 7.4 Hz, 1H), 7.04 (t, *J* = 7.6 Hz, 1H), 6.59 (d, *J* = 8.0 Hz, 1H), 6.54 (t, *J* = 7.3 Hz, 1H), 3.58 – 3.51 (m, 3H), 2.64 (dd, *J* = 15.3, 8.5 Hz, 1H), 2.50 (dd, *J* = 15.3, 5.7 Hz, 1H), 2.12 (s, 3H). **^{13}C NMR** (151 MHz, 0.1 M NaOD in D_2O) δ 180.69, 179.42, 165.41, 131.26, 128.51, 126.89, 119.18, 113.85, 67.71, 52.66, 38.40, 37.64. **HRMS** (ESI$^+$): calcd. for $C_{12}H_{16}NO_5$ [M+H]$^+$: 254.1023, found: 254.1022. **Chiral HPLC**: Chirex 3126 (*D*)-penicillamine column (250 x 4.6 mm, Phenomenex), phase A: 2.0 mM aqueous $CuSO_4$, phase B: isopropanol, 10% B (v/v), flow rate 1.0 mL/min, 50 °C, UV detection at 254 nm. T_R(*S*) = 13.9 min, T_R(*R*) = 14.5. The *ee* was determined to be 88% (*S*) by chiral HPLC analysis using a racemic standard.

***N*-methyl-*N*-(2-methylbenzyl)-*L*-aspartic acid (*L*-5h)**

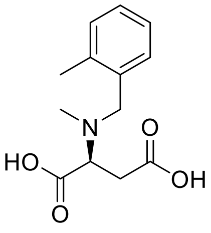

Yellowish solid, 61 mg (24% yield). **^{1}H NMR** (600 MHz, 0.1 M NaOD in Deuterium Oxide) δ 7.35 (d, *J* = 7.0 Hz, 1H), 7.29 – 7.21 (m, 3H), 3.71 (d, *J* = 12.9 Hz, 1H), 3.66 – 3.58 (m, 2H), 2.69 (dd, *J* = 15.2, 8.0 Hz, 1H), 2.53 (dd, *J* = 15.2, 6.6 Hz, 1H), 2.37 (s, 3H), 2.19 (s, 3H). **^{13}C NMR** (151 MHz, 0.1 M NaOD in D_2O) δ 180.39, 178.70, 138.06, 136.61, 130.91, 130.36, 127.52, 125.71, 68.05, 55.69, 38.65, 37.91, 18.61.

HRMS (ESI^+): calcd. for $C_{13}H_{18}NO_4$ $[M+H]^+$: 252.1230, found: 252.1220. **Chiral HPLC**: Nucleosil Chiral-1 column (5 μm, 250 x 4 mm, Macherey-Nagel), mobile phase: 0.5 mM aqueous $CuSO_4$, flow rate 1.0 mL/min, 60 °C, UV detection at 254 nm. T_R(*R*) = 5.6 min, T_R(*S*) = 6.2. The *ee* was determined to be 58% (*S*) by chiral HPLC analysis using a racemic standard.

III NMR spectra

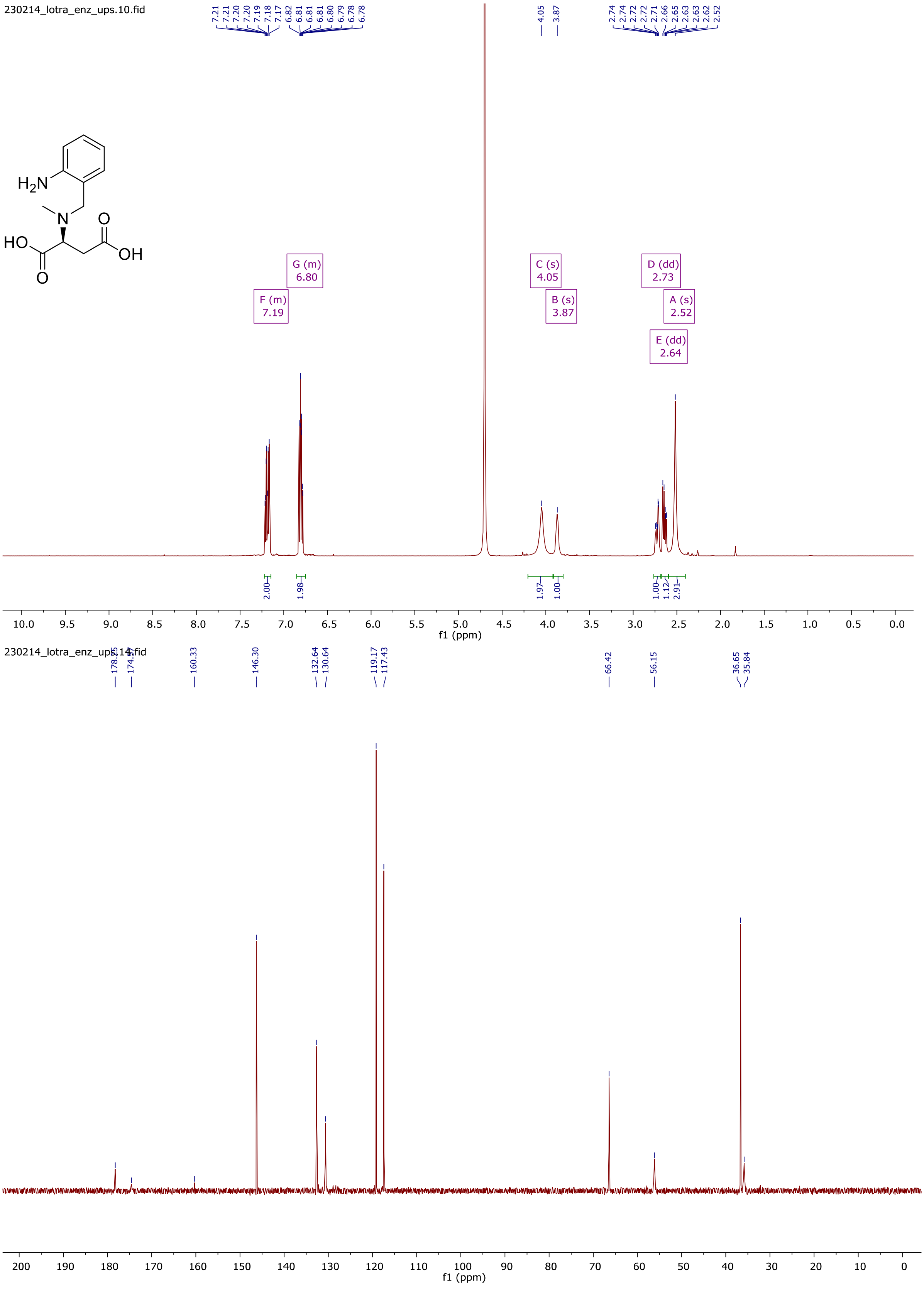

Figure S5. ^{1}H NMR (top) and ^{13}C NMR (bottom) of *N*-(2-aminobenzyl)-*N*-methyl-*L*-aspartic acid (***enz*-5a**) in D_2O

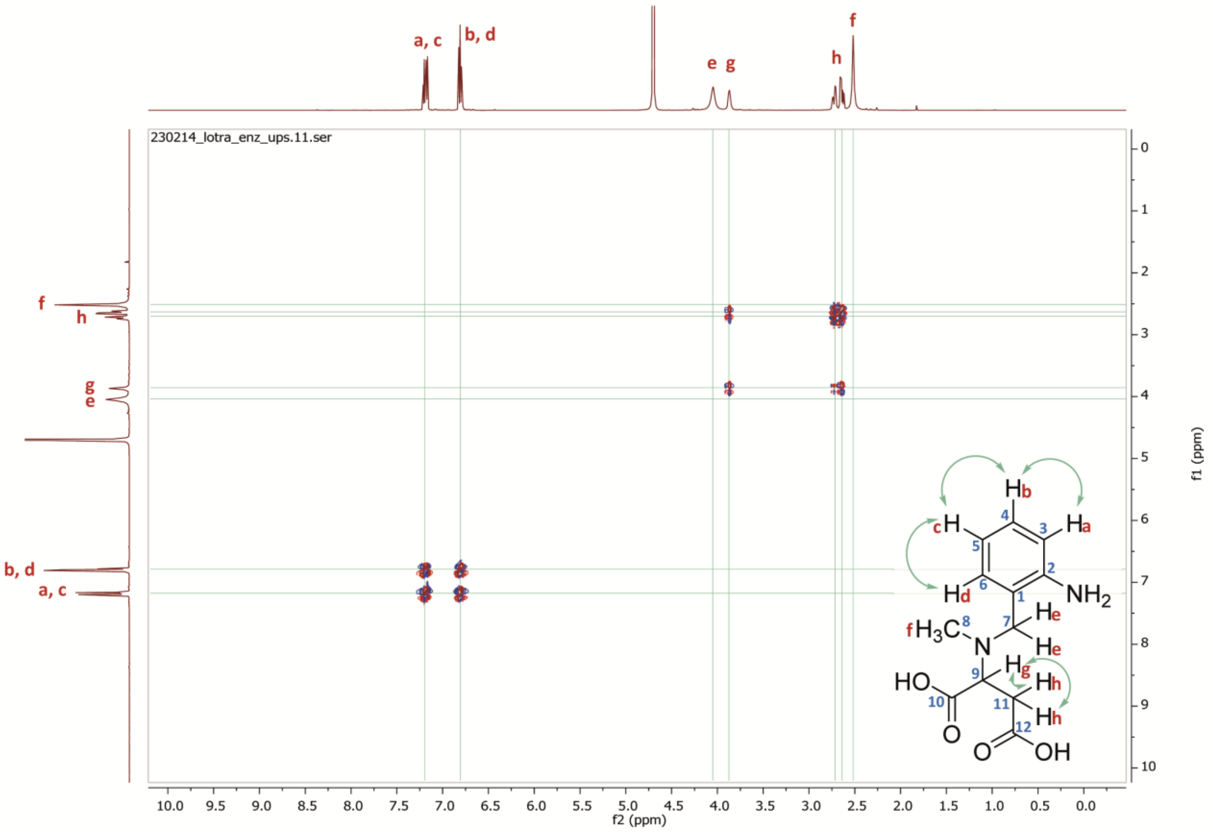

Figure S6. ¹H-¹H COSY NMR of *N*-(2-aminobenzyl)-*N*-methyl-*L*-aspartic acid (***enz*-5a**) in D_2O

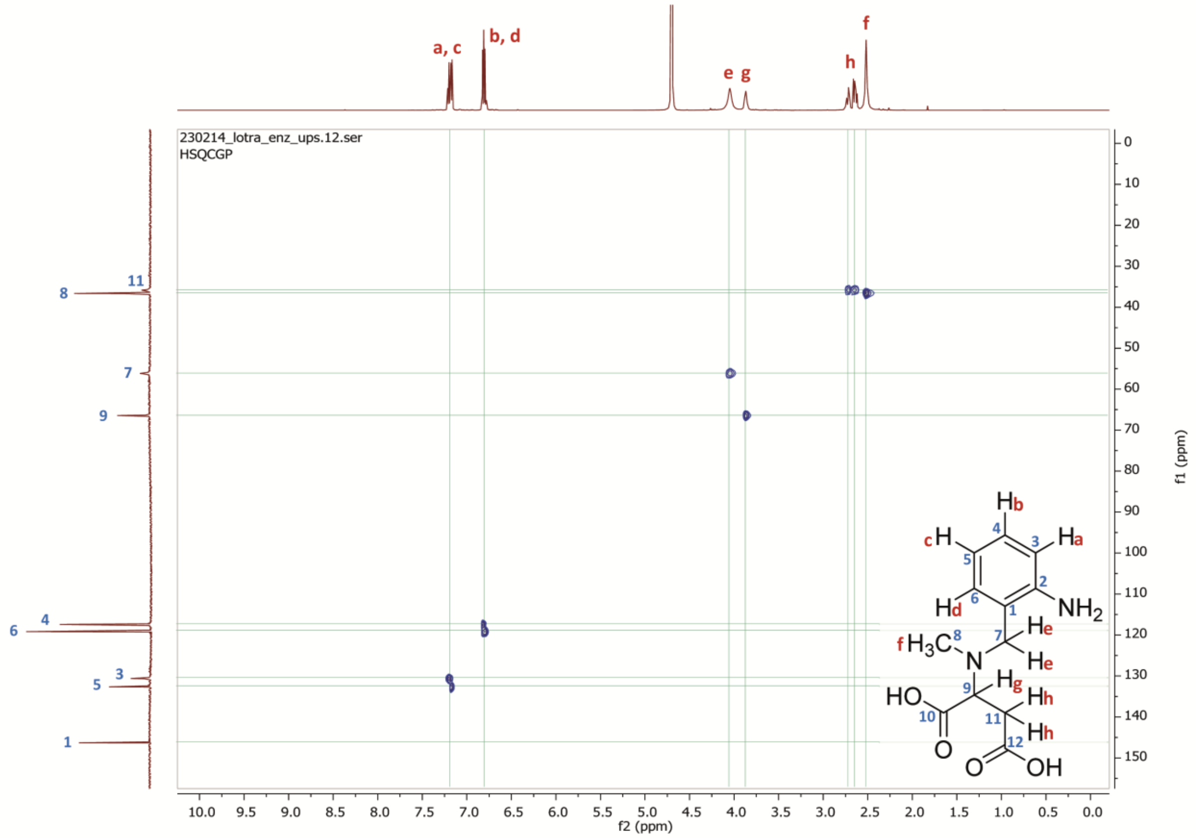

Figure S7. ¹H-¹³C HSQC NMR of *N*-(2-aminobenzyl)-*N*-methyl-*L*-aspartic acid (***enz*-5a**) in D_2O

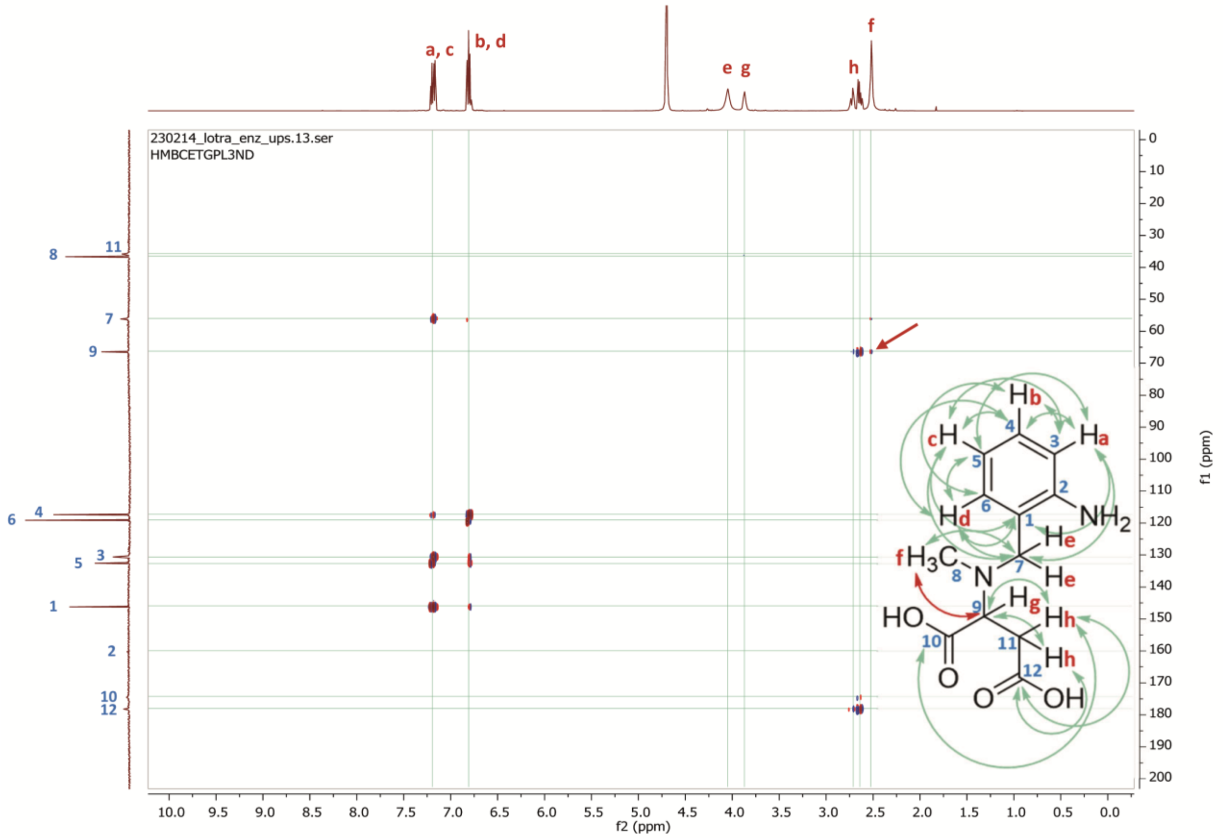

Figure S8. ^{1}H-^{13}C HMBC NMR of *N*-(2-aminobenzyl)-*N*-methyl-*L*-aspartic acid (***enz*-5a**) in D_2O

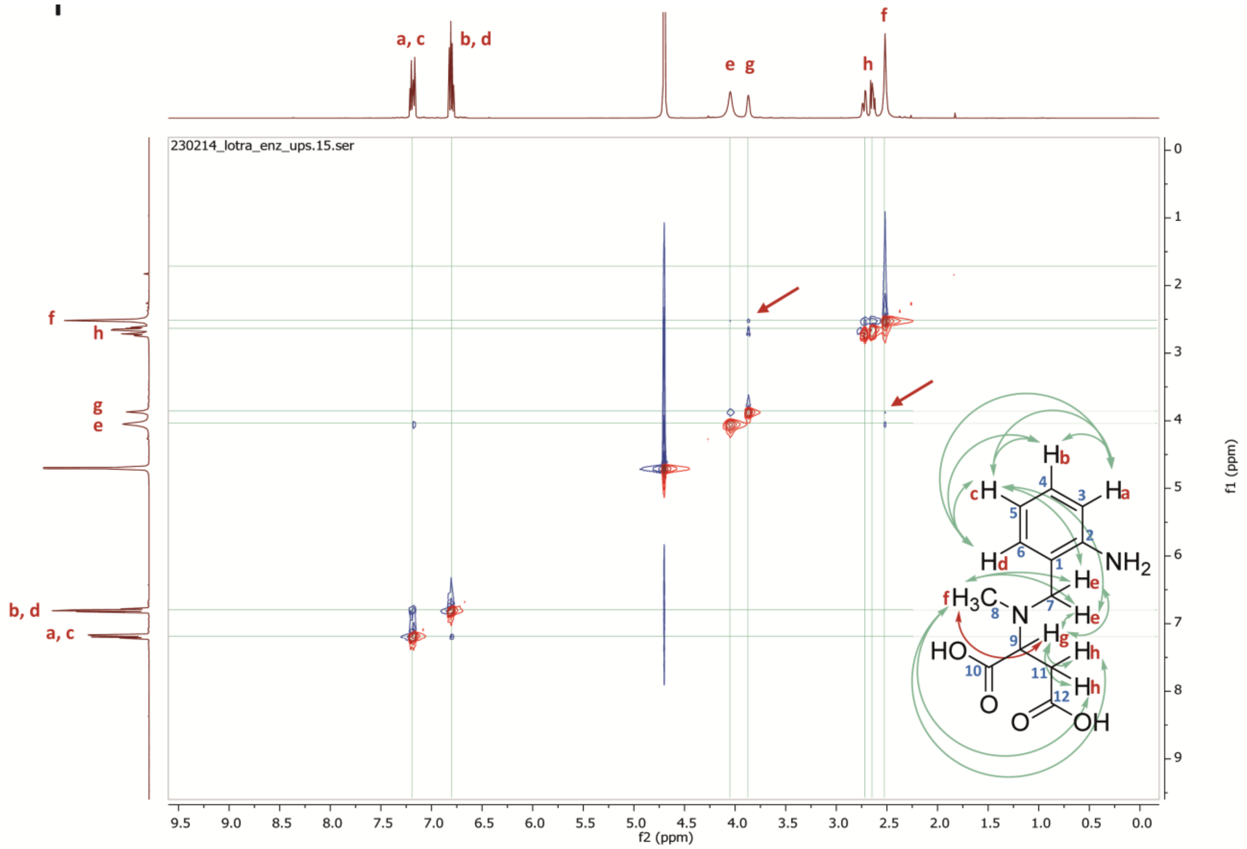

Figure S9. ^{1}H-^{1}H NEOSY NMR of *N*-(2-aminobenzyl)-*N*-methyl-*L*-aspartic acid (***enz*-5a**) in D_2O

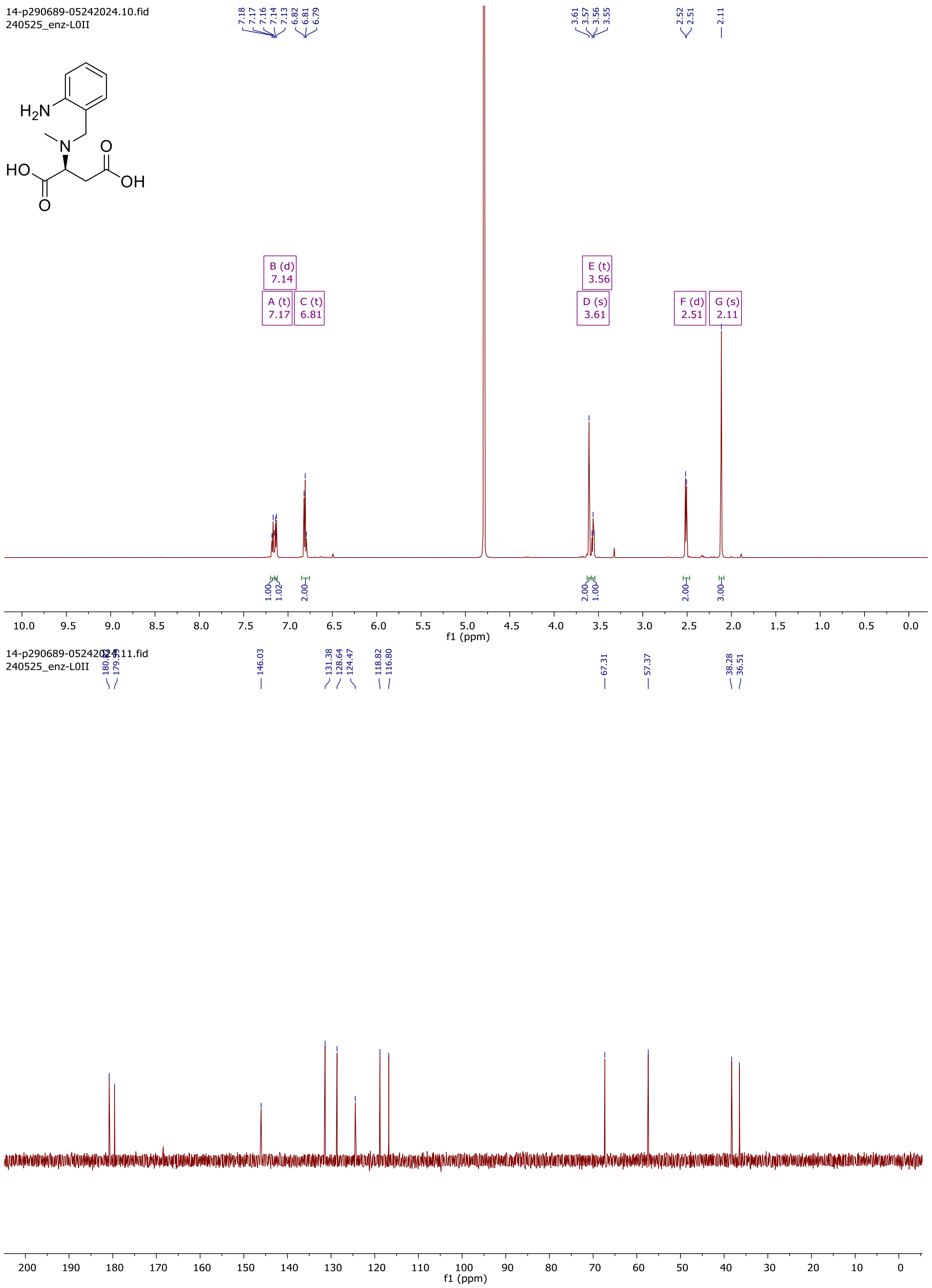

Figure S10. ^{1}H NMR (top) and ^{13}C NMR (bottom) of *N*-(2-aminobenzyl)-*N*-methyl-*L*-aspartic acid (***enz*-5a**) in 0.1 M NaOD/D_2O

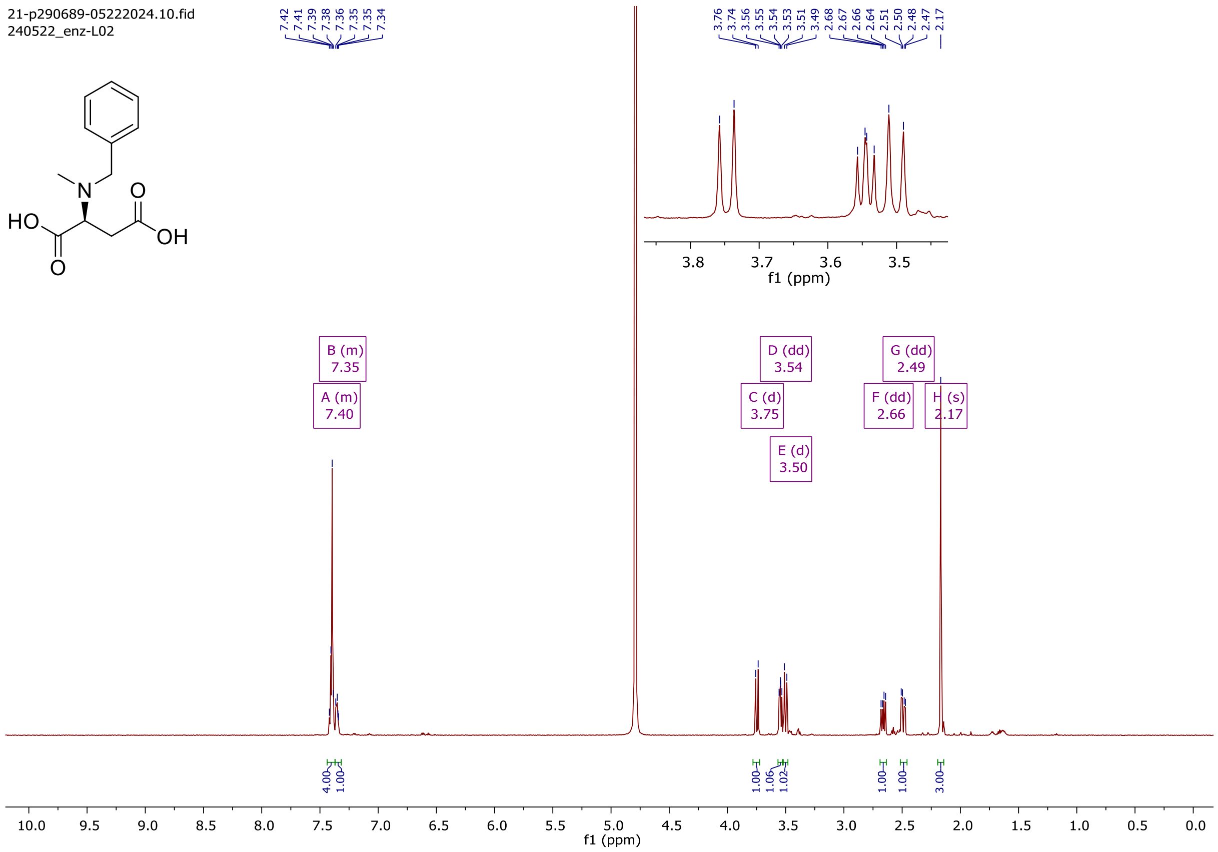

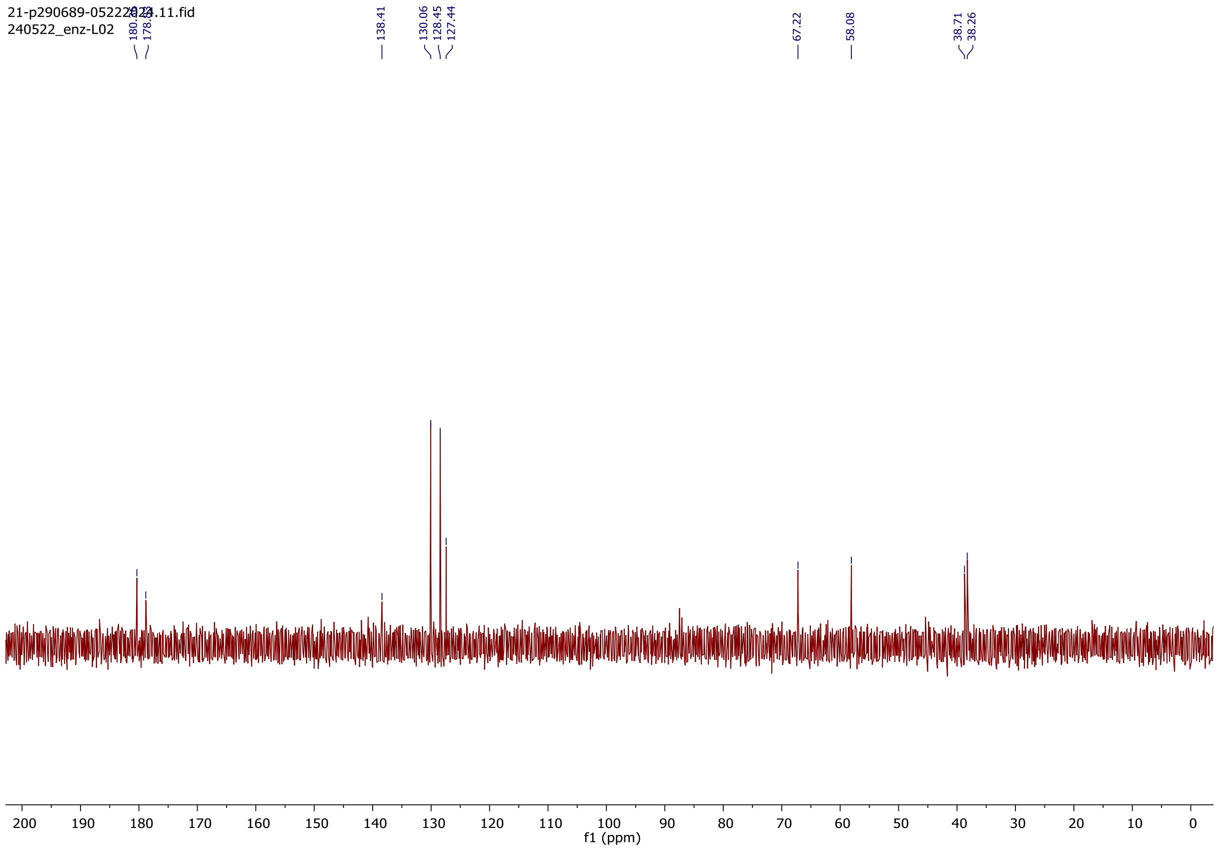

Figure S11. ^{1}H NMR (top) and ^{13}C NMR (bottom) of *N*-benzyl-*N*-methyl-*L*-aspartic acid (***enz*-5b**) in 0.1 M NaOD/D_2O

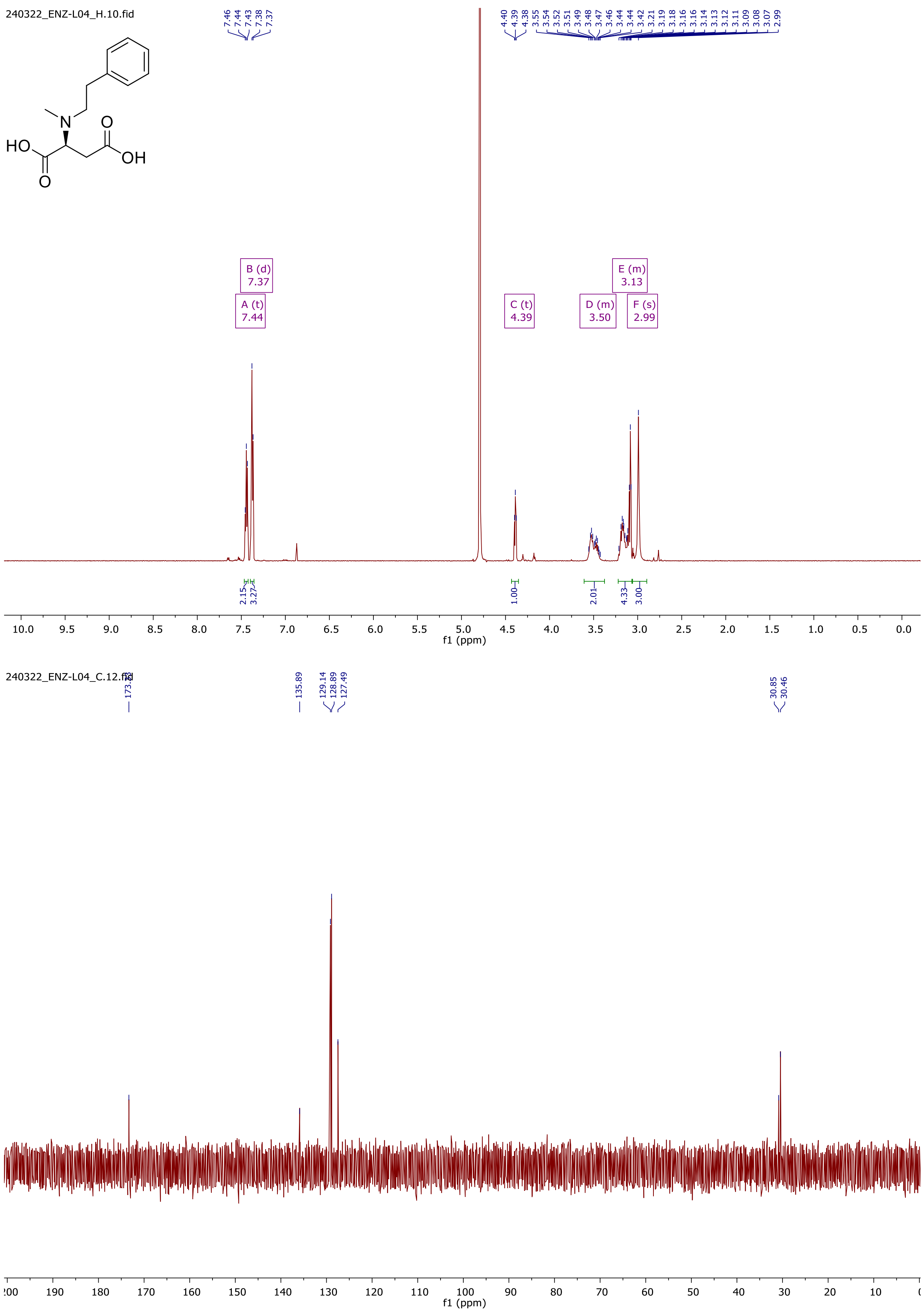

Figure S12. ^{1}H NMR (top) and ^{13}C NMR (bottom) of *N*-methyl-*N*-phenethyl-*L*-aspartic acid (***enz*-5c**) in 0.1 M DCl/D_2O

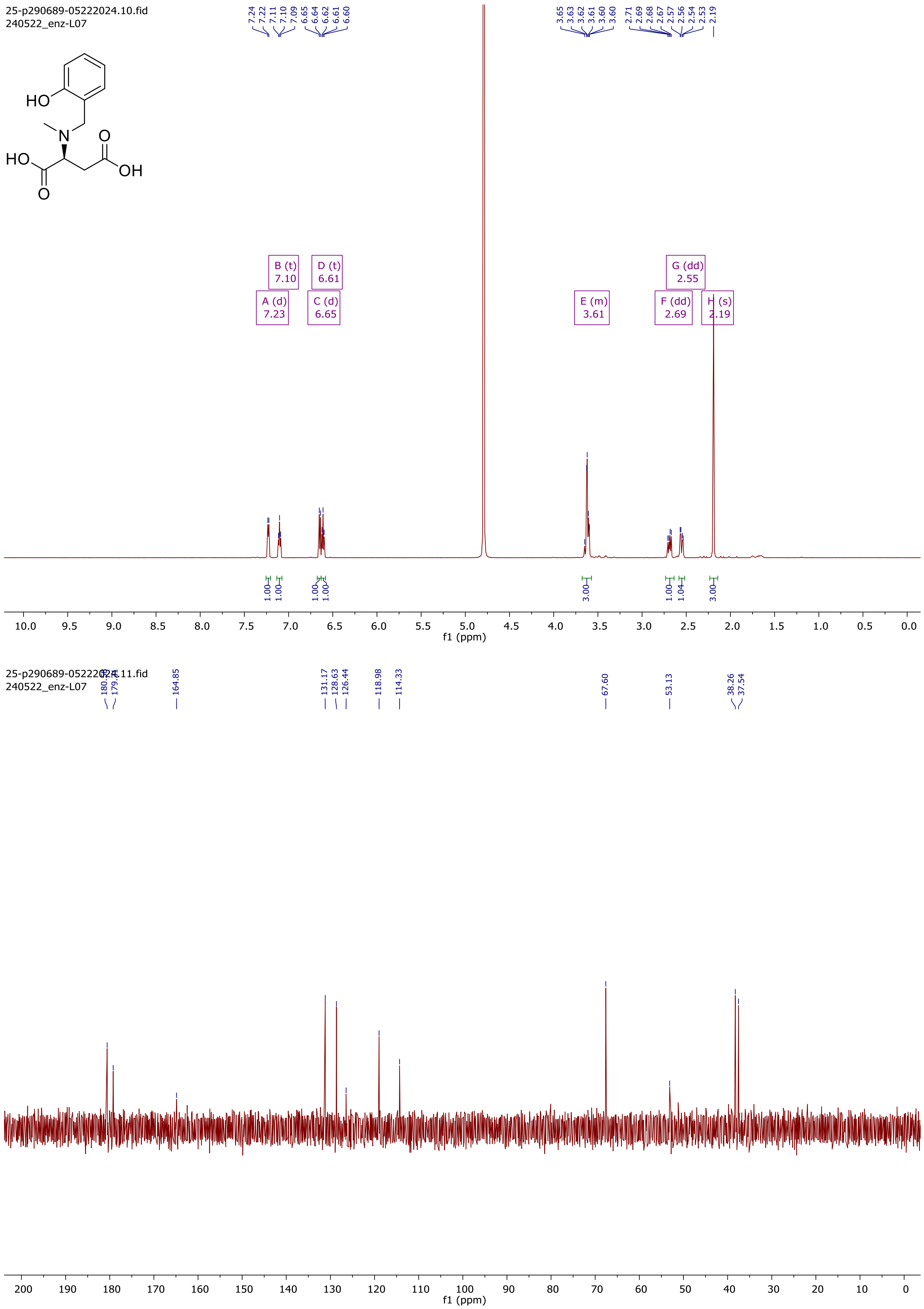

Figure S13. ^{1}H NMR (top) and ^{13}C NMR (bottom) of *N*-(2-hydroxybenzyl)-*N*-methyl-*L*-aspartic acid (***enz*-5d**) in 0.1 M NaOD/D_2O

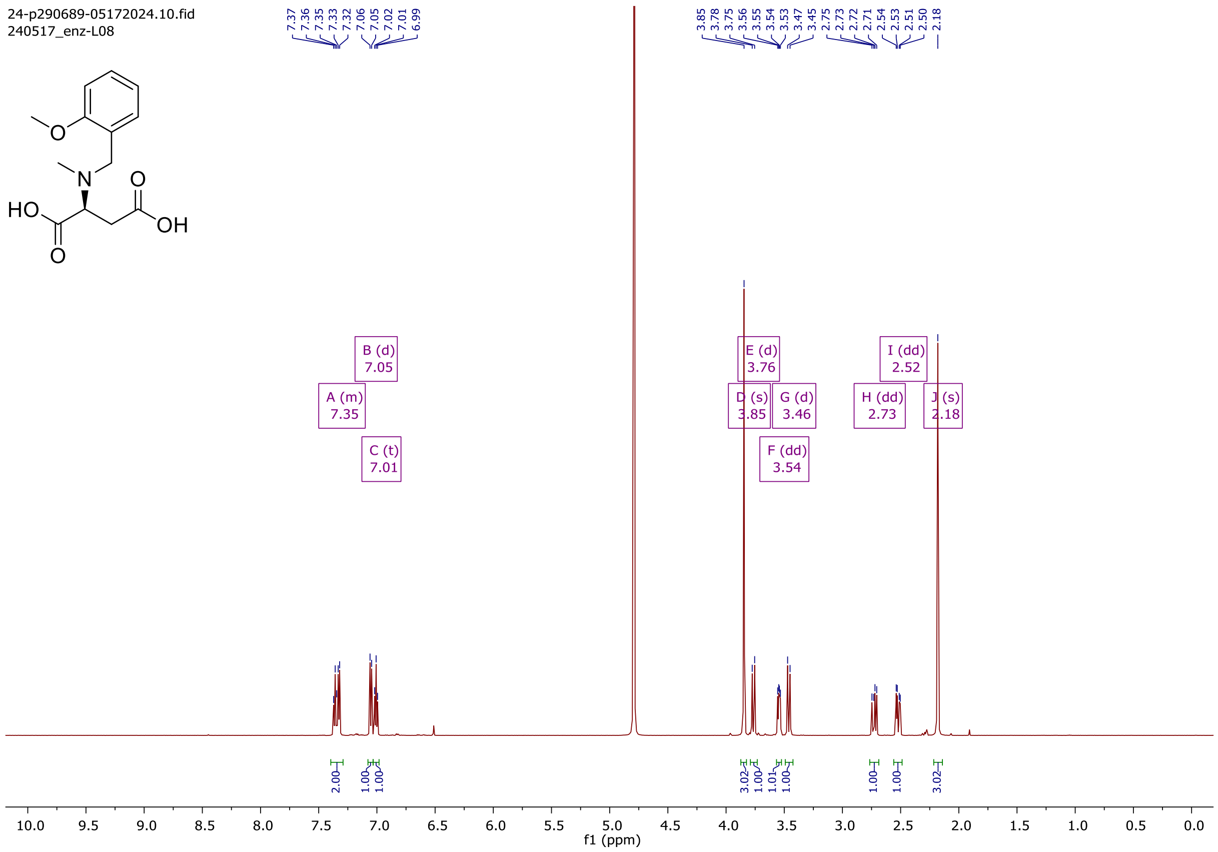

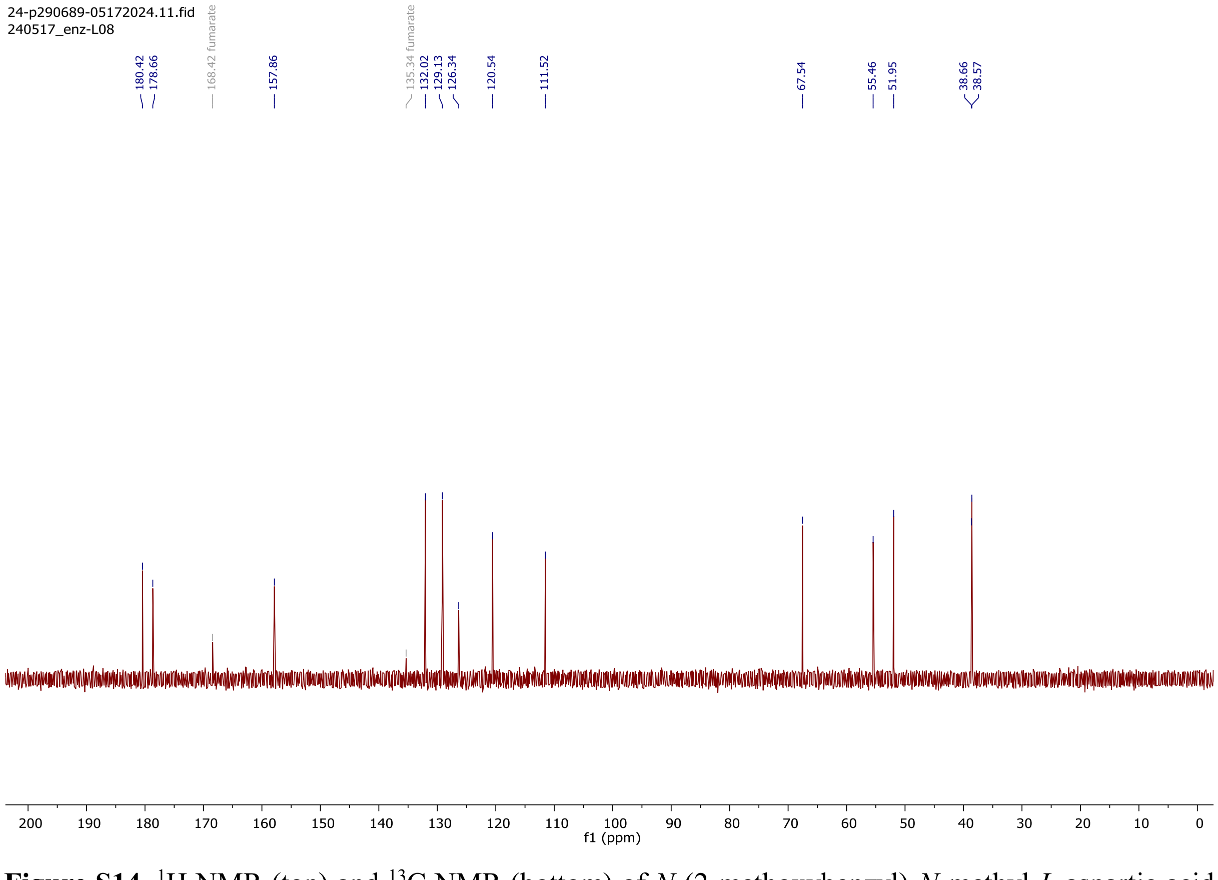

Figure S14. ^{1}H NMR (top) and ^{13}C NMR (bottom) of *N*-(2-methoxybenzyl)-*N*-methyl-*L*-aspartic acid (***enz*-5e**) in 0.1 M NaOD/D_2O, contains traces of fumaric acid

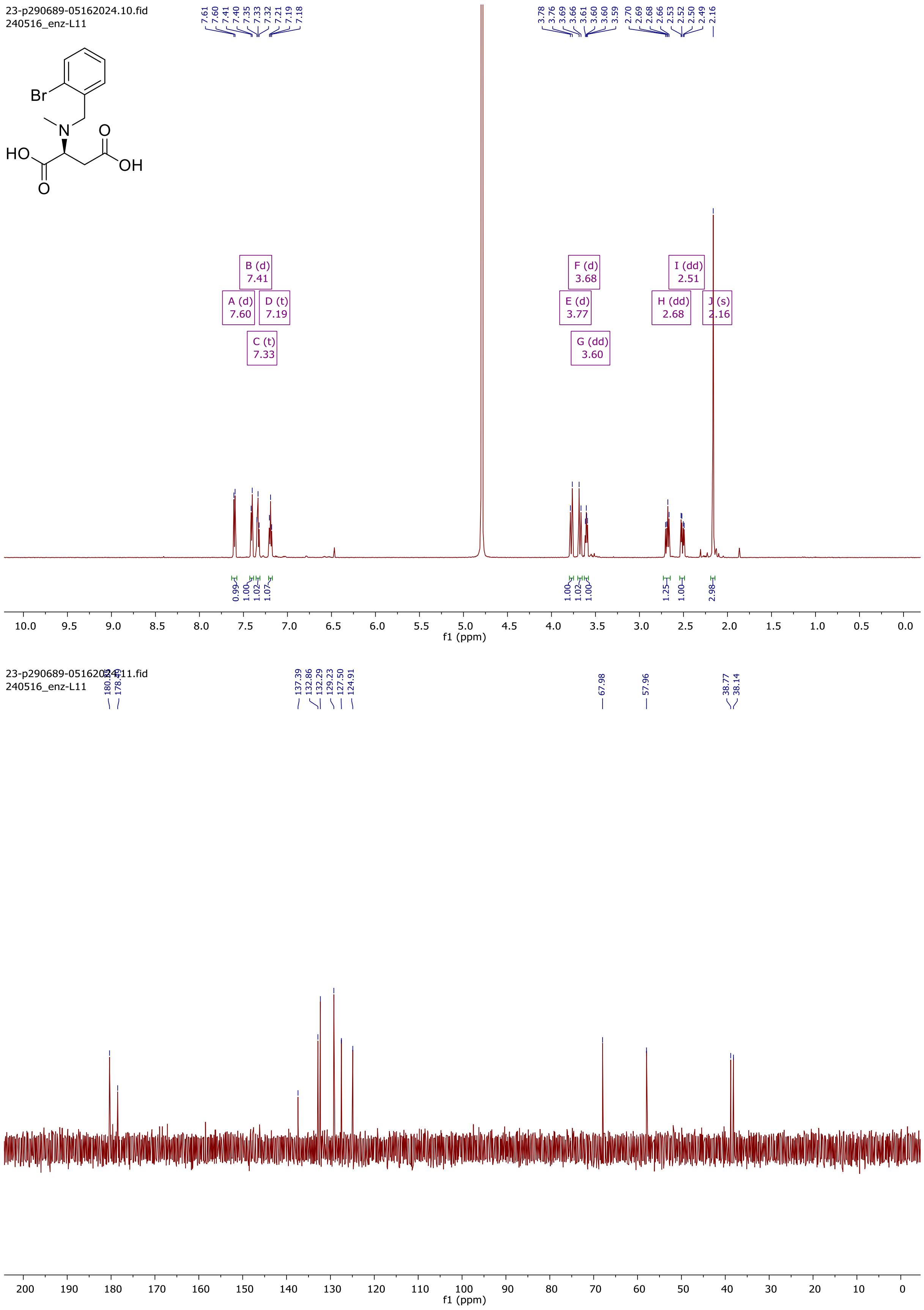

Figure S15. ^{1}H NMR (top) and ^{13}C NMR (bottom) of *N*-(2-bromobenzyl)-*N*-methyl-*L*-aspartic acid (***enz*-5f**) in 0.1 M NaOD/D_2O

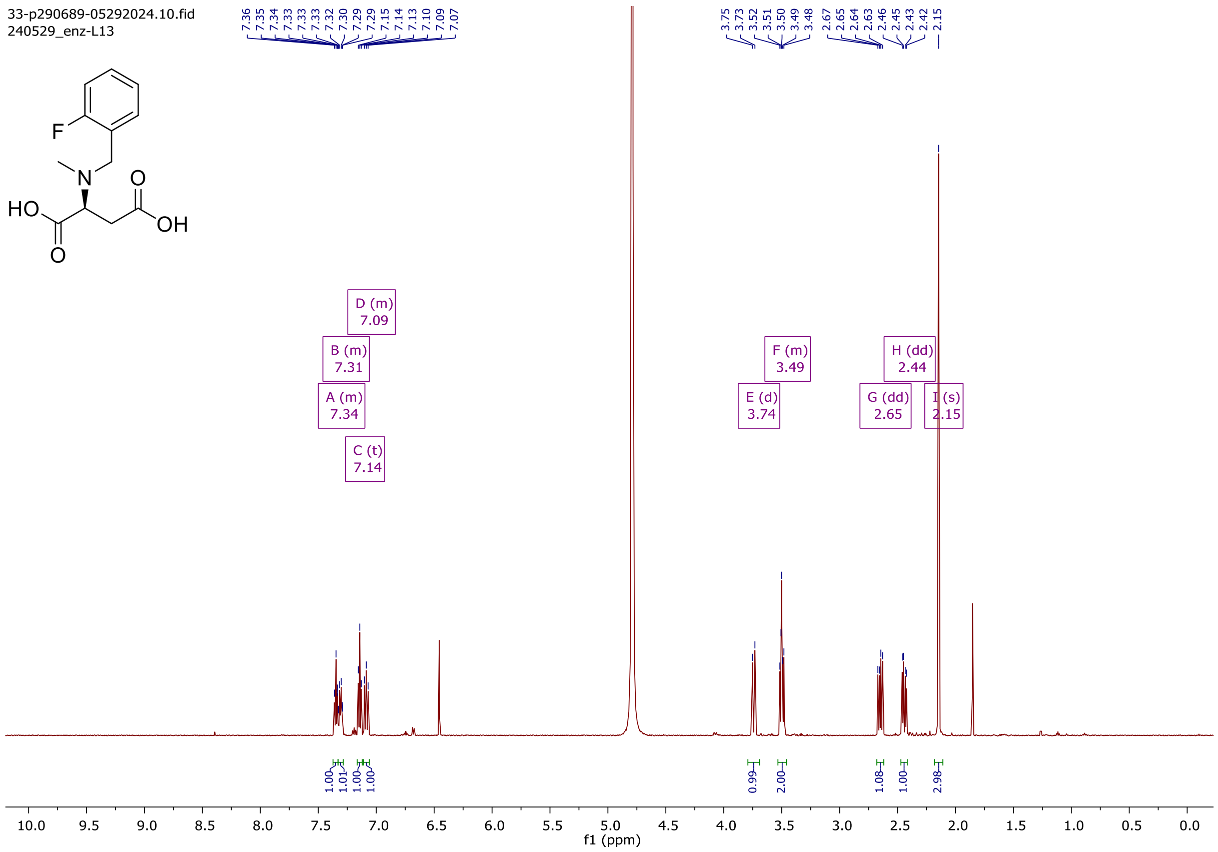

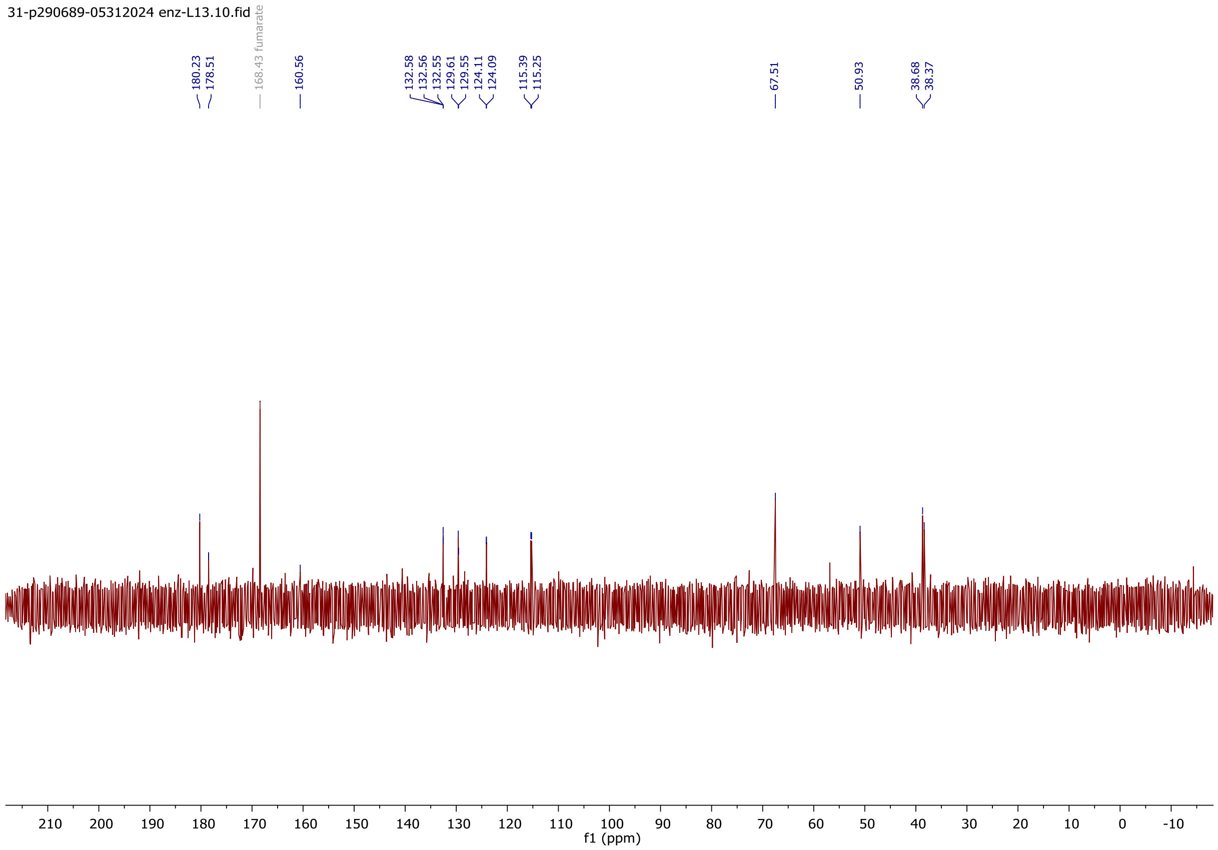

Figure S16. ^{1}H NMR (top) and ^{13}C NMR (bottom) of *N*-(2-fluorobenzyl)-*N*-methyl-*L*-aspartic acid (***enz*-5g**) in 0.1 M NaOD/D_2O, contains traces of fumaric acid

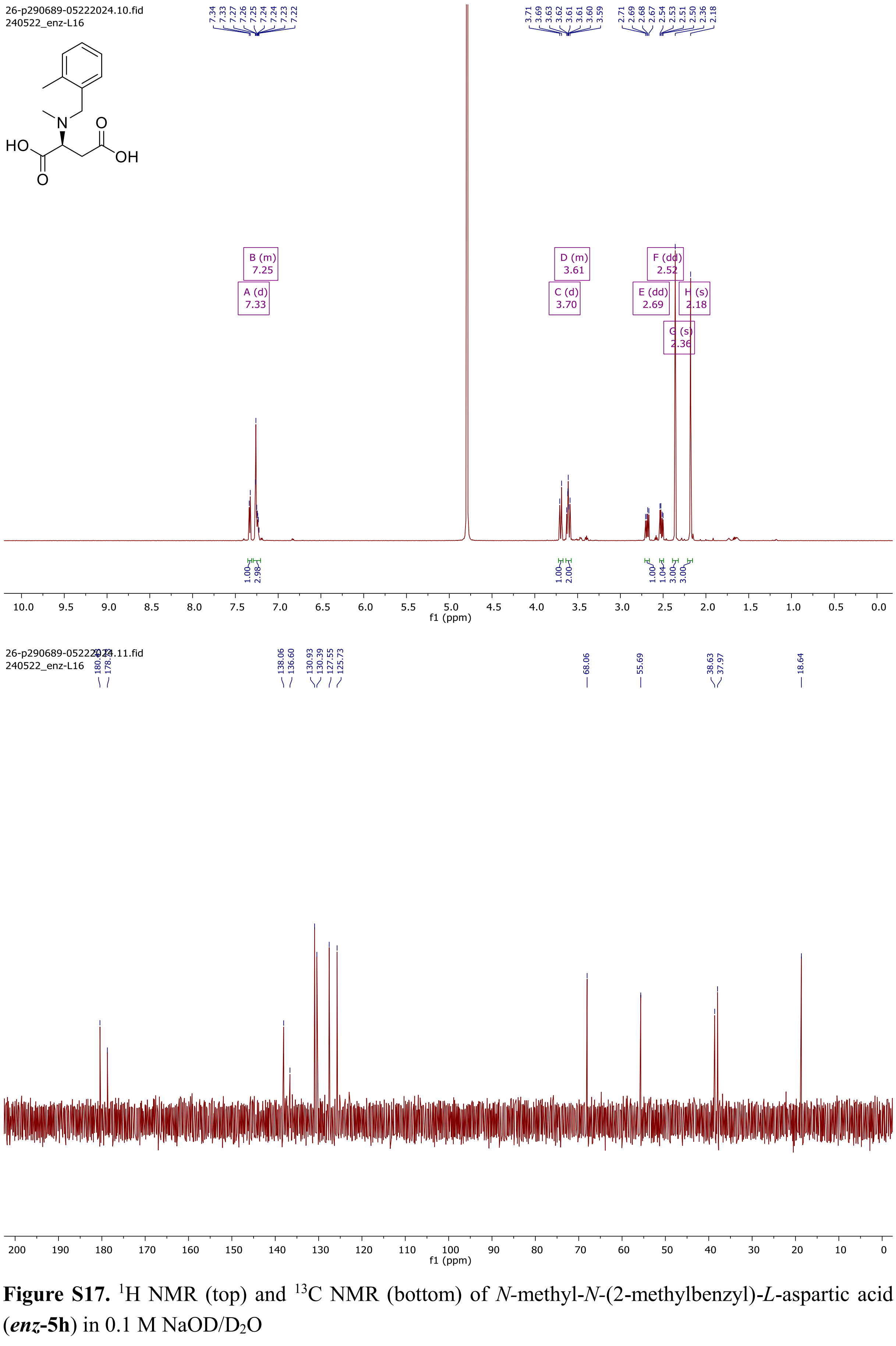

Figure S17. ^{1}H NMR (top) and ^{13}C NMR (bottom) of *N*-methyl-*N*-(2-methylbenzyl)-*L*-aspartic acid (***enz*-5h**) in 0.1 M NaOD/D_2O

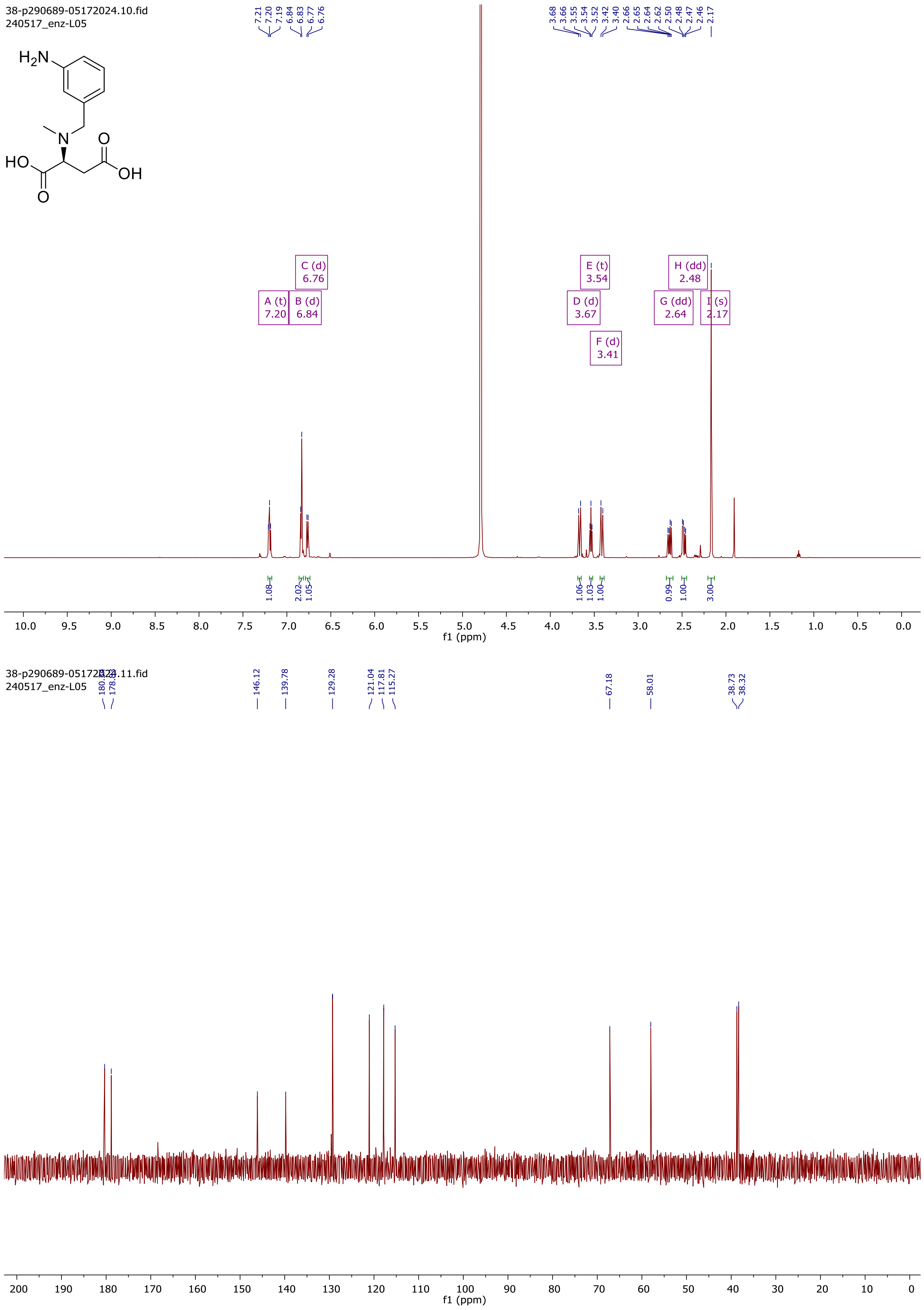

Figure S18. ^{1}H NMR (top) and ^{13}C NMR (bottom) of *N*-(3-aminobenzyl)-*N*-methyl-*L*-aspartic acid (***enz*-5i**) in 0.1 M NaOD/D_2O

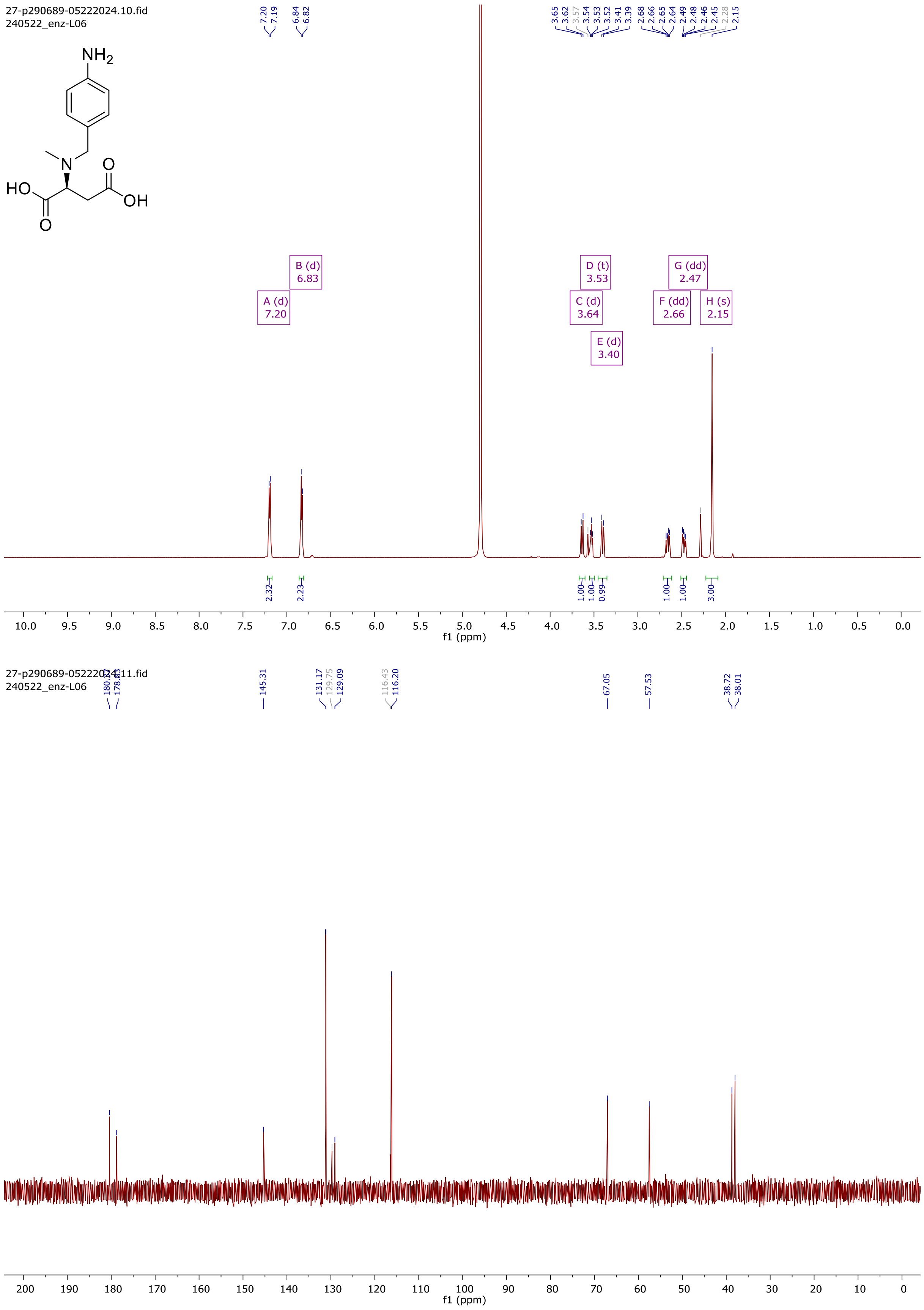

Figure S19. ^{1}H NMR (top) and ^{13}C NMR (bottom) of *N*-(4-aminobenzyl)-*N*-methyl-*L*-aspartic acid (***enz*-5j**) in 0.1 M NaOD/D_2O, contains traces of substrate **2j**

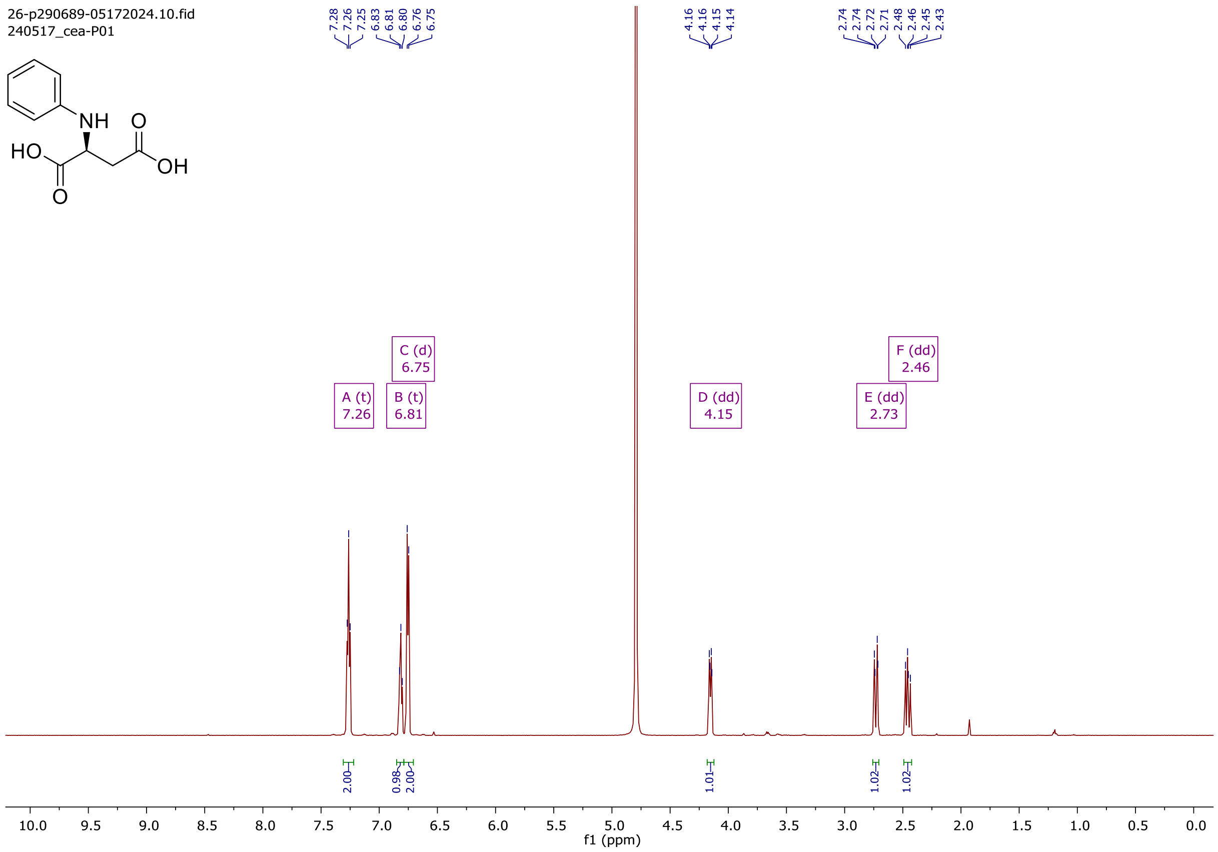

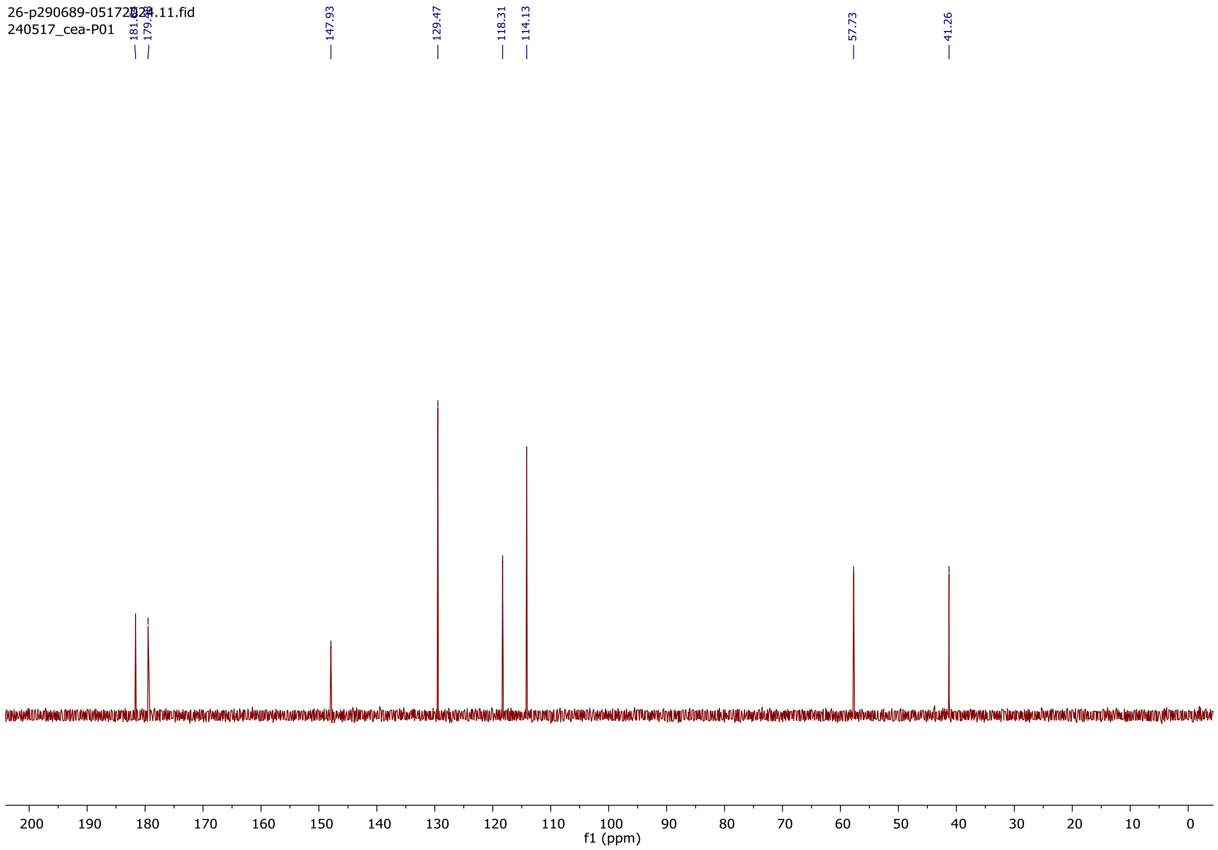

Figure S20. ^{1}H NMR (top) and ^{13}C NMR (bottom) of phenyl-*L*-aspartic acid (***enz*-3b** obtained with EDDS lyase CEA) in 0.1 M NaOD/D_2O

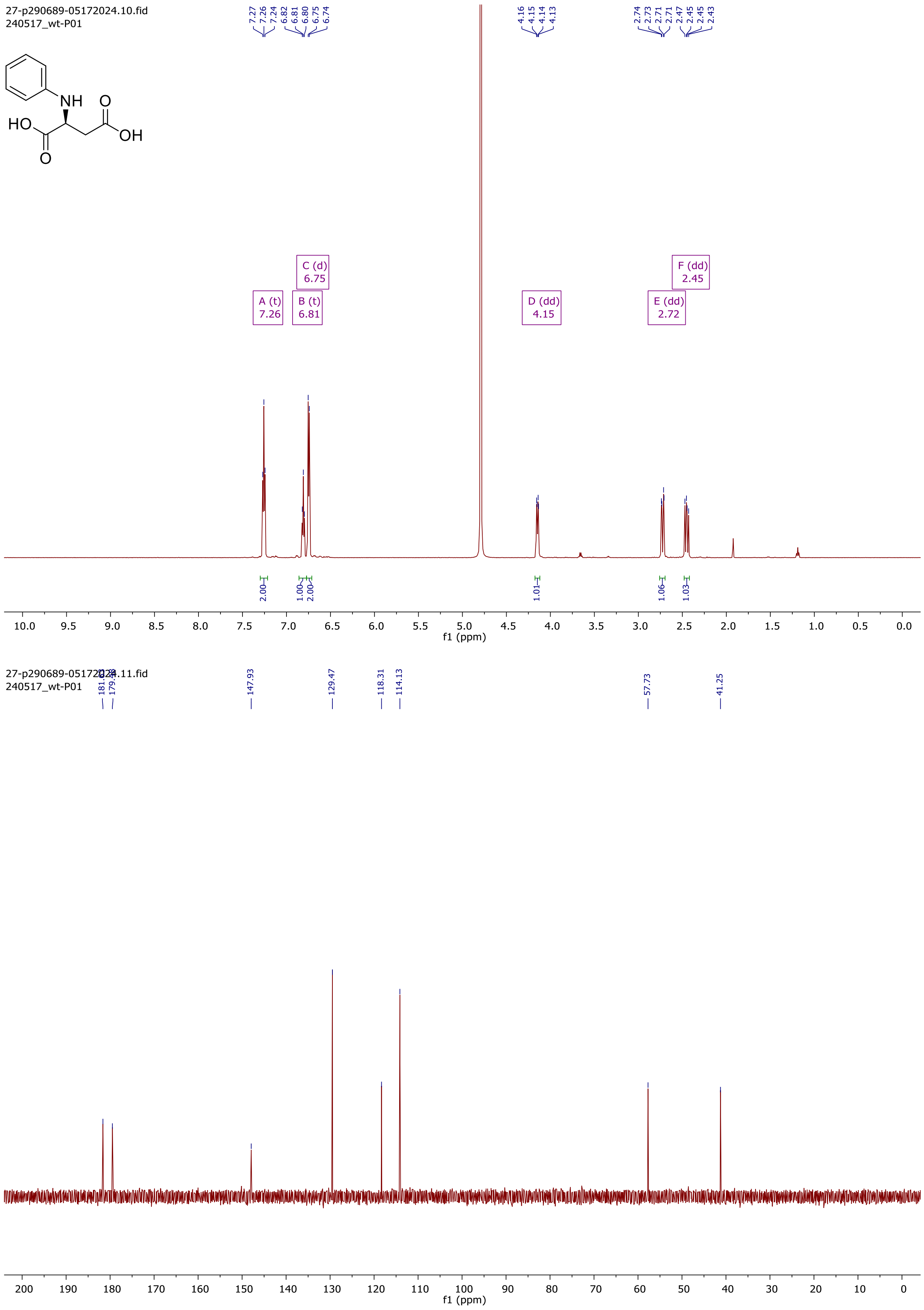

Figure S21. ^{1}H NMR (top) and ^{13}C NMR (bottom) of phenyl-*L*-aspartic acid (***enz*-3b** obtained with EDDS lyase WT) in 0.1 M NaOD/D_2O

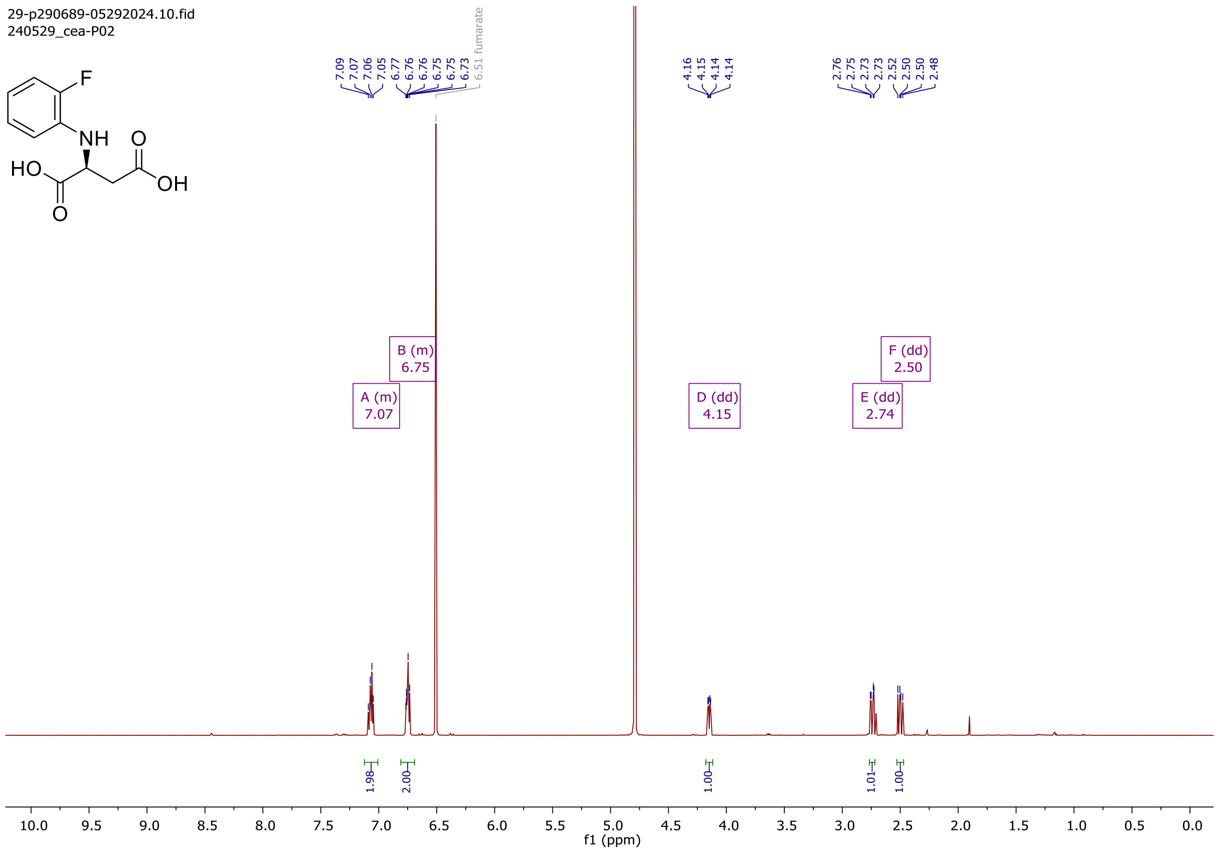

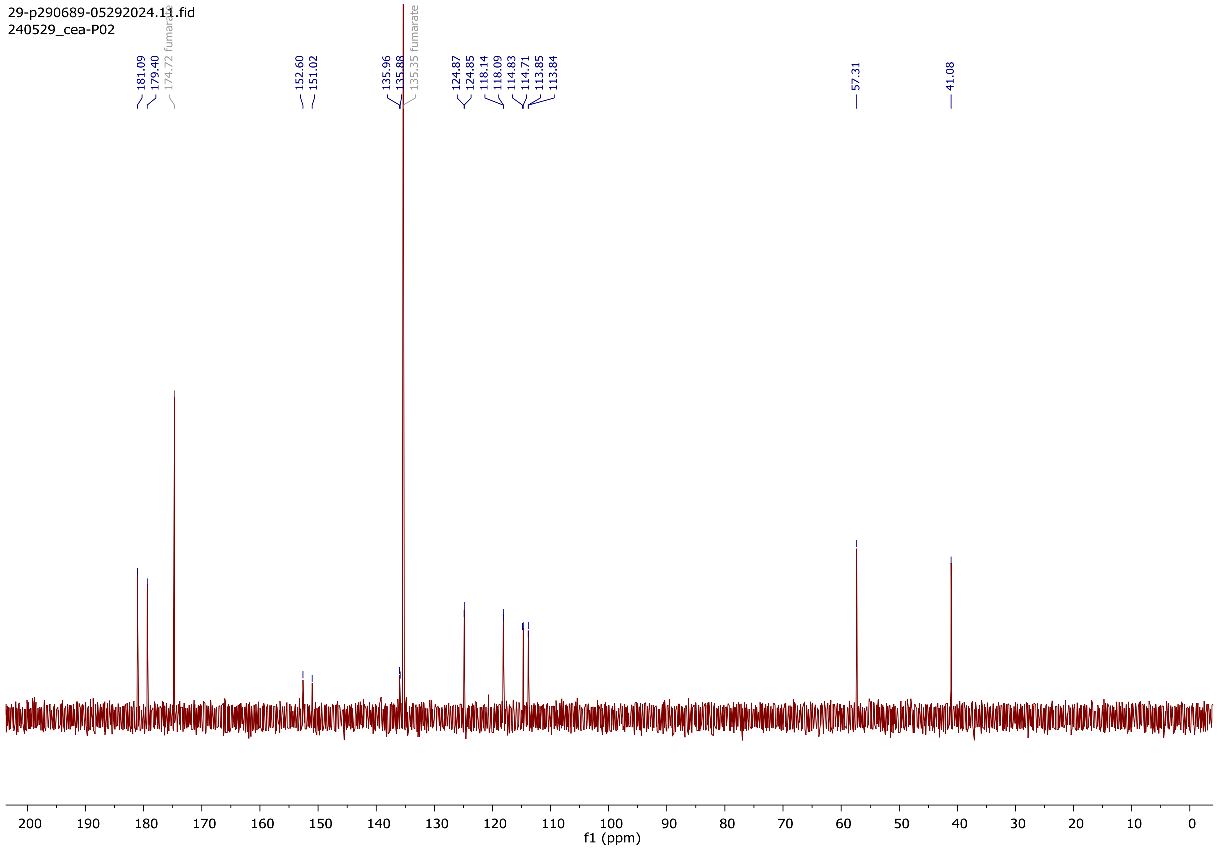

Figure S22. ^{1}H NMR (top) and ^{13}C NMR (bottom) of (2-fluorophenyl)-*L*-aspartic acid (***enz*-3c**) in 0.1 M NaOD/D_2O

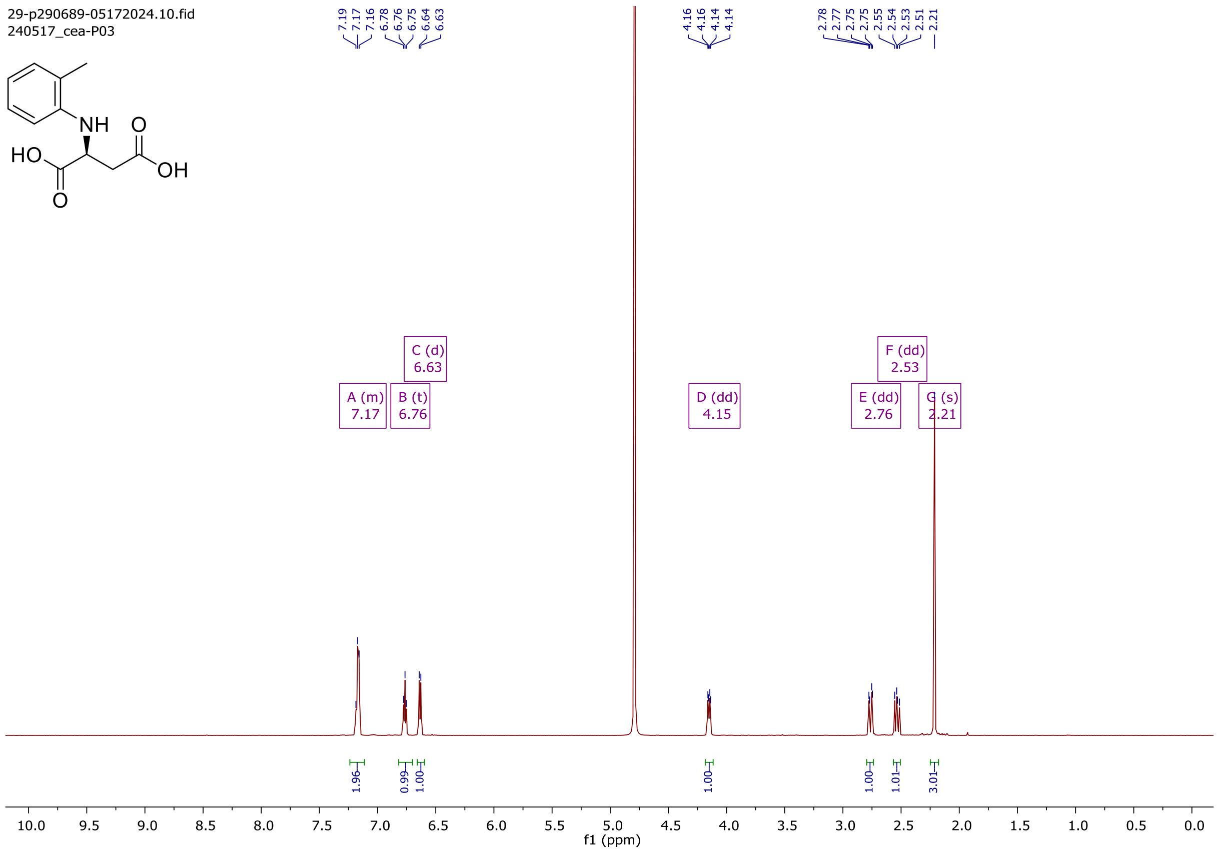

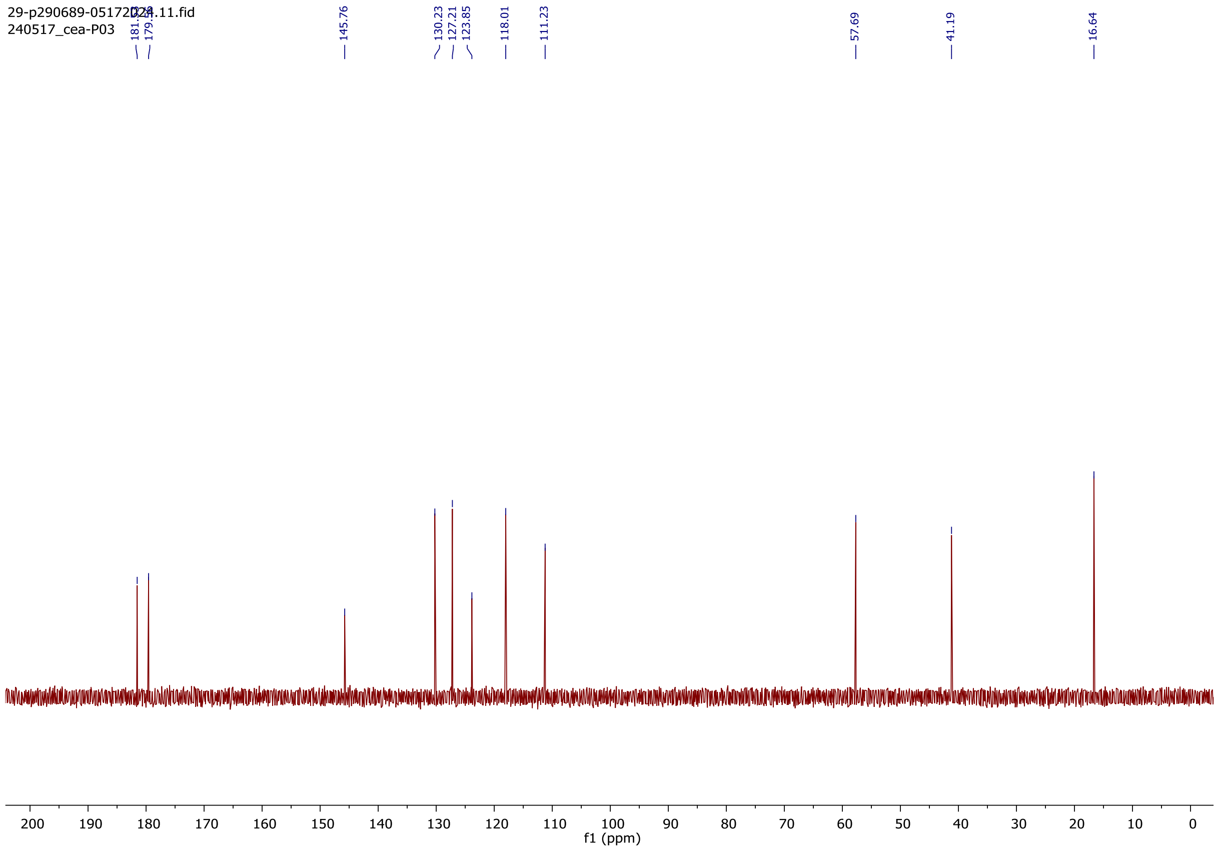

Figure S23. ^{1}H NMR (top) and ^{13}C NMR (bottom) of *o*-tolyl-*L*-aspartic acid (***enz*-3d**) in 0.1 M NaOD/D_2O

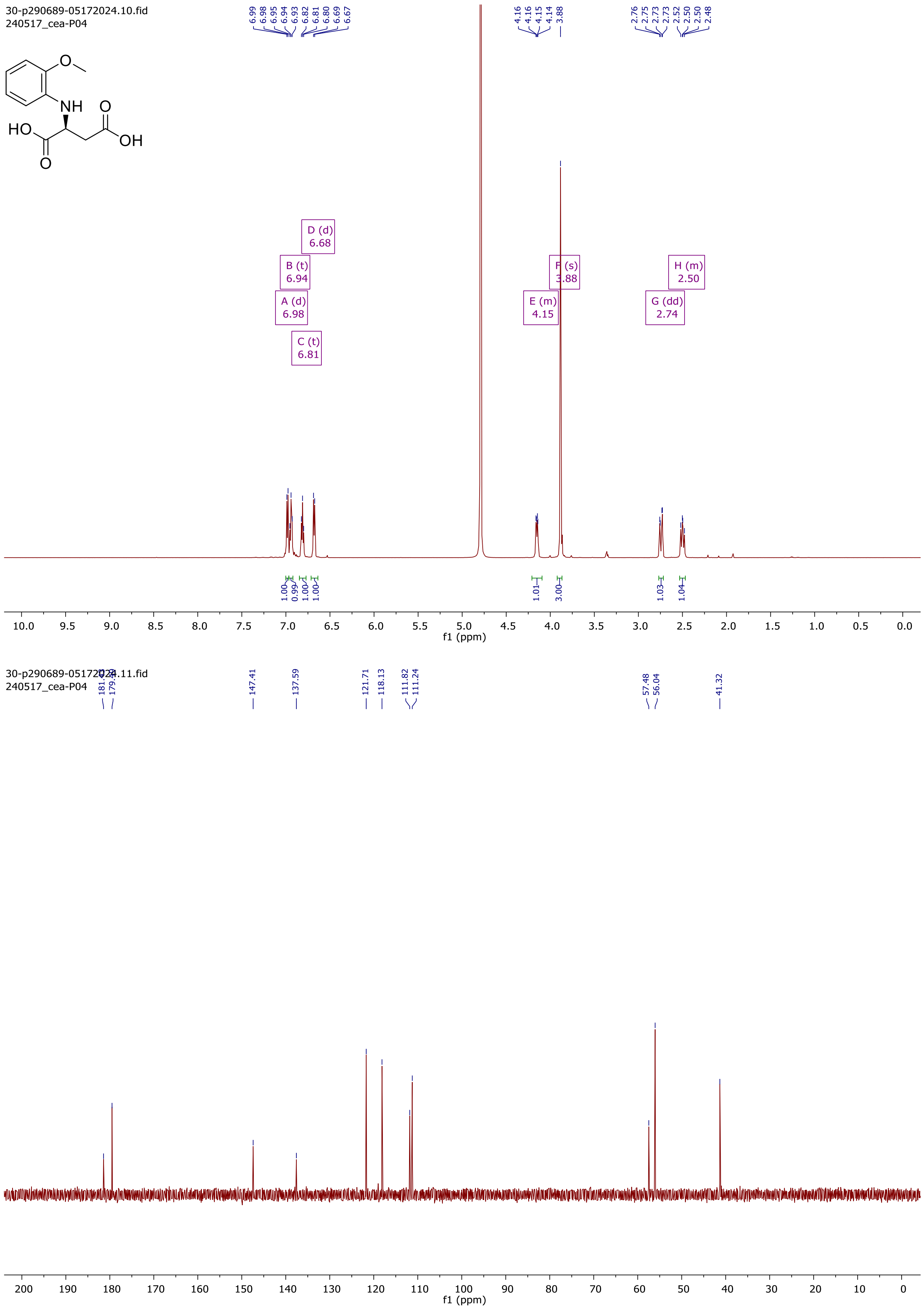

Figure S24. ^{1}H NMR (top) and ^{13}C NMR (bottom) of (2-methoxyphenyl)-*L*-aspartic acid (***enz*-3e**) in 0.1 M NaOD/D_2O

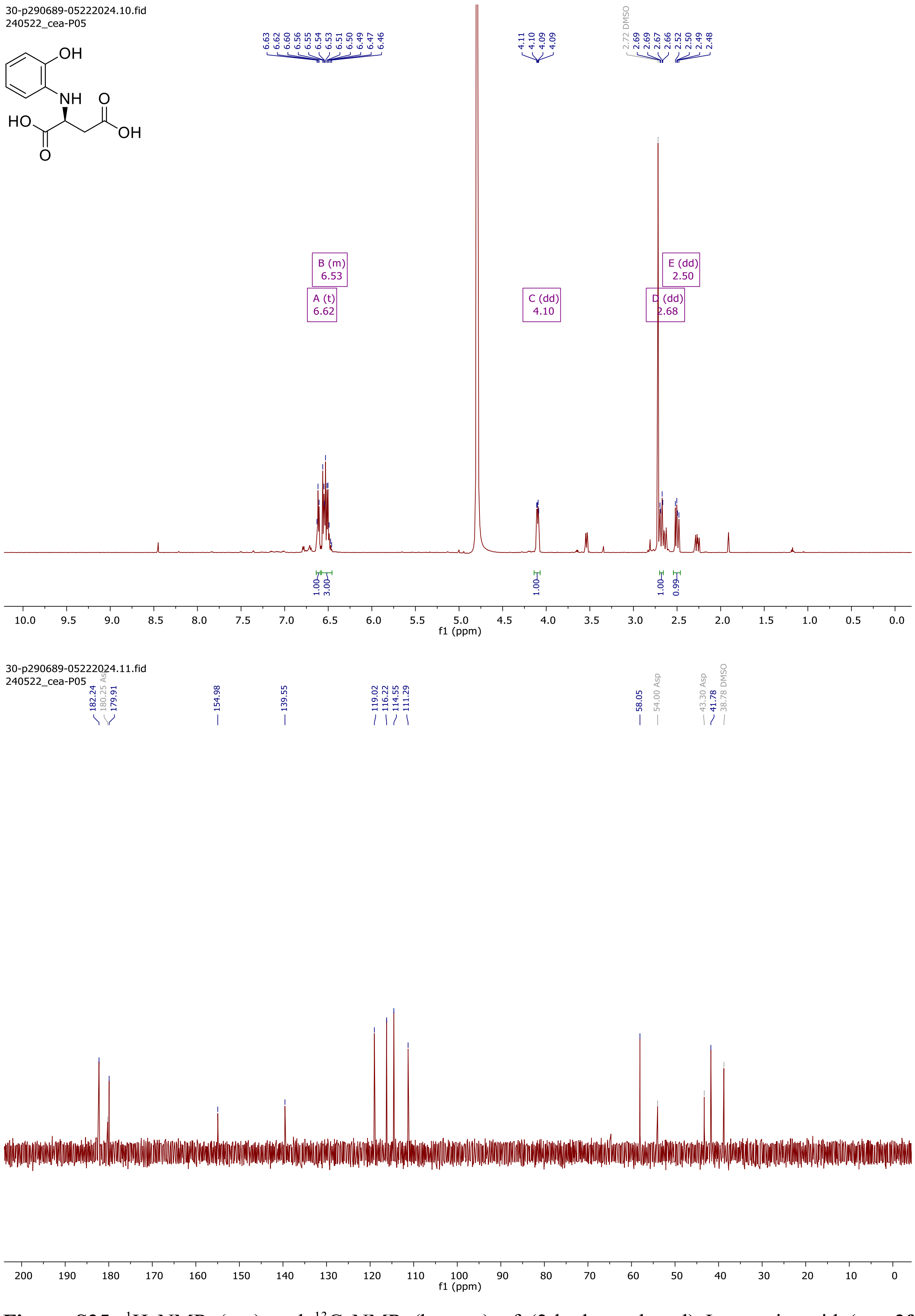

Figure S25. ^{1}H NMR (top) and ^{13}C NMR (bottom) of (2-hydroxyphenyl)-*L*-aspartic acid (***enz*-3f** obtained with EDDS lyase CEA) in 0.1 M NaOD/D_2O, contains traces of DMSO and aspartic acid

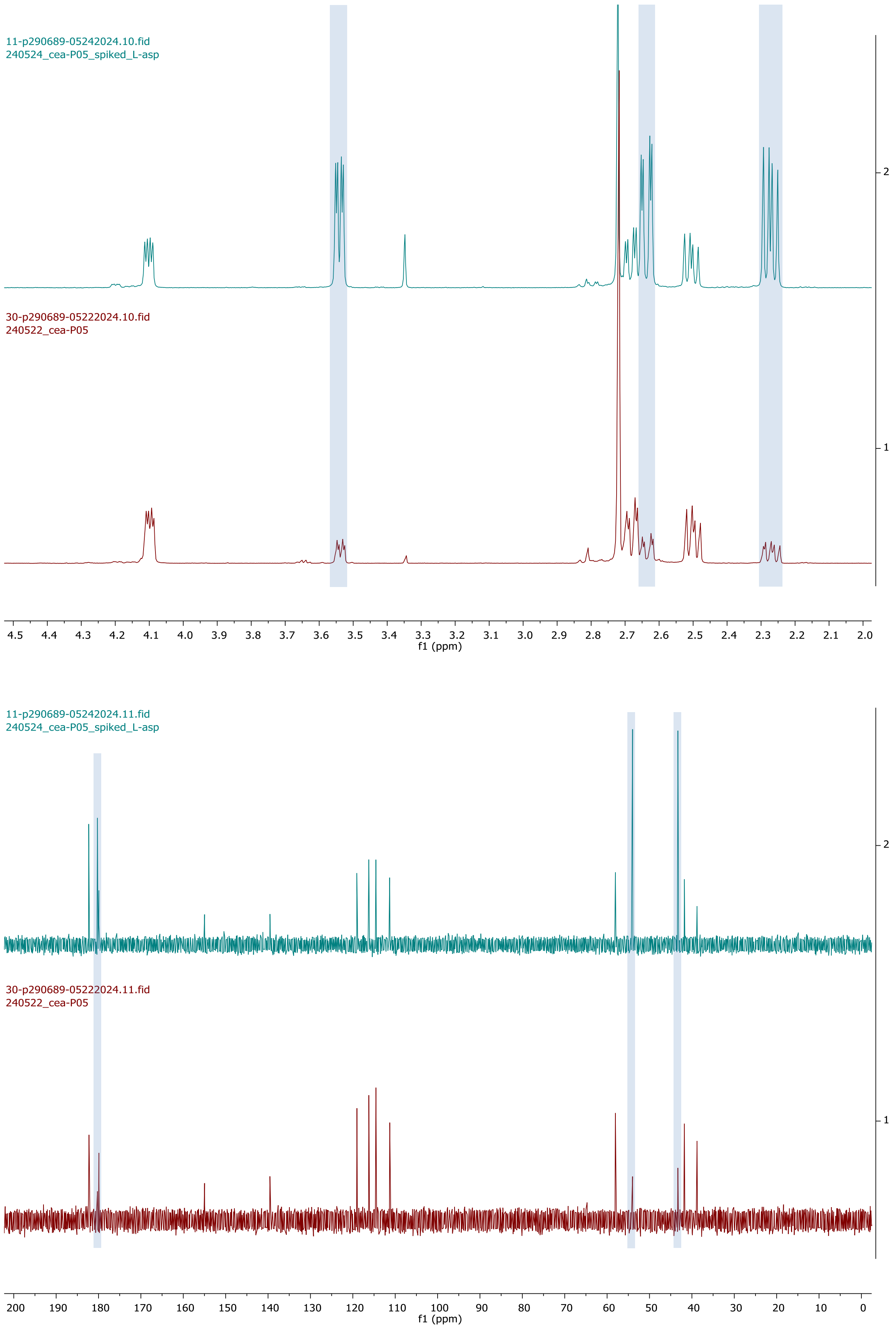

Figure S26. Comparison of ^{1}H NMR (top) and ^{13}C NMR (bottom) of ***enz*-3f** as obtained with EDDS lyase CEA (in green), and spiked with *L*-aspartic acid (in red).

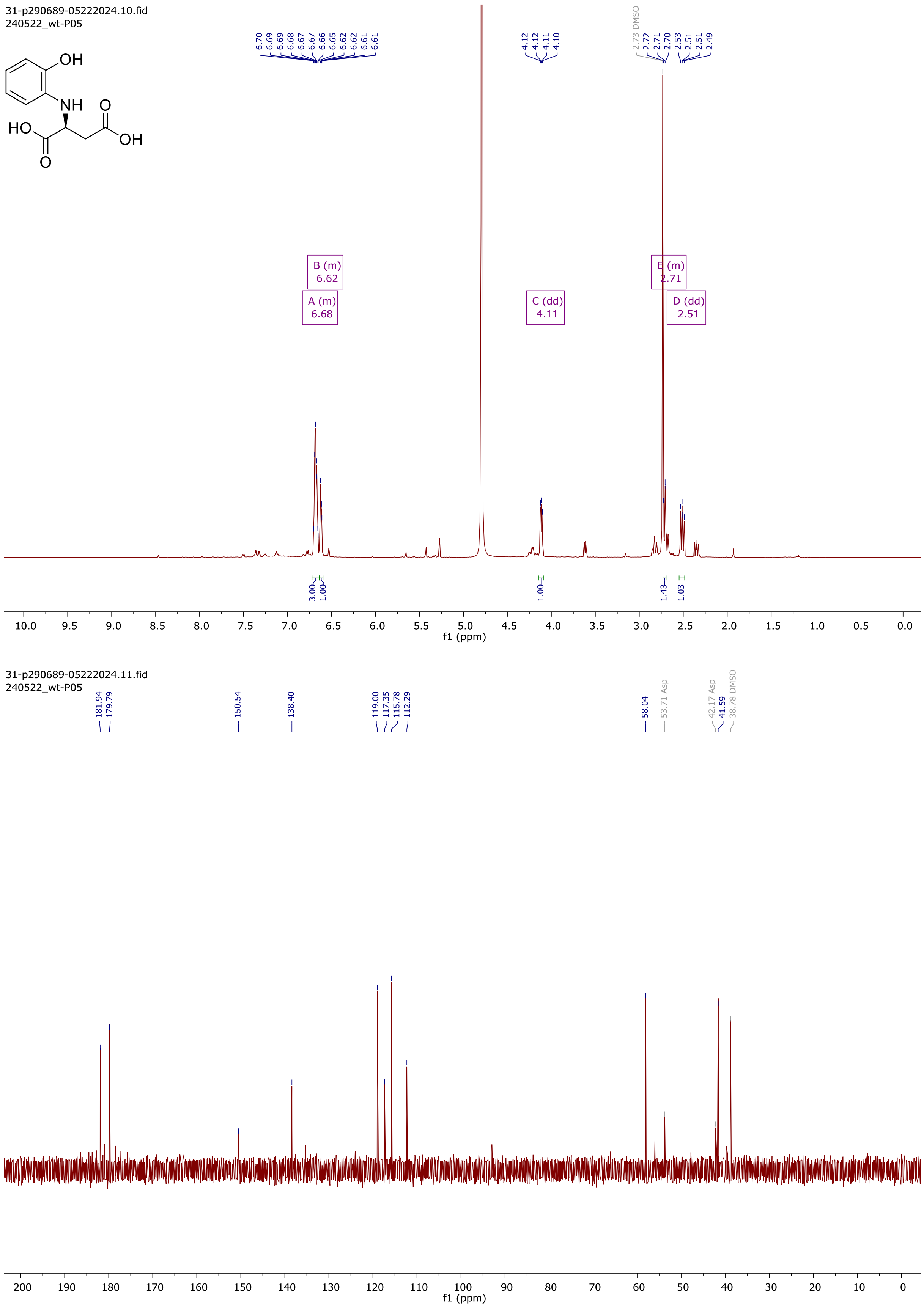

Figure S27. ^{1}H NMR (top) and ^{13}C NMR (bottom) of (2-hydroxyphenyl)-*L*-aspartic acid (***enz*-3f** obtained with EDDS lyase WT) in 0.1 M NaOD/D_2O, contains traces of DMSO and aspartic acid

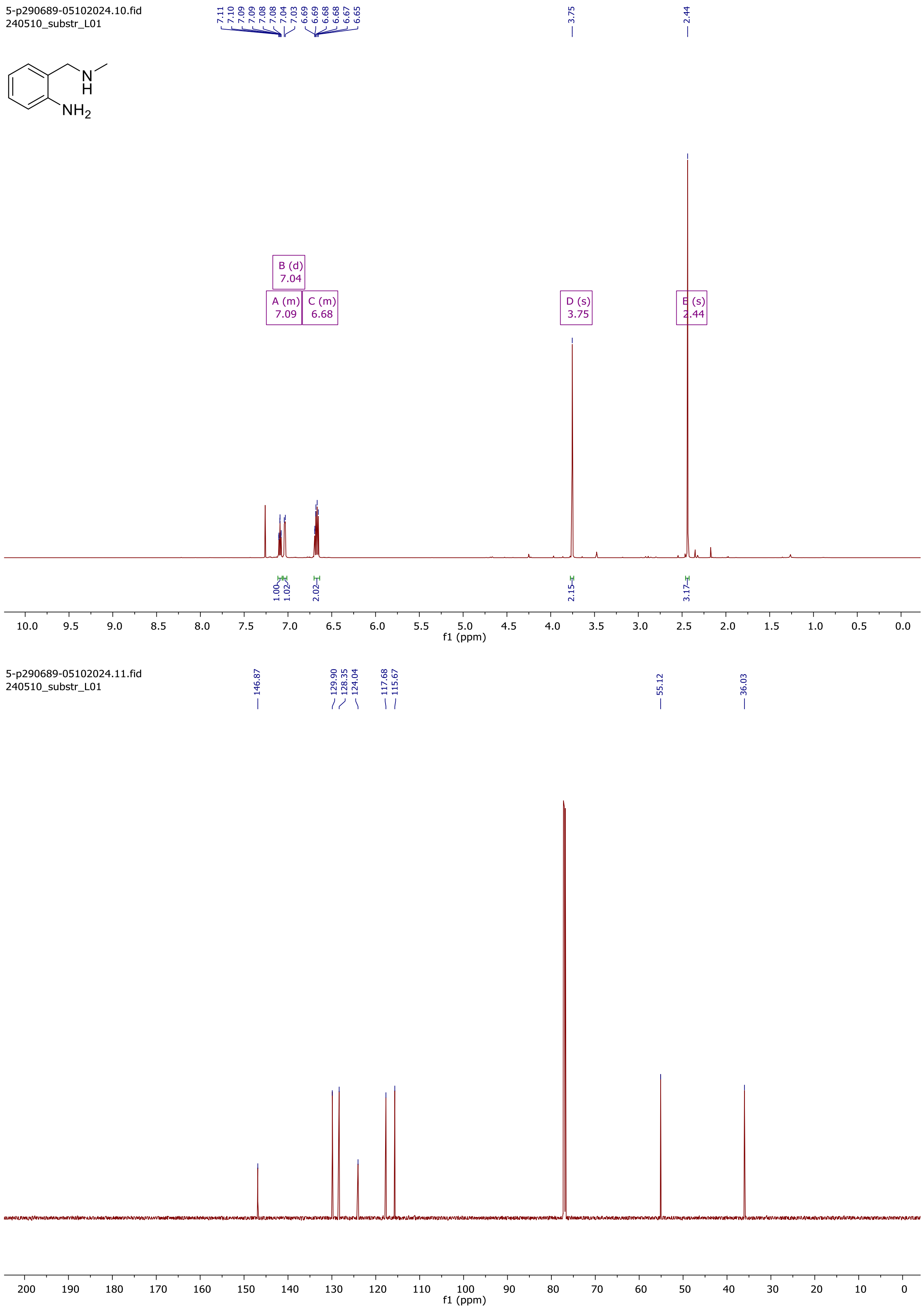

Figure S28. ^{1}H NMR (top) and ^{13}C NMR (bottom) of 2-((methylamino)methyl)aniline (**2a**) in $CDCl_3$

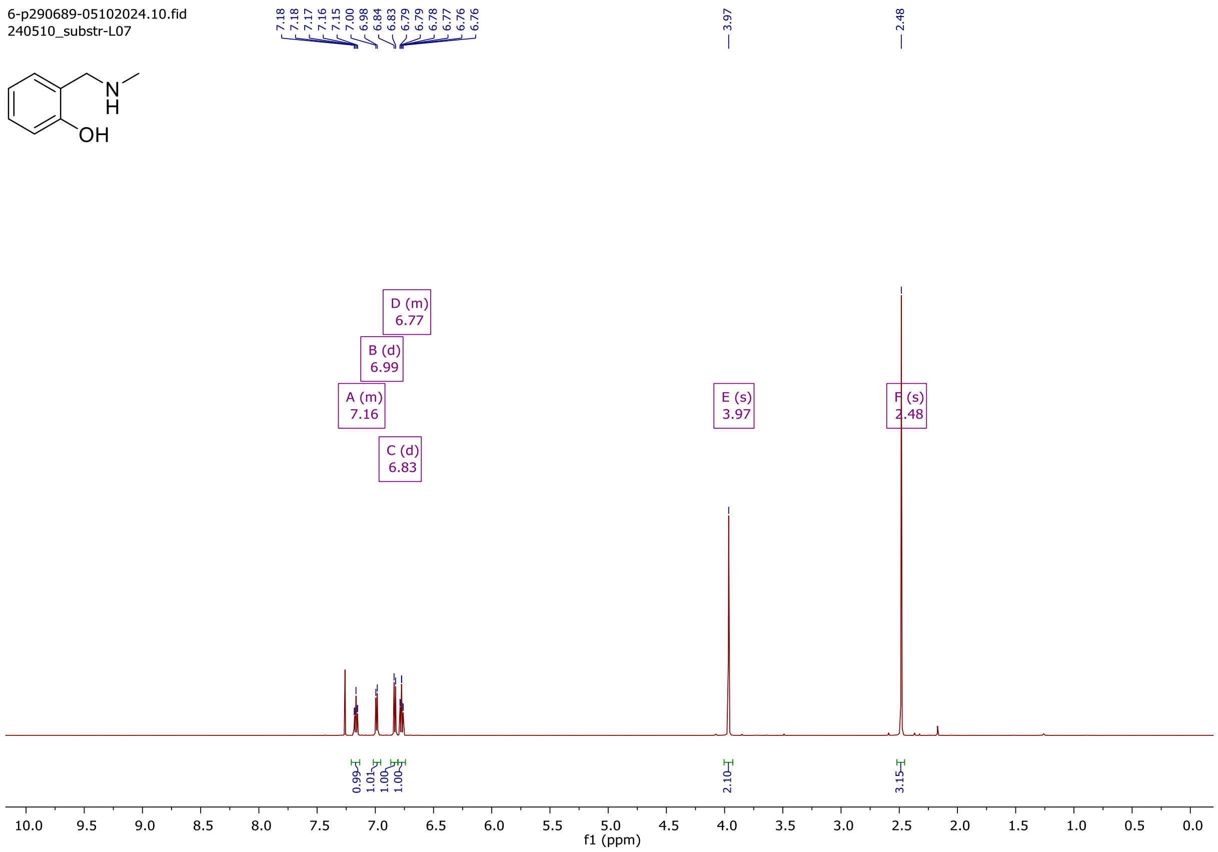

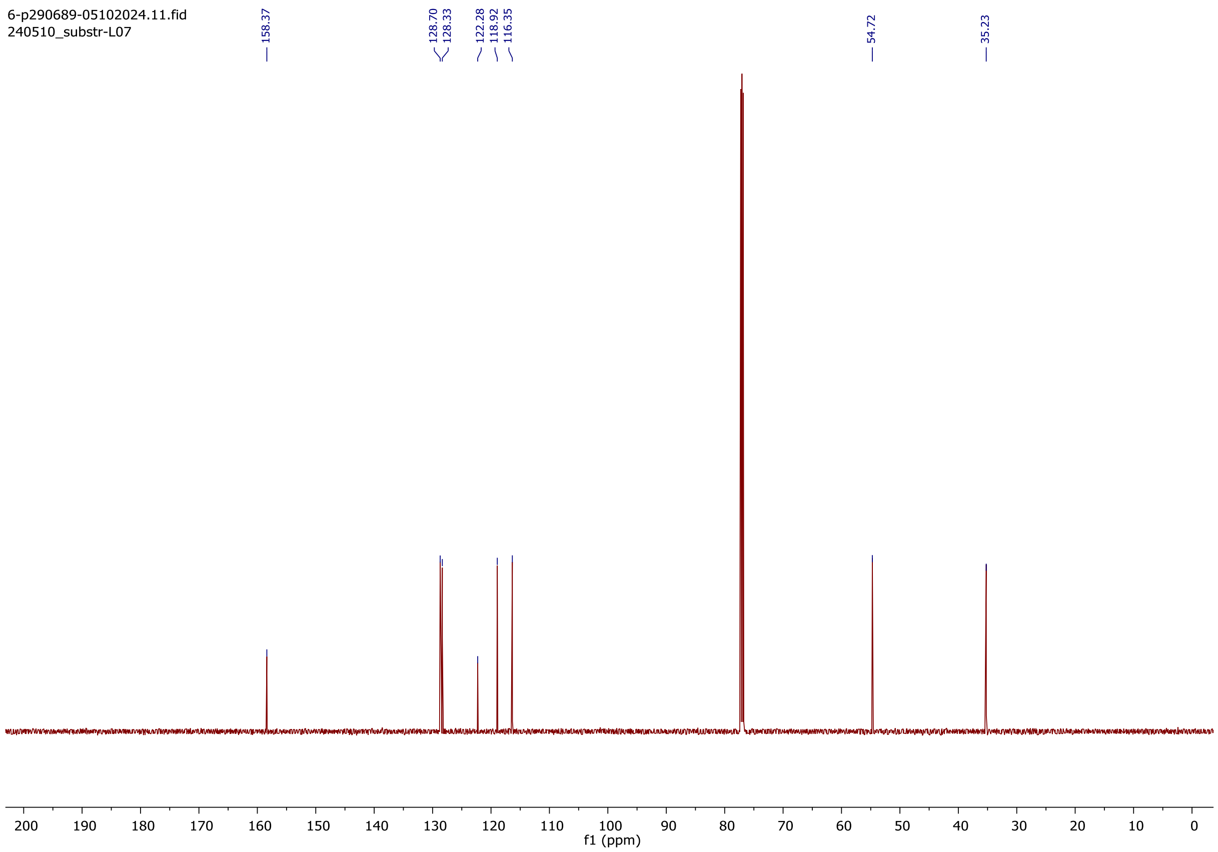

Figure S29. ^{1}H NMR (top) and ^{13}C NMR (bottom) of 2-((methylamino)methyl)phenol (**2d**) in $CDCl_3$

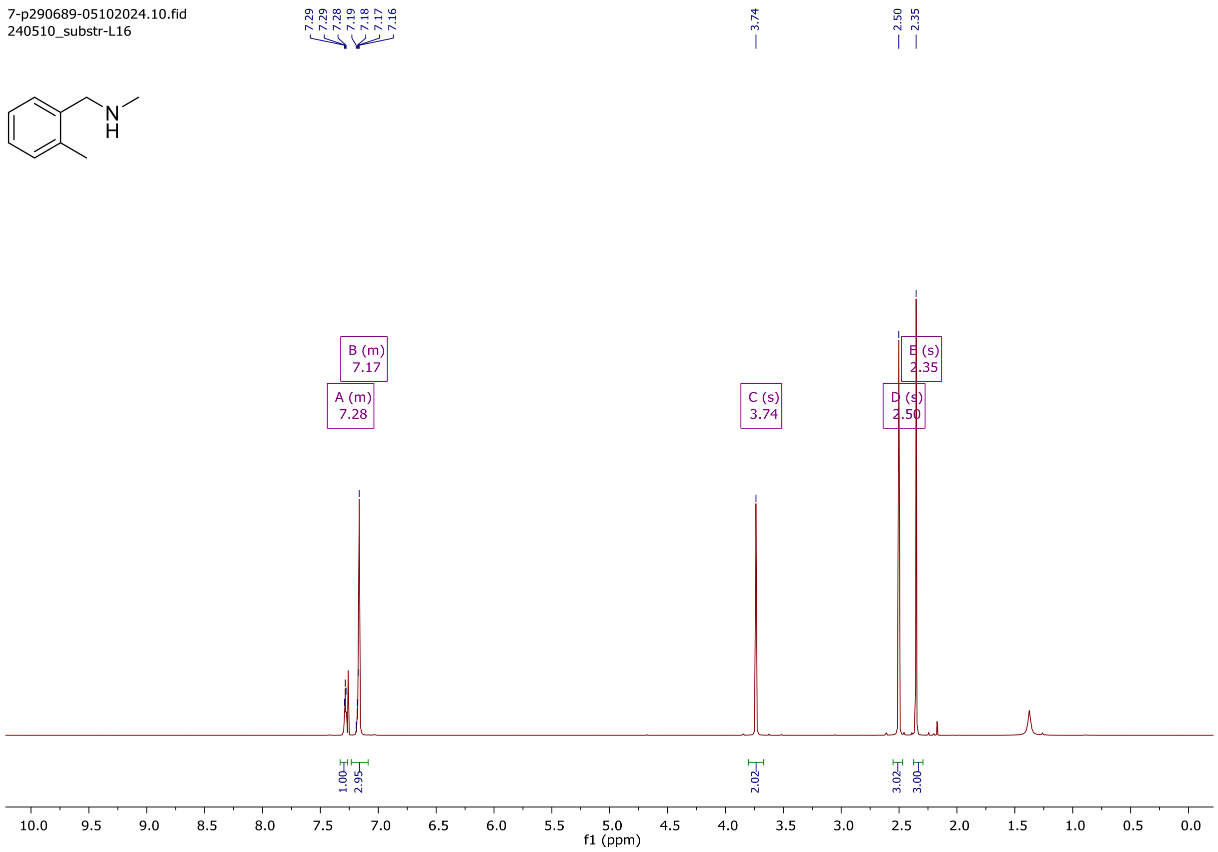

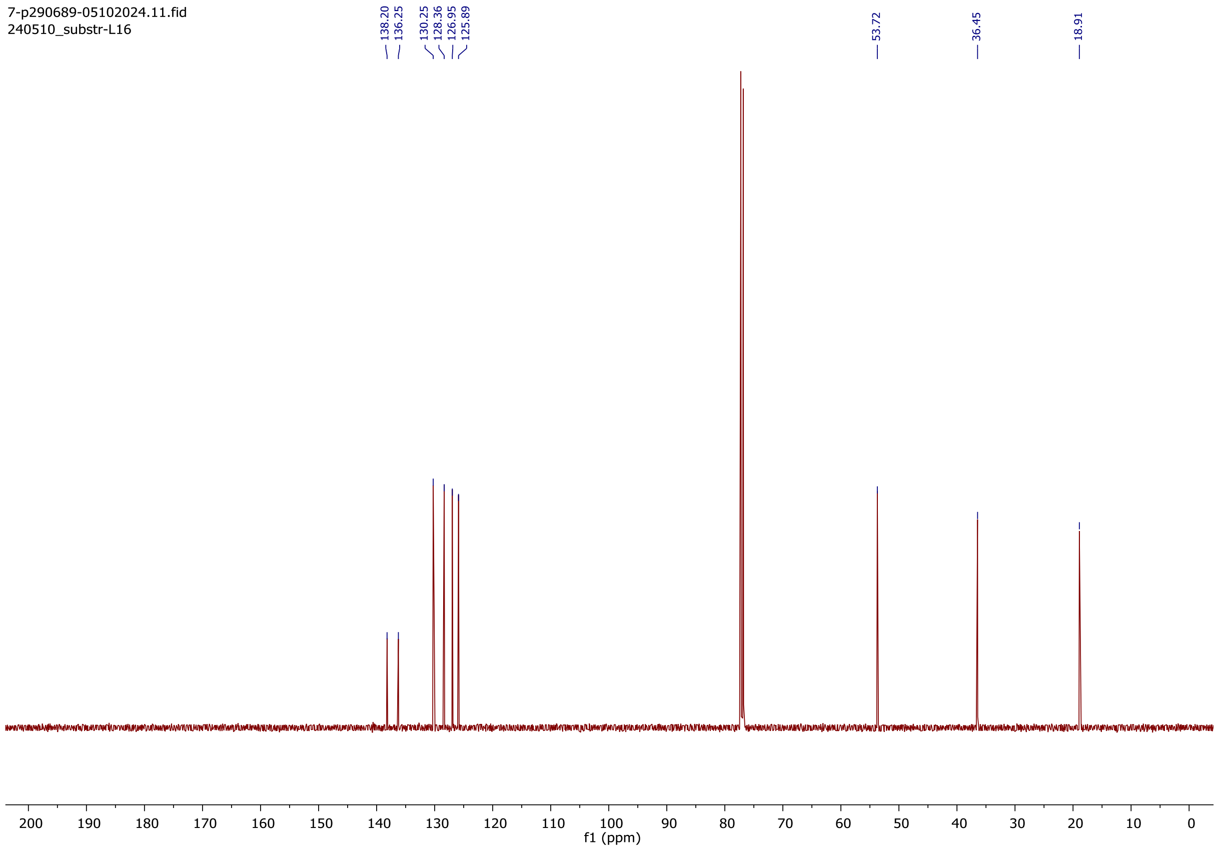

Figure S30. ^{1}H NMR (top) and ^{13}C NMR (bottom) of *N*-methyl-1-(o-tolyl)methanamine (**2h**) in $CDCl_3$

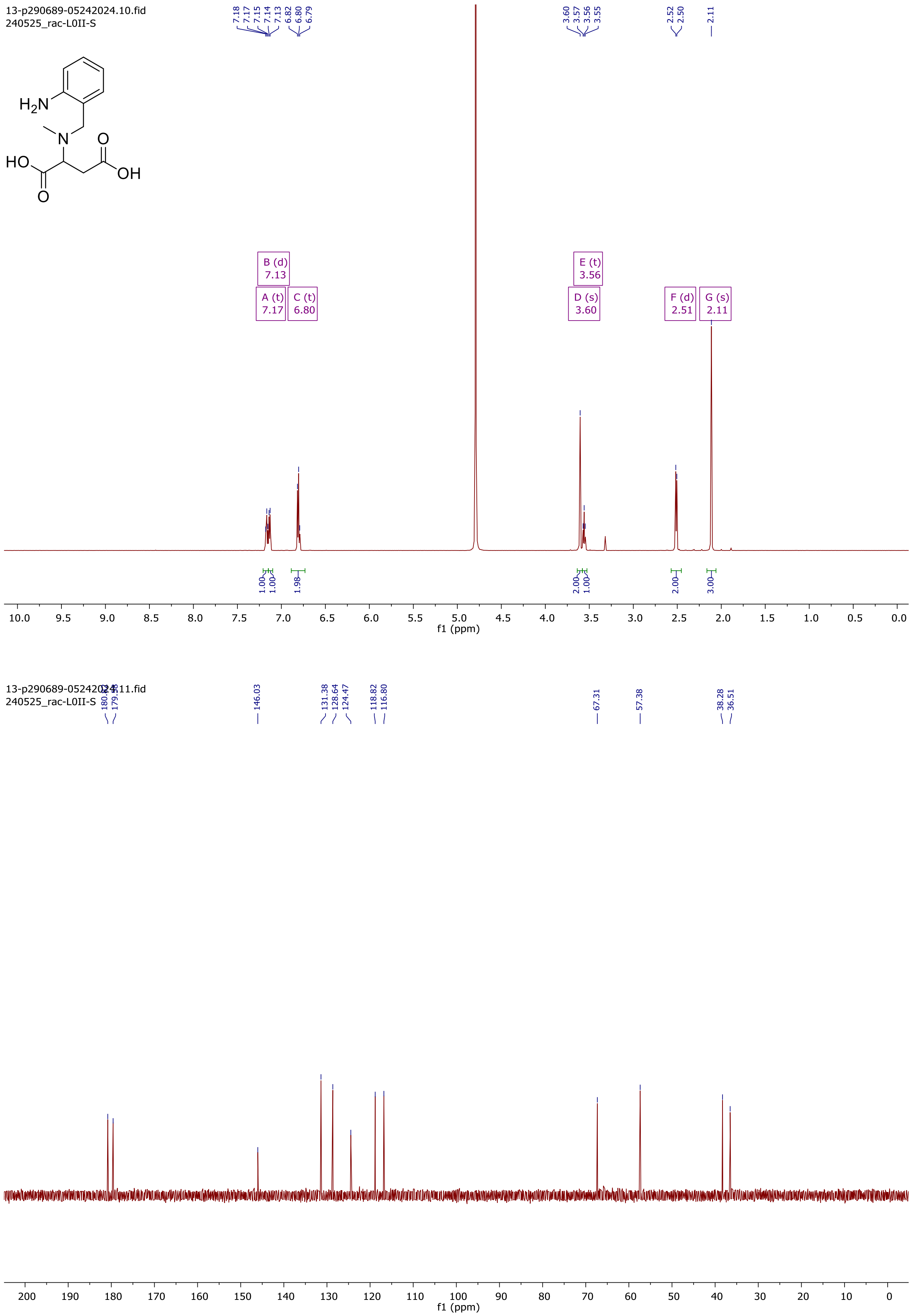

Figure S31. ^{1}H NMR (top) and ^{13}C NMR (bottom) of *N*-(2-aminobenzyl)-*N*-methylaspartic acid (***rac*-5a**) in 0.1 M NaOD/D_2O

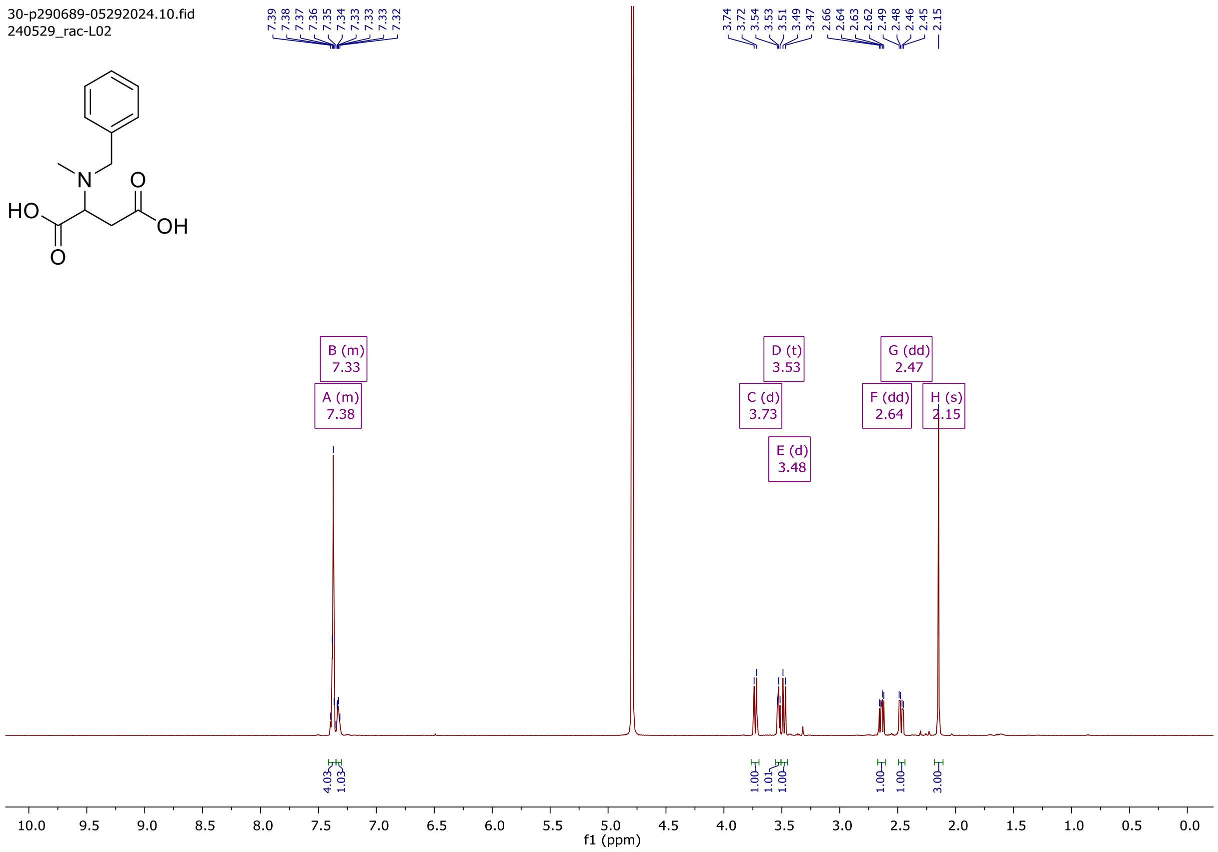

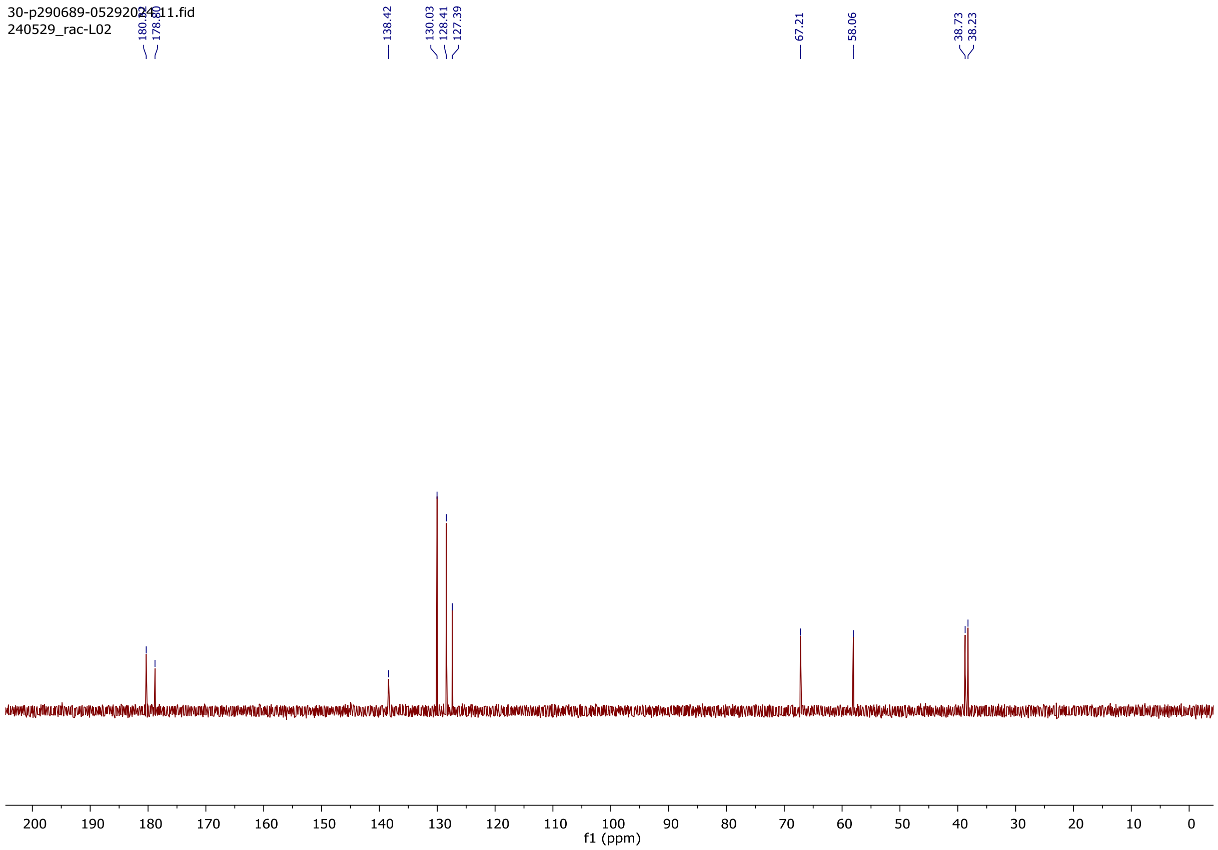

Figure S32. ^{1}H NMR (top) and ^{13}C NMR (bottom) of *N*-(benzyl)-*N*-methylaspartic acid (***rac*-5b**) in 0.1 M NaOD/D_2O

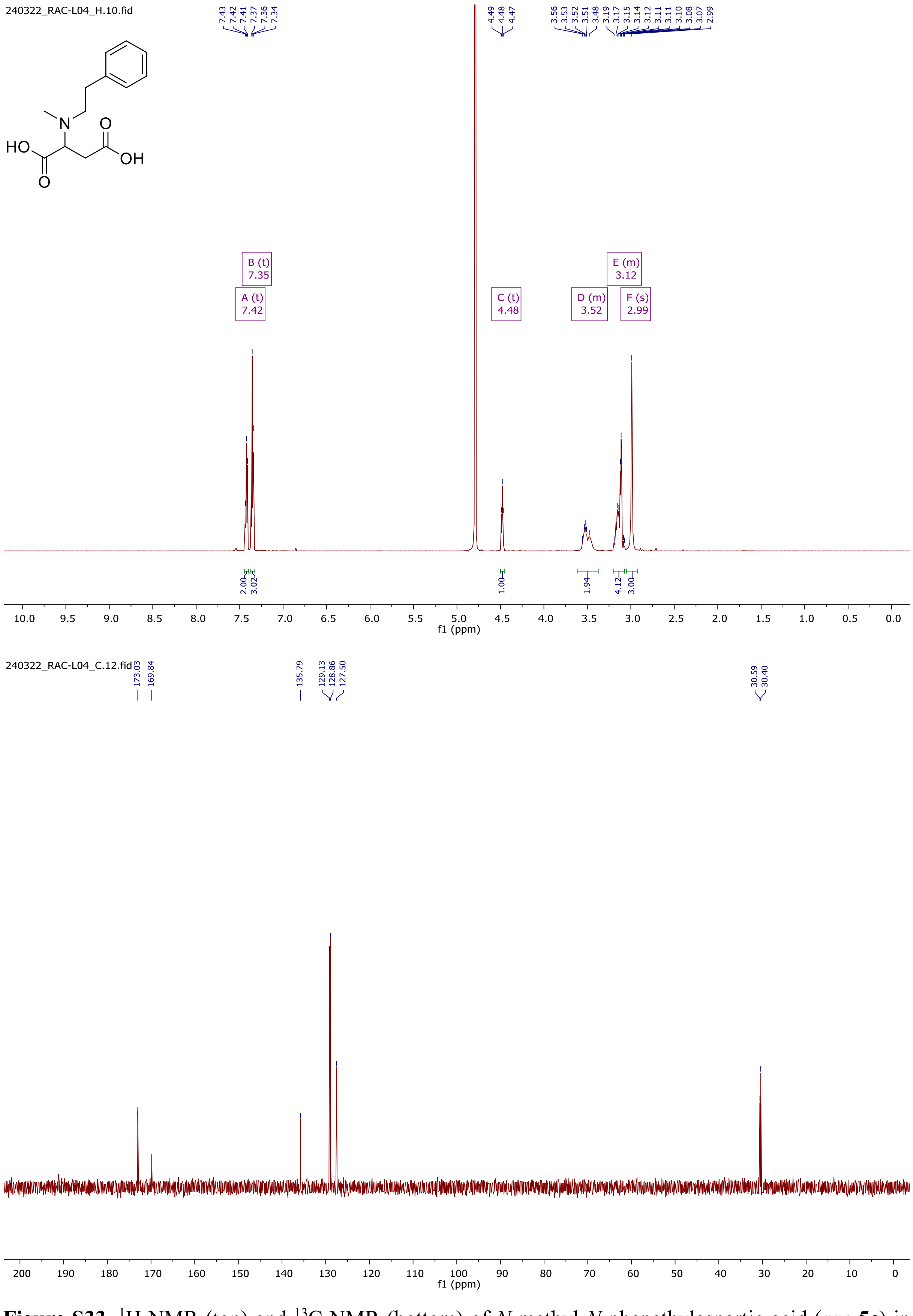

Figure S33. ^{1}H NMR (top) and ^{13}C NMR (bottom) of *N*-methyl-*N*-phenethylaspartic acid (***rac*-5c**) in 0.1 M DCl/D_2O

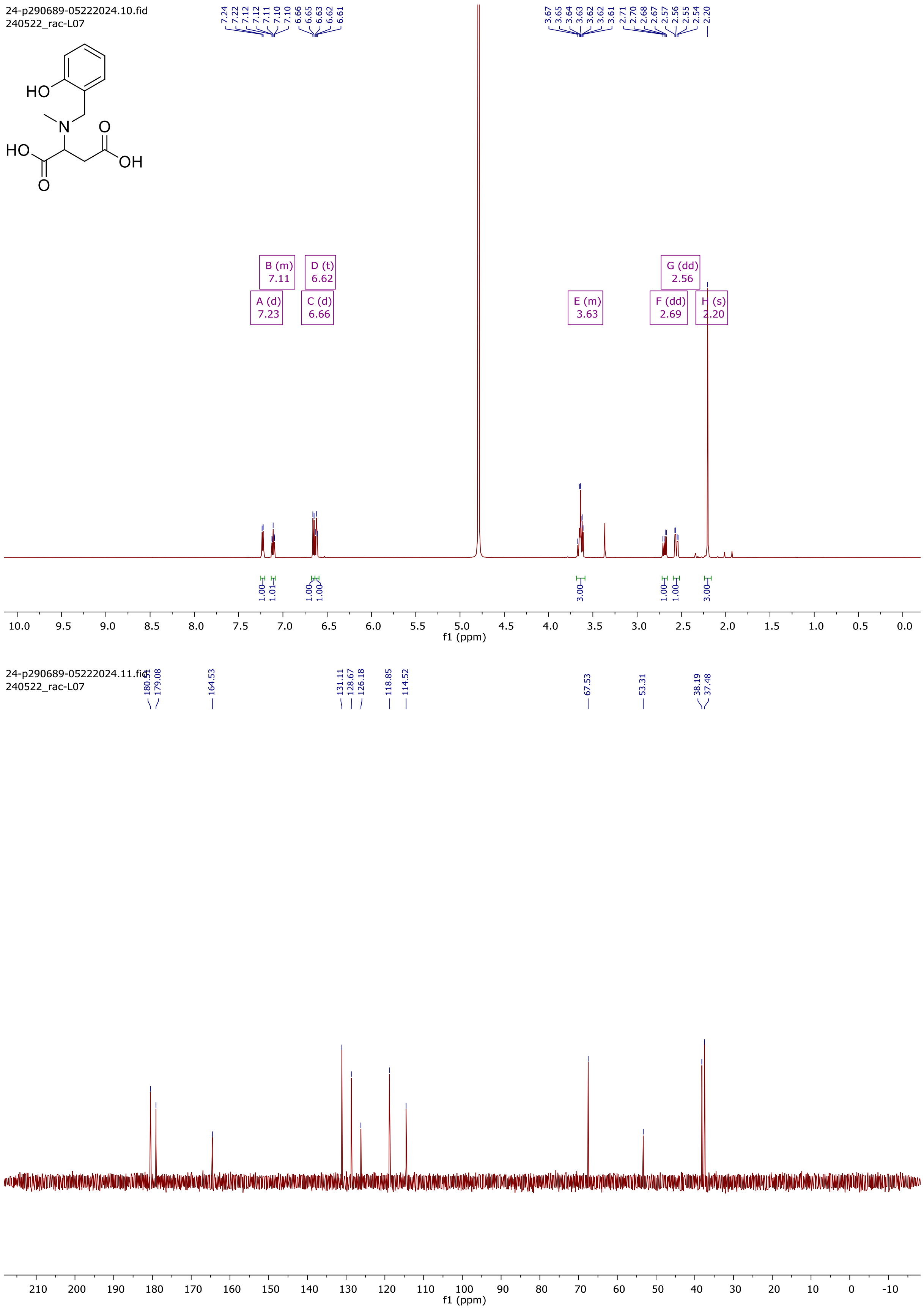

Figure S34. ^{1}H NMR (top) and ^{13}C NMR (bottom) of *N*-(2-hydroxybenzyl)-*N*-methylaspartic acid (***rac*-5d**) in 0.1 M NaOD/D_2O

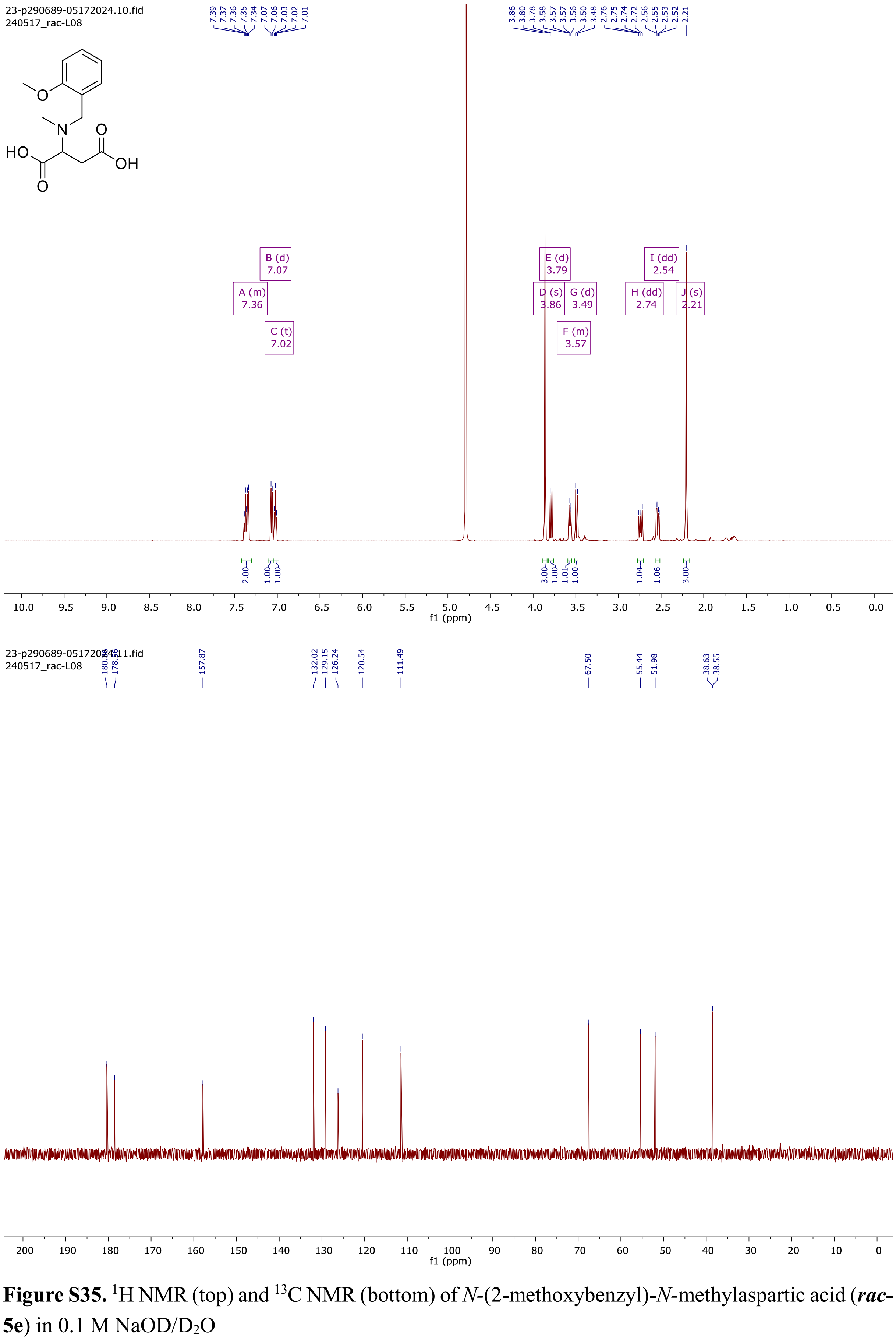

Figure S35. ^{1}H NMR (top) and ^{13}C NMR (bottom) of *N*-(2-methoxybenzyl)-*N*-methylaspartic acid (***rac*-5e**) in 0.1 M NaOD/D_2O

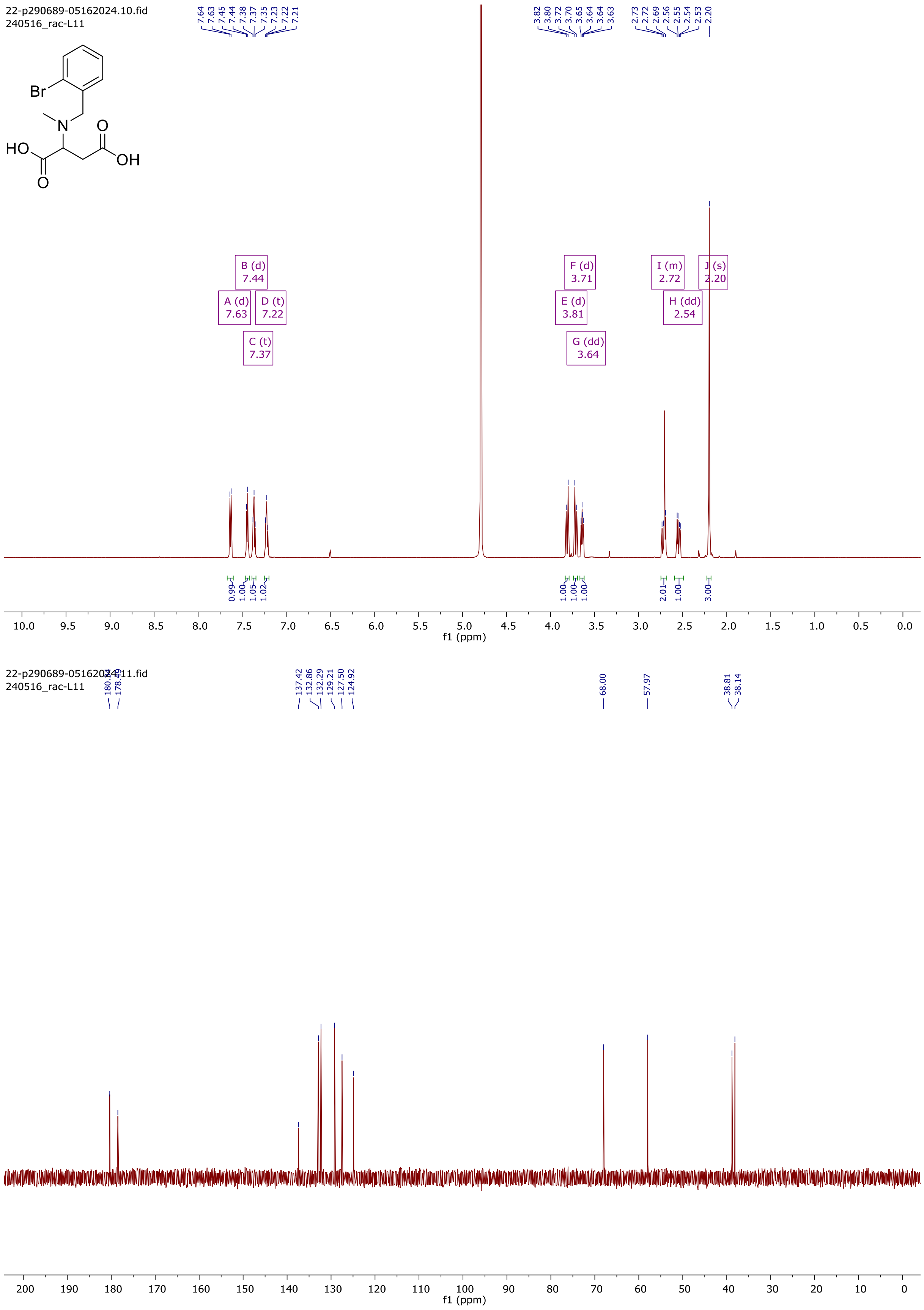

Figure S36. ^{1}H NMR (top) and ^{13}C NMR (bottom) of *N*-(2-bromobenzyl)-*N*-methylaspartic acid (***rac*-5f**) in 0.1 M NaOD/D_2O

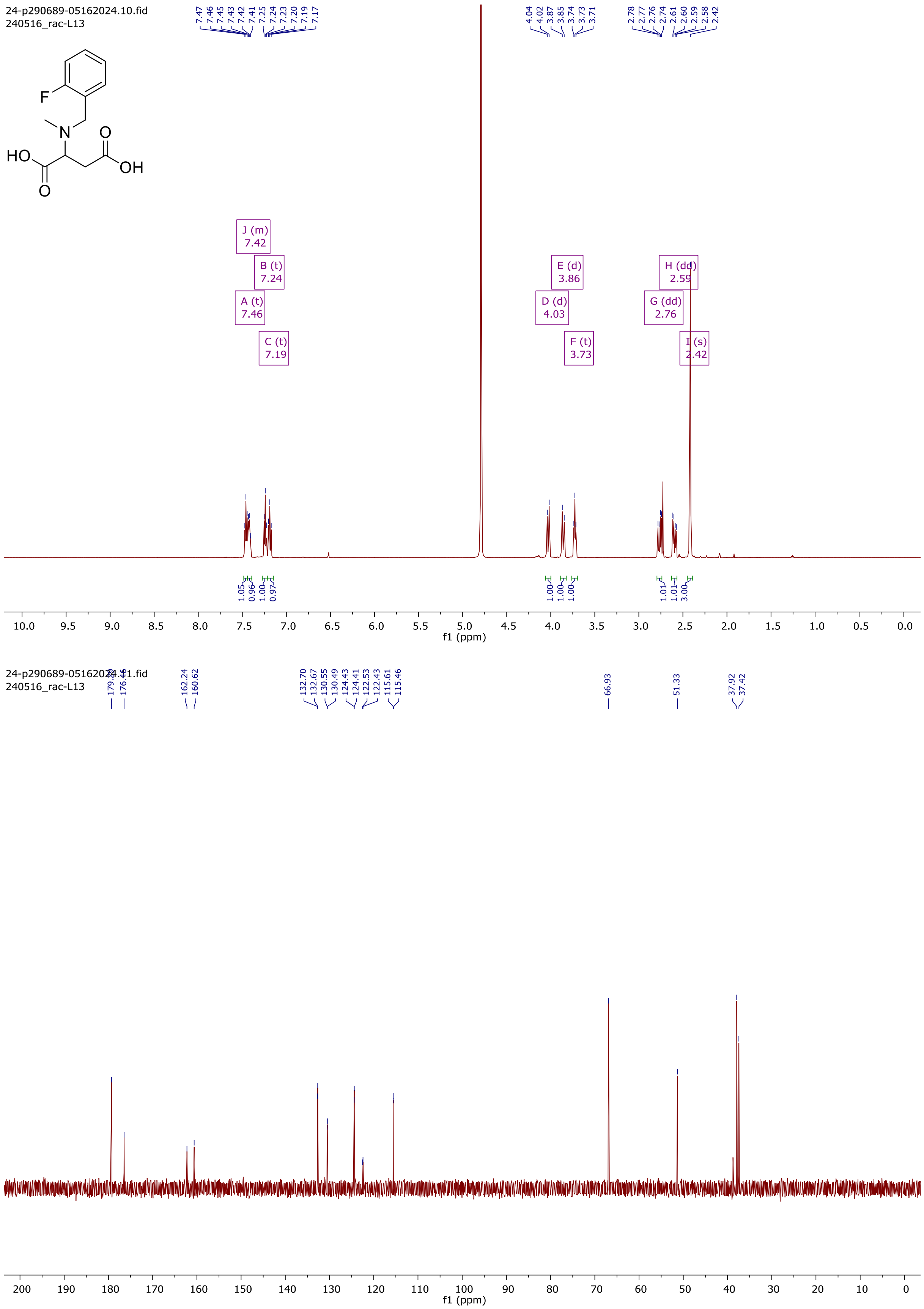

Figure S37. ^{1}H NMR (top) and ^{13}C NMR (bottom) of *N*-(2-fluorobenzyl)-*N*-methylaspartic acid (***rac*-5g**) in 0.1 M NaOD/D_2O

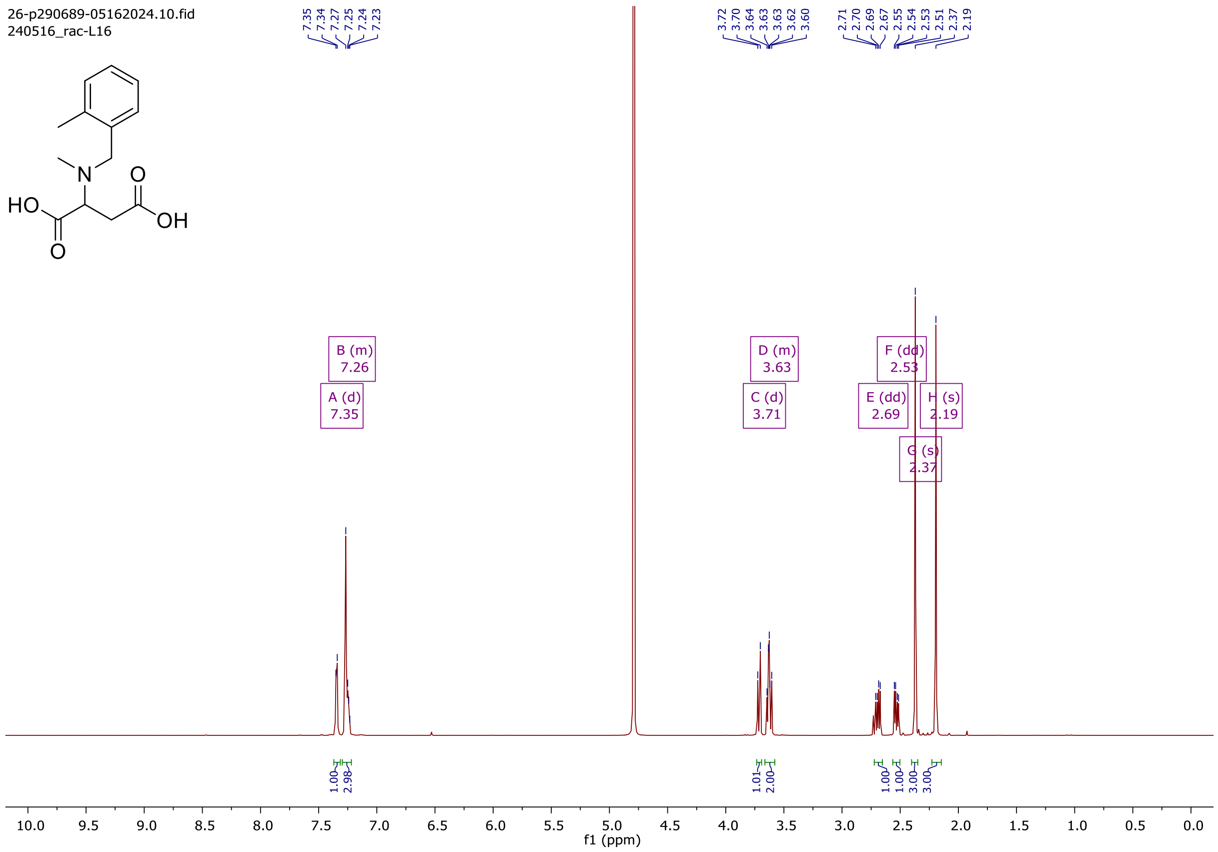

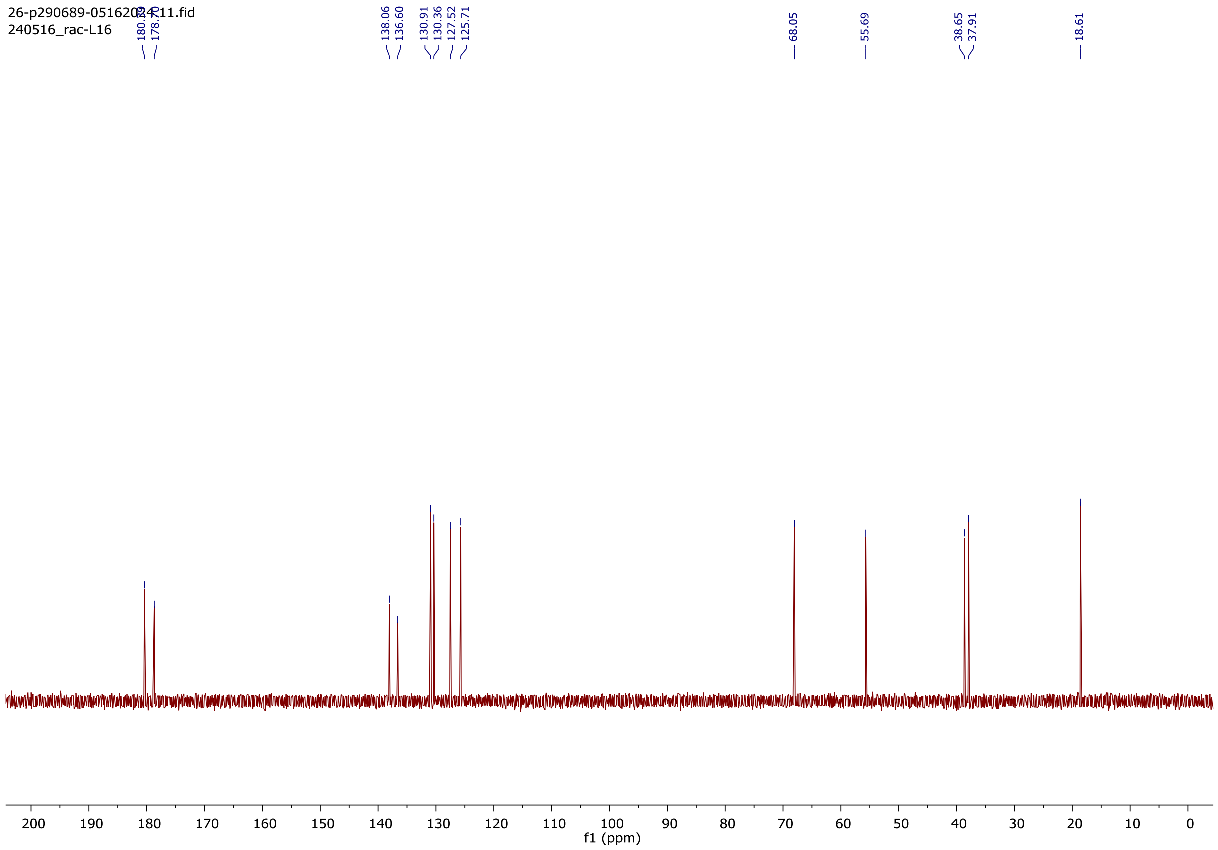

Figure S38. ^{1}H NMR (top) and ^{13}C NMR (bottom) of *N*-methyl-*N*-(2-methylbenzyl)aspartic acid (***rac*-5h**) in 0.1 M NaOD/D_2O

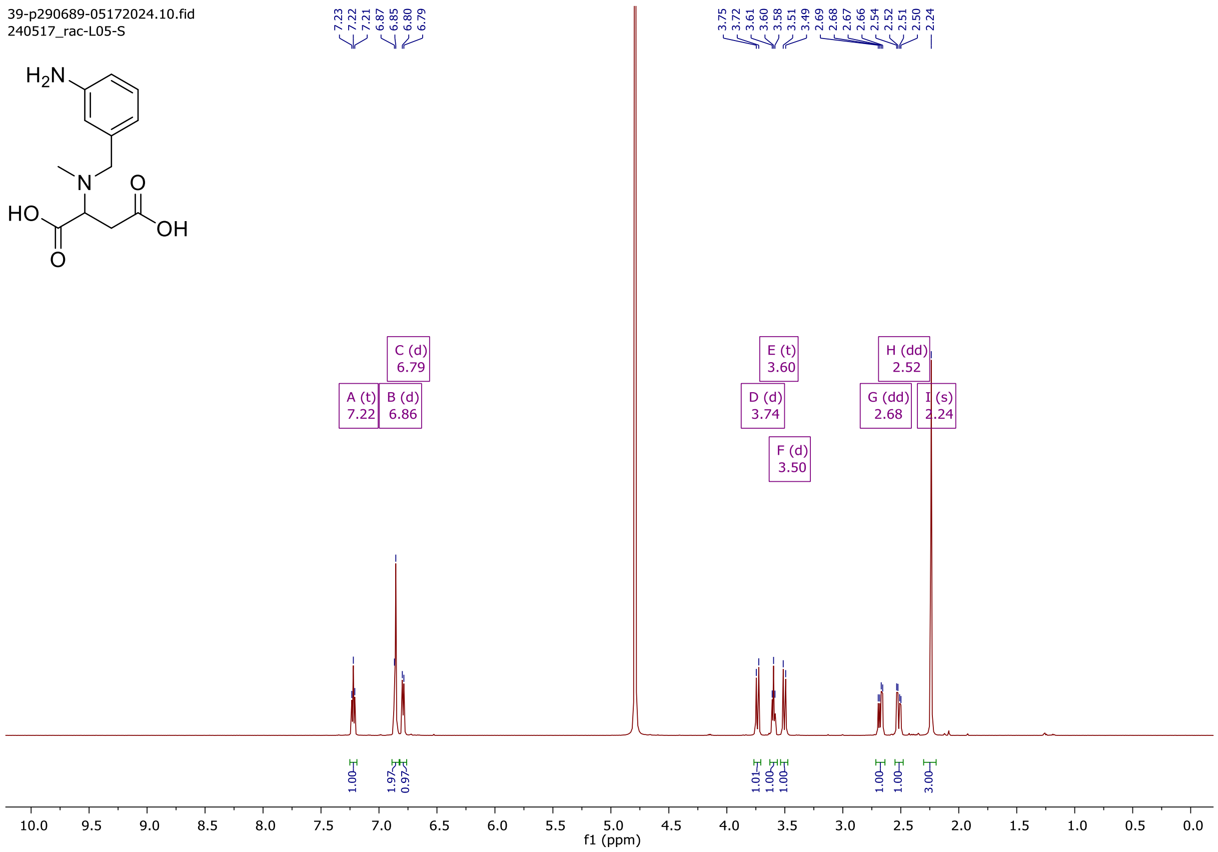

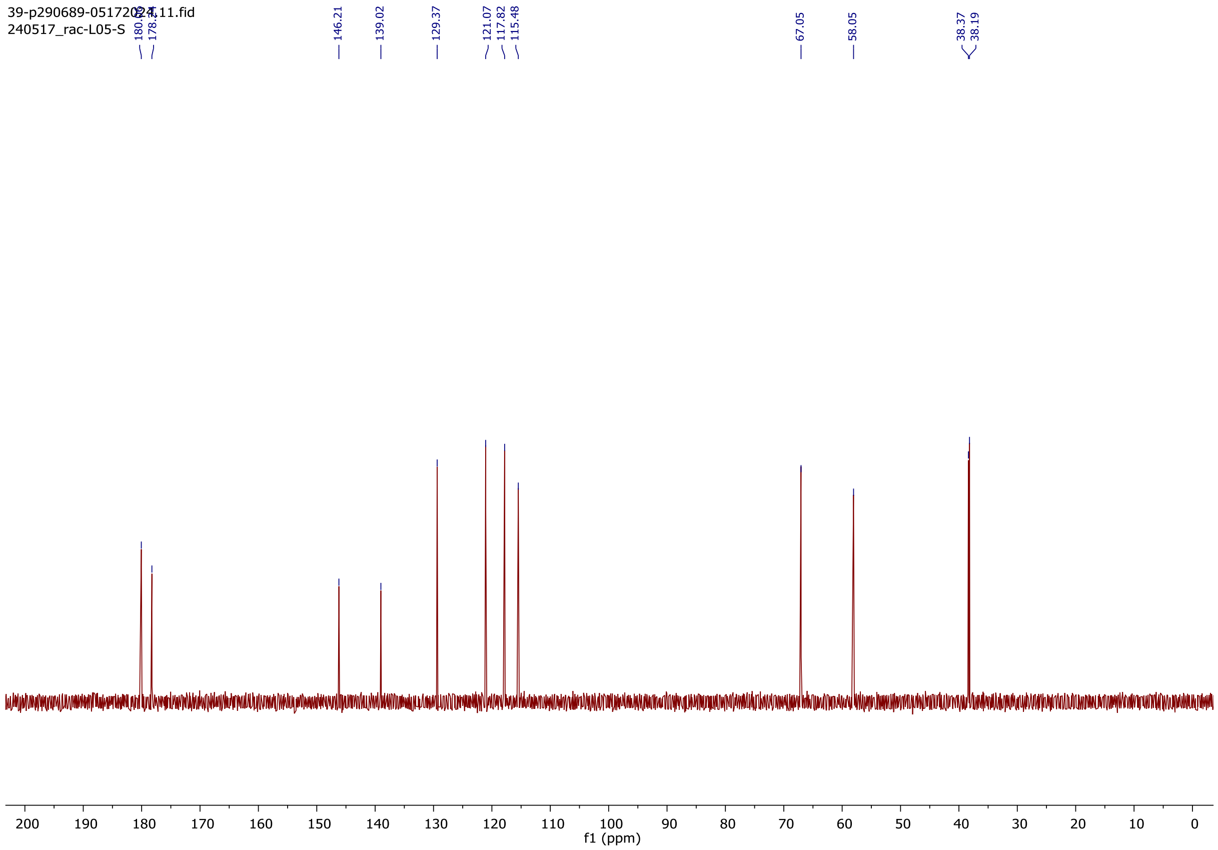

Figure S39. ^{1}H NMR (top) and ^{13}C NMR (bottom) of *N*-(3-aminobenzyl)-*N*-methylaspartic acid (***rac*-5i**) in 0.1 M NaOD/D_2O

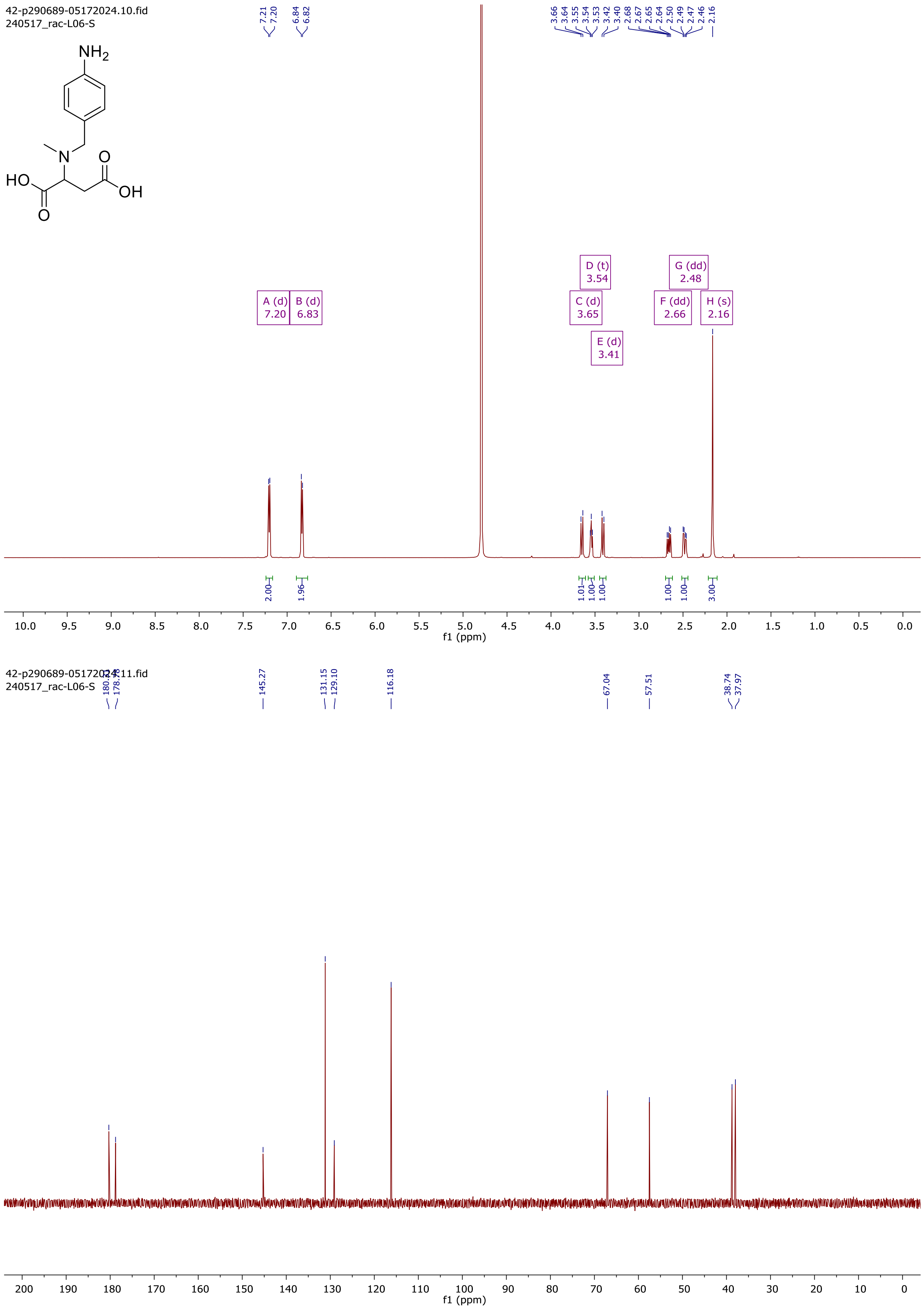

Figure S40. ^{1}H NMR (top) and ^{13}C NMR (bottom) of *N*-(4-aminobenzyl)-*N*-methylaspartic acid (***rac*-5j**) in 0.1 M NaOD/D_2O

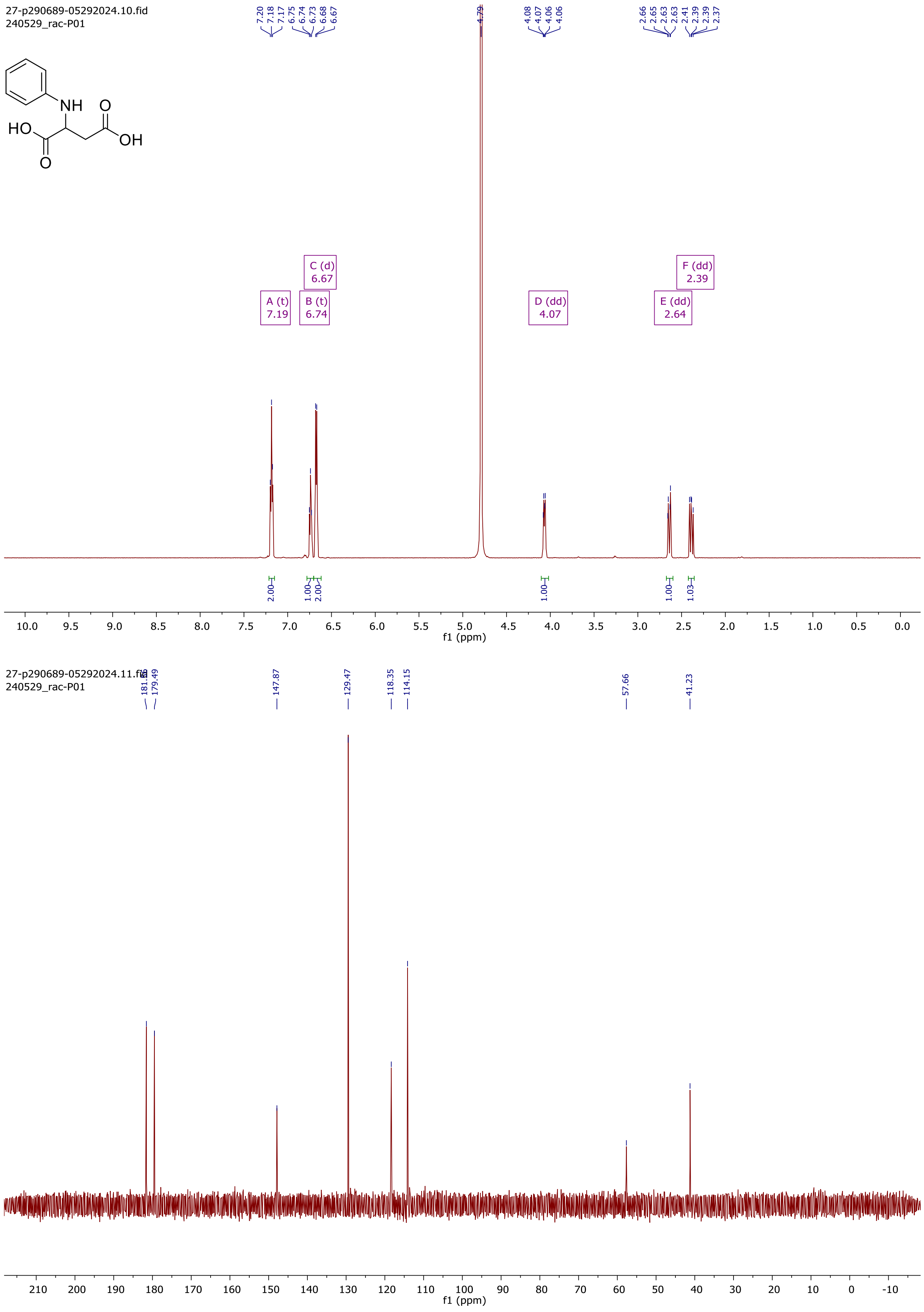

Figure S41. ^{1}H NMR (top) and ^{13}C NMR (bottom) of phenylaspartic acid (***rac*-3b**) in 0.1 M NaOD/D_2O

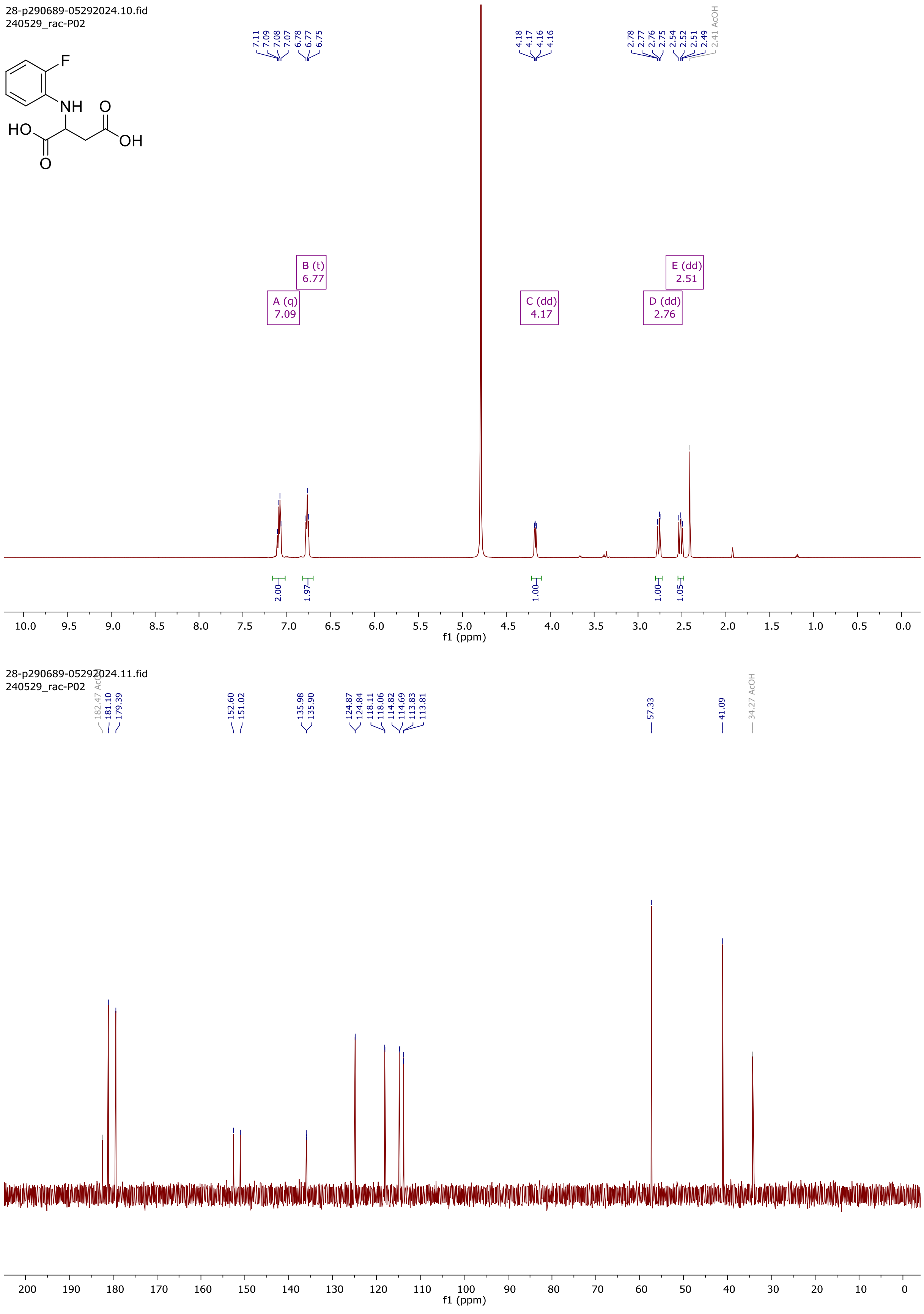

Figure S42. ^{1}H NMR (top) and ^{13}C NMR (bottom) of (2-fluorophenyl)aspartic acid (***rac*-3c**) in 0.1 M NaOD/D_2O, contains traces of acetic acid

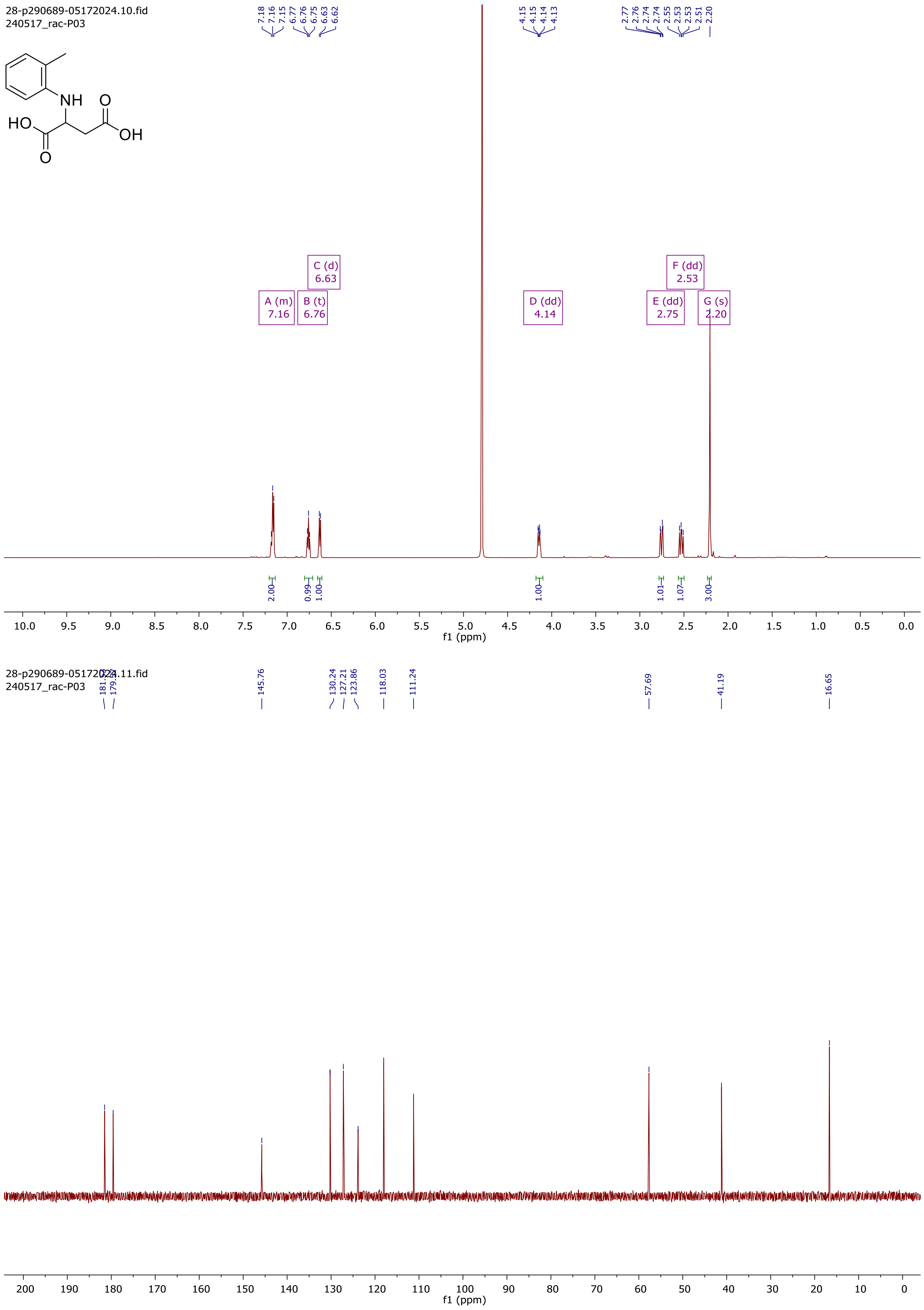

Figure S43. ^{1}H NMR (top) and ^{13}C NMR (bottom) of *o*-tolylaspartic acid (***rac*-3d**) in 0.1 M NaOD/D_2O

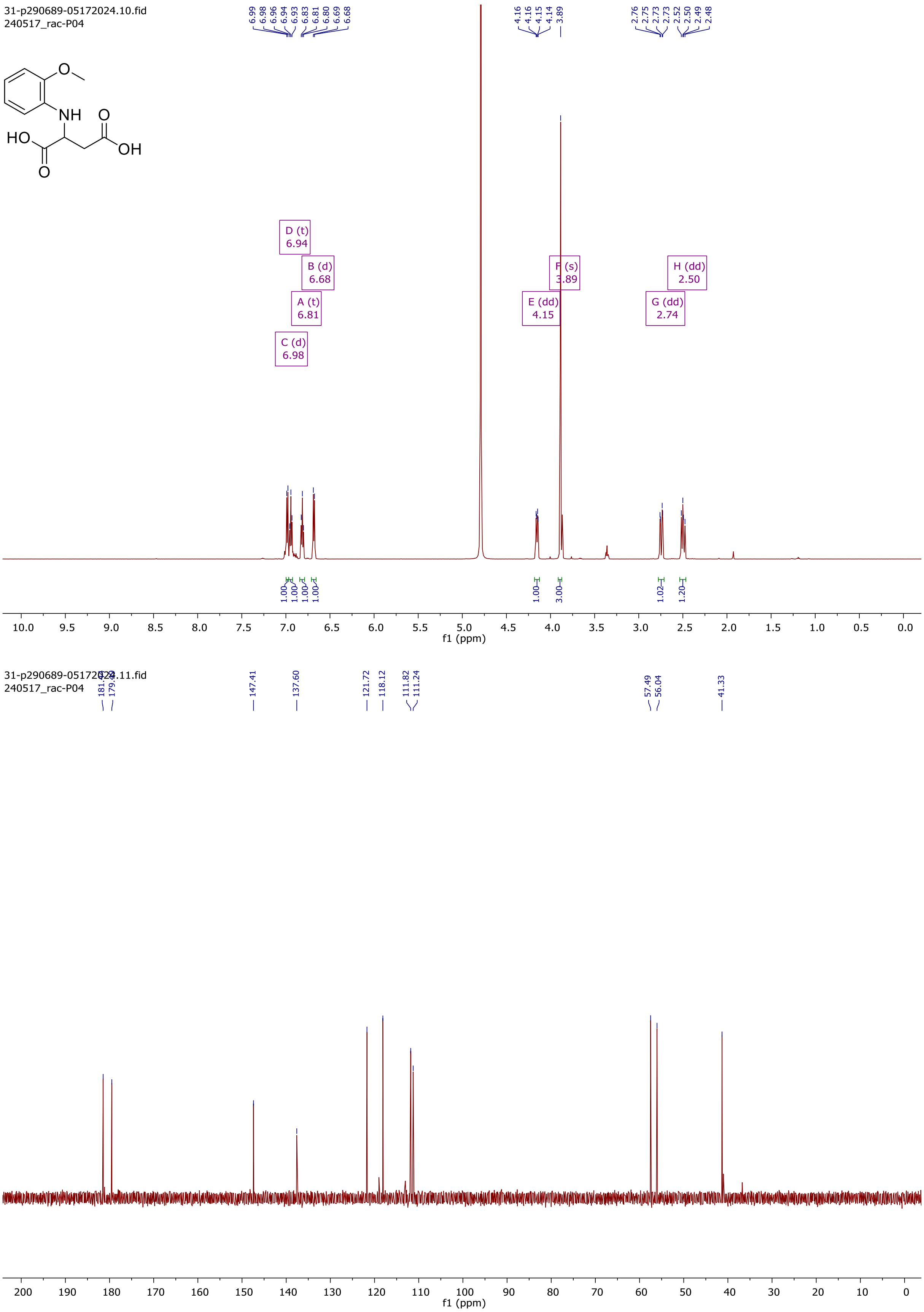

Figure S44. ^{1}H NMR (top) and ^{13}C NMR (bottom) of (2-methoxyphenyl)aspartic acid (***rac*-3e**) in 0.1 M NaOD/D_2O

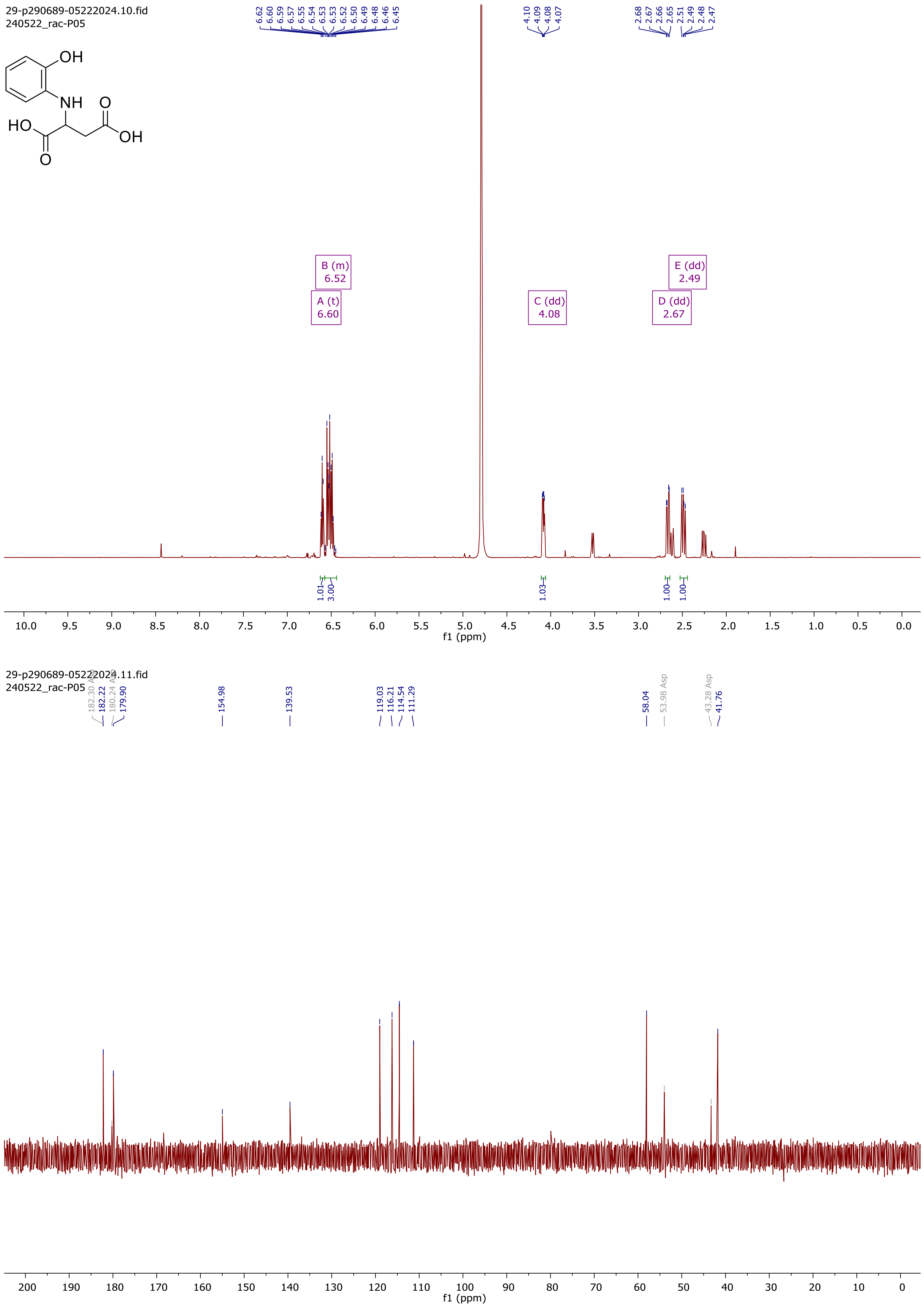

Figure S45. ^{1}H NMR (top) and ^{13}C NMR (bottom) of (2-hydroxyphenyl)aspartic acid (***rac*-3f**) in 0.1 M $NaOD/D_2O$, contains traces of aspartic acid

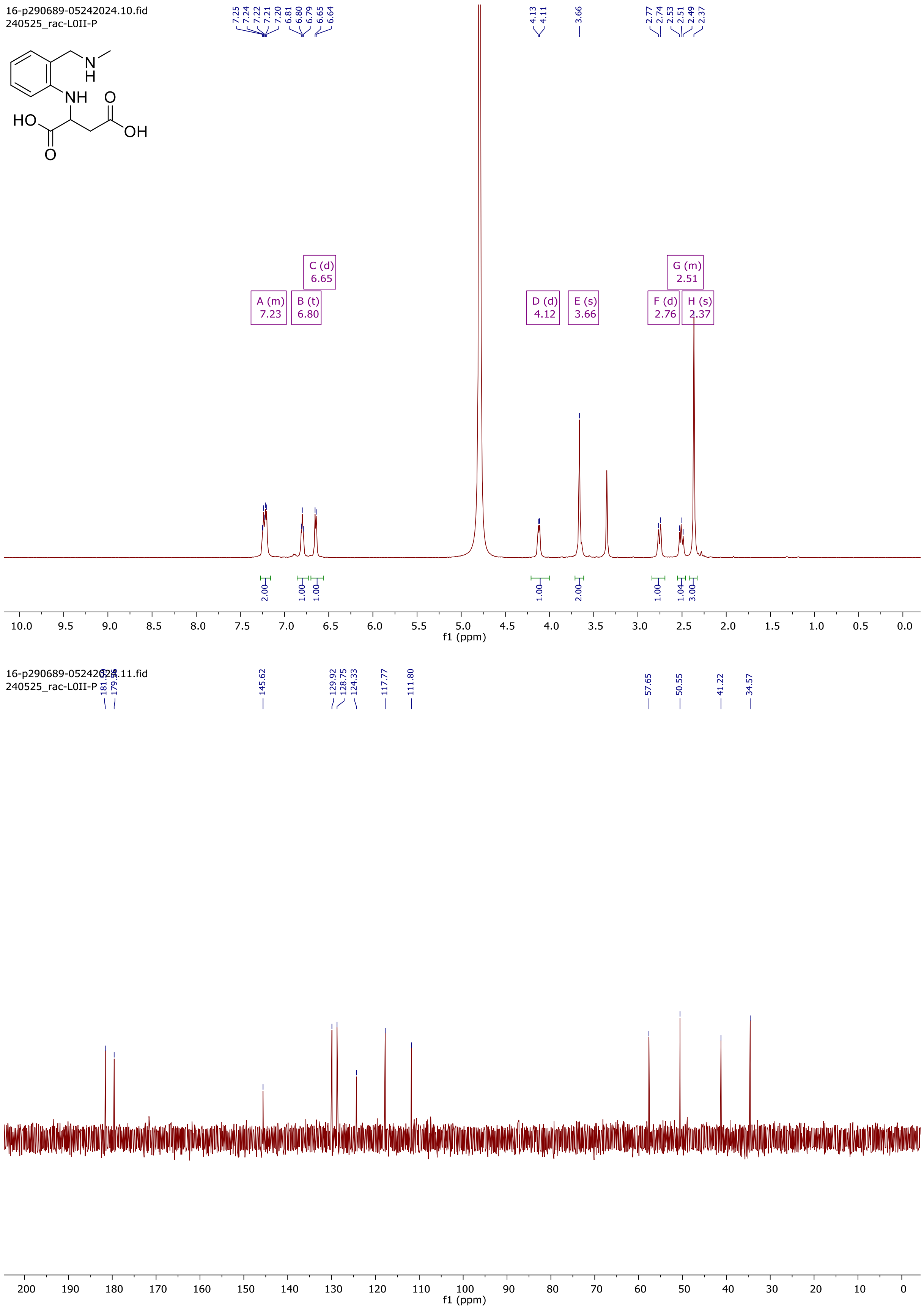

Figure S46. ^{1}H NMR (top) and ^{13}C NMR (bottom) of (2-((methylamino)methyl)phenyl)aspartic acid (***rac*-3a**) in 0.1 M NaOD/D_2O

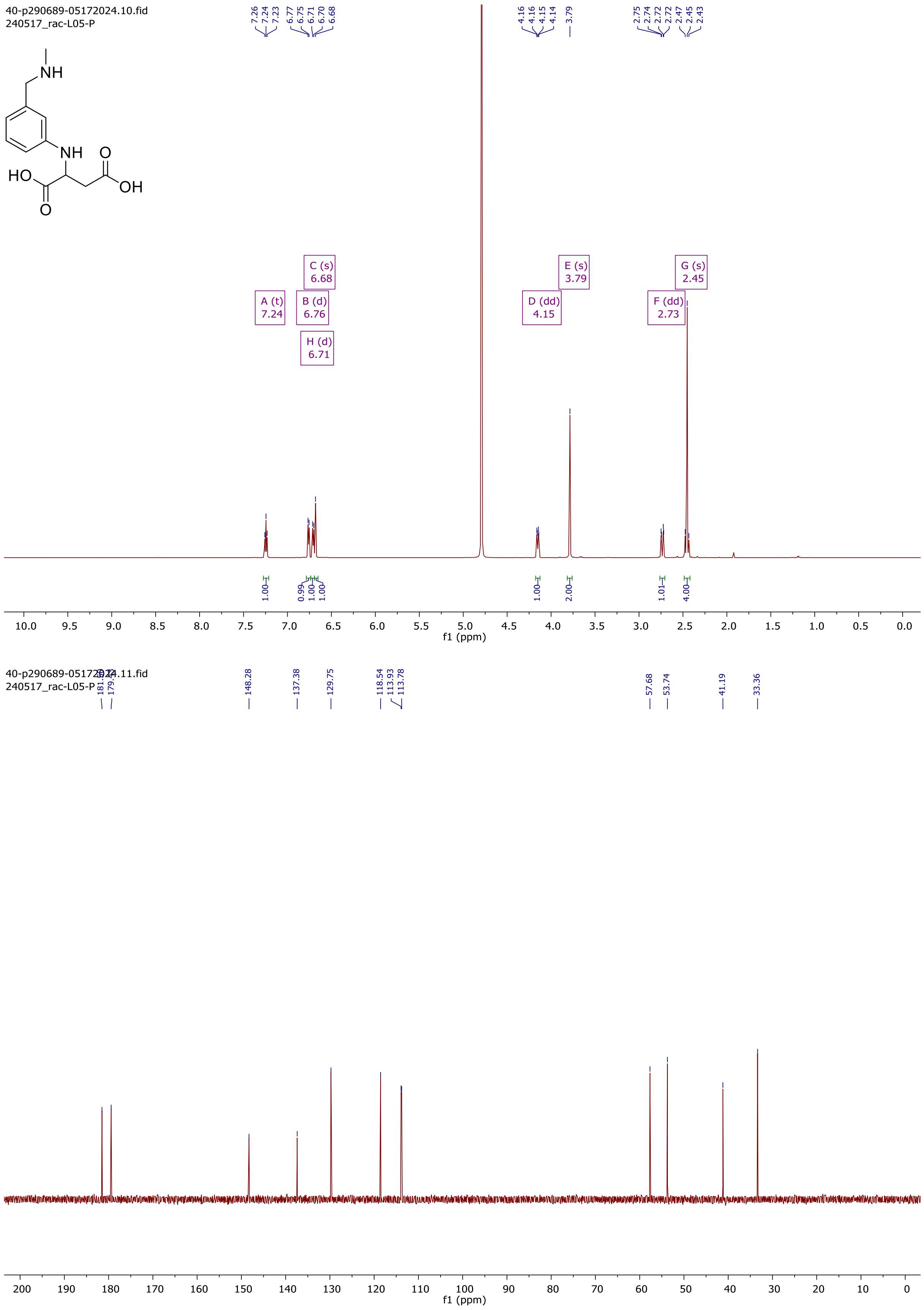

Figure S47. ^{1}H NMR (top) and ^{13}C NMR (bottom) of (3-((methylamino)methyl)phenyl)aspartic acid (***rac*-3i**) in 0.1 M NaOD/D_2O

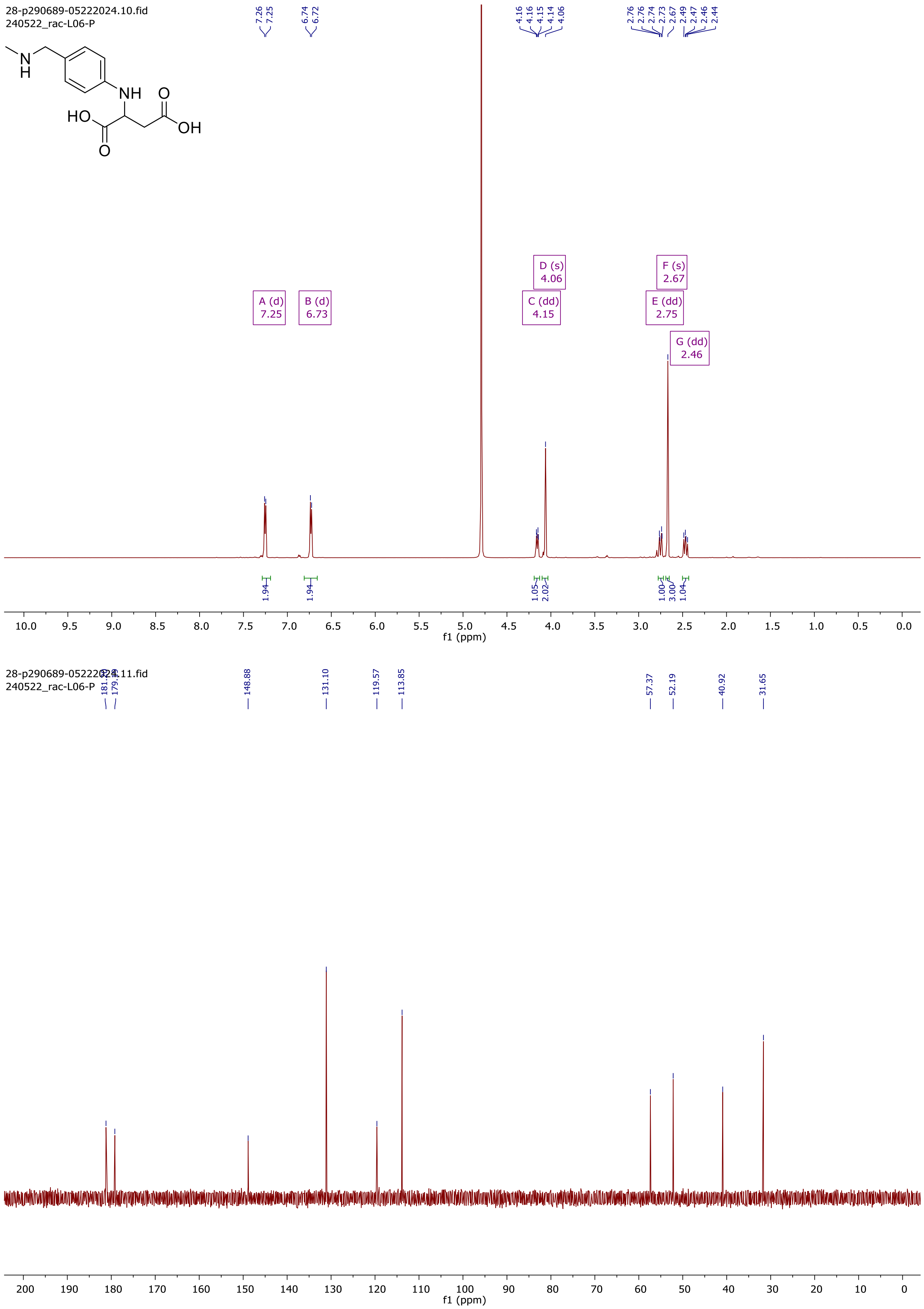

Figure S48. ^{1}H NMR (top) and ^{13}C NMR (bottom) of (4-((methylamino)methyl)phenyl)aspartic acid (***rac*-3j**) in 0.1 M NaOD/D_2O

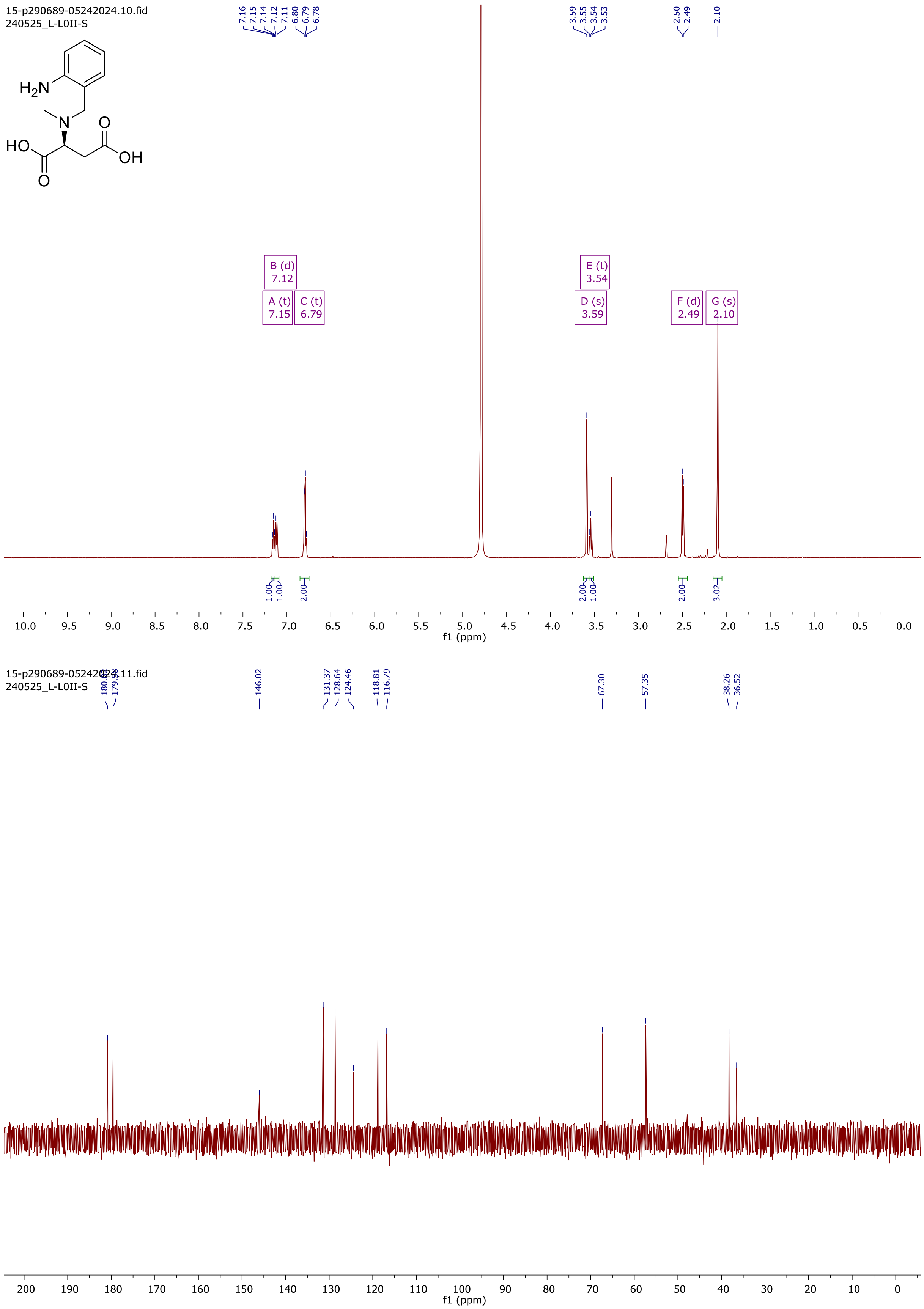

Figure S49. ^{1}H NMR (top) and ^{13}C NMR (bottom) of *N*-(2-aminobenzyl)-*N*-methyl-*L*-aspartic acid ((***S***)-**5a**) in 0.1 M NaOD/D_2O

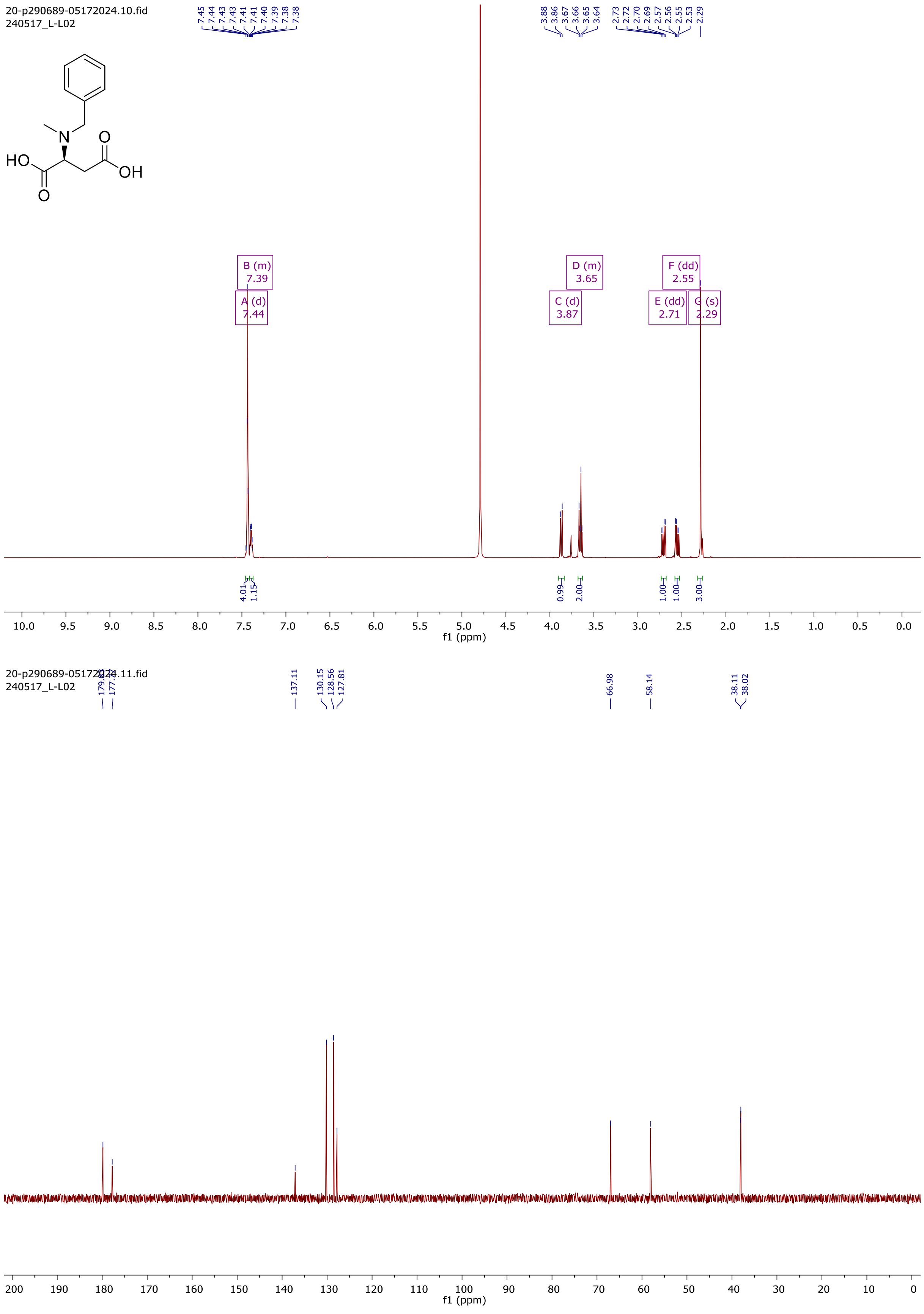

Figure S50. ^{1}H NMR (top) and ^{13}C NMR (bottom) of *N*-benzyl-*N*-methyl-*L*-aspartic acid (**(*S*)-5b**) in 0.1 M NaOD/D_2O

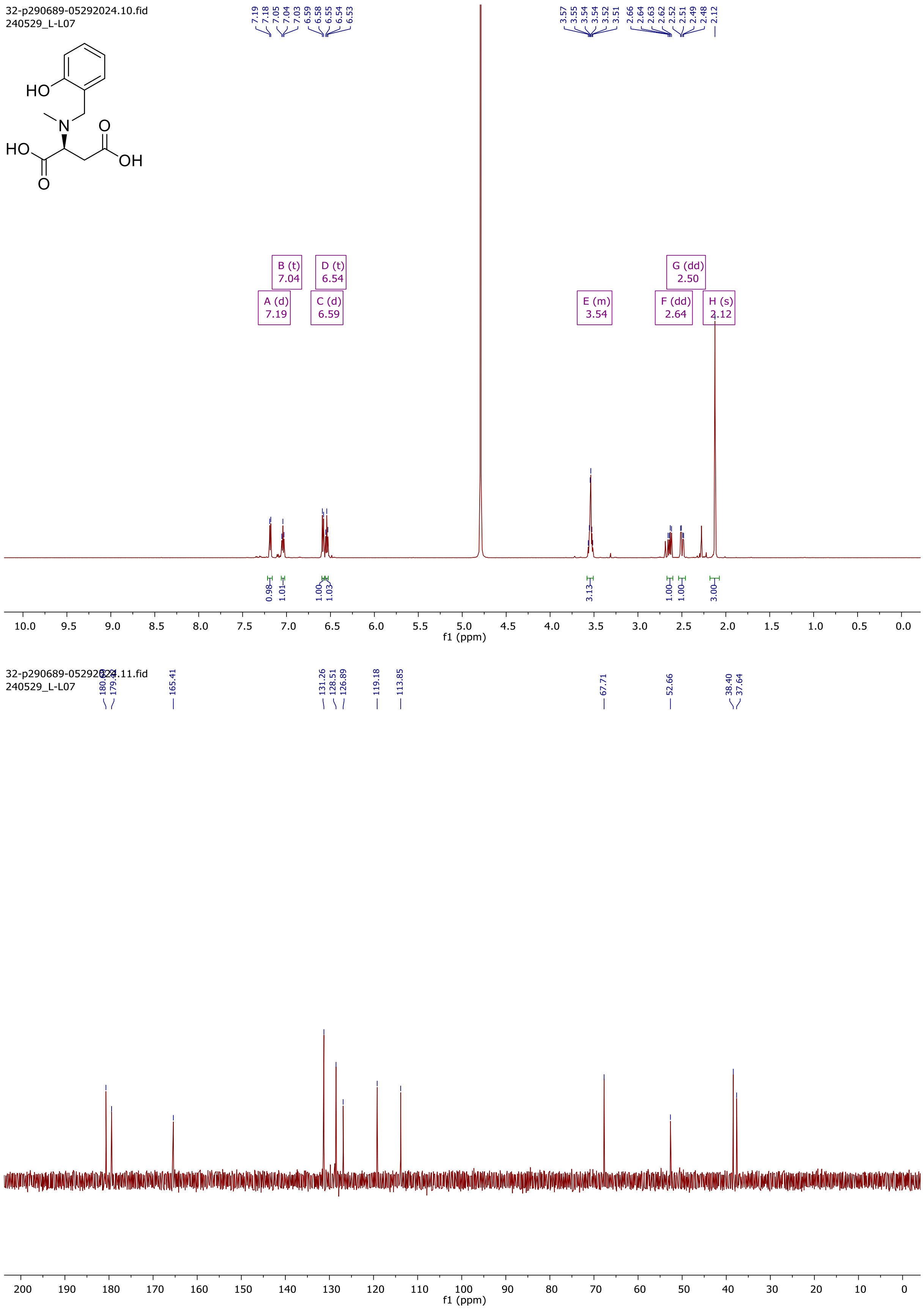

Figure S51. ^{1}H NMR (top) and ^{13}C NMR (bottom) of *N*-(2-hydroxybenzyl)-*N*-methyl-*L*-aspartic acid (**(*S*)-5d**) in 0.1 M NaOD/D_2O

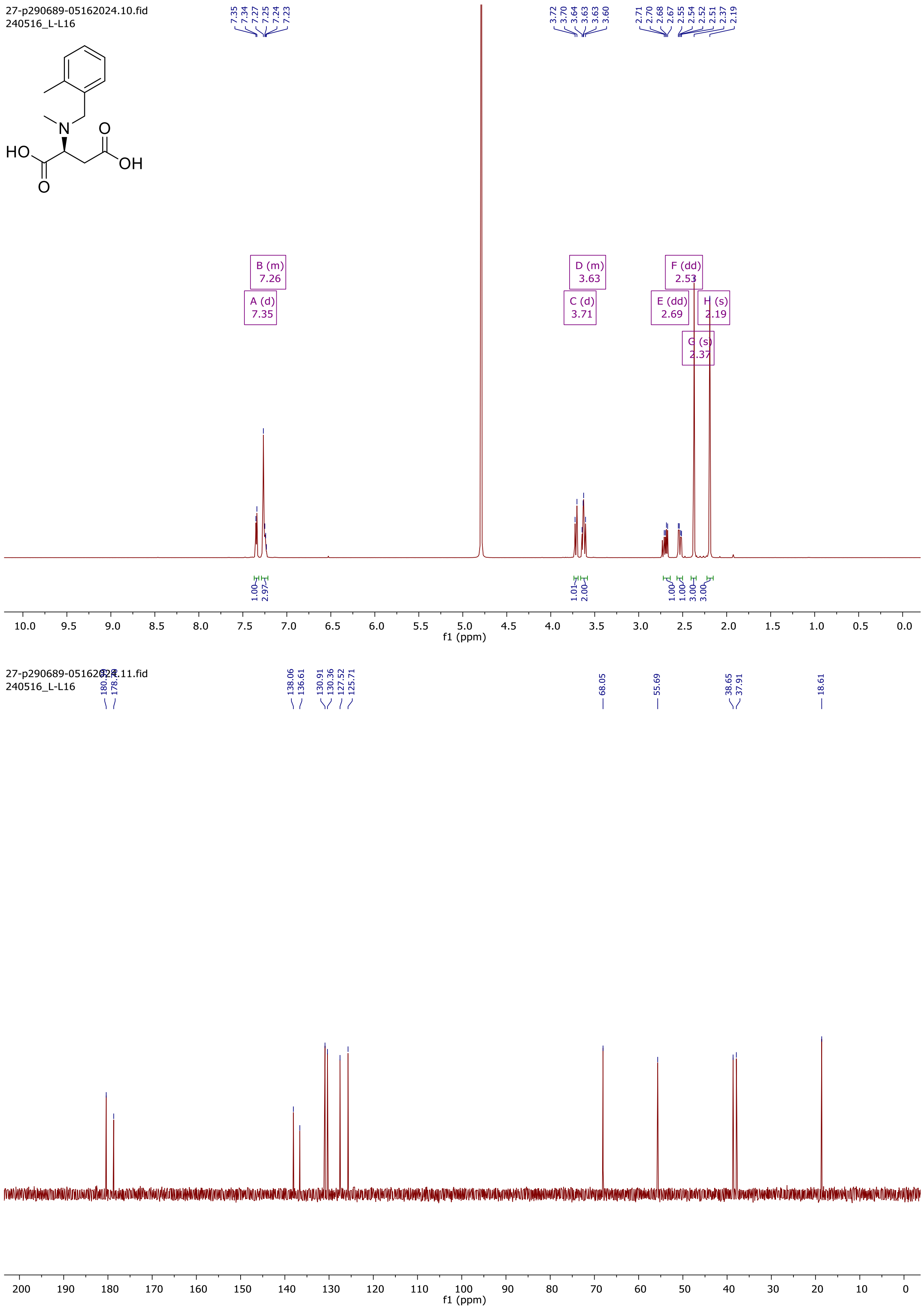

Figure S52. ^{1}H NMR (top) and ^{13}C NMR (bottom) of *N*-methyl-*N*-(2-methylbenzyl)-*L*-aspartic acid ((***S***)**-5h**) 0.1 M NaOD/D_2O

IV Chiral HPLC data

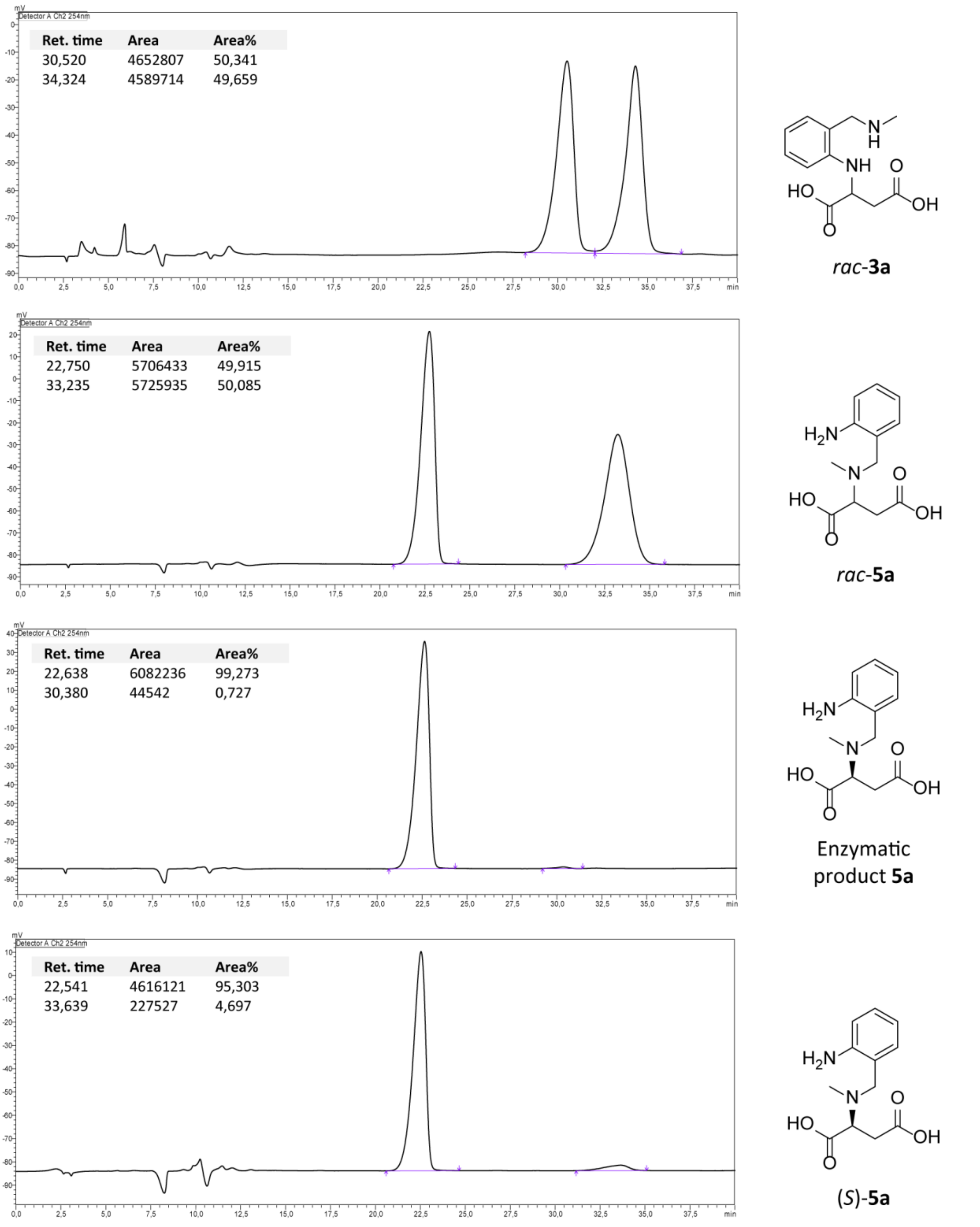

Figure S53. Chiral HPLC analysis of product **5a**. Chiral HPLC conditions: Chirex 3126 (*D*)-penicillamine column (250 x 4.6 mm, Phenomenex), with 2.0 mM aqueous $CuSO_4$ as mobile phase at a flow rate of 1.0 mL/min, 50 °C, and UV detection at 254 nm.

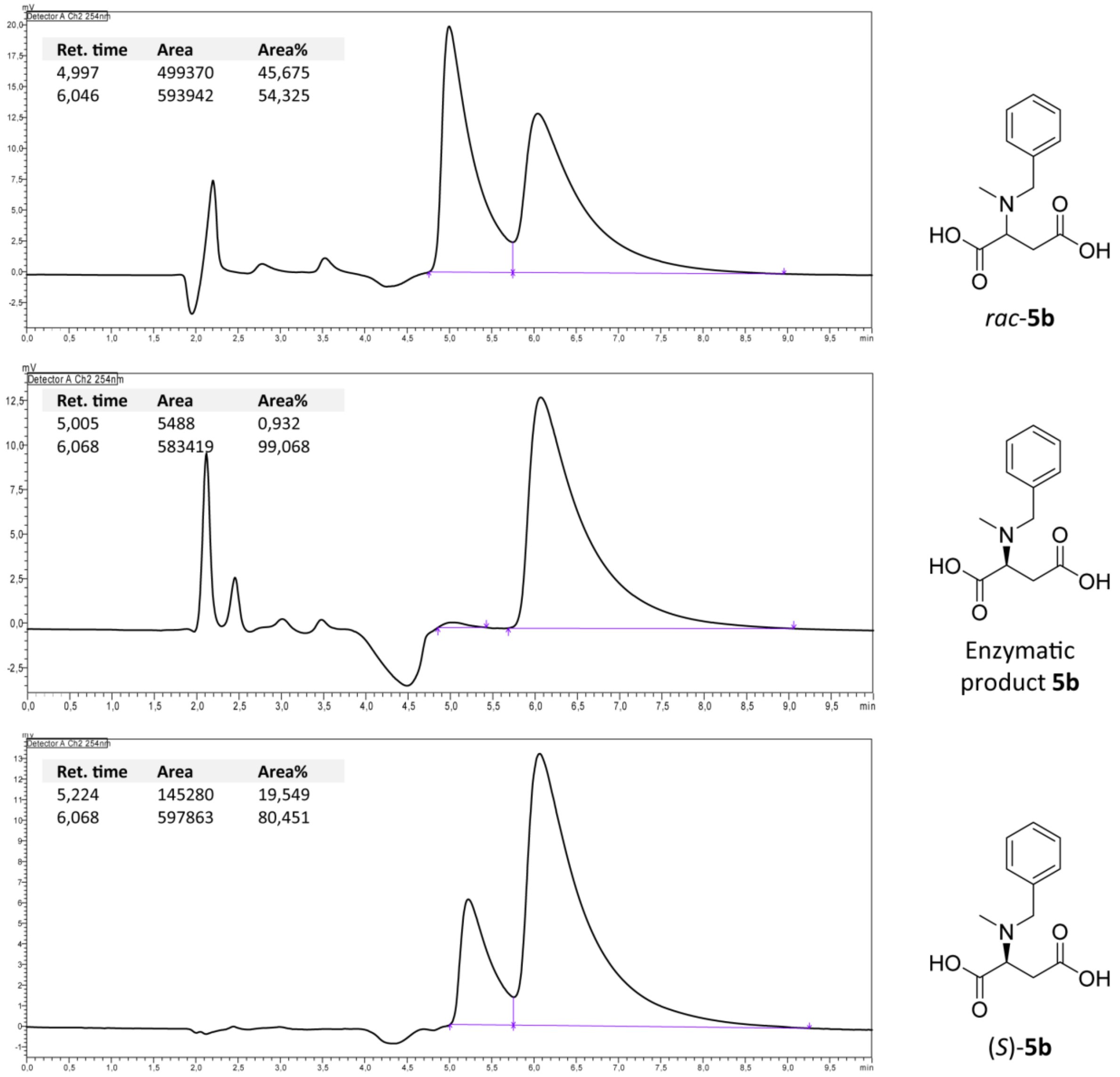

Figure S54. Chiral HPLC analysis of product **5b**. Chiral HPLC conditions: Nucleosil Chiral-1 column (5 µm, 250 x 4 mm, Macherey-Nagel), with 0.5 mM aqueous $CuSO_4$ as mobile phase at a flow rate of 1.0 mL/min, 60 °C, and UV detection at 254 nm.

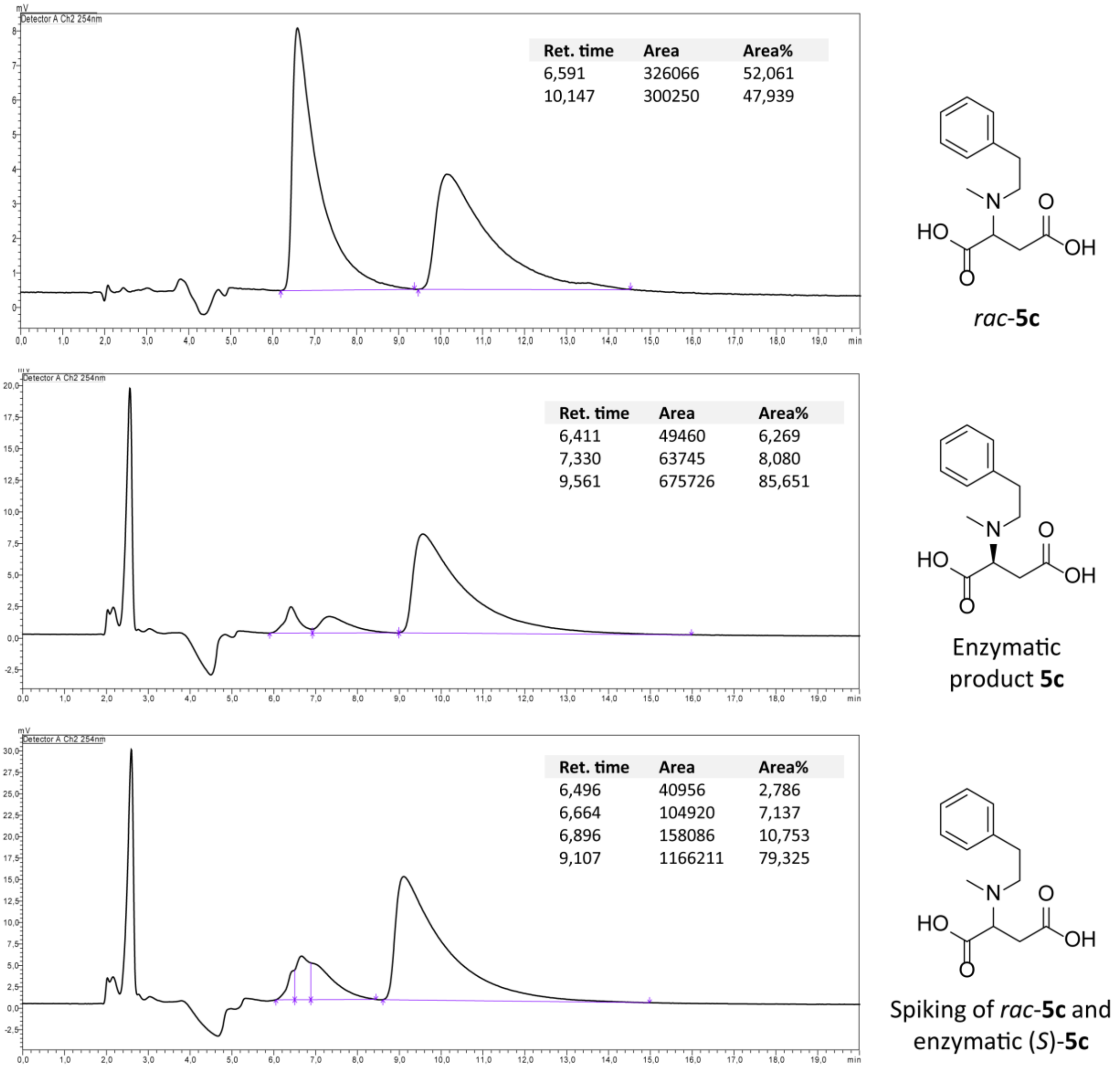

Figure S55. Chiral HPLC analysis of product **5c**. Chiral HPLC conditions: Nucleosil Chiral-1 column (5 µm, 250 x 4 mm, Macherey-Nagel), with 0.5 mM aqueous $CuSO_4$ as mobile phase at a flow rate of 1.0 mL/min, 60 °C, and UV detection at 254 nm.

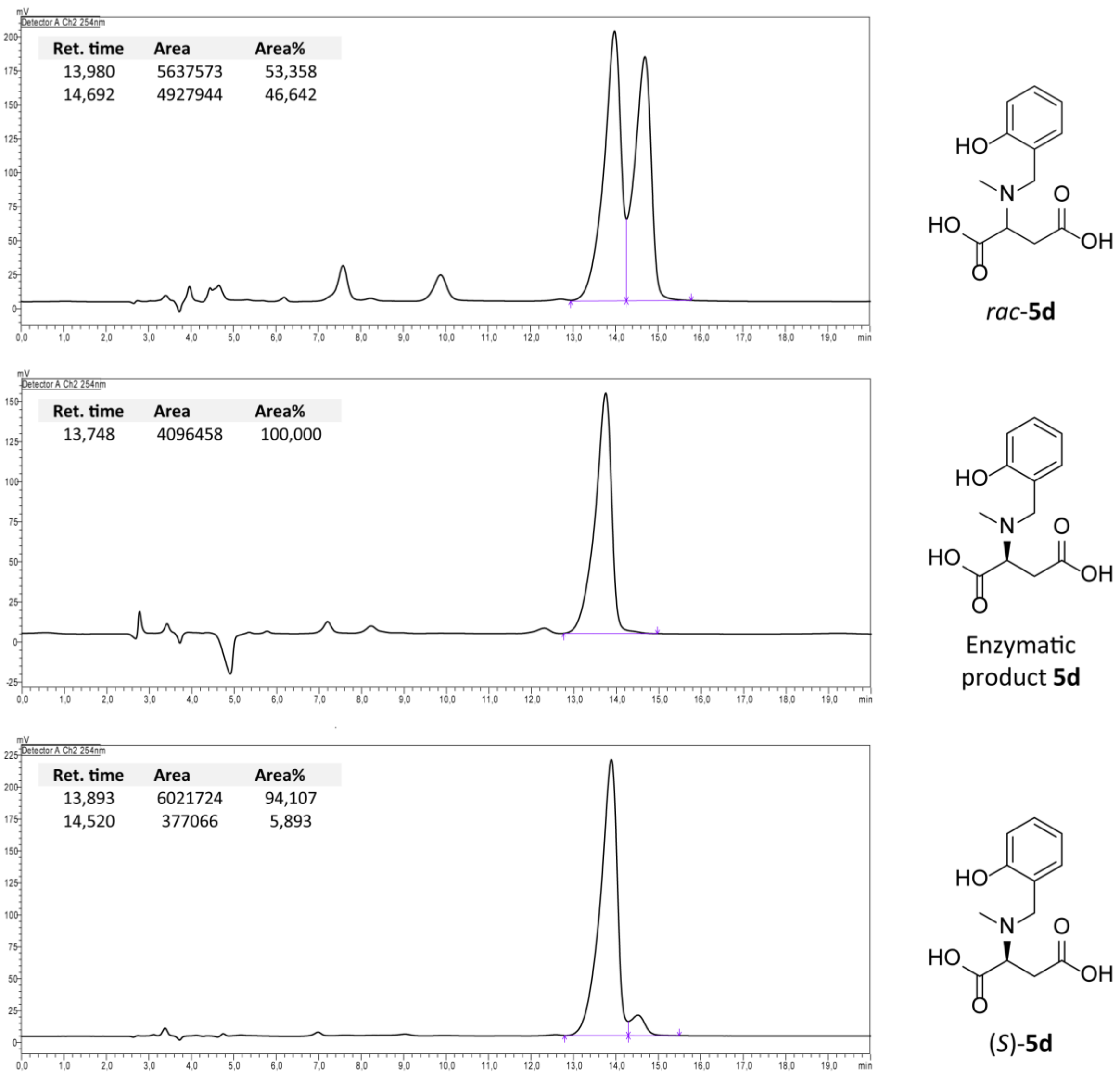

Figure S56. Chiral HPLC analysis of product **5d**. Chiral HPLC conditions: Chirex 3126 (*D*)-penicillamine column (250 x 4.6 mm, Phenomenex). Phase A: 2.0 mM aqueous $CuSO_4$, phase B: isopropanol, 10% v/v as mobile phase at a flow rate of 1.0 mL/min, 50 °C, and UV detection at 254 nm.

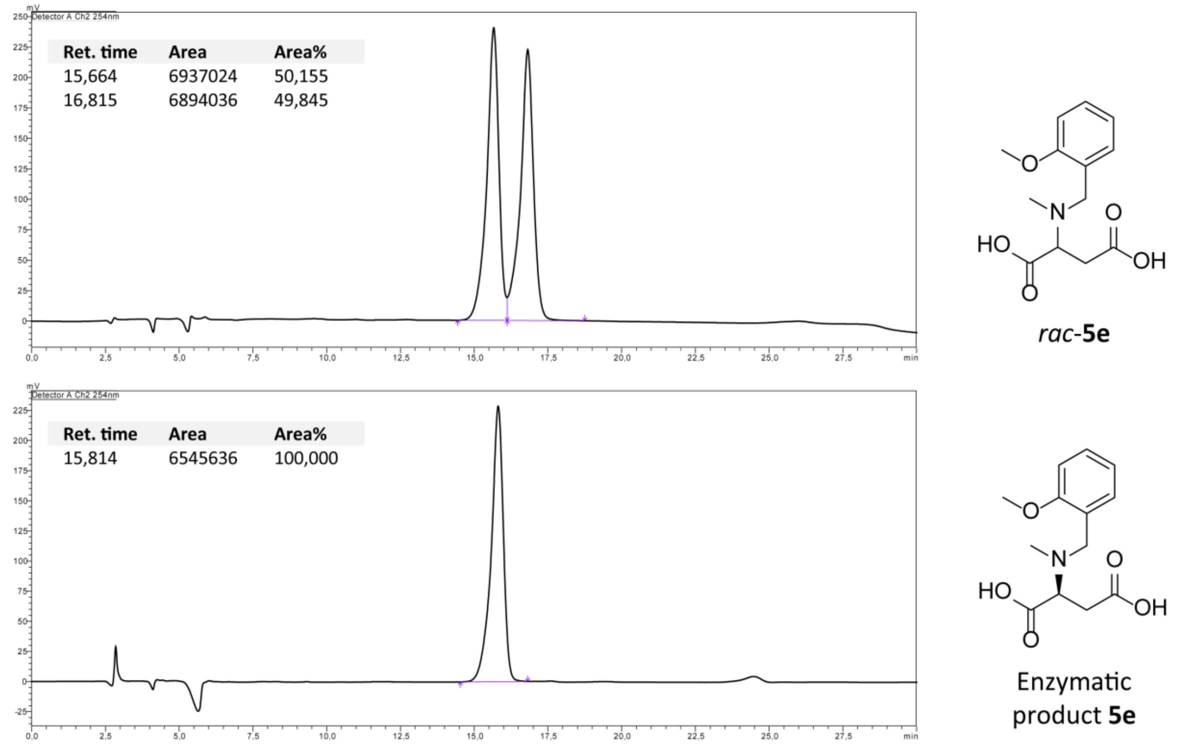

Figure S57. Chiral HPLC analysis of product **5e**. Chiral HPLC conditions: Chirex 3126 (*D*)-penicillamine column (250 x 4.6 mm, Phenomenex). Phase A: 2.0 mM aqueous $CuSO_4$, phase B: isopropanol, 5% v/v as mobile phase at a flow rate of 1.0 mL/min, 50 °C, and UV detection at 254 nm.

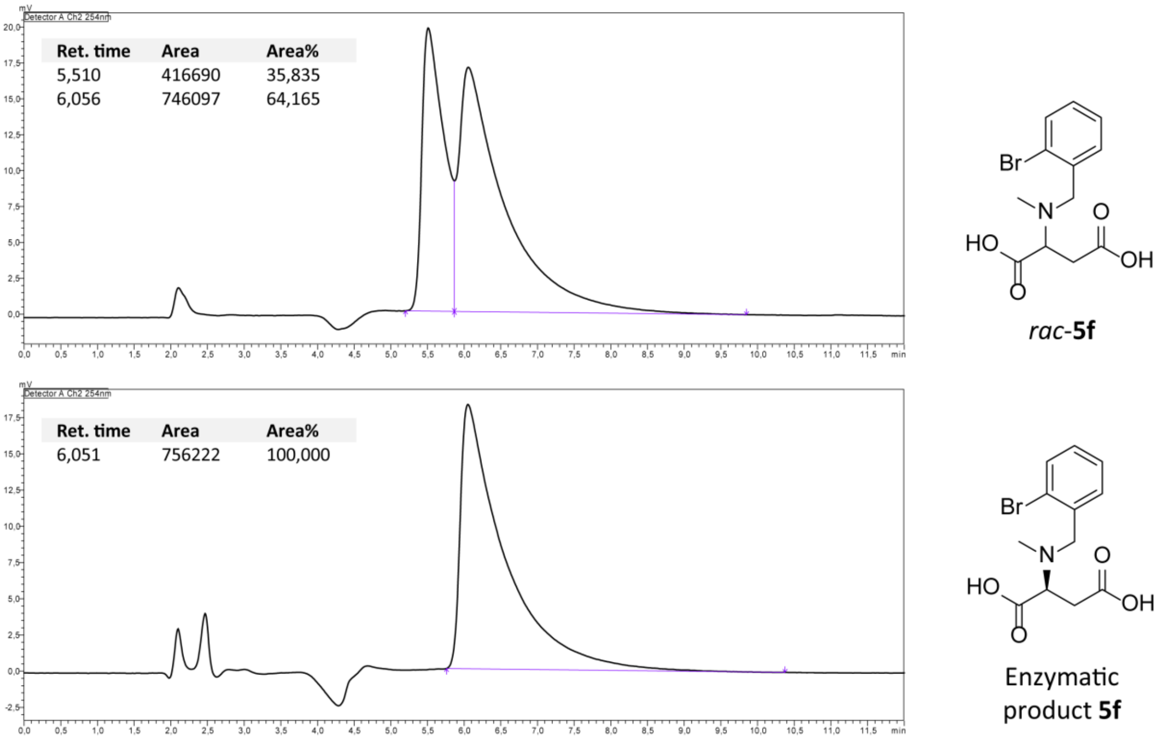

Figure S58. Chiral HPLC analysis of product **5f**. Chiral HPLC conditions: Nucleosil Chiral-1 column (5 µm, 250 x 4 mm, Macherey-Nagel), with 0.5 mM aqueous $CuSO_4$ as mobile phase at a flow rate of 1.0 mL/min, 60 °C, and UV detection at 254 nm.

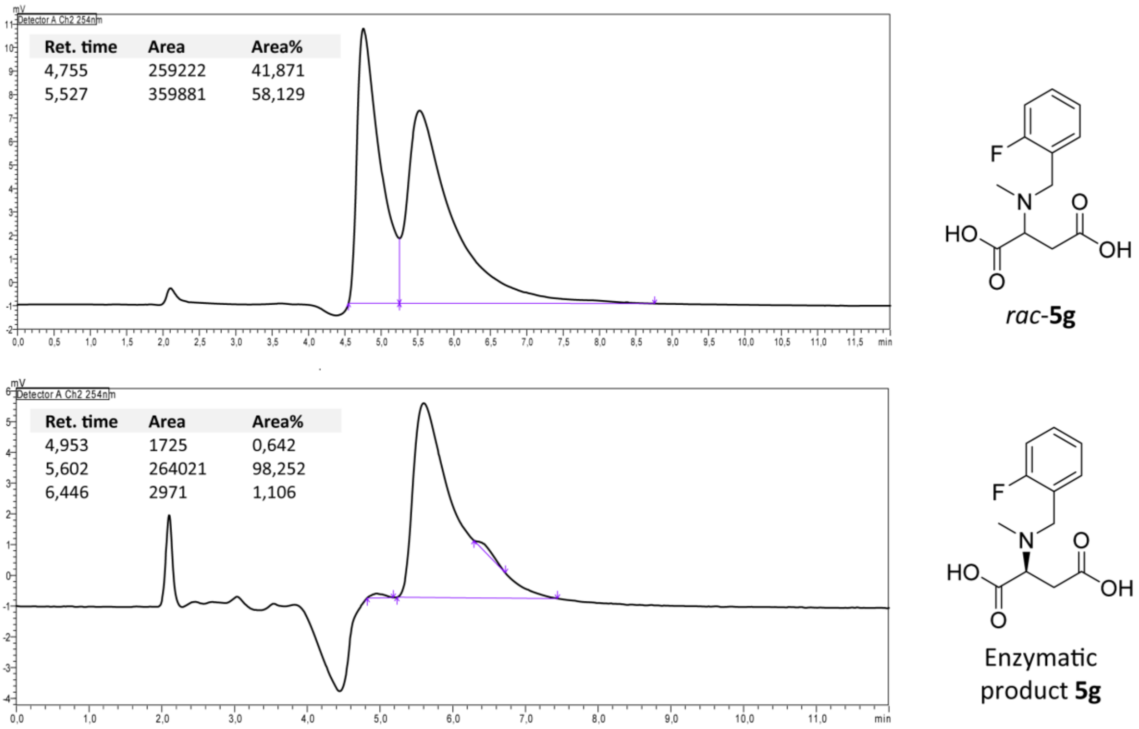

Figure S59. Chiral HPLC analysis of product **5g**. Chiral HPLC conditions: Nucleosil Chiral-1 column (5 µm, 250 x 4 mm, Macherey-Nagel), with 0.5 mM aqueous $CuSO_4$ as mobile phase at a flow rate of 1.0 mL/min, 60 °C, and UV detection at 254 nm.

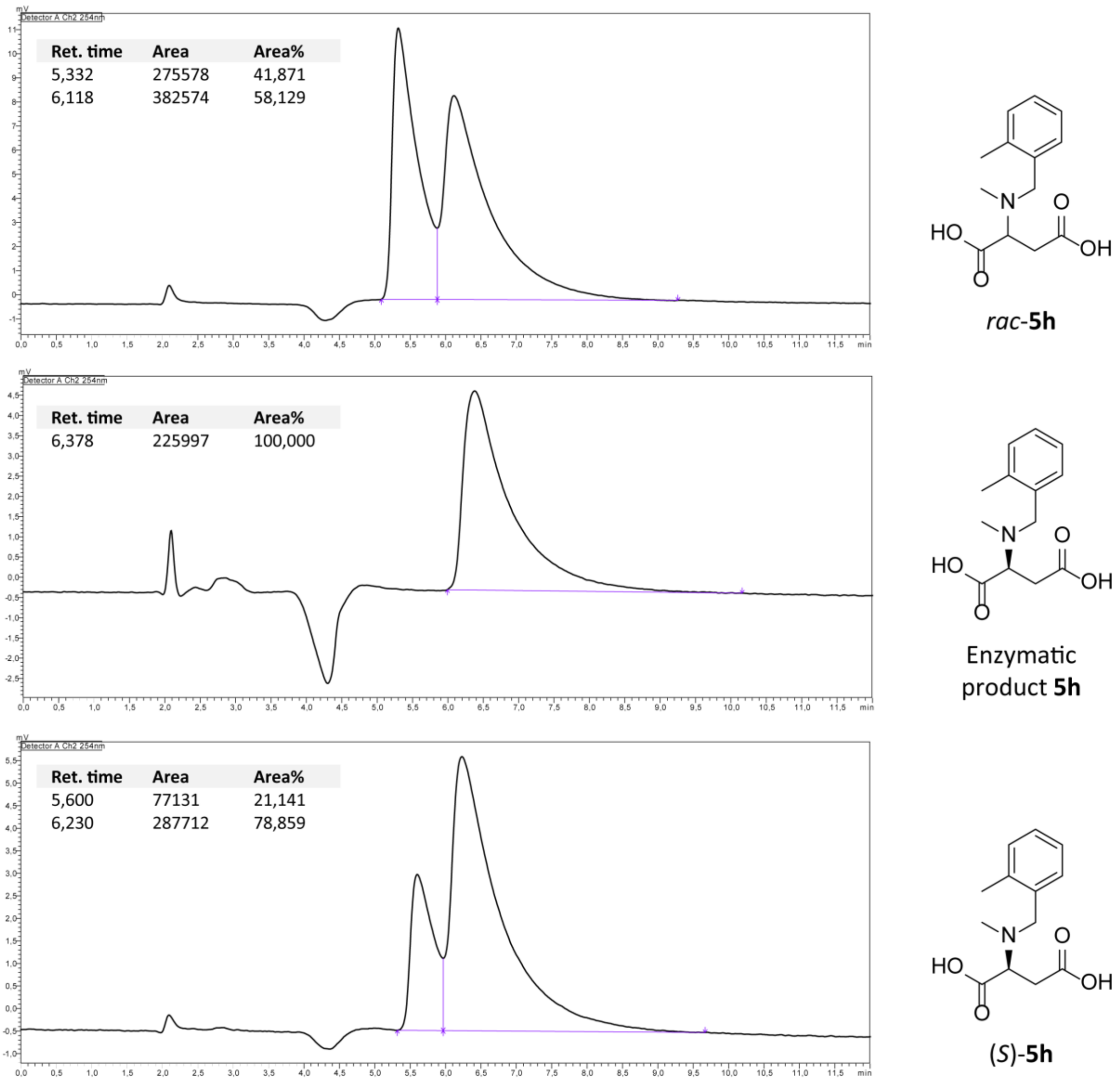

Figure S60. Chiral HPLC analysis of product **5h**. Chiral HPLC conditions: Nucleosil Chiral-1 column (5 µm, 250 x 4 mm, Macherey-Nagel), with 0.5 mM aqueous $CuSO_4$ as mobile phase at a flow rate of 1.0 mL/min, 60 °C, and UV detection at 254 nm.

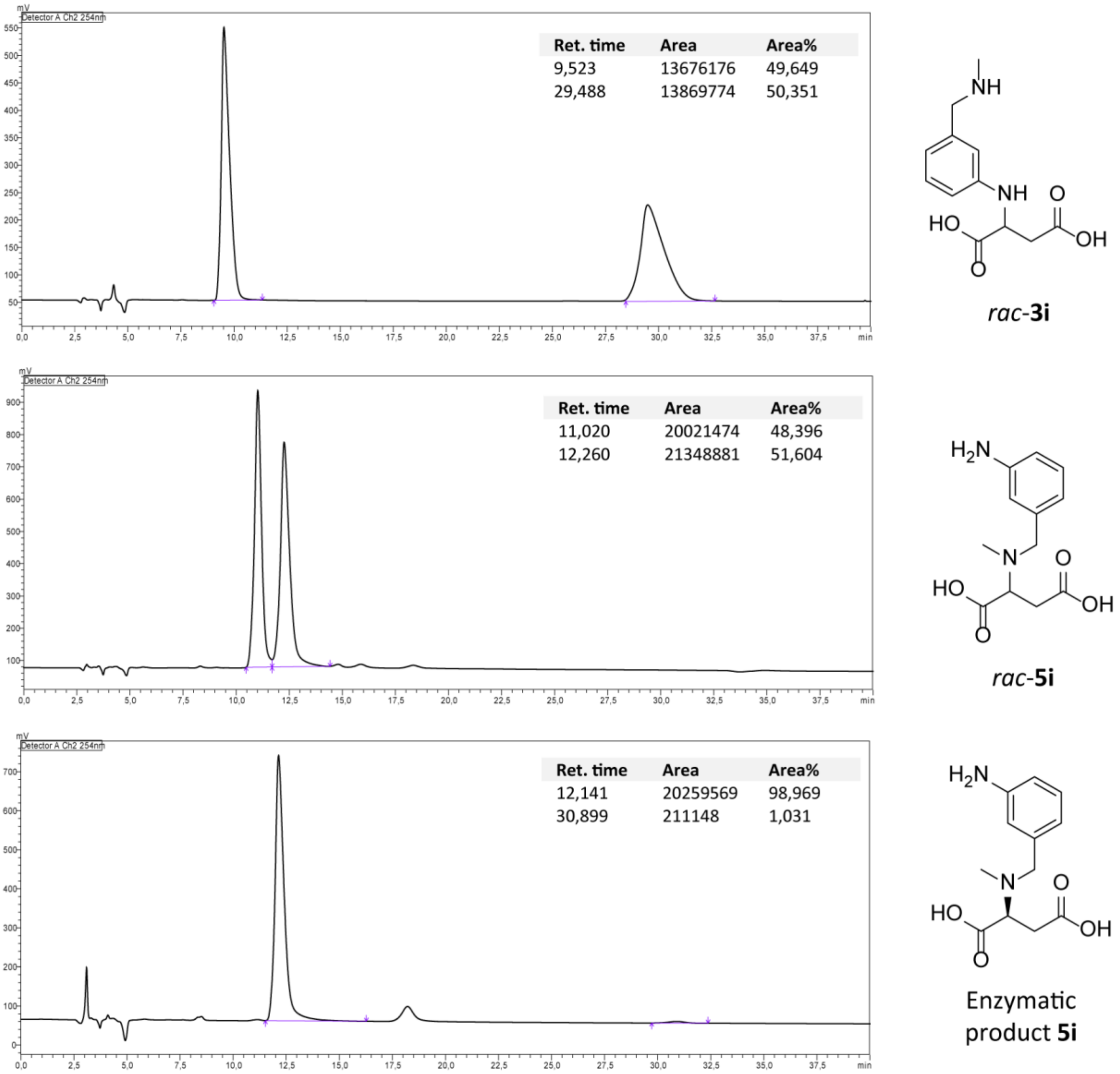

Figure S61. Chiral HPLC analysis of product **5i**. Chiral HPLC conditions: Chirex 3126 (*D*)-penicillamine column (250 x 4.6 mm, Phenomenex). Phase A: 2.0 mM aqueous $CuSO_4$, phase B: isopropanol, 10% v/v as mobile phase at a flow rate of 1.0 mL/min, 50 °C, and UV detection at 254 nm.

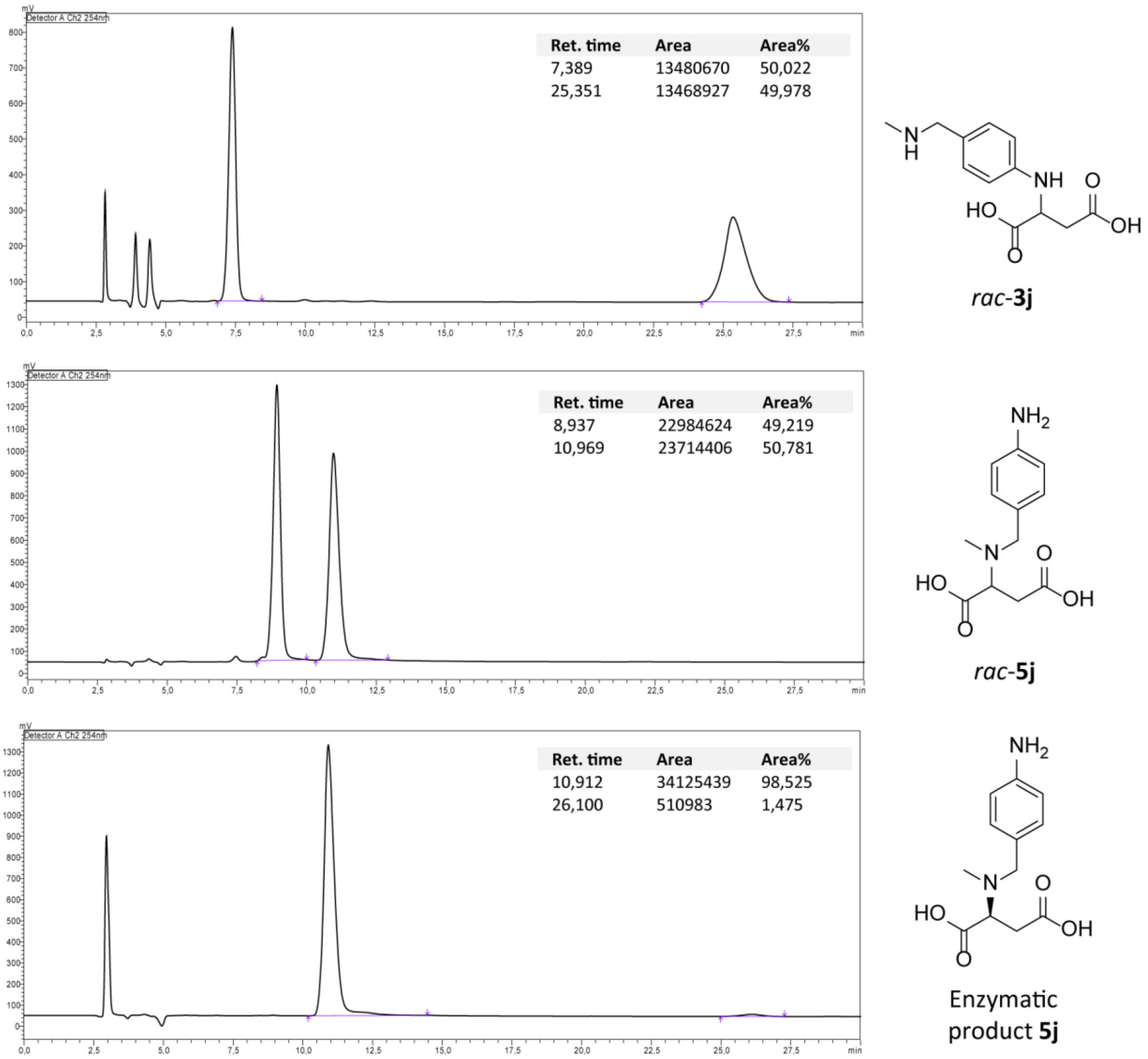

Figure S62. Chiral HPLC analysis of product **5j**. Chiral HPLC conditions: Chirex 3126 (*D*)-penicillamine column (250 x 4.6 mm, Phenomenex). Phase A: 2.0 mM aqueous $CuSO_4$, phase B: isopropanol, 10% v/v as mobile phase at a flow rate of 1.0 mL/min, 50 °C, and UV detection at 254 nm.

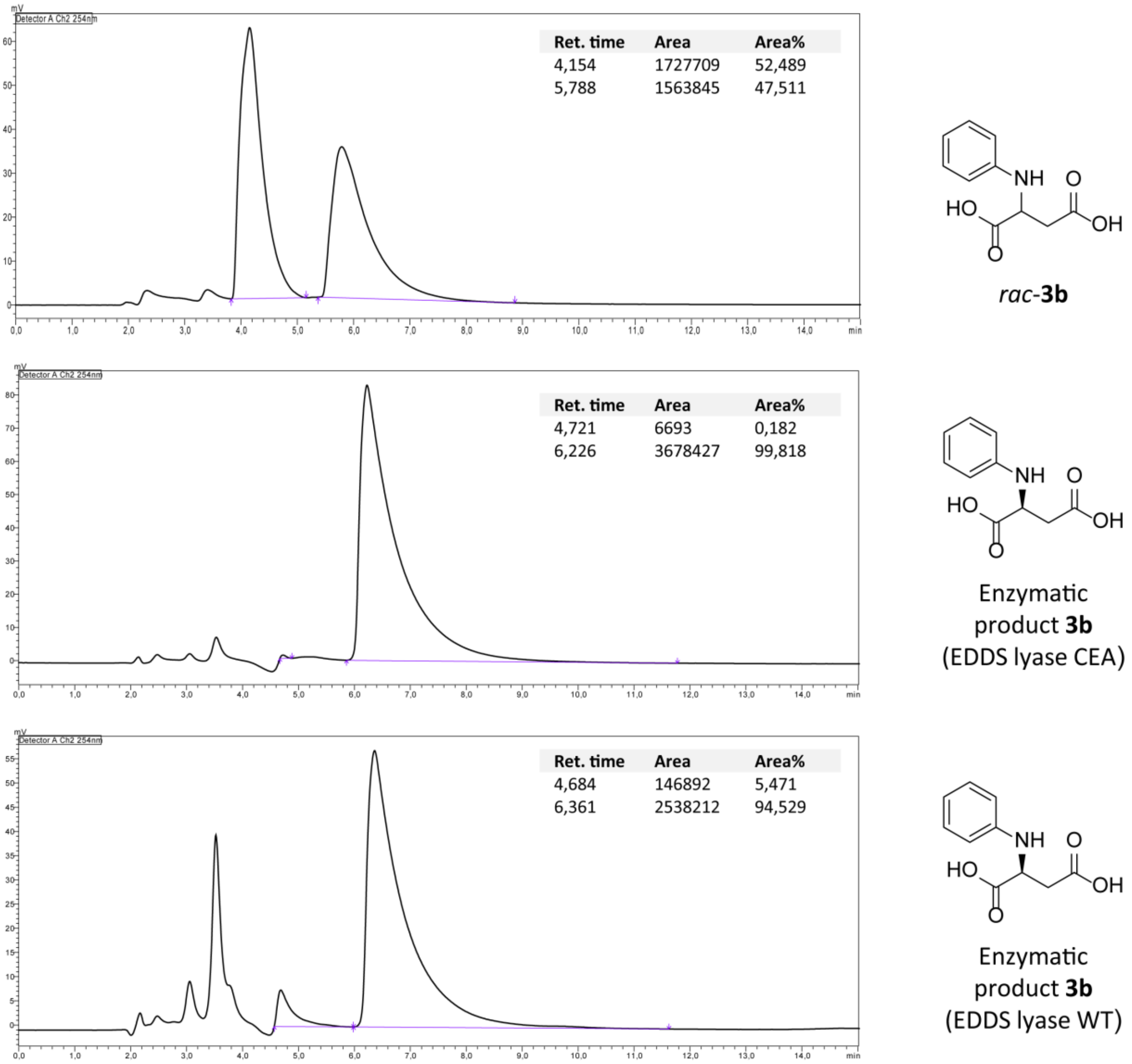

Figure S63. Chiral HPLC analysis of product **3b**. Chiral HPLC conditions: Nucleosil Chiral-1 column (5 µm, 250 x 4 mm, Macherey-Nagel), with 0.5 mM aqueous $CuSO_4$ as mobile phase at a flow rate of 1.0 mL/min, 60 °C, and UV detection at 254 nm.

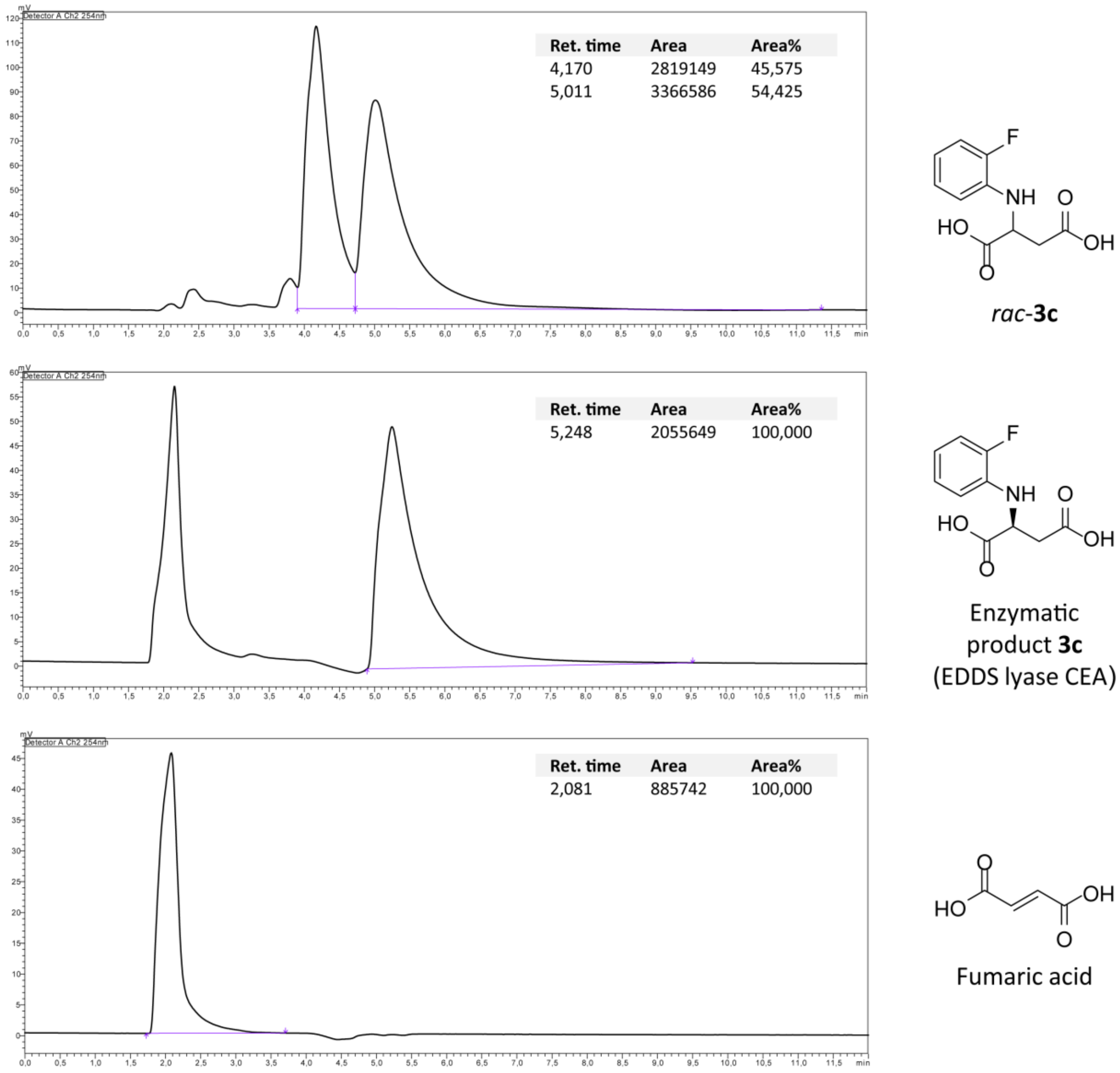

Figure S64. Chiral HPLC analysis of product **3c**. Chiral HPLC conditions: Nucleosil Chiral-1 column (5 µm, 250 x 4 mm, Macherey-Nagel), with 0.5 mM aqueous $CuSO_4$ as mobile phase at a flow rate of 1.0 mL/min, 60 °C, and UV detection at 254 nm.

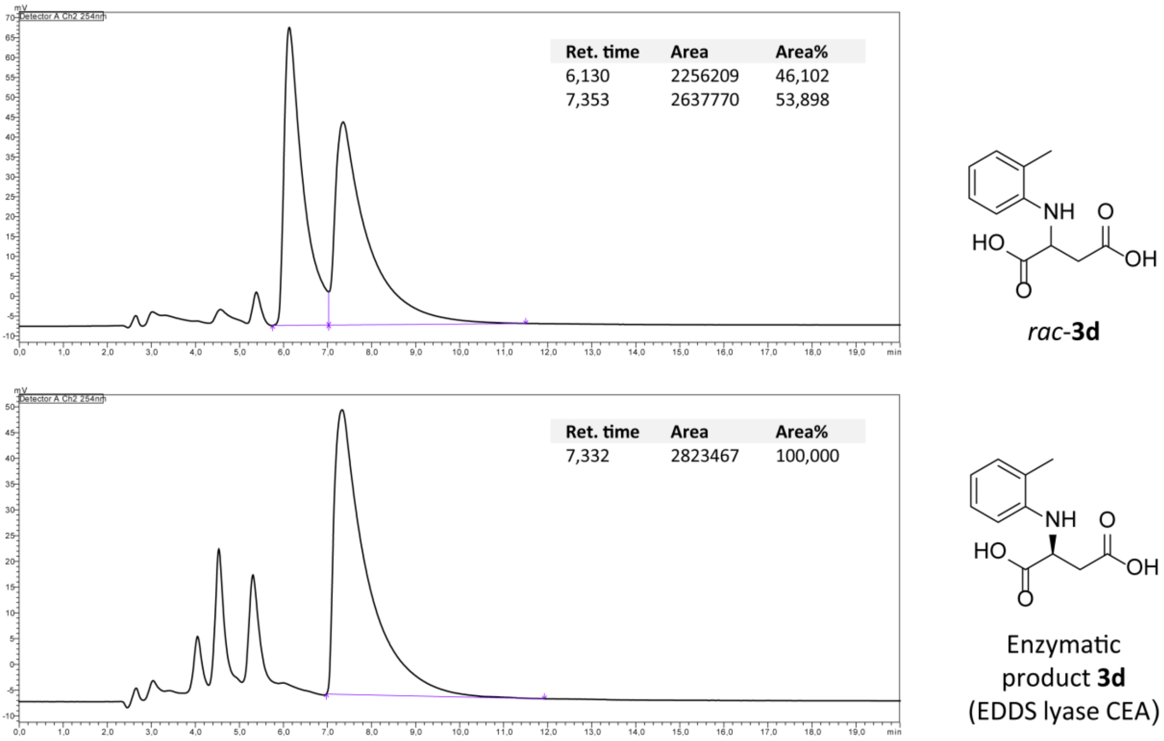

Figure S65. Chiral HPLC analysis of product **3d**. Chiral HPLC conditions: Nucleosil Chiral-1 column (5 µm, 250 x 4 mm, Macherey-Nagel), with 0.5 mM aqueous $CuSO_4$ as mobile phase at a flow rate of 0.8 mL/min, 60 °C, and UV detection at 254 nm.

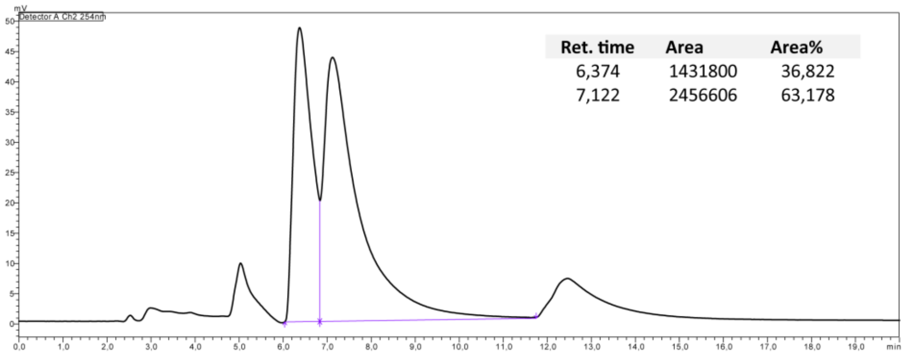

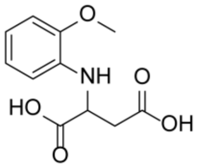

rac-**3e**

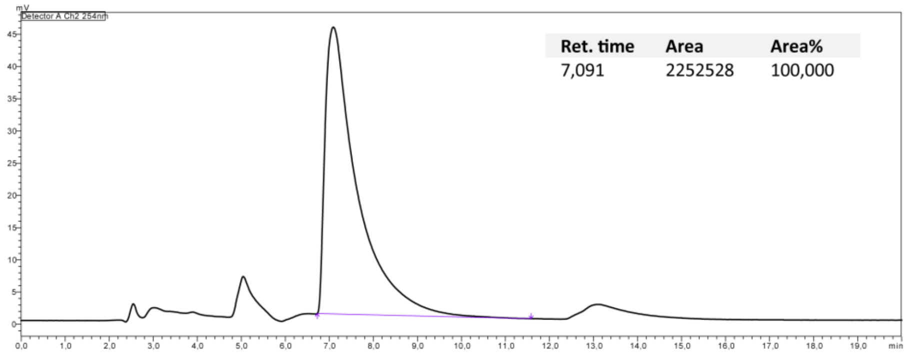

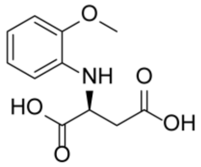

Enzymatic
product **3e**
(EDDS lyase CEA)

Figure S66. Chiral HPLC analysis of product **3e**. Chiral HPLC conditions: Nucleosil Chiral-1 column (5 µm, 250 x 4 mm, Macherey-Nagel), with 0.5 mM aqueous $CuSO_4$ as mobile phase at a flow rate of 0.8 mL/min, 60 °C, and UV detection at 254 nm.

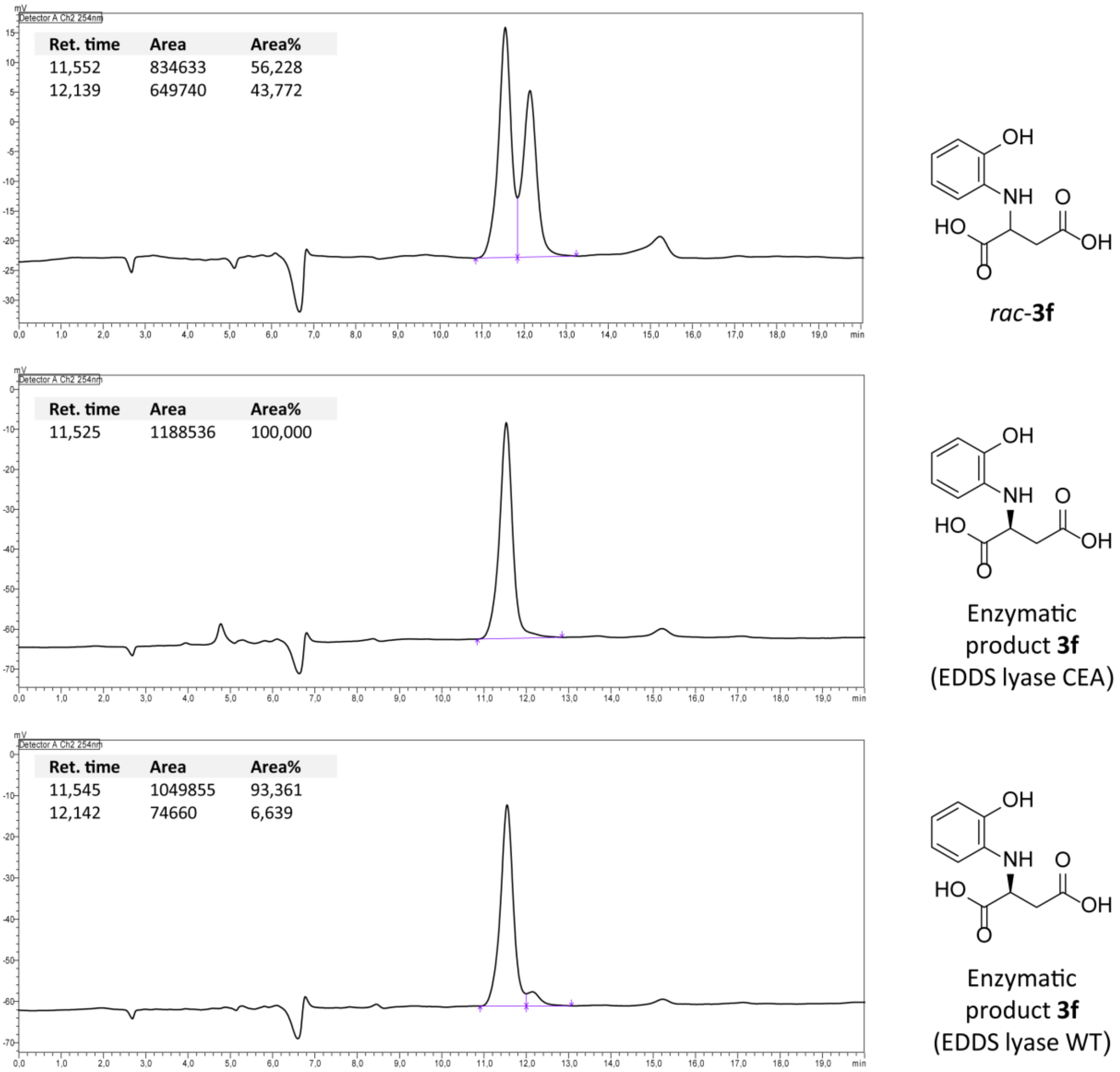

Figure S67. Chiral HPLC analysis of product **3f**. Chiral HPLC conditions: Chirex 3126 (*D*)-penicillamine column (250 x 4.6 mm, Phenomenex). Phase A: 2.0 mM aqueous $CuSO_4$, phase B: isopropanol, 2% v/v as mobile phase at a flow rate of 1.0 mL/min, 50 °C, and UV detection at 254 nm.

V References

[1] H. Poddar, J. de Villiers, J. Zhang, V. Puthan Veetil, H. Raj, A. W. H. Thunnissen, G. J. Poelarends, *Biochemistry* **2018**, *57* (26), 3752-3763.

[2] A. M. Waterhouse, J. B. Procter, D. M. Martin, M. Clamp, G. J. Barton, *Bioinformatics* **2009**, *25* (9), 1189-1191.

[3] H. Fu, A. Prats Luján, L. Bothof, J. Zhang, P. G. Tepper, G. J. Poelarends, *ACS Catal* **2019**, *9* (8), 7292-7299.

[4] M. F. Bhat, A. Prats Luján, M. Saifuddin, G. J. Poelarends, *ACS Catal* **2022**, *12* (18), 11421-11427.

[5] I. P. Andrews, R. J. Atkins, G. F. Breen, J. S. Carey, M. A. Forth, D. O. Morgan, A. Shamji, A. C. Share, S. A. C. Smith, T. C. Walsgrove, A. S. Wells, *Organic Process Research & Development* **2003**, *7* (5), 655−662.

[6] C.-E. Yeom, M. J. Kim, B. M. Kim, *Tetrahedron* **2007**, *63* (4), 904-909.

[7] L. Xi, D. Wu, H.-Y. Zhu, C.-H. Zhang, Y. Jin, J. Lin, *Curr Org Synth* **2015**, *12* (2), 197-201.
